# Expression Profiling Identifies TWIST2 Target Genes in Setleis Syndrome Patient Fibroblast and Lymphoblast Cells †

**DOI:** 10.3390/ijerph18041997

**Published:** 2021-02-19

**Authors:** Noe E. Crespo, Alexandra Torres-Bracero, Jessicca Y. Renta, Robert J. Desnick, Carmen L. Cadilla

**Affiliations:** 1 Department of Biochemistry, School of Medicine, University of Puerto Rico, Medical Sciences Campus, San Juan 00936, Puerto Rico; noe.crespo@upr.edu (N.E.C.); alexandra.torres9@upr.edu (A.T.-B.); jessicca.renta@upr.edu (J.Y.R.); 2 Department of Genetics and Genomic Sciences, Icahn School of Medicine at Mount Sinai, New York, NY 10029, USA; robert.desnick@mssm.edu

**Keywords:** focal facial dermal dysplasia, SS, TWIST2, bHLH, transcription factor, E-box, microarray, differential expression

## Abstract

*Background*: Setleis syndrome (SS) is a focal facial dermal dysplasia presenting with bilateral temporal skin lesions, eyelash abnormalities and absent meibomian glands. SS is a rare autosomal recessive disorder caused by mutations in the TWIST2 gene, which codes for a transcription factor of the bHLH family known to be involved in skin and facial development. *Methods*: We obtained gene expression profiles by microarray analyses from control and SS patient primary skin fibroblast and lymphoblastoid cell lines. *Results*: Out of 983 differentially regulated genes in fibroblasts (fold change ≥ 2.0), 479 were down-regulated and 509 were up-regulated, while in lymphoblasts, 1248 genes were down-regulated and 73 up-regulated. RT-PCR reactions confirmed altered expression of selected genes. *Conclusions*: TWIST2 is described as a repressor, but expression profiling suggests an important role in gene activation as well, as evidenced by the number of genes that are down-regulated, with a much higher proportion of down-regulated genes found in lymphoblastoid cells from an SS patient. As expected, both types of cell types showed dysregulation of cytokine genes. These results identify potential TWIST2 target genes in two important cell types relevant to rare disorders caused by mutations in this bHLH gene.

## 1. Introduction

Setleis syndrome (SS, MIM #227260) is a very rare autosomal recessive disorder. This disease is classified under the focal facial dermal dysplasias, which are disorders characterized by bitemporal or preauricular skin lesions resembling aplasia cutis congenita, affecting the development of hair, teeth, nails and/or skin. SS was first clinically described in patients of Puerto Rican ancestry [1] who presented with bilateral temporal skin lesions, absent eyelashes, an abnormal pattern of hair growth and absent meibomian glands [1,2,3]. Other studies identified SS patients with diverse lineages, such as European, Chinese/English/Italian, German, Omani and Canadian [4,5,6,7,8]. Molecular genetic studies first identified the SS [9] locus on chromosome 2q37.3 and nonsense mutations in the gene that codes for the TWIST2 bHLH transcription factor, i.e., c.486C > T (p.Gln119*) and c.324C > T (p.Gln65*), in Puerto Rican (PR) and Omani SS patients, respectively. The p.Gln119* and p.Gln65* nonsense mutations code for TWIST2 mutant proteins that lack the C-terminal region or both the bHLH and C-terminal regions, respectively, thus preventing dimer formation mediated by the C-terminal or HLH residues, affecting transcriptional regulation of target genes [9]. To validate these findings, a Twist2-knock-out mouse [10] was re-examined and shown to have phenotypic similarities to SS patients, particularly skin, hair and eye abnormalities, as well as previously reported spleen abnormalities, and adipose tissue deficiencies [9]. 

**Figure 1:**
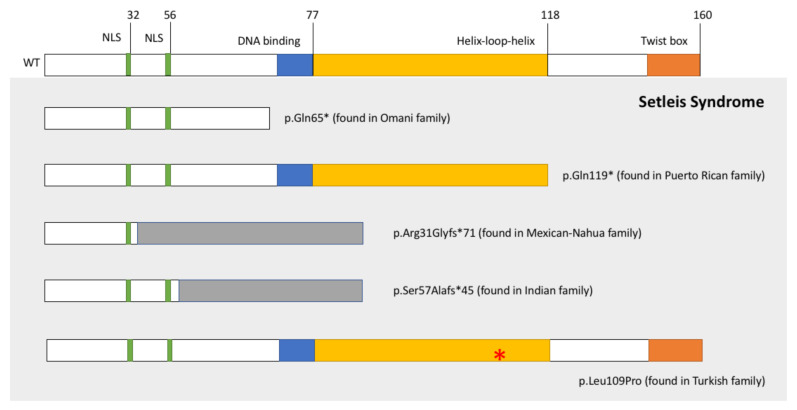
The schematic domain structure of TWIST2 protein and the location of TWIST2 mutations identified in SS patients. NLS, nuclear localization signal. Since the original report by Tukel et al., five different homozygous mutations in the TWIST2 gene have been identified p.Gln65*-Omani, p.Gln119*-Puerto Rican, p.Arg31Glyfs*71-Mexican-Nahua, p.Ser57Alafs*45-Indian and p.Leu109Pro-Turkish families. All five TWIST2 mutations are suggested to alter dimer formation. Grey bars represent regions in the proteins predicted to have a different amino acid sequence than the wild type protein.

Subsequently, severe frameshift and missense mutations in TWIST2 have been found in Mexican-Nahua (pS57AfsX45), Indian (pR31GfsX71), and Turkish (p.Leu109Pro) SS patients (Figure 1, [11,12,13], with the characteristic features of the syndrome. Copy number variants in chromosome 1p36 [14,15], may cause SS, and developmental delay or intellectual disabilities in some patients. Heterozygotes with severe TWIST2 mutations found in SS patients that eliminate the both the bHLH and C-terminal domains may have syndromic manifestations [7,11,13], such as mild dysmorphic features and only bitemporal scars (p.Gln65*, [7]) or distichiasis of the upper eyelids and partial absence of lower eyelids (pS57AfsX45, [11]). Carriers of helix disrupting TWIST2 mutations may have prominent jaw and chin, laterally sparsed eyebrows and redundant facial skin (p.Leu109Pro, [13]). Mutations in conserved residues of the basic region of TWIST2, which is involved in DNA binding, cause two related autosomal dominant syndromes, Barber Say (BSS, p.Glu75Gln, p.Glu75Ala and p.Gln77_Arg78dup) and Ablepharon Macrostomia (AMS, p.Glu75Lys) [16]. The phenotypic manifestations of SS, BSS and AMS are similar, which suggest that these syndromes form a continuum. The BSS and AMS patients have more severe eyelid malformations and eye manifestations not frequently reported in SS patients (such as strabismus and nystagmus) [17].

The TWIST2 basic helix-loop-helix (bHLH) transcription factor is 160 amino acid long and its expression pattern suggests that it functions as a regulator of gene expression in a subset of mesenchymal cell lineages including developing dermis [18,19]. Amino acid sequence analysis shows three main domains: a nuclear localization signal (residues 29–32 and 52–56), the bHLH domain (residues 66–118) and the Twist box (residues 141–160, [20]). The Twist box is only found in TWIST1 and TWIST2 proteins and interaction with it has been shown to inhibit RUNX2, MEF2, p53, ATF4 and RelA function [10,20,21,22,23]. The bHLH domain is found in a large group of transcription factors known as the bHLH transcription superfamily, which includes over 125 unique proteins in the human genome that are further sub-divided into Classes A to F [24]. Class A includes ubiquitous factors (e.g., TCF3-E12 and E47), and time- and tissue-specific factors like the members of the TWIST subfamily (TWIST-1 and -2, HAND-1 and -2, PARAXIS and SCLERAXIS), among others [25]. The basic domain allows specific binding to DNA sequences known as E-boxes (5′- CANNTG-3′). Dimerization is a key characteristic of the bHLH transcription factors, mostly mediated by the HLH region. The bHLH protein monomers have a distinctive affinity to E-box half-sites (e.g., 5′-CAT or 5′-CAG) [26]. Therefore, pairing Class A ubiquitous and tissue-specific bHLH proteins in heterodimeric complexes, or as homodimers, can result in a large number of combinations of regulatory factors. Bioinformatic analyses identified a signature motif in TWIST1 and TWIST2 proteins [27], which may function redundantly [28]. It has been estimated that, genome-wide, one canonical E-box sequence can be found every 128 nucleotides [29].

The biological functions of TWIST2 involve cell lineage differentiation, particularly in skin, myoblast and osteoblast development. Gong and Li [30] showed that TWIST2 can repress myogenesis by altering MyoD binding to the MCK gene. Zhang et al. [31] reported that TWIST2 inhibits osteocalcin transcription and may interact or modulate the RUNX2 promoter, genes whose expression is needed for osteoblast maturation. Both myoblast and osteoblast cells are derived from mesenchymal stem cells (MSC), which are characterized by high levels of TWIST2 expression. Therefore, TWIST2 has been proposed to negatively regulate genes to maintain a MSC state, and after crucial tissue specific-stimuli allows transition to the next differentiation state, where genes previously repressed may be activated [11,27,28]. In the present study, we aimed to identify potential TWIST2 target genes by comparing the expression profiles of cultured cells expressing wild type vs. mutant TWIST2 protein from SS patients, assuming that dysregulated target genes would be revealed in these comparisons. Since the most frequently established cell culture lines in genetic studies are primary skin fibroblasts obtained from punch biopsies [32,33] and lymphoblastoid cells from immortalized white blood cell samples [34], we used them for this purpose. Since we used two types of cultured cell lines to achieve our aim, our results are limited to the types of cell lines and the number of different human subjects used to generate the cell lines available for expression profiling. In addition, since TWIST2 expression correlates with dermal cell determination and differentiation [18], skin fibroblasts were considered a good model for investigating changes in affected cells in SS. Lymphoblastoid cells were also available for analysis and were used to compare expression profiles from two different types of human cell lines. Our expression analyses using as model systems a limited number of skin fibroblast and lymphoblastoid cell lines (LCLs) suggest that TWIST2 can function as both an activator and repressor, the magnitude of each role depending on the type of cell line under study. We would expect that cell lines derived from SS patients with a mutation that may disrupt TWIST2 dimerization, such as the p.Gln119* mutation found in Puerto Rican patients, should exhibit patterns of dysregulated genes due to incomplete or ineffective binding to target genes. Our data supports future TWIST2 studies which should consider the different roles of the transcription factor may display in the context of the biological model that is employed.

## 2. Materials and Methods 

### 2.1. Sample Collection and Primary Cell Cultures

Collection of blood and skin biopsy samples from SS patients and controls for cell culture was performed after obtaining informed consent. This study protocol was approved by the University of Puerto Rico Medical Sciences Campus Institutional Review Board (Protocol 0150198). The skin fibroblast cell cultures derived from Puerto Rican Setleis patients homozygous for the p.GlnQ119* TWIST2 mutation and controls wild type for TWIST2 were generated as previously described [9,29,32,33]. Skin fibroblast cell pellets used for total RNA isolation prior to microarrays analysis were derived from PR SS patient cell lines B1601 (passage 5) and B1602 (passage 3), from affected siblings, female and male, respectively; B1608 (passages 3 and 4) from an affected 34 years-old PR female SS patient. For normal controls, we used the B1610 cell line (passages 1 and 3), derived from an unaffected sister of the PR SS patient that provided the skin biopsy sample for the B1608 cell line; the B16X cell line (passage 2) from an unrelated PR subject; and the GM00037 cell line (passage 4), which is a human fibroblast cell line from a PR female (obtained from Coriell Cell repositories, Camden, NJ, USA). LCLs were generated from a PR SS patient homozygous for the p.GlnQ119* TWIST2 mutation (MS4883, passages 9, 14 and 19) and an unaffected sibling homozygous wild type for TWIST2 (MS4881, passages 9, 10 and 17) by transformation with Epstein-Bar Virus as described [34], grown in suspension in culture-flasks and maintained in RPMI-1640 media supplemented with 10% fetal bovine serum (FBS) (Thermo Fisher Scientific, Waltham, MA, USA) and 2 mM L-glutamine (Lonza). For lymphoblastoid cell expression profiling, we used three biological replicates from different passages of the MS4883 cell line, which was derived from the same Puerto Rican SS patient of the B1608 cell line described above. For controls, we extracted RNA from cell pellets (three biological replicates) of different passages of the MS4881 cell line, derived from the same unaffected sibling which gave rise to the B1610 cell line.

### 2.2. RNA Extraction and Integrity Determination 

RNA extractions were performed from 30–100 mg of pelleted cells using the RNeasy kit (Qiagen, Germantown, MD, USA) following the manufacturer’s instructions. Concentrations of total RNAs were measured using a NanoDrop UV-Vis spectrophotometer (Thermo Fisher Scientific, Wilmington, DE). The RNA Integrity Number (RIN) for the RNA samples were obtained using the Agilent 2100 Bioanalyzer (Agilent Technologies, Santa Clara, CA, USA) and the RNA 6000 Nano Assay. 

### 2.3. Microarray Analysis

Expression profiles of RNAs derived from Puerto Rican control and Setleis patient primary fibroblasts were examined by microarray analysis using Affymetrix 3′IVT U233plus2.0 GeneChips (NCBI GEO #GSE16524, previously reported [29]). Two GeneChips were reacted with cDNAs derived from two different passages from the B1608 PR SS patient cell line. One GeneChip was hybridized with cDNA from a 1:1 Pool of total RNA from the B16X and GM00037 PR control cell lines. For controls, one GeneChip was hybridized with cDNAs derived from a 1:1 pool of total RNA from the B1601 and B1602 PR SS patient cell lines and two other GeneChips were reacted with cDNAs derived from two different passages from the B1610 PR control cell line. Affymetrix Human Exon Array (HuEx-1_st-v2) GeneChips were hybridized with labeled sense DNA derived from total RNAs from three different passages of LCLs from a Puerto Rican control and a Setleis patient, as recommended by the manufacturer. Only one LCL of each type (PR SS vs. PR control) was used in the Exon Array comparisons, which is a limitation of this study, since we did not have several different cell lines of each type available for analysis. Arrays were washed, stained, and scanned according to the protocol described in WT Sense Target Labeling Assay Manual to generate gene expression profiles (NCBI GEO #GSE160893). The fibroblast and lymphoblastoid microarray. CEL files were analyzed using the Affymetrix Transcriptome Analysis Console (TAC) 4.02 software (Affymetrix, Santa Clara, CA) using Gene level–Core RMA and positive vs. negative AUC threshold = 0.7 [35]. The *p*-values were calculated with a one-way ANOVA test with eBayes correction. The differentially expressed gene lists with fold change of <-2 or >2 and gene level *p*-value of 0.05 (Anova method:ebayes) were used to generate hierarchical clusters and heatmaps and identify WIKI Pathways with the Affymetrix TAC software, as well as canonical pathways and functional networks using Ingenuity Pathways Analysis (IPA, Ingenuity^®^ Systems, www.ingenuity.com (accessed on 16 October 2020), QIAGEN Digital Insights, Germantown, MD, USA) [36]. IPA Network scores were calculated using the Fisher exact test, where the network score equals -log (Fisher exact test result): the higher the score, the lower the chance of obtaining the specific network when choosing molecules randomly in the Qiagen Knowledge database. Using machine learning techniques, IPA analysis was used to generate graphical summaries that provide a quick overview of the major biological themes in the IPA Core Analysis and illustrate how those concepts relate to one another by selecting and connecting a subset of the most significant entities predicted in the analysis, creating a coherent and comprehensible synopsis of the analysis.

### 2.4. Real Time PCR Validation

RNAs derived from SS patient and normal control cell lines were used for validation of microarray results. We used 10 ng of RNA from fibroblasts derived from the SS patient cells lines B1601, B1602, B1608 and two control cell lines B1610 and B16X, which were analyzed individually in duplicate. For the lymphoblast experiments, we used 25 ng of RNA per reaction from the SS patient cell lines MS4883 and MS4792 and the MS4881 control cell line, run in duplicate. At least three biological replicates (from three different passages) were used for each gene tested for both the fibroblast and lymphoblast experiments. Housekeeping genes were run each time differentially regulated genes were tested. Differentially-regulated genes were chosen based on microarray fold change and *p*-value for validation by qPCR, focusing on the top down-regulated and up-regulated genes, including some genes that had low fold-changes as well. Real time PCR reactions to validate differentially regulated genes were carried out with the QuantiTect SYBR Green RT-PCR Kit (QIAGEN, Germantown, MD, USA) in an iCycler (BioRad, Hercules, CA, USA) or a QuantStudio 6 Real Time PCR System (Applied Biosystems, Foster City, CA, USA). PCR primer sets, including the GAPDH housekeeping gene, were from the QuantiTect Primer Assay collection (QIAGEN, Germantown, MD, USA) and used as recommended by the manufacturer, with 10 ng of total RNA used per reaction for fibroblasts. The fold-changes of detected amplicons were calculated using the ∆∆Ct method [37,38] by comparing the average threshold cycles (Ct) of the one or three housekeeping genes to that of the tested genes. The mean, standard deviation and standard error of the mean (SEM) were calculated as described [38].

## 3. Results

### 3.1. Gene Expression Profiles of Fibroblast and Lymphoblastoid Cell Lines from SS Patients and Normal Controls

#### 3.1.1. Fibroblasts

Microarray analyses of samples from fibroblast cell lines derived from SS patients and normal Puerto Rican controls revealed that out of the top 983 differentially regulated genes (those with a fold change ≥ +/− 2.0 and *p*-value < 0.05), 479 were down-regulated and 509 were up-regulated (Supplementary Table S1). The genes downregulated more than 3-fold numbered 236, while 159 were upregulated more than 3-fold. Only 8 genes were upregulated more than 10-fold and 42 were downregulated 10-fold or greater. Although TWIST2 has been described mostly as a repressor, in fibroblast cell lines it appears to function as both a repressor as well as an activator, presumably causing activation of a large number of genes that include many that code for cytokines and extracellular matrix proteins. Hierarchical clustering resulted in two main clusters, one for each type of sample evaluated (Supplementary Figure S1).

#### 3.1.2. Lymphoblasts

In order to determine if the TWIST2 pathological mutation found in PR SS patients caused changes in steady state levels of specific mRNAs in cultured lymphoblastoid cells, we employed only two cell lines, one from a PR SS patient and another cell line from a PR control, which is a limitation of this study. We tested RNAs derived from three different passages from each cell line, which only reflect the biological variation of each type of cell line over time in culture [39,40]. Hence, the results obtained cannot reveal the biological variation in expression profiles of LCLs derived from different SS patients with the same TWIST2 mutation. Expression profiling of normal and SS lymphoblasts with Exon array GeneChips revealed that out of the top 1223 differentially regulated genes, 1155 were down-regulated and only 70 were up-regulated, which is a higher proportion of genes (~17 times) that apparently are activated by TWIST2 than those estimated to be activated in skin fibroblasts of SS patients (Supplementary Table S2). The genes downregulated more than 3-fold numbered 472 while only 15 were upregulated more than 3-fold. Only 1 gene was upregulated more than 10-fold and 2 were downregulated 10-fold or greater. In addition, TWIST2 is estimated to regulate a greater number of genes in lymphoblasts than in skin fibroblasts, which may indicate that TWIST2 has an important regulatory role in these cell types. Hierarchical clustering resulted in three main clusters, with two arrays hybridized with PR SS cDNAs in one cluster, a second cluster with two arrays reacted with PR control cDNAs and a third cluster with the remaining two arrays (one PR SS and one PR control) (Supplementary Figure S1), even though the heatmap shows that the 3 PR control arrays resemble each other more than the PR SS arrays do.

### 3.2. Quantitative RT-PCR Validation for Target Genes in SS Patient and Control Cell Lines

Table 1 summarizes validation of the 3′IVT microarray experiments performed with fibroblast cell lines by quantitative RT-PCR (qRT-PCR). All the 18 genes tested had the expected differential expression, although the magnitude of the fold-change was not the same. The SS cell lines showed lower expression level variability than the PR control cell lines. Nevertheless, the highest qRT-PCR fold changes were found in the top differentially regulated genes identified by microarray analyses (*CHRDL1, POSTN, PGF, IL8, CCL20* and *IL1RN)*. We have previously described TWIST2 regulation of *POSTN* in human cells [41]. Many cytokine genes were dysregulated, as expected from previous reports of TWIST2′s role in cytokine gene expression [10]. Extracellular matrix genes were also dysregulated in fibroblasts, which may explain the phenotype seen in SS patients and the Twist2 knockout mice [9,10]. 

Table 2 summarizes the validation of the Exon GeneChip experiments by quantitative RT-PCR (qRT-PCR) using LCLs derived from one Puerto Rican SS patient and one control. Of the 14 genes tested, 11 had the expected differential expression. One of the genes tested that did not validate (*SNX33*), showed a reduction (−6.3 ± 4.3-fold) in expression, rather than the expected ~2-fold increase in mRNA steady state levels. Two other genes (*LCE2D* and *REXO1L1P*) had high error rates both in the microarray expression levels and in the qRT-PCR results and did not validate either. The SMU1 gene had a lower than expected fold change (20% net decrease) and high error rates both in the microarray expression level and in the qRT-PCR result. Even though the RNAs evaluated came from different passages of the same cell lines, some genes exhibited higher variability in their steady state levels in exponentially growing cells. Compared to the results obtained from fibroblast cell lines, the lymphoblast expression profiles had greater variability. This is reflected also in the much lower *p*-values for the fibroblast dataset when compared to those of the lymphoblast results, as well as the much better validation results for the fibroblast expression by qRT-PCR. (Table 1 and Table 2 and Supplementary Tables S1 and S2). 

**Figure 2:**
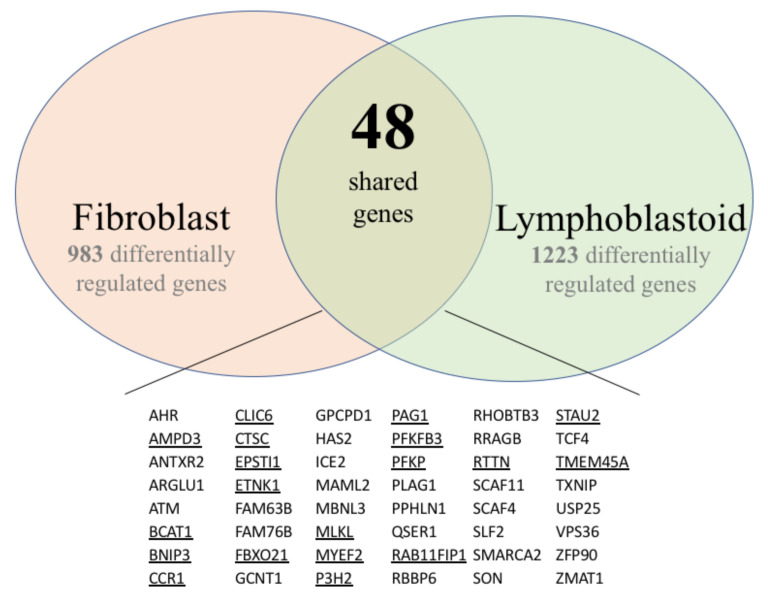
Differentially expressed genes detected in both skin fibroblast and lymphoblastoid cell lines. Genes that exhibited the same changes in gene expression in both cell types are underlined.

When comparing the differentially-regulated gene tables from fibroblasts and lymphoblasts, we found 48 genes shared by both gene lists (Figure 2), of which 17 had similar expression changes and 31 had opposite changes in gene expression. Those that exhibited the same changes in gene expression in both cell types are underlined in Figure 2. In Supplementary Table S3, genes that had the same changes in gene expression are underlined and in italics, while those that had opposite gene expression changes are indicated in bold letters. Further studies must follow up these results, yet we can infer a variation in function of the *TWIST2* gene due to the observed differences in gene regulation detected from microarrays analysis. Notably, the gene that codes for the chemokine receptor *CX3CR1* (*CCR1*) was downregulated in both cell types, which may imply a diminished wound healing function and immune response to specific viruses from fibroblasts and possibly B-lymphocytes, respectively. *CCR1* expression in fibroblasts is known to be associated with macrophages/monocytes recruitment at wound sites [42]. *CCR1* expression in B-lymphoblasts has been linked to Epstein-Barr virus (EBV) infections, specifically in viral clearance and attracting chemokine-activated cells [43]. Unfortunate, we cannot match clinical manifestation or possible outcomes of SS patients to genes shared in the two differentially-regulated gene lists due to lack of evaluation of SS adult patients. These results suggest that TWIST2 may employ different mechanisms to regulate a specific gene, most likely in combination with other trans factors that may be cell-type specific.

### 3.3. Cellular Pathways and Functional Networks Associated with SS

A large number of WikiPathways affected in SS patient fibroblast cell lines (143 with *p*-values < 0.05) included many differentially regulated genes (ranging from 35 to 1 gene, see Supplementary Table S4). Table 3 includes 10 pathways with ≥14 genes and *p*-values < 0.01, from 41 total pathways with at least 10 genes affected. The top five WikiPathways included the PodNet: protein-protein interactions in the podocyte, the Nuclear Receptors Meta-Pathway, the Adipogenesis, VEGFA-VEGFR2 Signaling, Endothelin and PI3K-Akt Signaling Pathways, among others. In contrast, many WikiPathways identified by TAC analysis in lymphoblasts (163 with *p*-values of <0.05, see Supplementary Table S5) included a smaller number of affected genes (ranging from 23 to 1 gene), where only 17 pathways contained >10 differentially regulated genes (included in Table 4) including 10 pathways with ≥15 genes and *p*-values < 0.05). The top five WikiPathways with the largest number of genes affected in lymphoblasts were EGF/EGFR Signaling, TGF-beta Signaling, JAK/STAT, Olfactory receptor activity and the Mesodermal Commitment Pathways.

**Figure 3:**
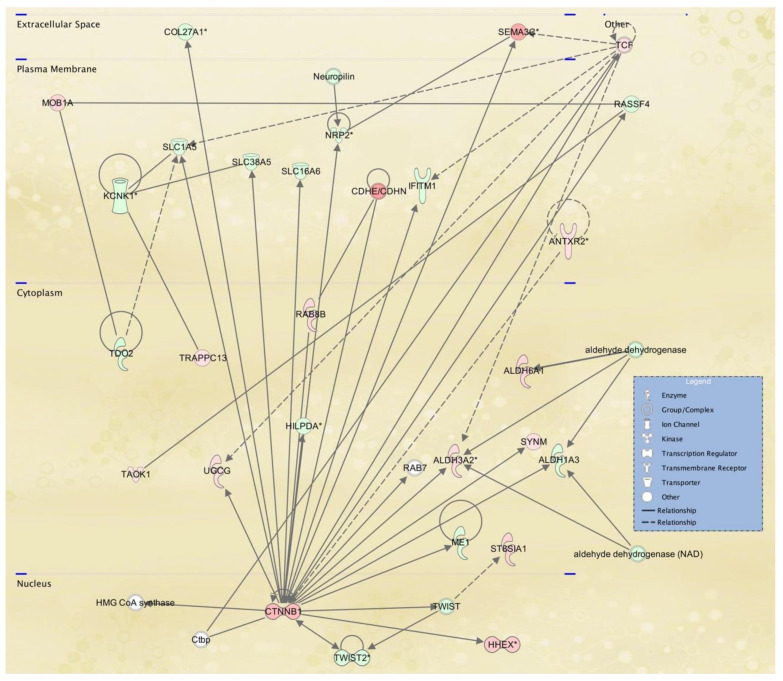
Lipid metabolism, molecular transport, small molecule biochemistry IPA network 2 affected in SS patient fibroblasts. IPA network 2 is centered in catenin beta. According to the Catenin beta Gene page in NCBI, this protein “anchors the actin cytoskeleton and may be responsible for transmitting the contact inhibition signal that causes cells to stop dividing once the epithelial sheet is complete” as well as being “part of a complex of proteins that constitute adherens junctions (AJs) necessary for the creation and maintenance of epithelial cell layers”. TWIST2 is down-regulated in this network and is a known regulator of epithelial to mesenchymal transitions (EMT). In addition, TWIST2′s role in lipid metabolism was evidenced in the phenotypes exhibited by the Twist2 knockout mice [11].

**Figure 4:**
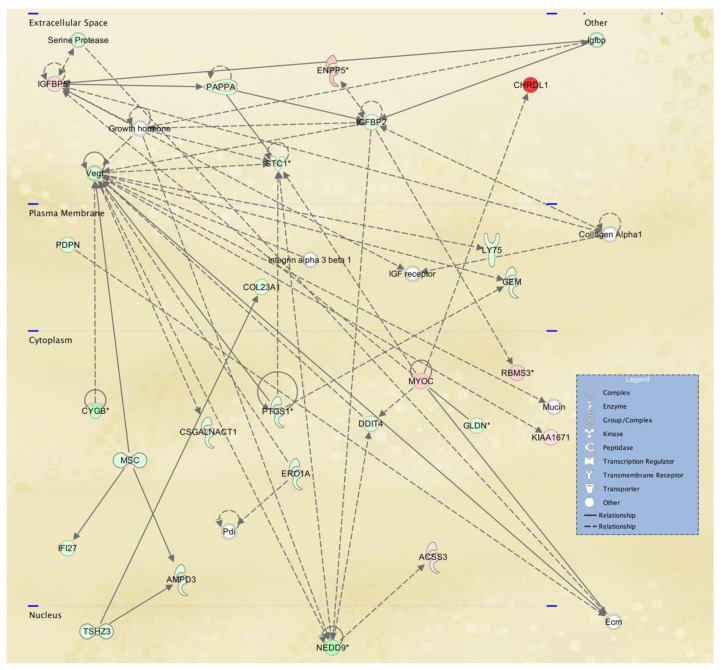
Cell signaling, free radical scavenging, small molecule biochemistry IPA network affected in SS fibroblasts. IPA network 5 includes chordin-like 1 (CHRDL1), a BMP4 antagonist that prevents its interaction with receptors as well as vascular endothelial growth factor (VEGF), which is down-regulated, and is the principal angiogenic growth factor modulating neovascularization.

**Figure 5:**
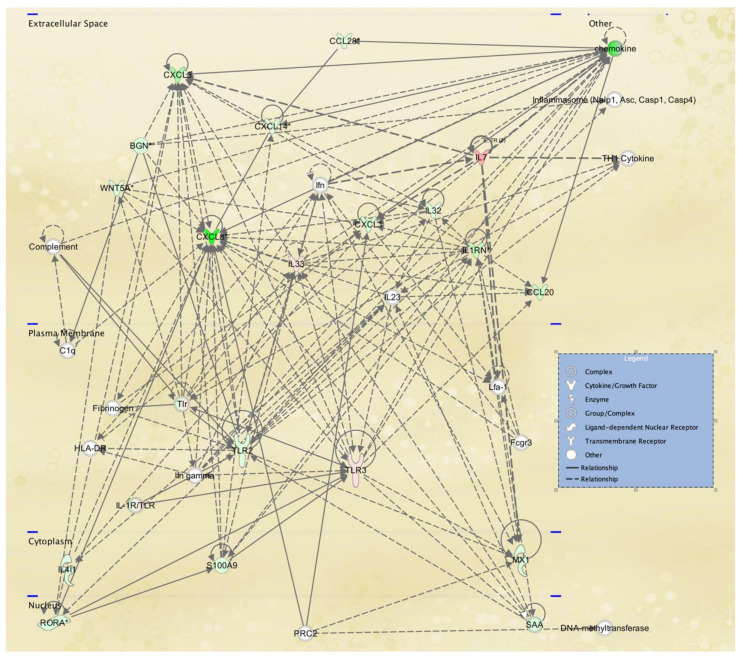
Cellular movement, hematological system development and function, tissue development IPA network affected in SS fibroblasts. IPA functional network 21 shows that mRNAs that code for multiple cytokine and chemokines, such as IL-8, IL-1RN and CXCL3, are down-regulated in fibroblasts derived from SS patients. Twist2′s role in regulating cytokine gene expression was highlighted in the Twist2 knockout phenotypic description [11].

To gain further insight into the potential TWIST2 target genes, the identified protein products for fibroblasts and lymphoblastoid cells were mapped to canonical pathways (Supplementary Tables S6 and S7, and top five pathways in Table 5 and Table 6, respectively) and functional networks (Supplementary Tables S8 and S9 and top five networks in Table 7 and Table 8, respectively) generated using IPA [36]. Red and green symbols represent upregulated and downregulated molecules, respectively, in the network images included below in Figure 3, Figure 4 and Figure 5. The straight lines in the IPA network figures represent direct relationships and the dotted lines represent indirect relationships. 

Twenty-five networks were identified in fibroblast cell lines from SS patients and ranked by the IPA score in the *p*-value calculation, which ranged from 17 to 44. The scores take into account the number of focus proteins and the size of the network to approximate the relevance of the network to the original list of focus proteins. The highest-scoring networks revealed a significant link with cancer, nervous system development and function and neurological disease. Furthermore, lipid metabolism (Figure 3), molecular transport, small molecule biochemistry (Figure 4) as well as cancer, organismal injury and abnormalities, reproductive system disease might also be influenced in the next two highest scoring networks, respectively. Figure 5 shows the cellular movement, hematological system development and function and tissue development IPA network, which includes many cytokine genes, as expected, since Twist2 has been proposed to regulate cytokine gene expression [10]. The components of all the IPA functional networks are included in Supplementary Table S8. 

**Figure 6:**
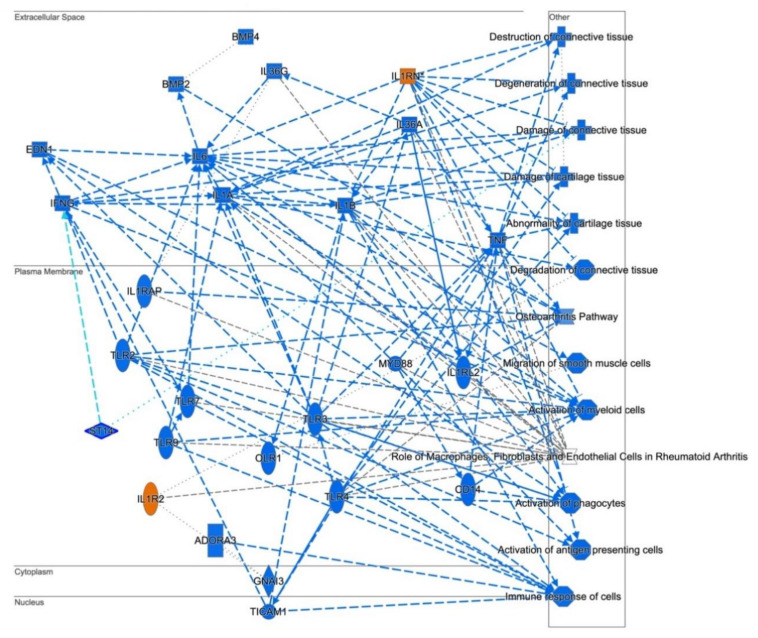
Graphical summary of human skin fibroblast profiling of SS patients. The Ingenuity Pathways Analysis showed that deficiency of TWIST2 activity impacts connective tissue, importantly in its destruction, degeneration and damage repair. Includes all entities with *p* < 0.05 and z score ≥ 2 for diseases, functions and upstream regulators. Orange entities repre-sent activated nodes with z score ≥ 2, while blue entities represent inhibited nodes with z score ≤ −2. Ellipses represent transmembrane receptors, diamonds = enzymes, squares = cytokines or growth factors, rectangles = G-protein coupled receptors, octagons = function, crosses = disease, squeezed rectangle = canonical pathway and circles = other.

The graphical summary in Figure 6 showed that deficiency of TWIST2 activity may impact connective tissue destruction, degeneration and damage repair. TWIST2 appears to have an important role in activation of myeloid cells, phagocytes and antigen-presenting cells, as identified by IPA.

In contrast, IPA of lymphoblastoid cell expression profiles identified twenty-five networks with IPA scores that ranged from 26 to 45. The five highest-scoring networks (Table 8) revealed a significant link with endocrine system disorders, hereditary disorder, organismal injury and abnormalities, cell cycle, cellular assembly and organization, DNA replication, recombination, and repair, cardiovascular disease, developmental disorder, digestive system development and function, hereditary disorder, metabolic disease, organismal injury and abnormalities and auditory disease, cellular development, cellular growth and proliferation. The components of all the IPA functional networks are shown in Supplementary Table S9. 

**Figure 7:**
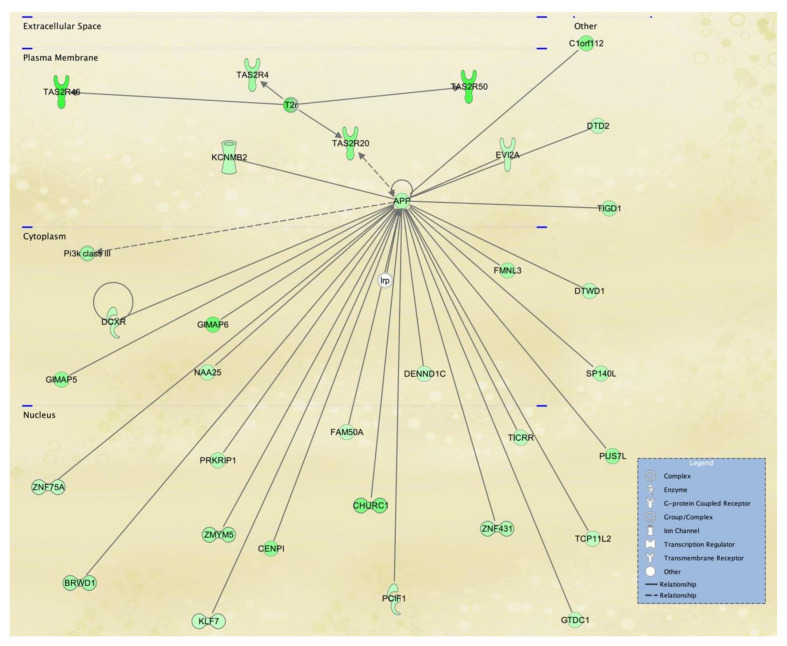
Hereditary disorder, metabolic disease, organismal injury and abnormalities network affected in lymphoblastoid cells derived from an SS patient. IPA network 4 is centered in the amyloid beta precursor protein (APP), which is also found in the WikiPathways for TGFβ signaling and the IPA Canonical Pathway for Reelin signaling in neurons. Several olfactory receptor genes are down-regulated and practically all of the genes in this causal network.

**Figure 8:**
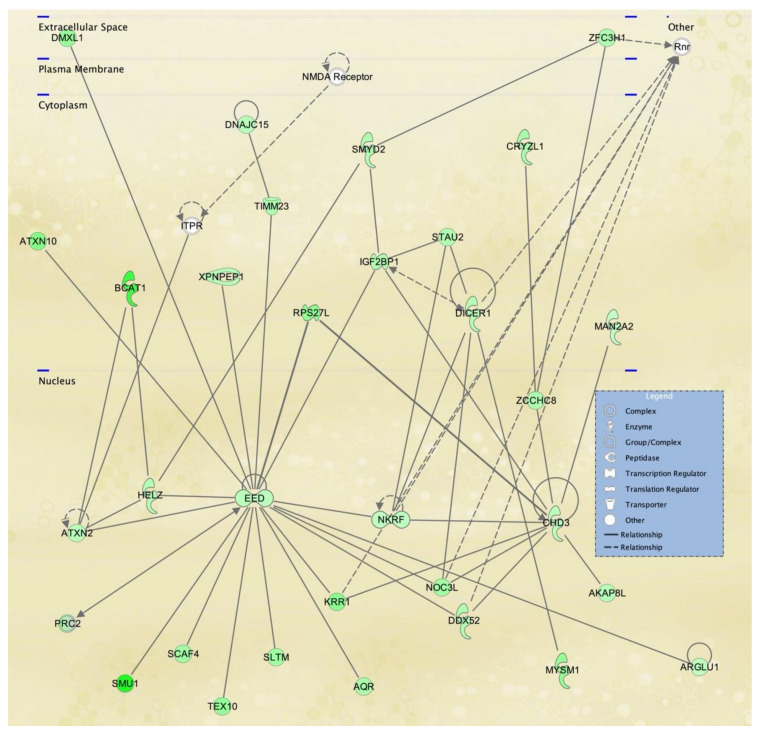
Cancer, organismal injury and abnormalities, RNA post-transcriptional modification network affected in lymphoblastoid cells derived from an SS patient. IPA Network 9 is centered on the EED gene, which encodes a member of the Polycomb-group (PcG) family, involved in maintaining the transcriptional repressive state of genes over successive cell generations through histone deacetylation. This gene network is essentially repressed in the LCL deficient in TWIST2 activity used in this study.

**Figure 9:**
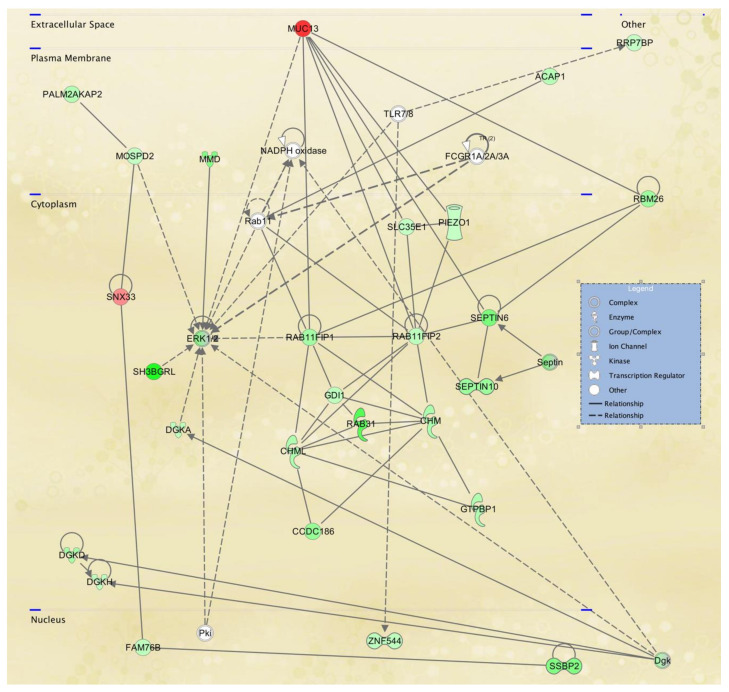
Hereditary disorder, organismal injury and abnormalities, post-translational modification network affected in lymphoblastoid cells derived from an SS patient. IPA network 17 includes two of the genes that were validated by qRT-PCR (MUC13 and RAB31).

To visualize selected IPA networks, we present the interconnection of differentiated genes by subcellular component from lymphoblasts in Figure 7, Figure 8 and Figure 9, corresponding to IPA networks 4, 9 and 17. Figure 7, Figure 8 and Figure 9 show dysregulation of genes in lymphoblasts; interestingly, more nuclear components and transcription regulators are dysregulated in these networks.

IPA network 4 (Figure 7) shows many down-regulated molecules in the lymphoblast cell line from an SS patient, which function at the nucleus and is centered on the Amyloid precursor protein (APP). APP is a ubiquitously expressed transmembrane protein with an unclear function other than pathophysiological reaction to aggregation of plaques upon cleavage, however in normal conditions APP has been suggested to mediate cell-cell interactions and immunological responses [44]. While one study provided evidence of uninterrupted thymus T lymphocyte development in APP-deficient mice, another discussed the mediation of the macrophage phenotype by APP in high-fat-diet mice [45]. Nevertheless, it is unknown whether down-regulation of APP in the SS patient cell line may reflect changes in APP expression in other tissues. Additionally, we do not understand if B cell maturation and antibody production may be affected in SS patients, since there is a paucity of clinical evaluation of SS patients in adulthood in organs or tissues besides the eye and skin.

IPA network 9 (Figure 8), like network 4, includes several nuclear molecules. Network 9 contains several down-regulated RNA processing (ATXN2, HELZ, SCAF4, TEX10, SLTM, AQR, KRR1, DDX52 and DICER1) and DNA replication (SMU1, NOC3L, CHD3, AKAP8L, MYSM1) molecules, but also Embryonic ectodermal development (EED) protein and Chromodomain Helicase DNA binding protein 3 (CHD3) emerge as key centric molecules. EED and CHD3 mediate histone modifications as part of the Polycomb repressive complex 2 (PRC2) and NuRD complex, respectively [46,47]. Both complexes are essential for Epithelial-mesenchymal transition (EMT) maintenance and cell differentiation corresponding to the TWIST2 known role in embryonic stem cells. Studies reveal that any potential inactivation of EED-via PRC2 and CHD3-via NuRD complexes can prevent lineage commitment and terminal differentiation. In particular, the PRC2 complex is partially responsible for pro-B to pre-B cells maturation by VJ558 gene rearrangement. On the other hand, there is evidence of TWIST recruiting the NuRD complex to mediate CDH1 gene promoter transcription repression to promote EMT in cancer cells [48]. Once again, it is unclear the consequences of the down regulation of EED and CHD3 in the SS patient LCL we used, and the impact it may have in B cell maturation [49]. These results may suggest that SS patients may be prone to immune-related diseases by partial or incomplete B cell maturation as a consequence of dysregulation of key genes. However, this hypothesis needs be examined experimentally and should include additional cell lines derived from SS patients and controls, in order to determine if what our results suggest is specific for the single PR SS and PR control cell lines we compared, or they may apply to SS patient vs. control LCLs in general. 

**Figure 10:**
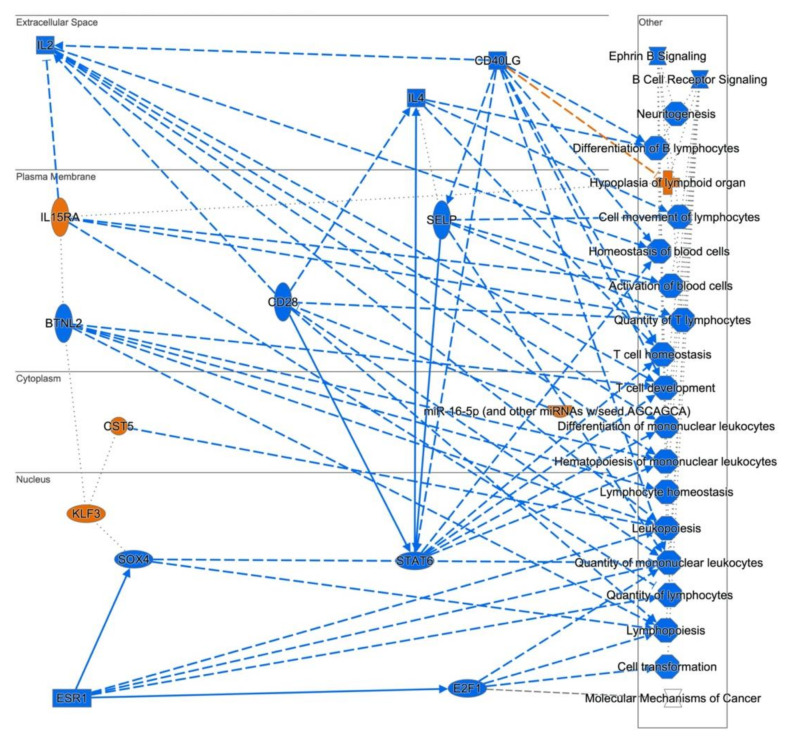
Graphical summary of expression profiling of human lymphoblastoid cells derived from an SS patient. Many of the processes affected in these types of cells are essential for lymphocyte differentiation and formation, homeostasis, cell survival and B Cell receptor signaling. Includes all entities with *p* < 0.05 and z score ≥ 2 for diseases, functions and upstream regulators. Orange entities repre-sent activated nodes with z score ≥ 2, while blue entities represent inhibited nodes with z score ≤ −2. Vertical ellipses represent transmembrane receptors, horizontal ellipses = transcription regulators, rectangular bar = ligand-dependent nuclear receptor, squares = cytokines or growth factors, octagons = function, crosses = disease, squeezed rectangle = ca-nonical pathway and circles = other.

The graphical summary in Figure 10 suggests that many of the processes affected in lymphoblastoid cells are lymphocyte differentiation and formation, homeostasis, cell survival and B Cell receptor signaling. 

## 4. Discussion

Microarray analysis of cell lines typically collected in human genetic studies can provide valuable information on disease mechanisms. Since TWIST2 is a transcription factor, we expected that expression profiling would unearth target gene information. In this study, we generated differentially regulated gene lists for two types of cell lines (fibroblast and lymphoblastoid) that were used to identify putative affected cellular pathways (Wiki Pathways and IPA canonical pathways) and causal networks generated from individual relationships curated from the literature, which help to interpret the expression profiles [32]. Since we used cell lines derived from a small number of PR SS patients and controls, the results of our study are limited in scope, especially with the expression profiles obtained from lymphoblasts derived from a single PR SS patient and a single PR control. In addition, since LCLs arise after Epstein-Barr viral infection, virus-induced cellular changes due to the transformation process may impact the resulting expression profiles. Single cell RNA sequencing has revealed that LCLs may exhibit substantial phenotypic diversity [50]. Hence, the variability we observed in the microarray results from three different passages of PR SS and PR control LCLs is not unexpected. 

The top WikiPathway in our fibroblast cells dataset is the VEGFA-VEGFR2 signaling pathway in endothelial cells [51,52], where 35 genes were differentially regulated (See Supplementary Figure S1). Vascular endothelial growth factor (VEGF) is the principal angiogenic growth factor modulating neovascularization [53]. SS fibroblasts exhibited down-regulation of VEGFA, affecting other WikiPathways (the endothelin, PodNet: protein-protein interactions in the podocyte pathways as well as the PI3K-Akt signaling/focal adhesion-PI3K-Akt-mTOR-signaling and the sudden infant death syndrome (SIDS) Susceptibility pathways (Table 4). Podocytes, which are visceral epithelial cells, comprise the main filtration barrier in the glomerulus and express VEGF [54]. 

Two top IPA networks in the fibroblast dataset include cancer as a process, which may be associated with the TWIST proteins role in EMT [55], an important process in tumor metastasis. The second top network generated by IPA involves lipid metabolism and molecular transport as an affected function, which deserves further study given the absence of brown fat and lipid droplets in the Twist2 knockout mice [10]. Similar findings have not been reported in SS patients, which highlights the need to further investigate the manifestations patients may have in organs like the liver, spleen, and skeletal muscle, as well as in blood. Other top networks may be involved in undescribed manifestations of the syndrome. IPA Network 21 (Figure 5) contains many cytokine and chemokine genes in a network centered on IL-8, which is the top down-regulated gene, corroborating TWIST2′s role in regulation of the expression of many cytokines [10]. 

TAC analysis of the human lymphoblast microarray dataset identified cytokine and inflammatory response genes as severely affected, which was also expected [10], suggesting that the inflammatory response genes may be down-regulated in SS patients, which deserves further study. One of the top disease functions affected is cell migration and connective tissue degeneration, destruction and repair, which is not surprising based on the SS facial manifestations, that result from improper migration of cells during development. 

The amyloid precursor protein (APP) [44,45], down regulated in the SS LCL is included in the TGFβ signaling WikiPathway, the Reelin signaling in neurons IPA canonical pathway, and IPA network 4 for hereditary disorder, metabolic disease, organismal injury and abnormalities. APP is described in its Gene page at NCBI (https://www.ncbi.nlm.nih.gov/gene/351 (accessed on 10 December 2020)) as follows: “encodes a cell surface receptor and transmembrane precursor protein that is cleaved by secretases to form a number of peptides.” As indicated in the NCBI APP gene page, “some of the peptide products of APP cleavage are secreted and can bind to the acetyltransferase complex APBB1/TIP60 to promote transcriptional activation, while others form the protein basis of the amyloid plaques found in the brains of patients with Alzheimer disease. In addition, two of the APP cleavage peptides are antimicrobial peptides, having been shown to have bacteriocidal and antifungal activities.” IPA Network 4 links APP with down regulation of many nuclear proteins, the majority being transcription factors, down regulated themselves as a consequence of APP’s expression level.

We expected that TWIST2 would regulate different sets of genes in different cell types and the results obtained in the two different types of cell lines employed provide evidence in favor of this hypothesis, even with the limitations mentioned above. It remains to be explained how TWIST2 may influence the activity of other transcription factors, co-repressors or co-activators, which may have important roles during development and beyond. There are several known transcription factors that interact with TWIST2, some by using the C-terminal TWIST box domain [10,20,21,22,23], but other regions besides the HLH domain, such as the N-terminus, have the capacity to interact as well [30]. Being a small bHLH protein certainly does not limit TWIST2 from using several mechanisms to interact with other trans factors. The severe manifestations in multiple facial structures point to the importance of TWIST2 in skin, eye and eyelid and limb formation. This study, which has limitations due to the small number of patients used to generate the cell lines used for expression profiling, provides information on potential target genes that may be studied further to begin to understand TWIST2′s role in cell differentiation and development.

## 5. Conclusions

In summary, these studies have provided important information on putative TWIST2 target genes, cellular pathways and causal networks that may provide insight for future studies of this important transcription factor. TWIST2 appears to have a variable role, depending on the cell type and most likely, its interacting partner in dimerization in the regulation of specific target genes.

## Figures and Tables

**Table 1:** Selected genes showing differential hybridization results in Setleis versus control human skin fibroblasts.

Gene Symbol	SS Ave ± Std Dev (log2)	WT Ave_ Std Dev (log2)	MA Fold Change	Mod t/ *p*-Value	Mean qRT-PCR Fold Change ± SEM	Full Name, Function
CXCL8/IL8	5.60 ± 0.21	12.94 ± 0.80	−162	−35.8/ 8.7 × 10^−7^	−251.9 ± 65.2	Interleukin 8, CXC chemokine
COL15A1	14.24 ± 0.89	9.10 ± 0.9	−29	−13.12/ 0.0002	−4.91 ± 0.4	Collagen type XV, alpha
POSTN	4.63 ± 1.38	9.35 ± 0.86	−28.3	−8.8/ 0.002	−144.0 ± 31.6	Periostin. Secreted extracellular matrix protein
CXCL3	4.84 ± 0.23	9.32 ± 1.36	−22.3	−9.8/ 0.0003	−14.2 ± 2.1	MIP2−Beta. CXC chemokine
IL1RN	4.79 ± 0.67	8.68 ± 0.72	−14.8	−16.8/ 0.0003	−48.7 ± 9.7	Interleukin 1 receptor antagonist.
CSTA	4.68 ± 0.57	8.40 ± 0.75	−13	−12.7/ 0.0001	−26.6 ± 5.4	Cystatin A. Cysteine protease inhibitor
CCL20	3.98 ± 0.33	7.54 ± 1.42	−11.8	−9.8/ 0.0005	−239.5 ± 38.5	MIP3α. Chemokine (C−C motif) ligand 20
PGF	5.21 ± 0.66	8.60 ± 0.96	−10.4	−10.1/ 0.0004	−56.4 ± 14.2	Placental growth factor
RUNX3	4.59 ± 0.26	7.50 ± 0.88	−7.5	−7.45/ 0.0014	−23.2 ± 9.8	runt domain transcription factor
TRIB3	8.19 ± 0.08	9.28 ± 0.10	−2.1	−23.2/ 2.3 × 10^−5^	−4.6 ± 0.8	Tribbles pseudokinase 3
LAMA4	11.36 ± 0.45	10.10 ± 0.12	+2.4	+19.9/ 1.6 × 10^−5^	2.9 ± 0.4	Laminin alpha 4
RALGPS2	7.38 ± 0.42	5.56 + 0.13	+3.5	+15.9/ 8.8 × 10^−5^	+1.9 ± 0.3	Ral GEF
MEST	9.93 ± 0.52	7.22 ± 0.95	+6.5	+9.7/ 0.0005	+15.5 ± 5.6	[α]/[β] hydrolase fold family member
IL7	7.72 ± 0.20	5.00 ± 0.70	+6.7	+35.8/ 0.0002	+3.2 ± 0;.7	Interleukin 7
PRLR	5.48 ± 0.77	4.20 ± 0.43	+7.6	+10.9/ 0.0076	+54.1 ± 5.6	Prolactin receptor
MYRIP	6.78 ± 0.48	3.64 ± 0.27	+8.8	+15.1/ 1.14 × 10^−5^	+26.6 ± 8.8	Rab27A-binding protein and actin interacting protein
CHRDL1	11.19 ± 0.82	5.12 ± 0.76	+67	+15.3/ 1.3 × 10^−5^	+74.3 ± 11.9	Chordin like 1, bone morphogenetic protein 4 antagonist

SS ave ± std dev (log2) = Log2 of the average expression levels ± standard deviations for samples from PR SS fibroblast cell lines; WT ave ± std dev = Log2 of the average expression levels ± standard deviations for samples from PR Control fibroblast cell lines; MA fold change = average fold difference (in linear space) of microarray expression levels between Puerto Rican SS patients vs. normal controls; mod t = moderated t statistic. The qRT-PCR fold changes represent the mean of at least three analyses performed in duplicate, normalized against the expression of three control genes (RPS11, β-actin and α-tubulin) using the ΔΔCt method [37,38].

**Table 2:** Selected genes showing differential hybridization results in Setleis versus control human lymphoblasts.

Gene Symbol	SS Ave ± Std Dev (log2)	WT Ave_ Std Dev (log2)	MA Fold Change	*p*-Value	Median qRT-PCR Fold Change *	Protein Product Name, Function
PRKCH	5.43 ± 0.58	8.63 ± 0.72	−9.2	0.0006	−14.1 ± 7.9	Protein Kinase C, eta
SMU1	4.83 ± 2.15	7.80 ± 1.32	−7.8	0.0399	−2.2 ± 1.0	SMU1 DNA Replication Regulator
BCAT1	5.94 ± 1.11	8.69 ± 1.07	−6.8	6.83 × 10^−5^	−2.7 ± 0.6	Cytosolic branched-chain amino acid transaminase
LPXN	6.89 ± 1.6	9.56 + 0.14	−6.4	0.0105	−4.1 ± 0.6	Leupaxin, Transcriptional coactivator
TAS2R50	4.09 ± 0.49	6.76 ± 0.92	−6.4	0.0057	−4.2 ± 2.9	Taste 2 Receptor Member 50
RAB31	6.24 ± 0.69	8.80 ± 0.46	−5.9	0.0011	−5.8 ± 3.3	Ras-Related Protein Rab-31
RHOBTB3	4.51 ± 0.71	6.95 ± 0.81	−5.4	0.0075	−2.3 ± 0.8	Rho Related BTB Domain Containing 3-Rab9-regulated ATPase
NPIPB4	6.99 ± 1.04	9.33 ± 0.51	−5.1	0.0055	−7.7 ± 3.8	Nuclear Pore Complex Interacting Protein Family Member B4
GIMAP6	5.64 ± 0.67	7.98 ± 0.97	−5.0	0.0137	−40.1 ± 27.7	GTPases of immunity-associated proteins (GIMAP) family member
CCR1	3.65 ± 0.50	5.72 ± 1.21	−4.2	0.0286	−7.7 ± 3.8	chemokine (C-C motif) receptor 1
SNX33	5.64 ± 0.37	4.55 ± 0.31	2.1	0.0219	−6.3 ± 4.3	Sorting nexin 33
MUC13	9.37 ± 1.27	6.37 ± 0.47	8.0	0.0156	+339.5 ± 307.4	Mucin 13,
LCE2D	5.75 ± 1.11	2.5 ± 1.39	9.5	0.0086	12.8 ± 12.6	late cornified envelope 2D
REXO1L1P	7.33 ± 2.68	3.62 ± 0.52	13.1	0.0417	6.45 ± 6.3	RNA exonuclease 1 homolog-like 1 pseudogene

SS ave ± std dev (log2) = Log2 of the average expression levels ± standard deviations for samples from a PR SS LCL; WT ave ± std dev = Log2 of the average expression levels ± standard deviations for samples from a PR Control LCL. MA Fold change = average fold difference (in linear space) of microarray expression levels between different passages of a Puerto Rican SS patient cell line vs. a normal control cell line. *The quantitative RT-PCR (qRT-PCR) fold changes represent the mean of at least three biological replicates performed in duplicate, normalized against the expression of the GAPDH housekeeping gene using the ΔΔCt method [37,38].

**Table 3:** Top ten WikiPathways affected in fibroblasts derived from SS patients. See Supplementary Figures for depictions of the top five WikiPathways.

Pathway	#Total	Genes in Pathway *	Significance	*p*-Value
VEGFA-VEGFR2 Signaling Pathway	35	**PRKD2,HSPA1A,SMARCA2,NCK1,QGAP1,** **PLAUPIK3R1,TXNIP,GSK3B,CTNNB1,** **FBXW11,EPS15,TNXB,ADAM10,S1PR1,ADRB2PDE4DIP**,*VEGFA,SPHK1,PRKCD,NFKBIA,* *NRP2,PTGS2,CXCL8,ICAM1,PLAUR,SOD2,* *IGFBP7,PGF,SLC39A14,CSRP2,PGK1,SSR3,* *CGNL1,P4HA2*	6.24	0.000001
PodNet: protein-protein interactions in the podocyte	31	**MYOC,SMAD3,AGTR1,IQGAP1,UTRN,** **ADAM10,SPTAN1,KCNMA1,PLCE1,NCK1,** **SP1,SPTBN1,PIK3R1,CTNNB1,MET,ACTR2,** *CTTN,KRT7IGFBP7PTGS2,VEGFA,DDR1,NOTCH3,ANGPTL4,PLAUR,IGFBP2,CDKN1C,PDPN,* *COL18A1,PALLD,SYNPO*	8.02	0
Nuclear Receptors Meta-Pathway	30	**NR3C1,SDPR,PPARGC1A,NCOA2,SP1,** **HSPA1A,ABHD2,PLK2,AHR,NCOA3,SLC6A15** *SLC7A11,CES1,SRGN,TSC22D3,SLC2A1,* *TNFAIP3,PTGS2,CCL20,ANGPTL4,SLC7A5,* *GSTT2,SRXN1,AMIGO2GCLM,CDKN1C,NRG1,ME1,SLC6A8,SLC39A14*	6.88	0
Endothelin Pathway	24	**CDH2,VCAN,IGFBP5,CTNNB1,ACTR2,GULP1CASP8,GSK3B,TCF4,** *RGS3,CXCL8,PLOD2,* *PTGS2,COL5A1,MMP3,COMP,MMP1,ADAM12,EDNRA,NFKBIA, IL6,CXCL1,MCAM, VEGFA*	3.73	0.000186
PI3K-Akt Signaling Pathway	23	**PIK3R1,ITGA7,COL6A6,LAMA4,PRLR,TNXB,** **PDGFD,MET,GNG12,GSK3B,IL7,NTRK2** *GYS1,COMP,DDIT4,ITGA8,FGF1,VEGFA,PGF,* *EIF4EBP1,IL6,TLR2,TLR4*	3.13	0.000737
Focal Adhesion-PI3K-Akt-mTOR-signaling pathway	22	**PIK3R1,ITGA7,MET,LAMA4,PRLR,TNXB,** **PDGFD,GNG12,GSK3B,PPARGC1A,** *COMP,* *COL5A1,DDIT4,ITGA8,FGF1,VEGFA,PGF,* *SLC2A1,GYS1,PFKFB3,PFKFB4,EIF4EBP1*	3.51	0.00031
Adipogenesis	19	**NR3C1,RBL1,SP1,PPARGC1A,NCOA2,MBNL1,CTNNB1,SMAD3,PRLR,AHR,LMNA,** *DDIT3,* *LIF,CYP26B1,MIF,LEP,IL6,TRIB3,RORA*	7.53	0
Circadian rhythm related genes	19	**PRKG2, NCOA2,DDX5,KCNMA1,OGT,** **PPARGC1A,PRKDC,FBXW11,GSK3B,NLGN1,** **NR1D2,DHX9,AHR,** *LEP,RORA,IL6,OPN3,ID2,* *NAMPT*	4.58	0.000026
IL-18 signaling pathway	17	**CASP8,CTNNB1,GSK3B,SLC4A7,KLF2,PIK3R1** **SP1,** *MMP3,FOXN3,NFKBIA,IL6,CCL20,CXCL8,* *MMP1,SPON1,PTGS2,PRKCD*	2.04	0.009034
Sudden Infant Death Syndrome (SIDS) Susceptibility Pathways	14	**NR3C1,SP1,PPARGC1A,THRB,CTNNB1,NTRK2,SPTBN1,** *RUNX3,RORA,IL6,IL1RN,VEGFA,* *TAC1,POU2F2*	3.26	0.000553

* Gene names in bold = upregulated; italics = Downregulated.

**Table 4:** WikiPathways affected in lymphoblastoid cells derived from an SS patient. See Supplementary Figures for depictions of the top five WikiPathways.

Pathway	#Total	Genes in Pathway *	Significance	*p*-Value
EGF/EGFR Signaling	23	*MAPK1,MAPK8,EPS15L1,RASA1,VAV2,SOS2,MAPK3,* *JAK1,CDC42,ATXN2,RPS6KB1,ASAP1,SYNJ1,MAP4K1,USP6NL,ABI1,ROCK1,USP8,PTEN,STAM,STAT5B,* *CBL,PTK2*	2.37	0.004236
TGF-beta Signaling	22	**SPTBN1,** *MAPK1,MAPK8,WWP1,NUP214,TGFBR1,* *RBL2,MAPK3,CDC42,SMAD2,* *STAMBPL1,BTRC,SMURF2,E2F4,ITGB1,MAP4K1,APPMAP2K6,PIAS1,ROCK1,ZEB1,PTK2*	3.07	0.000854
JAK/STAT	19	*PTPN6,MAPK3,FLNA,STAT5B,VAV2,CBL,CHUK,* *RPS6KB1,PTK2,MAPK1,REL,BAX,ITGB1,IGF1,JAK1,* *CDC42,ROCK1,CFL2,MAPK8*	2.84	0.001456
Olfactory receptor activity	19	**OR6C76,OR6C74,OR9G1,OR1M1,OR52L1,OR2L8,** **OR7C2OR2B3, OR1L1,OR4N5,OR10H2,OR6C3, OR8K5, OR4N2,OR52N2,OR52E2,OR10A7,** *OR52H1,OR4B1*	1.38	0.041643
Mesodermal Commitment	19	**LEF1,** *PIAS1,NLK,WDFY2,BMPR2,CCDC6,ELP4,* *C9orf72,TRIM5,FGFR1,LATS1,KDM6A,MBTD1TCF4,* *BMPR1A,TRERF1,TWSG1,SMAD2,AEBP2*	1.34	0.045833
Genes involved in male infertility	18	**UBD,** *ABLIM1,AHR,ARNTL,ATM,CLOCK,EPSTI1,FASLIG4,MLH3PEMT,RGS9,SHMT1,TEX15,USP8,CCNT1,CCNK,CDK9*	1.39	0.040353
Endoderm Differentiation	18	**LEF1,** *RTF1,RFX7,BPTF,MBTD1,SP4,APP,AEBP2,BMPR1A,SMAD2,PIAS1,NLK,WDFY2,ELP4,TRIM5,TCF4,* *EMSY,TRERF1*	1.39	0.040353
B Cell Receptor Signaling	16	*CHUK,REL,MAP2K6,VAV2,MALT1,PTPN6,MAPK3,* *MAPK8,RAPGEF1,MAX,CDC42,CBL,BLK,MAPK1,* *CD22,MAP4K1*	2.38	0.004197
T-Cell antigen Receptor(TCR) Signaling	15	*MAP4K1,MAPK8,LCP2,WAS,REL,CHUK,FYB,CDC42,GRAP2,MAPK3,MAPK1,MALT1,CBL,SKAP1,FAS*	2.31	0.004854
Integrin-mediated Cell Adhesion	15	**SEPP1,** *CDC42,ARHGEF7,VAV2,ROCK1,GIT2,* *PTK2,CAPN3,PAK6,MAP2K6,RAPGEF1,* *MAPK1,ITGAX,ITGB1,VCL*	1.89	0.012959

* Gene names in bold = upregulated; italics = Downregulated.

**Table 5:** Top five canonical pathways identified by IPA in skin fibroblasts and the differentially-regulated genes associated with these pathways.

Ingenuity Canonical Pathways	*p*-Value	Genes in Pathway *
Role of IL-17A in Psoriasis	7.94 × 10^−10^	*CCL20,CXCL1,CXCL3,CXCL5,CXCL6,CXCL8,S100A7,S100A9*
Osteoarthritis Pathway	7.24 × 10^−9^	**CASP8,** *CCN4* **,CTNNB1,** *CXCL8* **,** *DDIT4* **,DDR2,GDF5,** *LEP,MMP1,MMP3,NAMPT,PGF,* **PPARGC1A** **,** *PTCH1,PTGS2,S100A9* **,SMAD3,** *SOX9* **,SP1,** *SPHK1* **,TCF4,TCF7L2,** *TLR2,TLR4,VEGFA*
Role of Macrophages, Fibroblasts and Endothelial Cells in Rheumatoid Arthritis	3.16 × 10^−8^	**CAMK2D,CTNNB1** **,** *CXCL8* **,GSK3B,** *ICAM1,IL1RN* **,** *IL32* **,IL33,** *IL36B,IL6* **,** **IL7** **,** *MIF,MMP1,MMP3,NFKBIA* **,PDGFD,** *PGF* **,PIK3R1,** *PLCB4* **,PLCE1,** *PPP3CC,PRKCD,SFRP2* **,TCF4,TCF7L2,** *TLR2* **,TLR3,** *TLR4,VEGFA,* *WNT5A*
Hepatic Fibrosis/Hepatic Stellate Cell Activation	5.89 × 10^−8^	**AGTR1** **,** *COL15A1,COL18A1,COL23A1,COL27A1,COL5A1* **,COL6A6,** *CXCL3,CXCL8,EDNRA,FGF1,ICAM1* **,IGFBP5,** *IL6,LEP* **,MET,** *MMP1* **,** **PDGFD** **,** *PGF* **,SMAD3,** *TLR4,VEGFA*
Glucocorticoid Receptor Signaling	1.62 × 10^−7^	**ADRB2** **,** *CDKN1C,CXCL3,CXCL8* **,HSPA1A/HSPA1B,** *ICAM1,IL1RN,IL6,KRT14,KRT15,KRT19,KRT34,KRT6A,KRT6B,KRT7,MAPK13,MMP1* **,** **NCOA2,NCOA3,** *NFKBIA* **,NR3C1,PIK3R1,PLAU,** *POU2F2,PPP3CC,* *PTGS2* **,SMAD3,SMARCA2,TAF9B,** *TSC22D3*

* Gene names in bold = upregulated; italics = Downregulated.

**Table 6:** Top five canonical pathways identified by IPA in lymphoblastoid cells and the differentially-regulated genes associated with these pathways.

Ingenuity Canonical Pathways	*p*-Value	Genes in Pathway*
FAK Signaling	2.95 × 10^−5^	*ARHGEF6,ARHGEF7,ASAP1,CAPN3,GIT2,ITGB1,MAPK1,MAPK3,* *PIK3C2A,PIK3C3,PIK3R5,PTEN,PTK2,SOS2,VCL,WAS*
Ephrin B Signaling	7.59 × 10^−5^	*ABI1,CAP1,CBL,CDC42,CFL2,GNA13,GNG2,GNG7,MAPK1,MAPK3,PTK2,* *ROCK1,VAV2*
Molecular Mechanisms of Cancer	7.94 × 10^−5^	*ADCY3,ARHGEF6,ARHGEF7,ATM,BAX,BMPR1A,BMPR2,CBL,CDC42* *,CDK16,CDK9,CTNND1,E2F4,E2F7,E2F8,GNA13,ITGB1,JAK1,LEF1,LRP5,* *MAP2K6,MAPK1,MAPK3,MAPK8,MAX,NLK,PA2G4,PIK3C2A,PIK3C3,* *PIK3R5,PRKCH,PTK2,RAPGEF1,RASA1,RHOT1,SMAD2,SOS2,TCF4,TGFBR1*
Reelin Signaling in Neurons	0.000125	*APP,ARHGEF6,BLK,CDC42,CNR2,HCK,ITGB1,MAP2K6,MAP4K1,MAPK1,MAPK3,MAPK8,NDEL1,PDK3,PIK3C2A,PIK3C3,PIK3R5,RAPGEF1*
B Cell Receptor Signaling	0.000257	*APBB1IP,CD22,CDC42,CFL2,IGHG1,MALT1,MAP2K6,MAPK1,MAPK3,* *MAPK8,NFAT5,PAG1,PIK3C2A,PIK3C3,PIK3R5,PTEN,PTK2,PTPN6,RPS6KB1,SOS2,SYNJ1,VAV2*

* Gene names in italics = Downregulated.

**Table 7:** Top five functional networks identified by IPA in skin fibroblasts and the associated differentially-regulated genes. See Supplementary Figures for depictions of additional IPA Networks.

Network ID	Genes in Network *	Score	Focus Molecules	Top Diseases and Functions
1	**ARAP2,DDX5,EHBP1,ENPP4,G3BP1,***H1-2***, KCNE4,***H2AC18/H2AC19*,Histone h2a,**IGF2BP3,***KCNE4***, LAMA4,***LGALS8*,LDL-cholesterol, **LMNA,***MAP7, MAPK13***,PTEFb,PRKD2,PRKDC,***RAB33A***,RADX,** **RESF1**,RNA polymerase II,Rnr*,SFN***,SLC20A1, SPATA6, SUPT16H,***SYNGR1***,TCF7L2,TMEM65,** **TMTC1,***UBAP1, VEGFA***,WDR36**	44	30	Cancer, Nervous System Development and Function, Neurological Disease
2	aldehyde dehydrogenase, aldehyde dehydrogenase (NAD),*ALDH1A3***,ALDH3A2,ALDH6A1,ANTXR2**,CDHE/CDHN,*COL27A1*,Ctbp,**CTNNB1,HHEX,** *HILPDA*,HMG CoA synthase,*IFITM1,KCNK1,ME1***, MOB1A,**Neuropilin,*NRP2***,RAB7,RAB8B,***RASSF4***,** **SEMA3C,***SLC16A6,SLC1A5***,***SLC38A5***,ST8SIA1,** **SYNM,TAOK1,TCF,***TDO2***,TRAPPC13**,TWIST, *TWIST2***, UGCG**	35	26	Lipid Metabolism, Molecular Transport, Small Molecule Biochemistry
3	**ADAM10,***ADAM12*,Alpha Actinin,**BEST1**, Cadherin,**CDH2,***COMP***,DCLK1,***DLL1***,GALNT5,** Growth factor,Hedgehog,**HELLS,***HOXA11***,Hsp27, KBTBD11,***KLF2*,MAC,**MOXD1,MYCBP2,NEAT1,** Notch,*PALLD***,PARP,PRSS12,***PTCH1***,RALGPS2,** *RDH10***,***SFRP2***,SH3D19,***SH3PXD2A***,***SOX9***,** SRC(family),*THBS1***,VGLL3**	35	26	Cancer, Organismal Injury and Abnormalities, Reproductive System Disease
4	*ADAM33***,ATM,***BOLA2/BOLA2B***,BRINP1,***C15orf48***,CCDC50,CHMP4B,DLST,FSTL1,***H2BC8,H4C8*,Hif1HISTONE,Histone h3,IgG2b,IL12 (family), Immunoglobulin,**KLF4,KMT2C,***LGALS3BP***,** Mitochondrial complex 1,*NAMPT,NDUFA4L2***,** **NDUFS1,NHLRC3,OGT,***P3H2,PDK1,RAB11FIP1***,** Secretase gamma, *SOD2,* Tnf (family),*TNFAIP3***, TPK1, WARS2**	35	26	Free Radical Scavenging, Neurological Disease, Organismal Injury and Abnormalities
5	**ACSS3,***AMPD3***,CHRDL1,***COL23A1*,Collagen Alpha1,*CSGALNACT1,CYGB,DDIT4*,Ecm,**ENPP5,** *ERO1A,GEM***,***GLDN*,Growth hormone, *IFI27*, IGF receptor,Igfbp,*IGFBP2***,IGFBP5**,Integrin alpha 3 beta 1,**KIAA1671,***LY75,MSC*,Mucin,**MYOC,***NEDD9,* *PAPPA*,Pdi,*PDPN,PTGS1***,RBMS3**,Serine Protease,*STC1, TSHZ3*,Vegf	33	25	Cell Signaling, Free Radical Scavenging, Small Molecule Biochemistry

* Gene names in bold = upregulated; italics = Downregulated.

**Table 8:** Top five functional networks identified by IPA in lymphoblastoid cells and the associated differentially-regulated genes. See Supplementary Figures for depictions of additional IPA Networks.

Network ID	Genes in Network *	Score	Focus Molecules	Top Diseases and Functions
1	*ANGEL1,ARHGAP25,ASCC2,CARD8,DIAPH2,* *DIDO1,HCFC2,HERC3,IKZF5,KDM5C,LACTB2,MBIP,MIS18BP1,NAGK,NFRKB,NRCAM,* **OR7C2,** *PATL1,PEMT,PPHLN1,Presenilin,RGS9,* *RNF26,SGF29,SMC5,SMDT1,TASOR2,TENT4B,* *TMEM62,UTP18,Vegf,WDR37,WDR74,ZNF143,* *ZNF33A*	45	33	Endocrine System Disorders, Hereditary Disorder, Organismal Injury and Abnormalities
2	*AGO3,AGO4,ANTXR2,APTX,ARFGAP3,ARGONAUTE,BICD1,CCP110,CENPJ,CEP290,CNOT2,* *CNTROB,CRTC3,DMAC2L,FBXO22,HMGXB4,* *KIAA0753,KIZ,LYST,MPHOSPH9,MRPL17,MRPL39,MRPL41,MRPS14,OFD1,PAN3,PIBF1,* **Pka** *,* *PPP2R3C,RNASEH2B,SMG9,TNRC6A,TNRC6B,* *TSPYL5,VBP1*	45	33	Cell Cycle, Cellular Assembly and Organization, DNA Replication, Recombination, and Repair
3	*ACIN1,ANKRD10,API5,ARID4A,ATP11C,ATP6V0A1,BCCIP,CENPV,CHORDC1,COX15,DPP9,* *DRG1,FAM98B,GABP,GABPA,GPCPD1,* *HNRNPH3,Immunoglobulin,MIER2,NSRP1,* *PHKG2,PIGN,PRKAB1,RBBP4,RYBP,* **SCGB1D2** *,SINHCAF,ST13,TMEM135,TMEM63A,TMLHE,* *TNPO2,YAF2,YTHDF1,ZFP36L1*	45	33	Cardiovascular Disease, Developmental Disorder, Digestive System Development and Function
4	*APP,BRWD1,C1orf112,CENPI,CHURC1,DCXR,DENND1C,DTD2,DTWD1,EVI2A,FAM50A,* *FMNL3,GIMAP5,GIMAP6,GTDC1,Irp,KCNMB2KLF7,NAA25,PCIF1,PI3k class III,PRKRIP1, PUS7L, SP140L,T2r,TAS2R20,TAS2R4,TAS2R46,* *TAS2R50,TCP11L2,TICRR,TIGD1,ZMYM5,* *ZNF431,ZNF75A*	42	32	Hereditary Disorder, Metabolic Disease, Organismal Injury and Abnormalities
5	*Adaptor protein 1,ADPGK,alcohol group acceptor phosphotransferase,AP1G2,AP1S3,ARL15,CEP83,* *DCAF10,DPY19L3,DYRK1A,Exocyst,GGA2,* *HAUS2,HIPK1,KLHL36,MAP2K6,MAPK3,* *MZT2A,PHLPP1,PIGB,POMK,PRKCH,PRKX,* *RALGAPA1,RALGAPA2,RALGAPB,RFX7,* *RIPOR2,SCAF11,TMEM120A,TUBG2,TUBGCP4,UFC1,ZNF507,ZNFX1*	42	32	Auditory Disease, Cellular Development, Cellular Growth and Proliferation

* Gene names in bold = upregulated; italics = Downregulated.

## Data Availability

The skin fibroblast and lymphoblastoid cell lines were generated at the Department of Genetics and Genomics Sciences of the Icahn School of Medicine at Mount Sinai. The Affymetrix .CEL files for the fibroblast microarrays have the NCBI GEO #GSE16524 and for the lymphoblast microarrays they were assigned the NCBI GEO #GSE160893.

## Supplementary Materials

The following are available online at https://www.mdpi.com/1660-4601/18/4/1997/s1, Figure S1: Hierarchical cluster analysis of microarray results from (A) Fibroblast and (B) Lymphoblast cell lines. Figure S2: Sample Signal plots of twelve selected differentially regulated genes in fibroblast (A) and lymphoblastoid (B) cell lines; Figure S3: Differentially regulated genes in SS fibroblasts in the VEGFA-VEGFR2 signaling pathway in endothelial cells; Figure S4: Differentially regulated genes in SS fibroblasts in the nuclear receptors meta-Pathway; Figure S5: Differentially regulated genes in SS fibroblasts in the endothelin pathway; Figure S6: Differentially regulated genes in SS fibroblasts in the PI3K-Akt signaling pathway; Figure S7: Differentially regulated genes in SS lymphoblasts in the EGF/EGFR signaling pathway; Figure S8: Differentially regulated genes in SS lymphoblasts in the TGF beta signaling pathway; Figure S9: Differentially regulated genes in SS lymphoblasts in the JAK/STAT WikiPathway; Figure S10: Differentially regulated genes in SS fibroblasts in IPA network 1: Cancer, nervous system development and function, neurological disease; Figure S11: Differentially regulated genes in SS fibroblasts in IPA network 3: Free radical scavenging, neurological disease, organismal injury and abnormalities; Figure S12: Differentially regulated genes in SS fibroblasts in IPA network 4: cell-to-cell signaling and interaction, cellular assembly and organization, tissue development; Figure S13: Differentially regulated genes in SS fibroblasts in IPA network 18: Cell-to-cell signaling and interaction, cellular assembly and organization, tissue development; Figure S14: Differentially regulated genes in SS fibroblasts in IPA network 23: Cardiovascular disease, cellular movement, organismal injury and abnormalities; Figure S15: Differentially regulated genes in SS Lymphoblasts in IPA network 1: Endocrine system disorders, hereditary disorder, organismal injury and abnormalities; Figure S16: Differentially regulated genes in SS Lymphoblasts in IPA network 2: Cell cycle, cellular assembly and organization, DNA replication, recombination, and repair; Figure S17: Differentially regulated genes in SS Lymphoblasts in IPA network 3: Cardiovascular disease, developmental disorder, digestive system development and function; Figure S18: Differentially regulated genes in SS Lymphoblasts in IPA network 5: Auditory disease, cellular development, cellular growth and proliferation; Table S1: Differentially-regulated genes in SS fibroblasts; Table S2: Differentially-regulated genes in SS lymphoblastoid cells; Table S3: Differentially expressed genes detected in both skin fibroblast and lymphoblastoid cell lines; Table S4: Wiki Pathways affected in SS fibroblasts; Table S5: Wiki Pathways affected in SS lymphoblasts; Table S6: IPA canonical pathways affected in SS fibroblasts; Table S7: IPA canonical pathways affected in SS Lymphoblasts; Table S8: IPA functional pathways affected in SS fibroblasts; Table S9: IPA functional pathways affected in SS lymphoblasts.
