# Supplementary material for: Expression Profiling Identifies TWIST2 Target Genes in Setleis Syndrome Patient Fibroblast and Lymphoblast Cells
Source: Int J Environ Res Public Health. 2021 Feb 19;18(4):1997. doi: 10.3390/ijerph18041997 (PMC7922891; doi:10.3390/ijerph18041997)
Supplement: Supplementary file 1 [file ijerph-18-01997-s001.pdf]

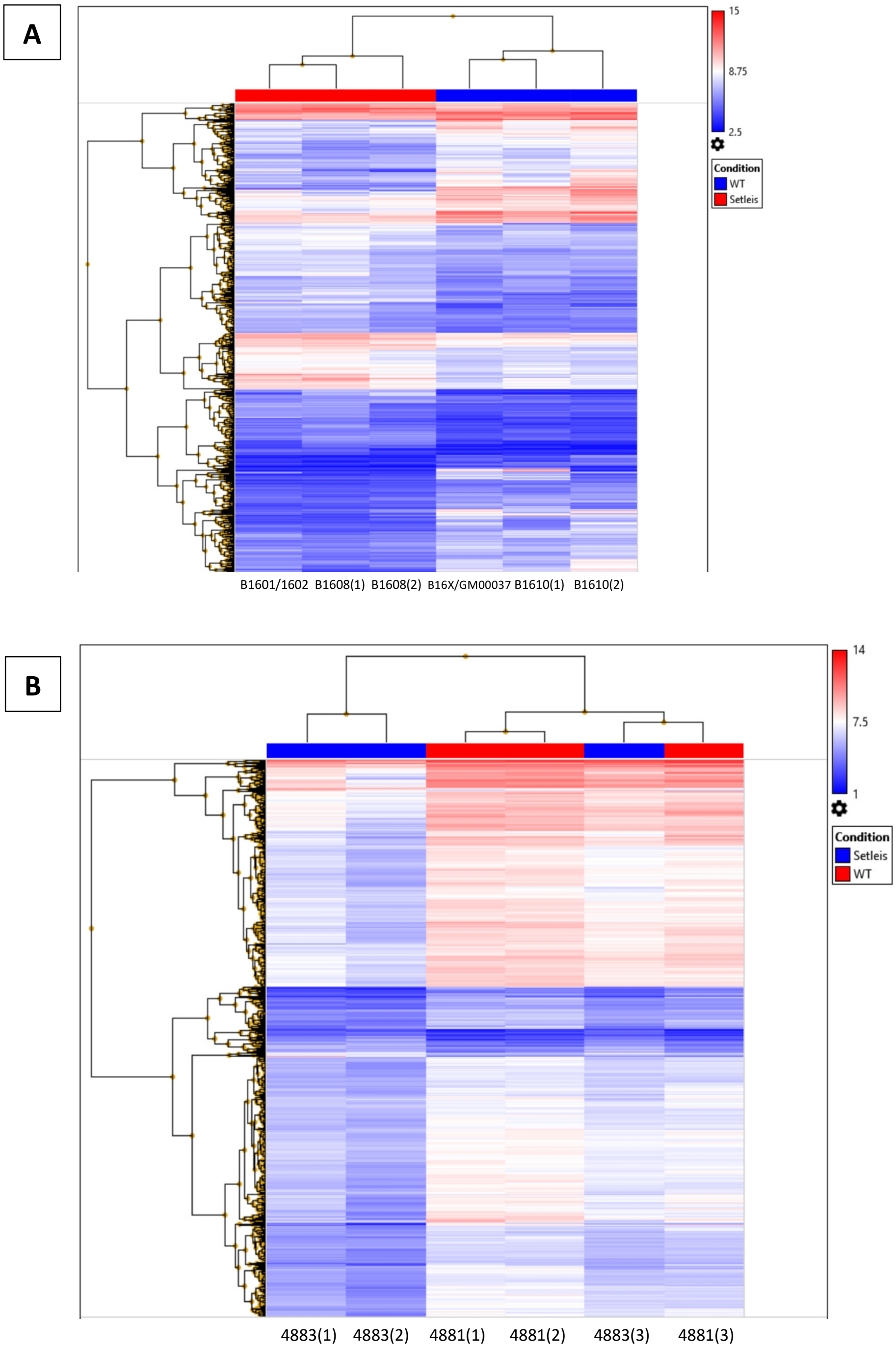

Supplementary Figure S1. Hierarchical cluster analysis of microarray results from A) Fibroblast and B) Lymphoblast cell lines.

A

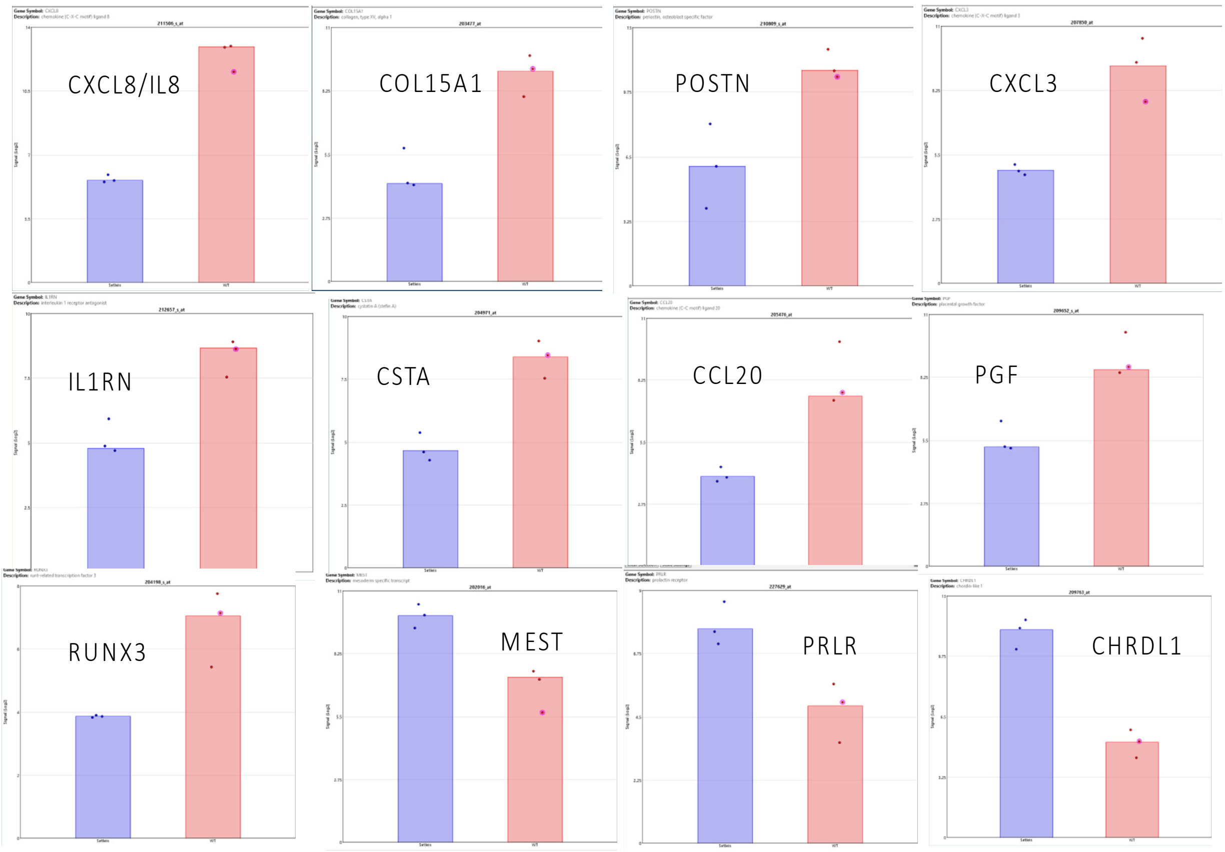

CXCL8/IL8
COL15A1
POSTN
CXCL3
IL1RN
CSTA
CCL20
PGF
RUNX3
MEST
PRLR
CHRDL1

B

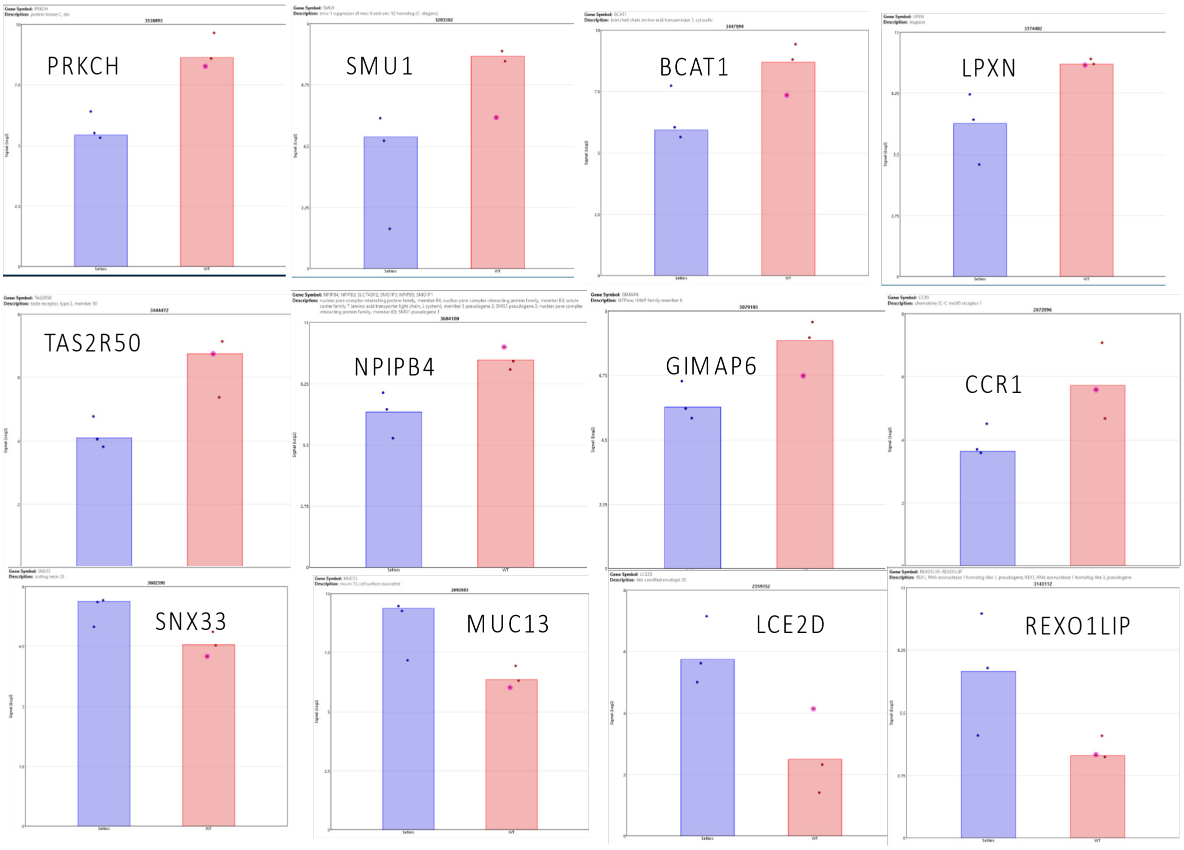

Supplementary Figure S2. Sample Signal plots of twelve selected differentially regulated genes in fibroblast (A) and lymphoblastoid (B) cell lines. The microarray log2 values of the expression levels for each gene are presented for PR SS patient (blue) and PR control cell lines (red).

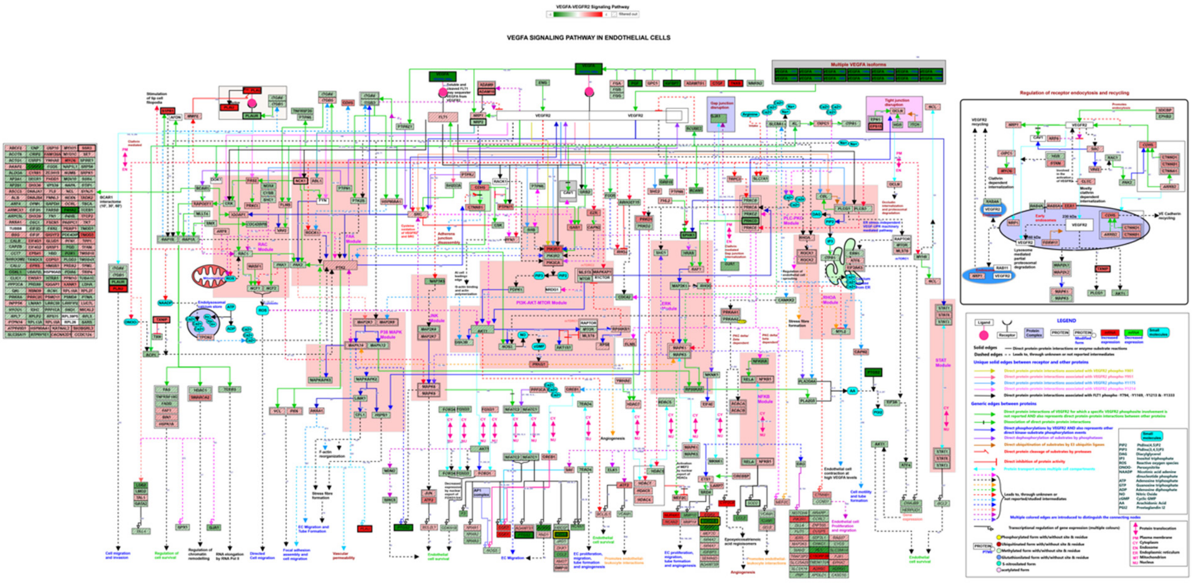

Supplementary Figure S3. Differentially regulated genes in SS fibroblasts in the VEGFA-VEGFR2 signaling pathway in endothelial cells. Vascular endothelial growth factor (VEGF) is the principal angiogenic growth factor modulating neovascularization [34]. This WikiPathway is described by its creators as follows: "Individual signaling events in VEGFR2 signaling networks leading to cell proliferation, migration and survival were identified and categorized into protein-protein interactions, enzyme-catalyzed events, activation/inhibition reactions, transport of protein across subcellular compartments, and gene regulation events" in https://www.wikipathways.org/index.php/Pathway:WP3888.

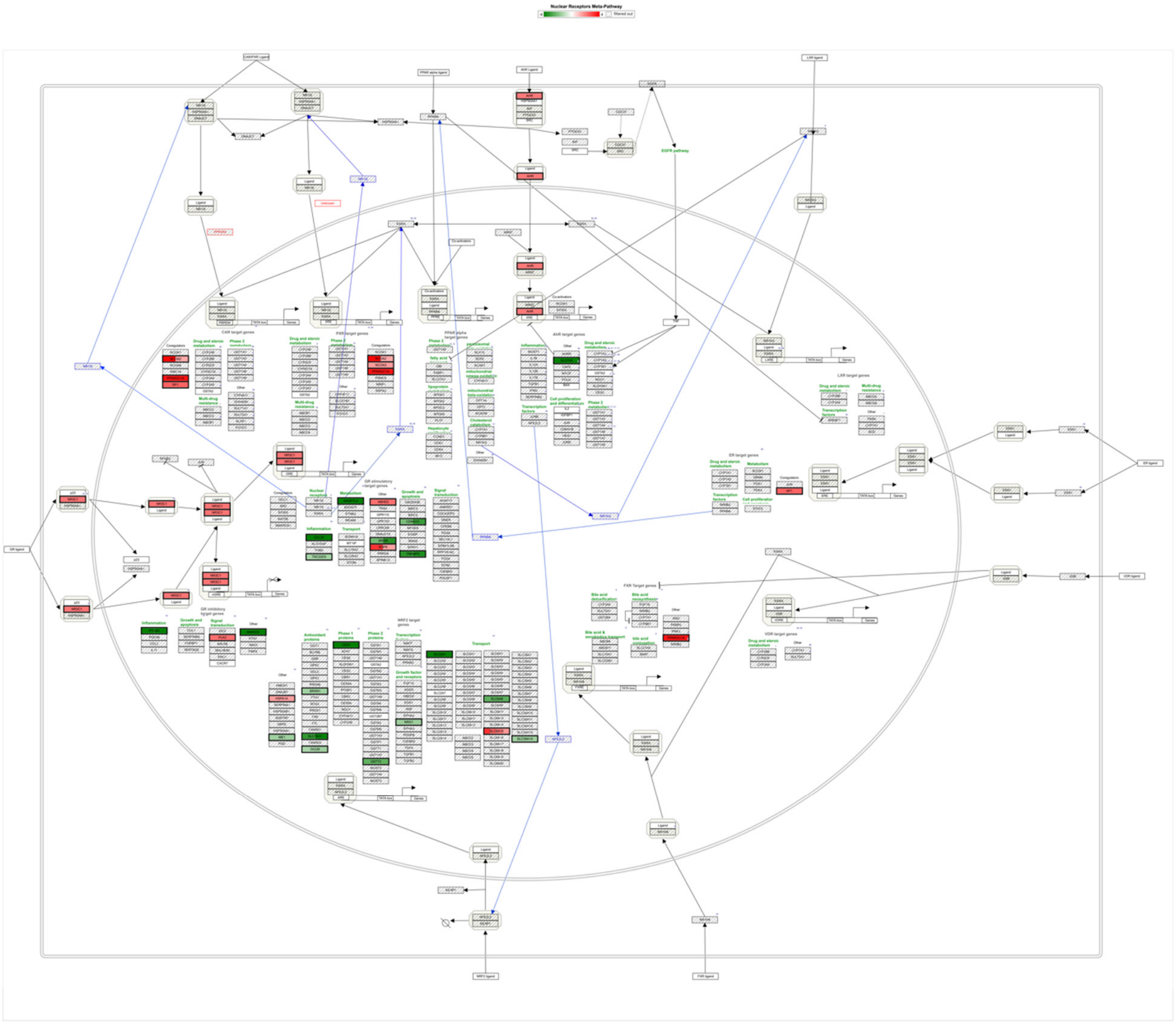

Supplementary Figure S4. Differentially regulated genes in SS fibroblasts in the nuclear receptors meta-Pathway. The following description was obtained from: (https://www.wikipathways.org/index.php/Pathway:WP2882). “Nuclear receptors are transcription factors that directly bind to DNA and regulate the expression of adjacent genes. Ligand binding to a nuclear receptor results in a conformational change in the receptor, which, in turn, activates the receptor, resulting in induction or repression of target genes. Nuclear receptors play key roles in both embryonic development and adult homeostasis.”

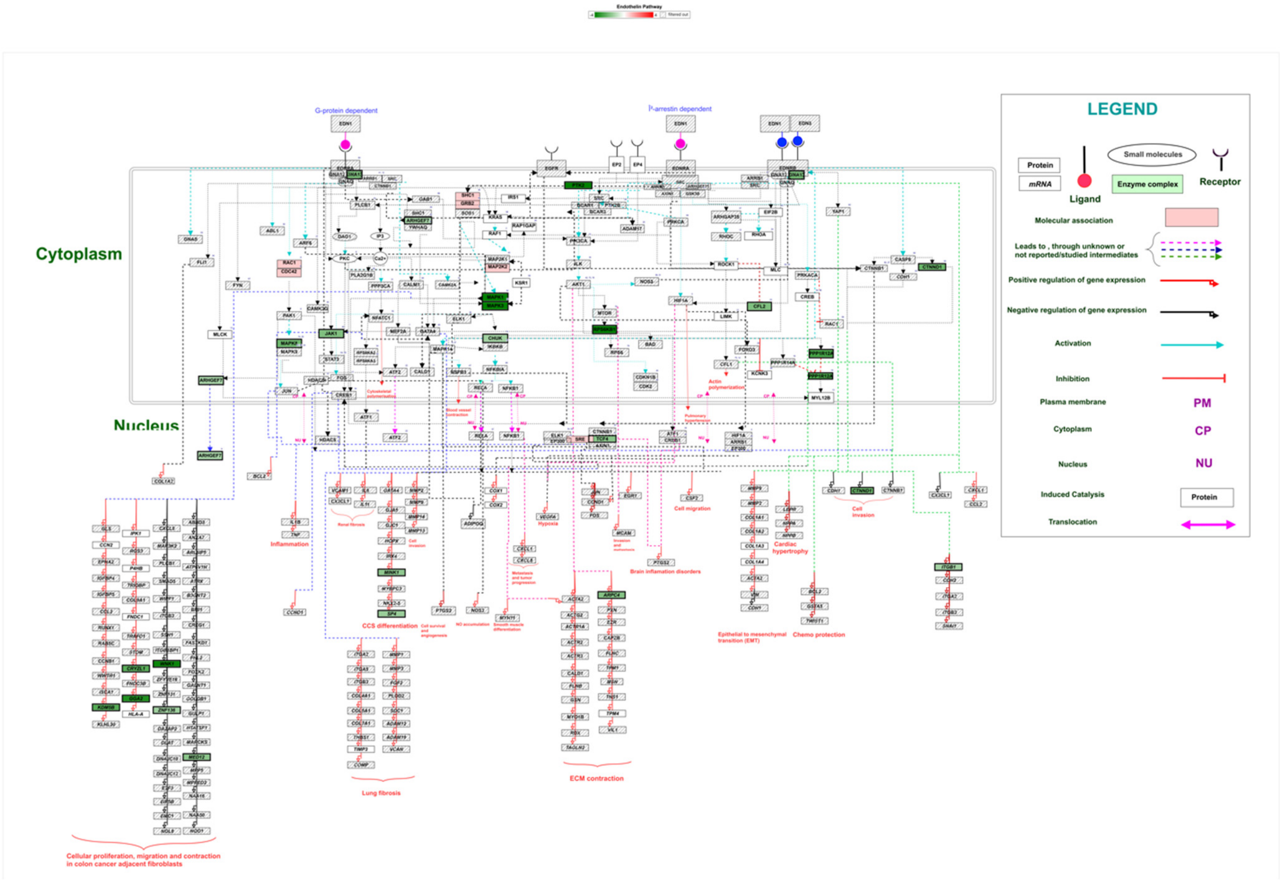

Supplementary Figure S5. Differentially regulated genes in SS fibroblasts in the endothelin pathway. “Endothelin is a strong peptide well known for its vasoconstriction activities. It is known for regulating extensive physiological and pathophysiological activities.” This WikiPathway description was obtained from https://www.wikipathways.org/index.php/Pathway:WP4857.

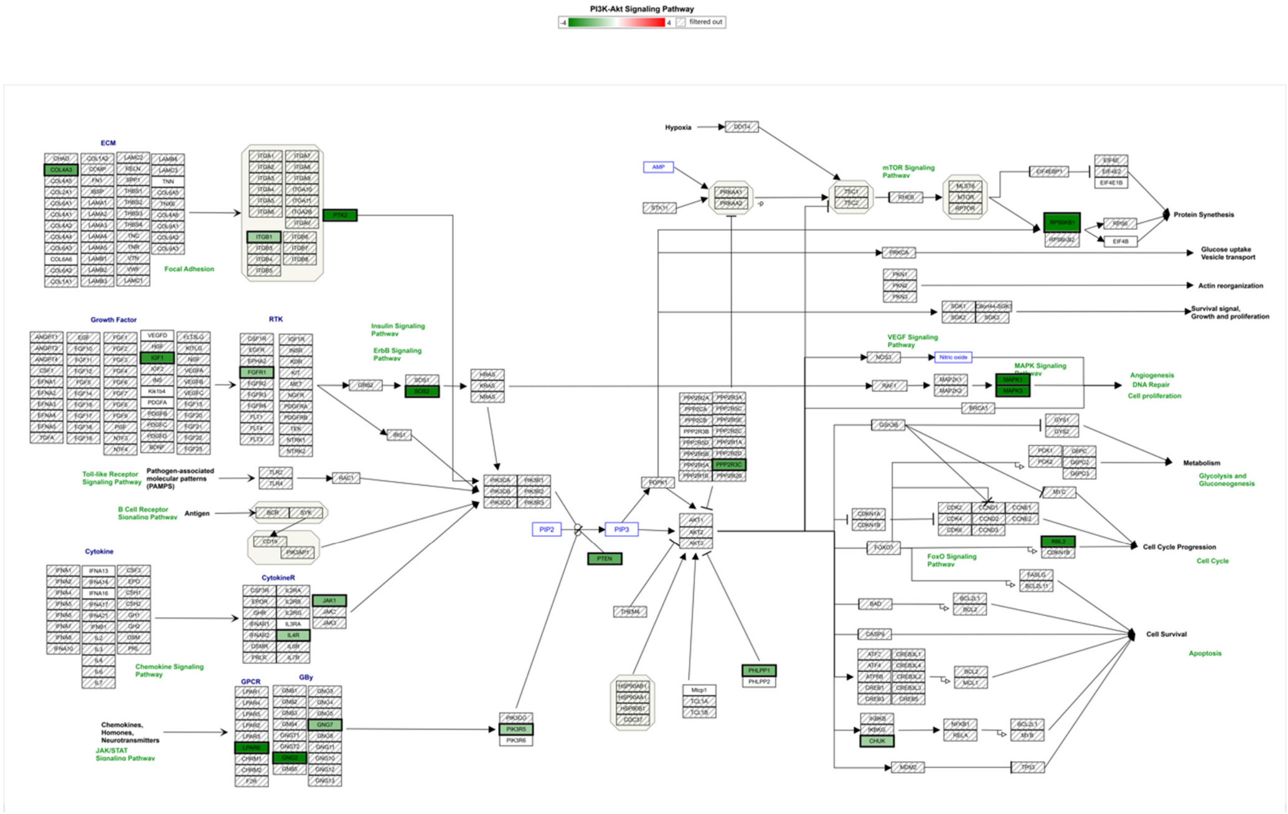

Supplementary Figure S6. Differentially regulated genes in SS fibroblasts in the PI3K-Akt signaling pathway. The following description was obtained from https://www.wikipathways.org/index.php/Pathway:WP4172. "The PI3K-Akt signaling pathway can be activated by many types of cellular stimuli or toxic insults and regulates many fundamental cellular functions. Activated PI3K phosphorylates AKT, thereby activating it. Once active, Akt controls a number of downstream cellular processes, including apoptosis, protein synthesis, metabolism, and cell cycle, by phosphorylating a range of substrates."

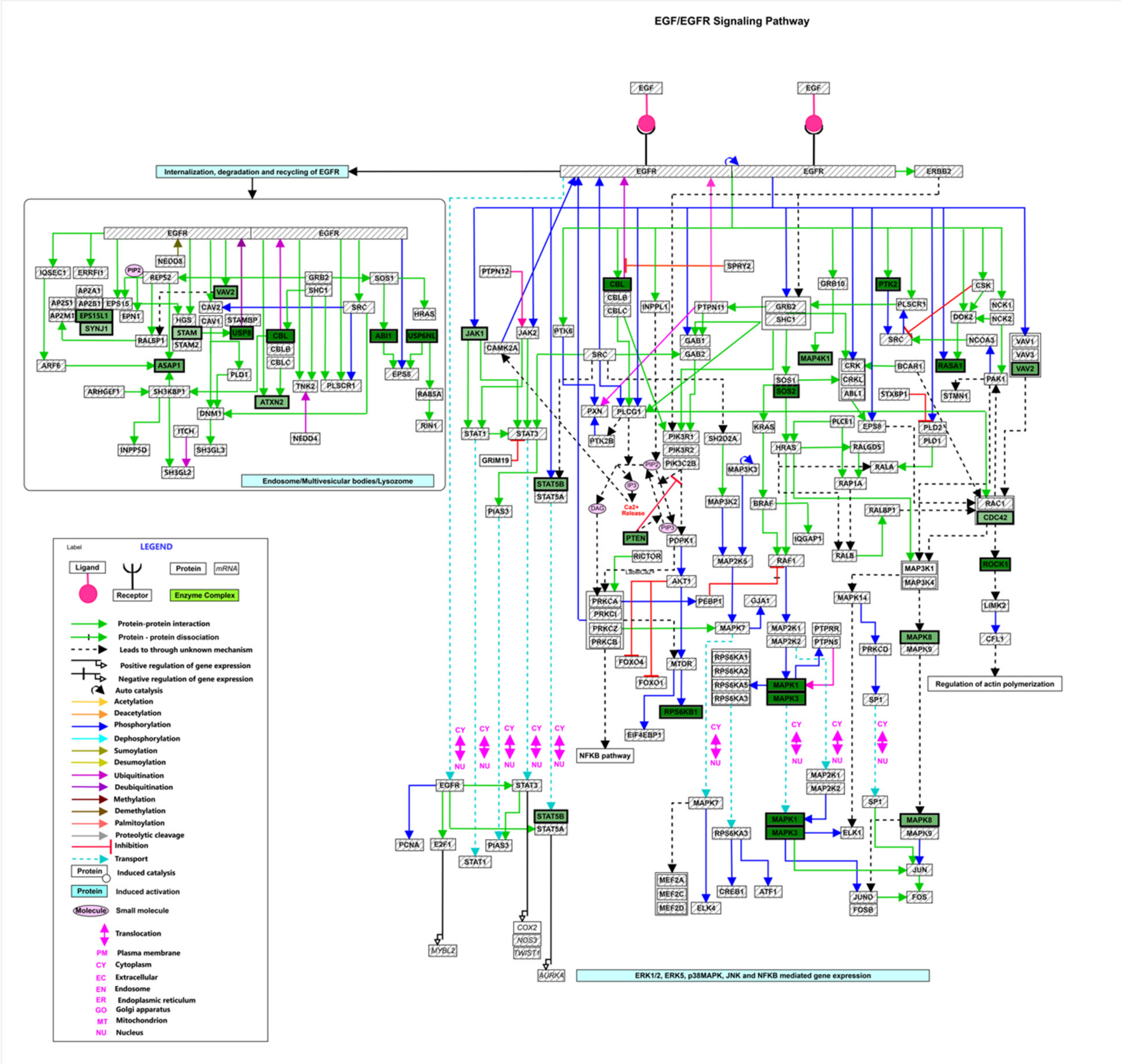

Supplementary Figure S7. Differentially regulated genes in SS lymphoblasts in the EGF/EGFR signaling pathway. The following description was obtained from https://www.wikipathways.org/index.php/Pathway:WP437. "Epidermal growth factor receptor (EGFR) also known as ErbB1/HER1 is a member of the ErbB family of receptor tyrosine kinases. The EGF/EGFR system induces growth, differentiation, migration, adhesion and cell survival through various inter-acting signaling pathways."

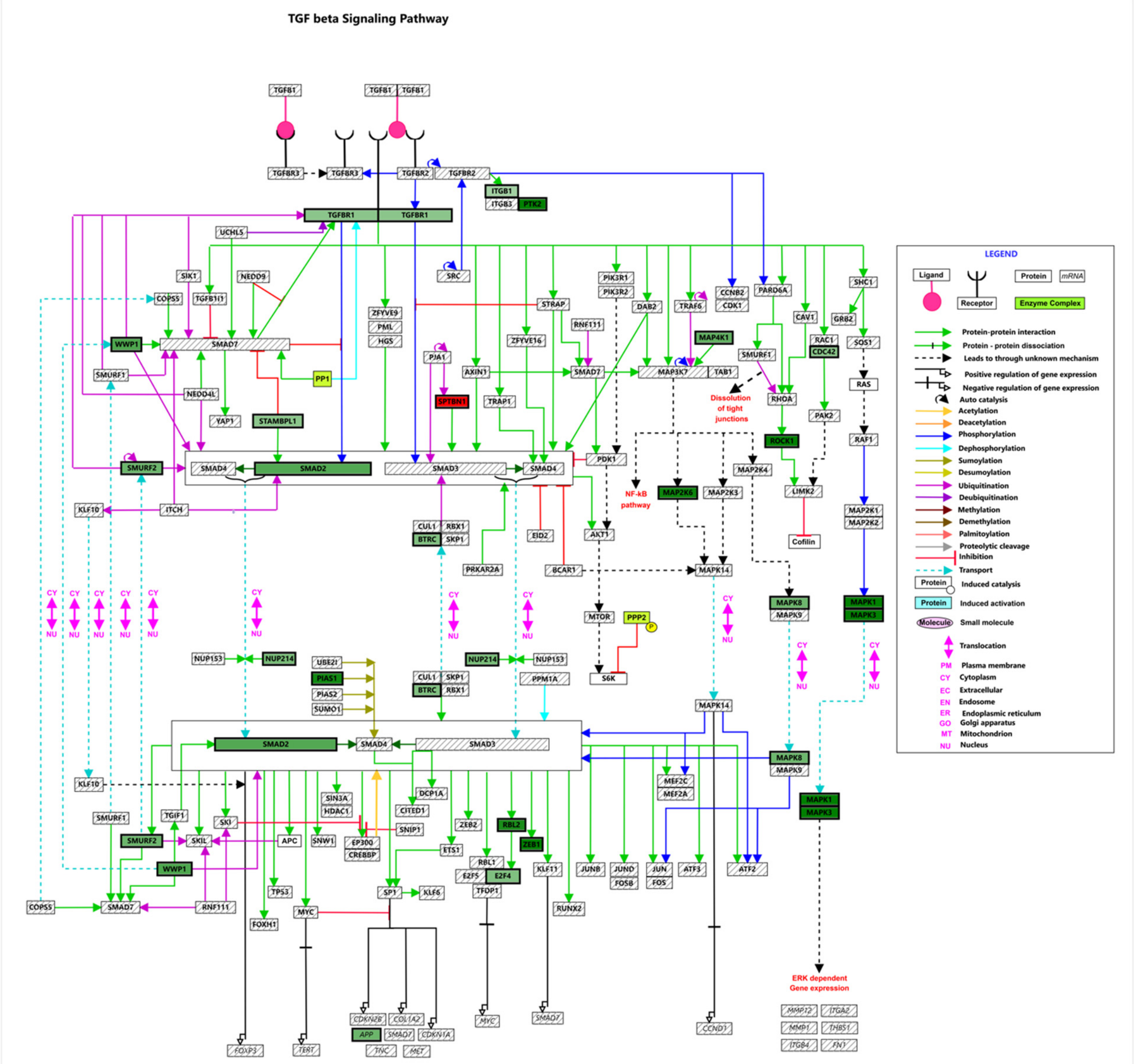

Supplementary Figure S8. Differentially regulated genes in SS lymphoblasts in the TGF beta signaling pathway. Description obtained from https://www.wikipathways.org/index.php/Pathway:WP366. " TGF-β ligands belong to a large superfamily of cytokines that bears its name (TGF-β Superfamily) and includes bone morphogenic proteins, activins, inhibin, growth/differentiation factors, Mullerian inhibiting substance, Nodal, and several other structurally-related polypeptides."

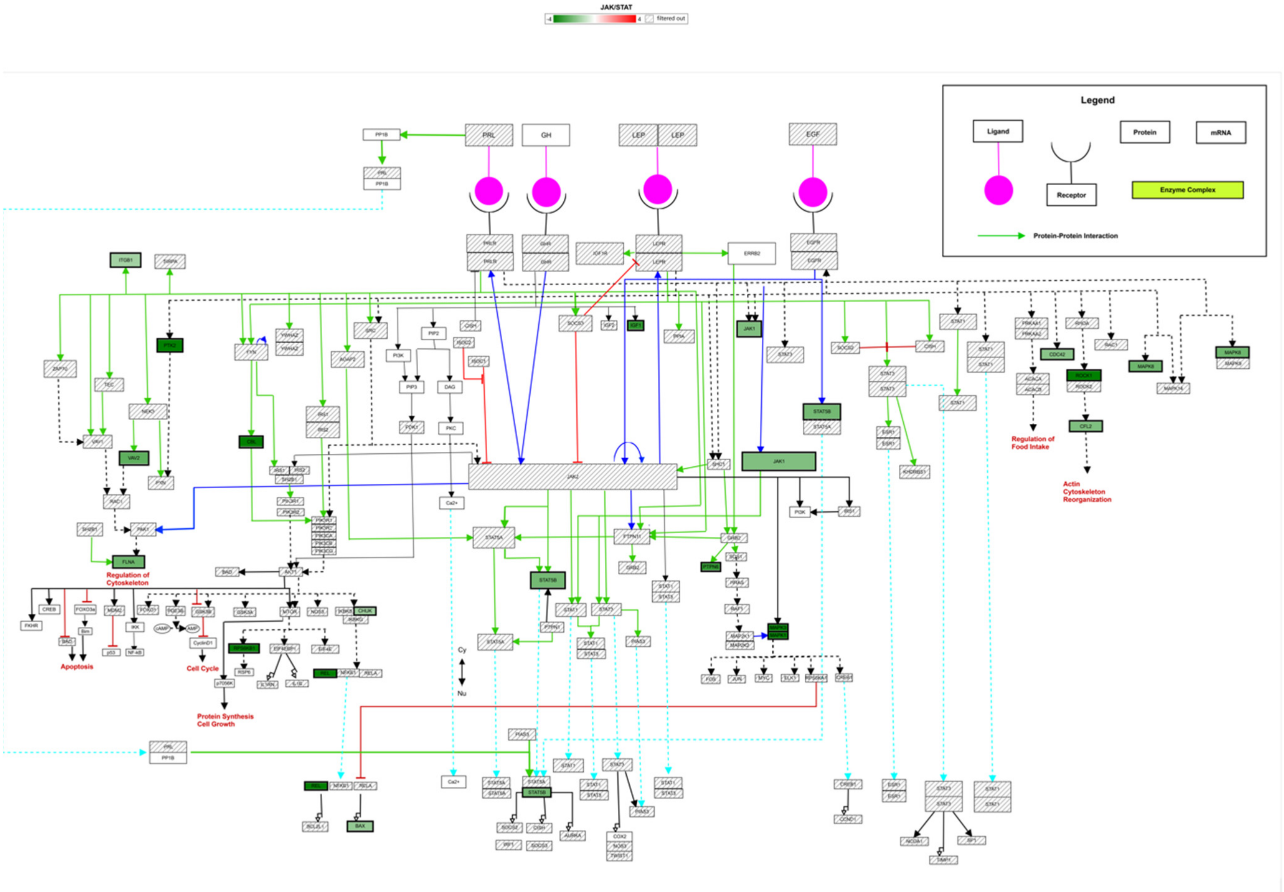

Supplementary Figure S9. Differentially regulated genes in SS lymphoblasts in the JAK/STAT WikiPathway. The following description was obtained from https://www.wikipathways.org/index.php/Pathway:WP2593. "The Janus kinase/signal transducers and activators of transcription (JAK/STAT) pathway is the central signalling mechanism for many growth factors and cytokines. JAK activation stimulates cell proliferation, differentiation, cell migration, and apoptosis. These events are critical to immune development, adipogenesis, sexually dimorphic growth, and other processes."

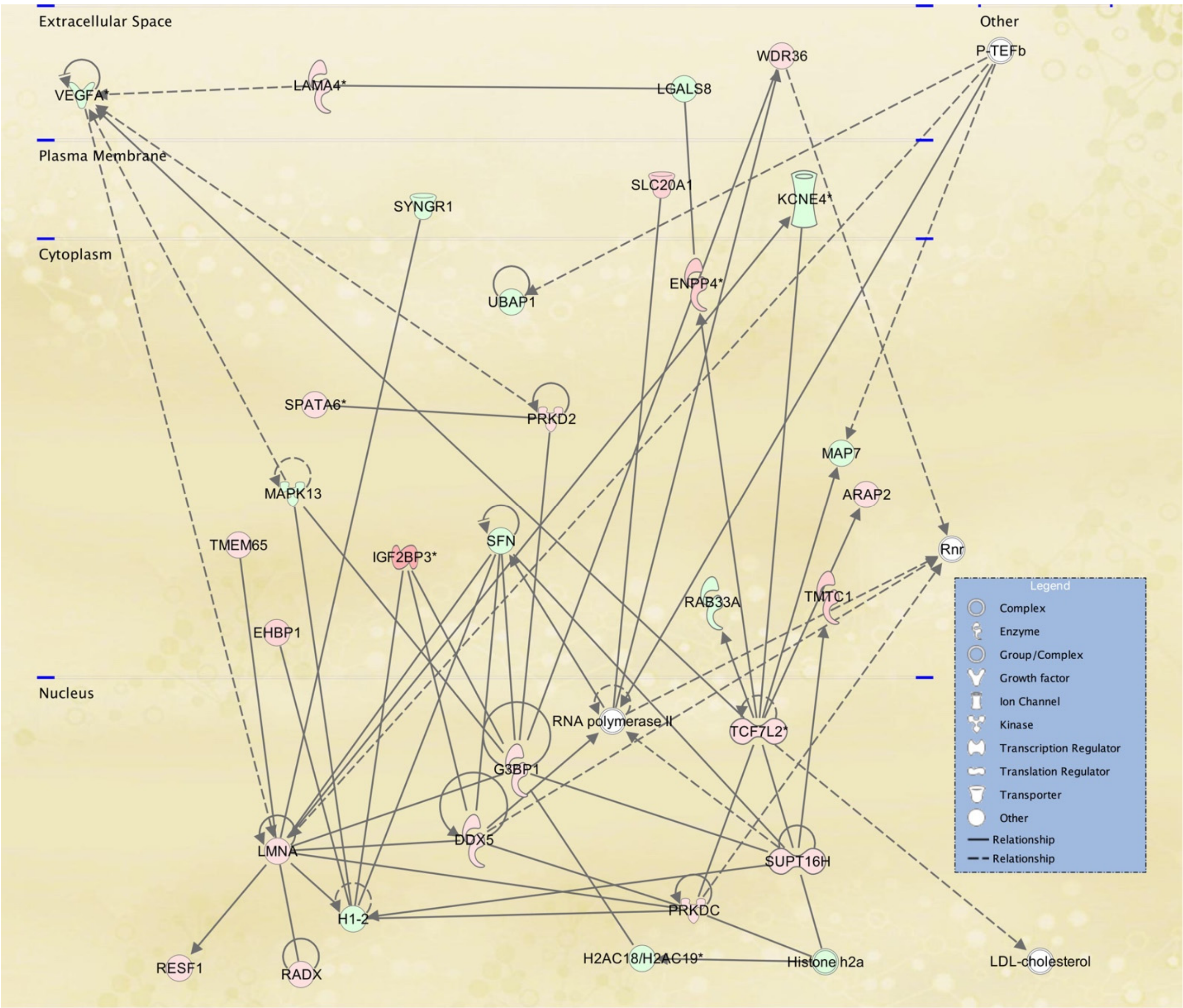

Supplementary Figure S10. Differentially regulated genes in SS fibroblasts in IPA network 1: Cancer, nervous system development and function, neurological disease.

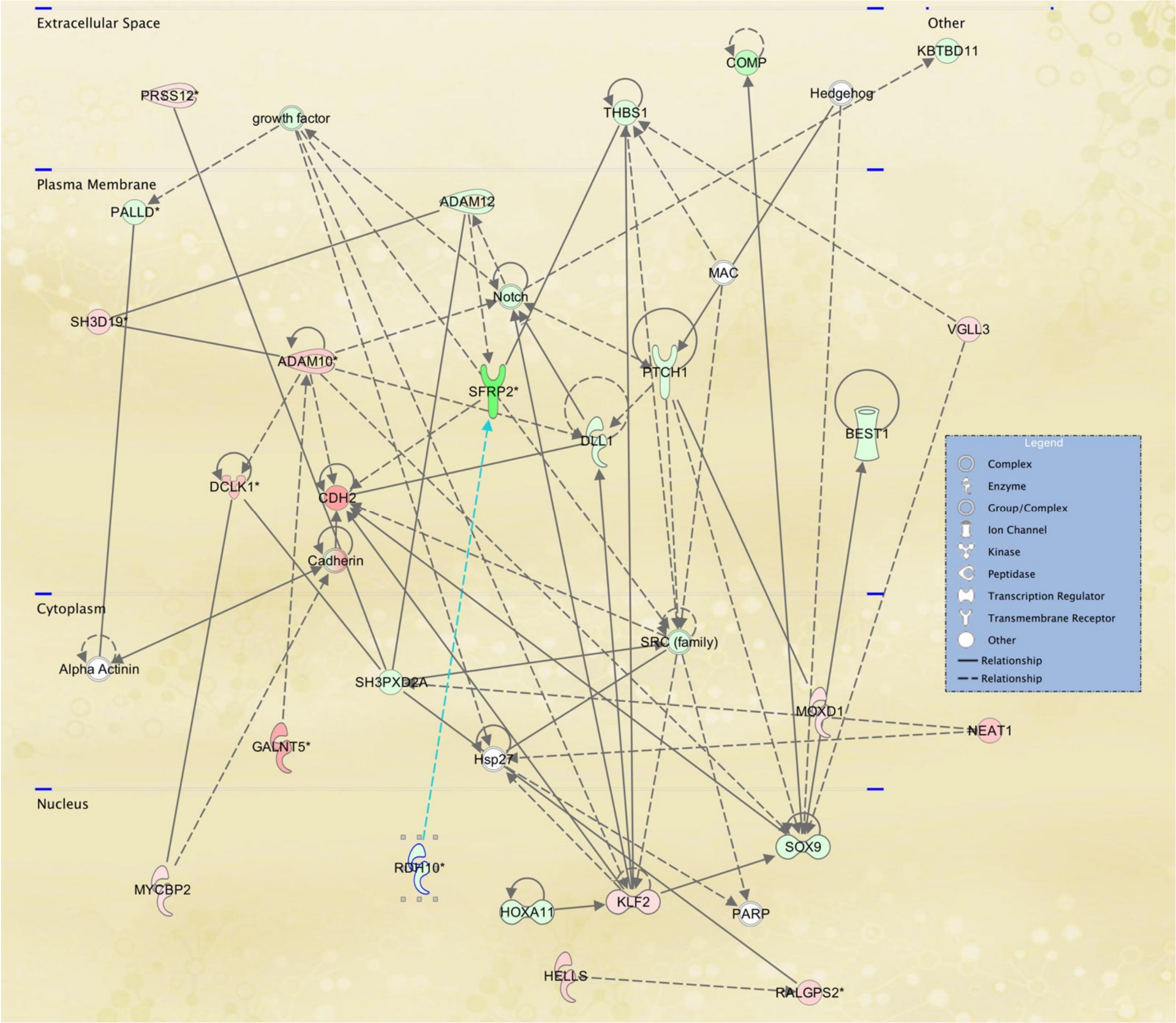

Supplementary Figure S11. Differentially regulated genes in SS fibroblasts in IPA network 3: Free radical scavenging, neurological disease, organismal injury and abnormalities.

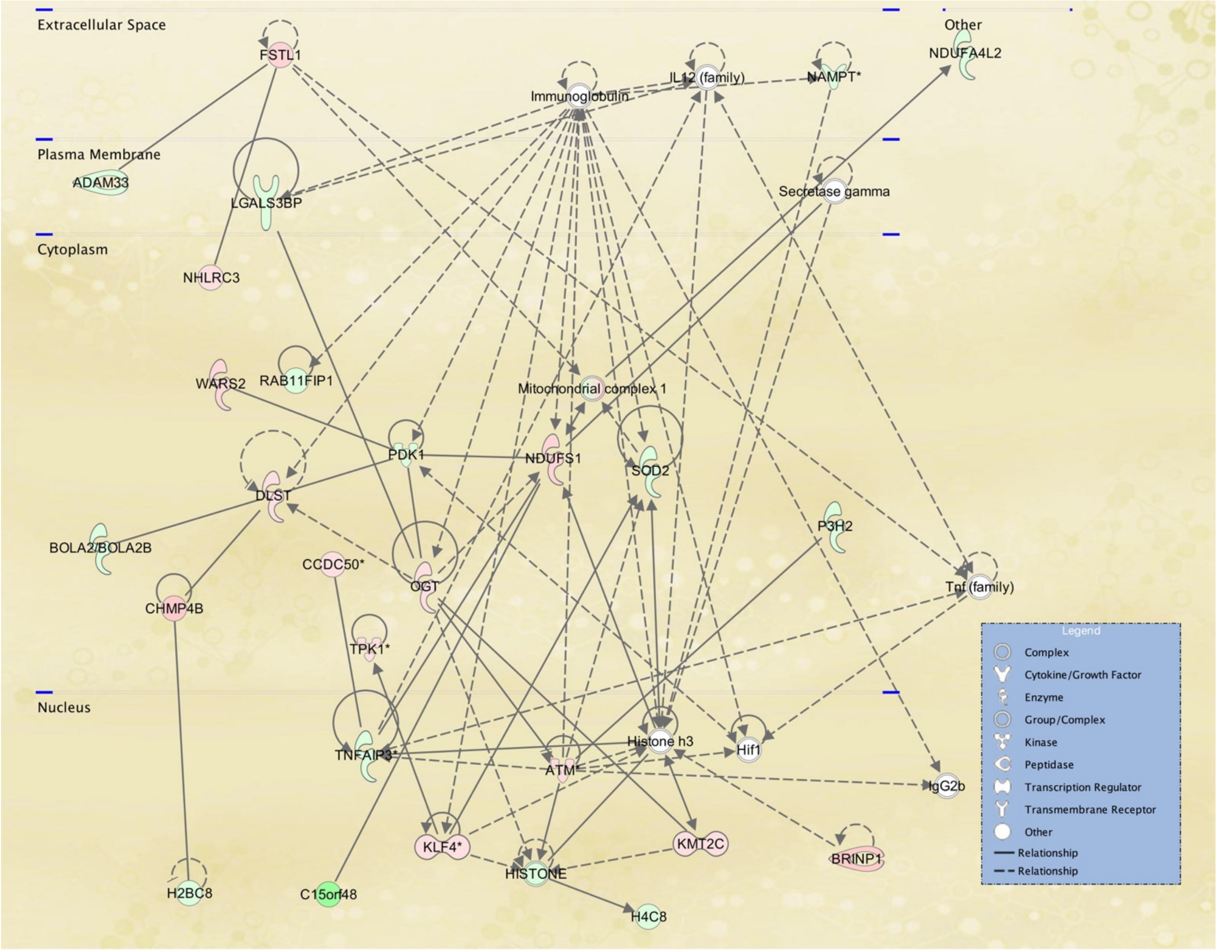

Supplementary Figure S12. Differentially regulated genes in SS fibroblasts in IPA network 4: cell-to-cell signaling and interaction, cellular assembly and organization, tissue development.

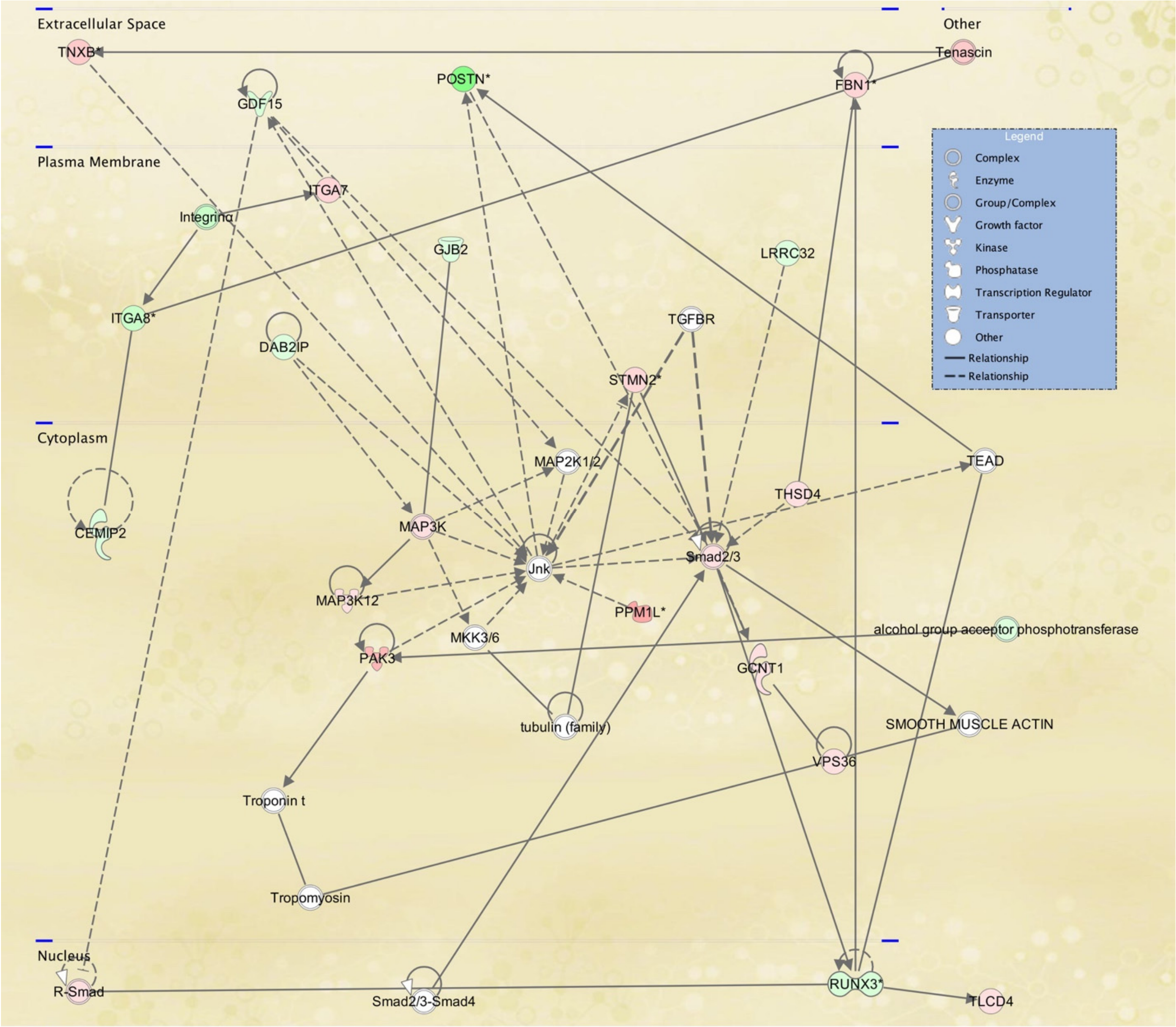

Supplementary Figure S13. Differentially regulated genes in SS fibroblasts in IPA network 18: Cell-to-cell signaling and interaction, cellular assembly and organization, tissue development. This network includes *POSTN*, a known Twist1/TWIST2 regulated gene [29].

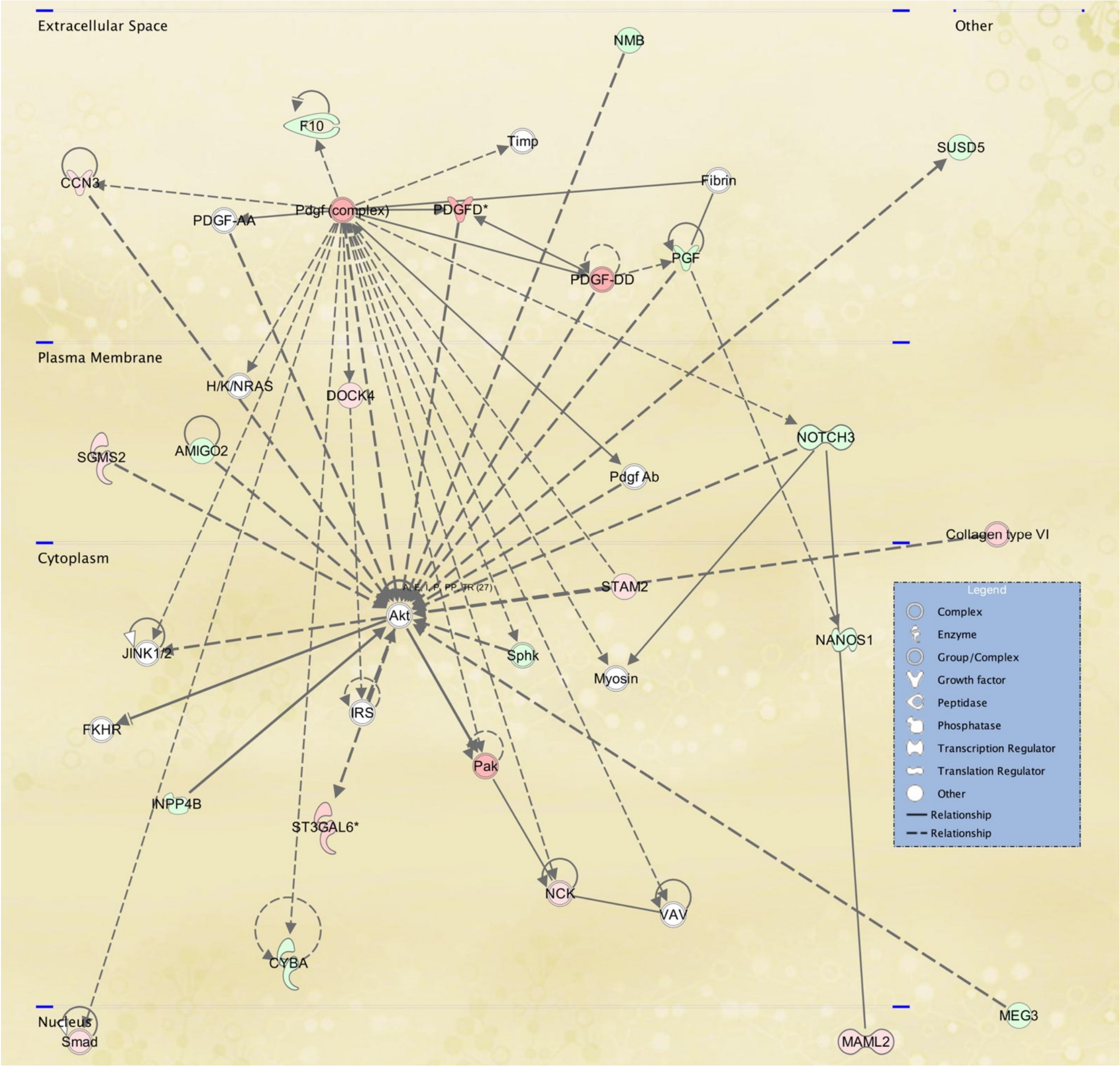

Supplementary Figure S14. Differentially regulated genes in SS fibroblasts in IPA network 23: Cardiovascular disease, cellular movement, organismal injury and abnormalities.

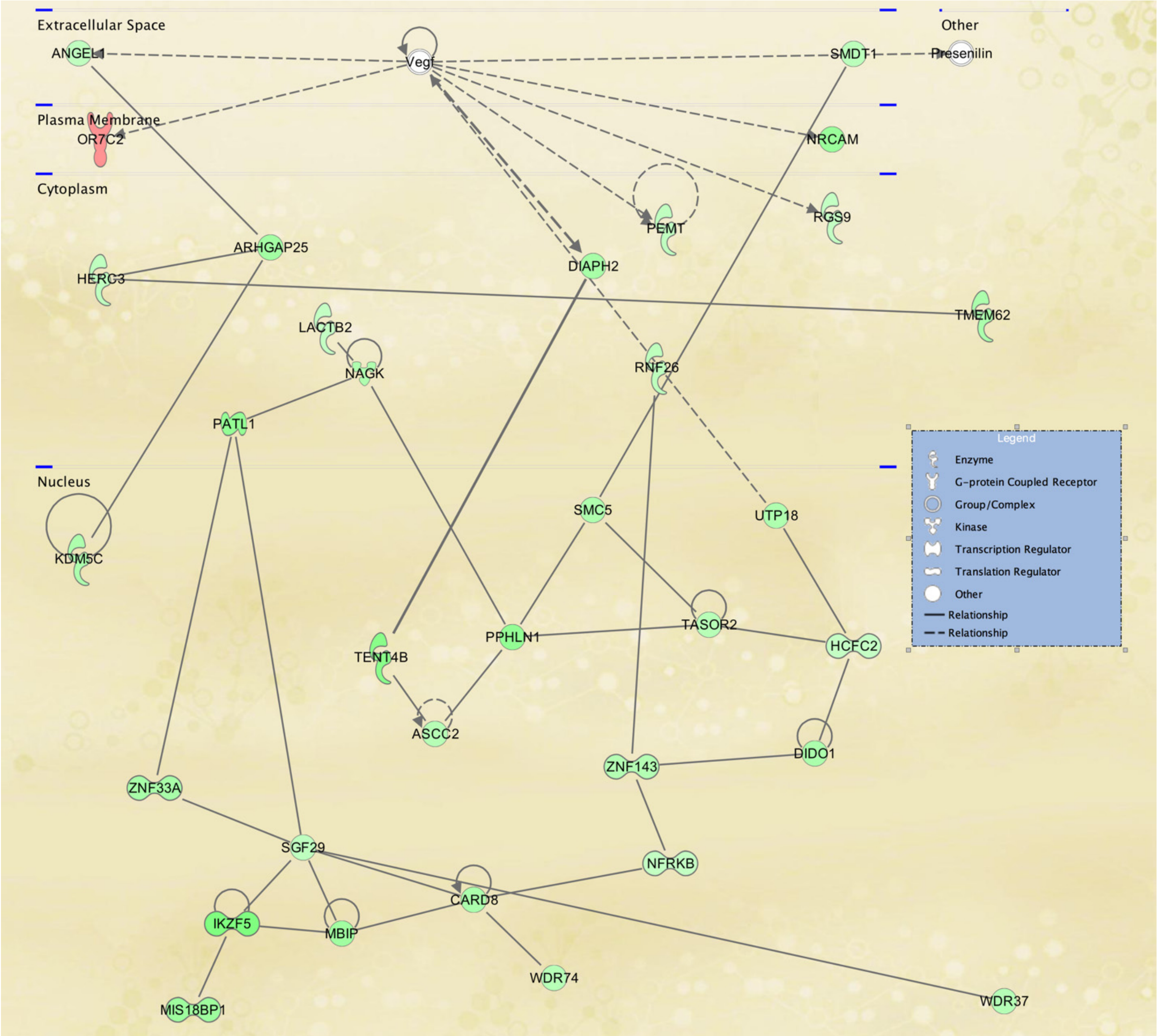

Supplementary Figure S15. Differentially regulated genes in SS Lymphoblasts in IPA network 1: Endocrine system disorders, hereditary disorder, organismal injury and abnormalities.

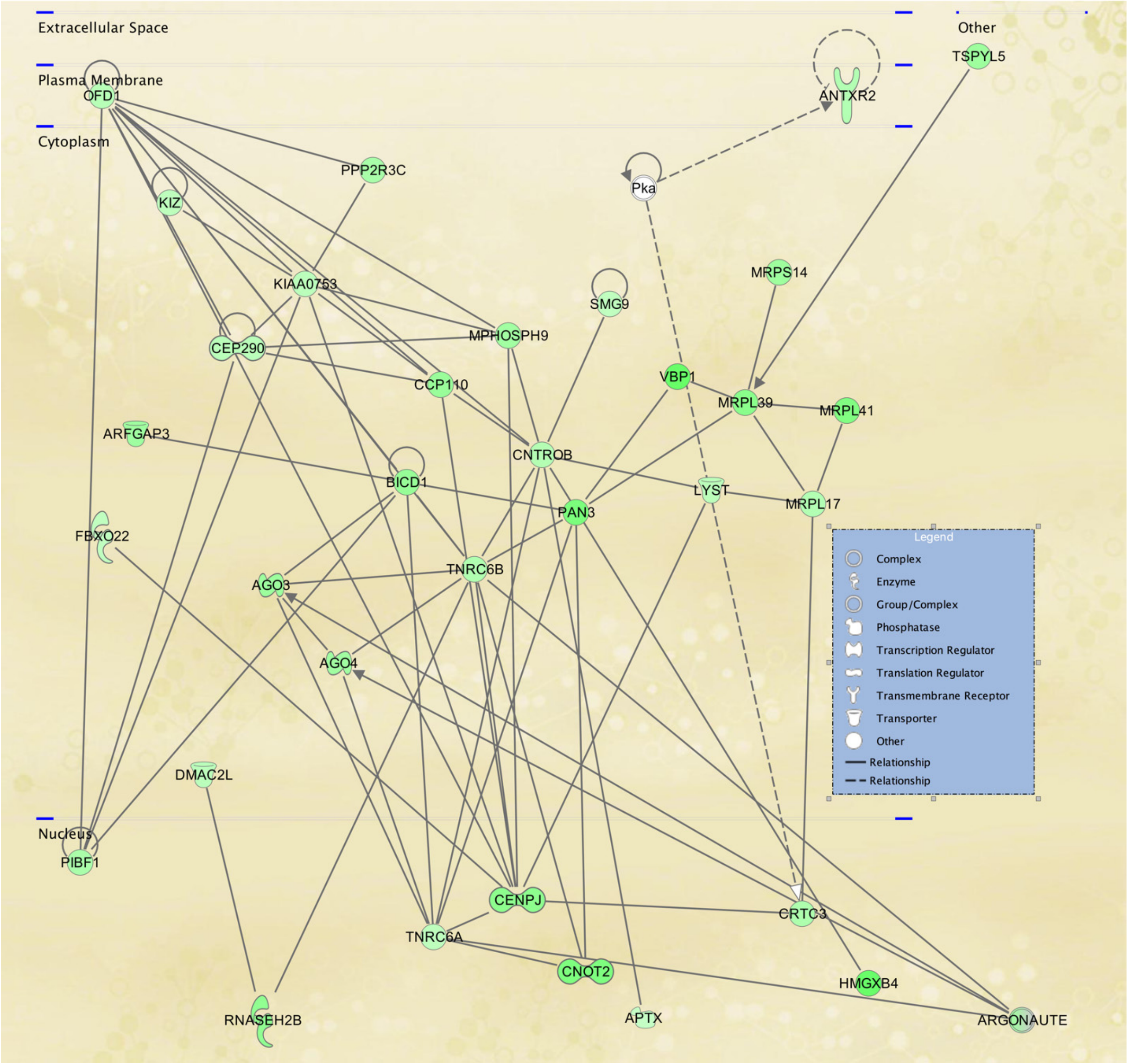

Supplementary Figure S16. Differentially regulated genes in SS Lymphoblasts in IPA network 2: Cell cycle, cellular assembly and organization, DNA replication, recombination, and repair.

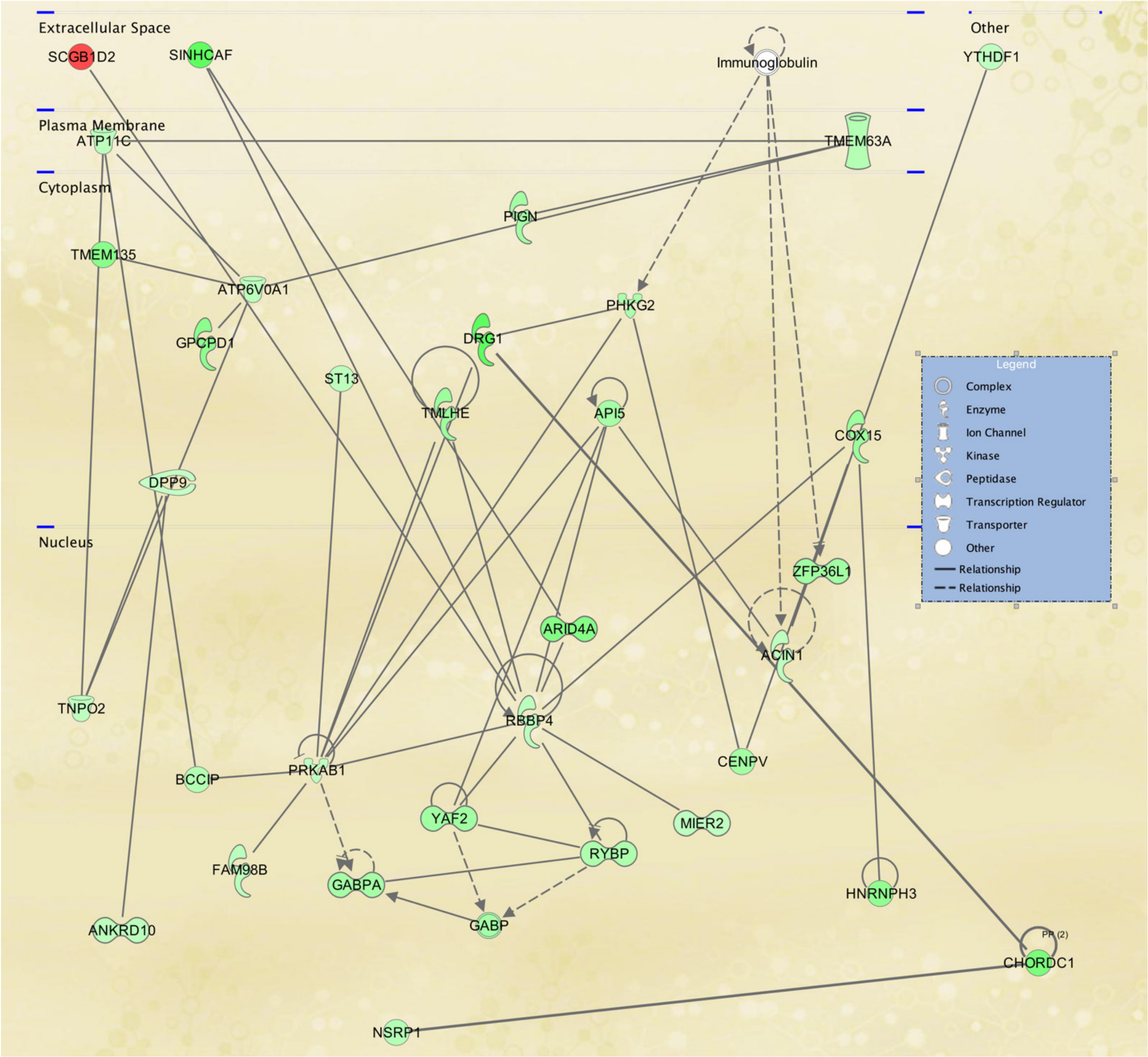

Supplementary Figure S17. Differentially regulated genes in SS Lymphoblasts in IPA network 3: Cardiovascular disease, developmental disorder, digestive system development and function.

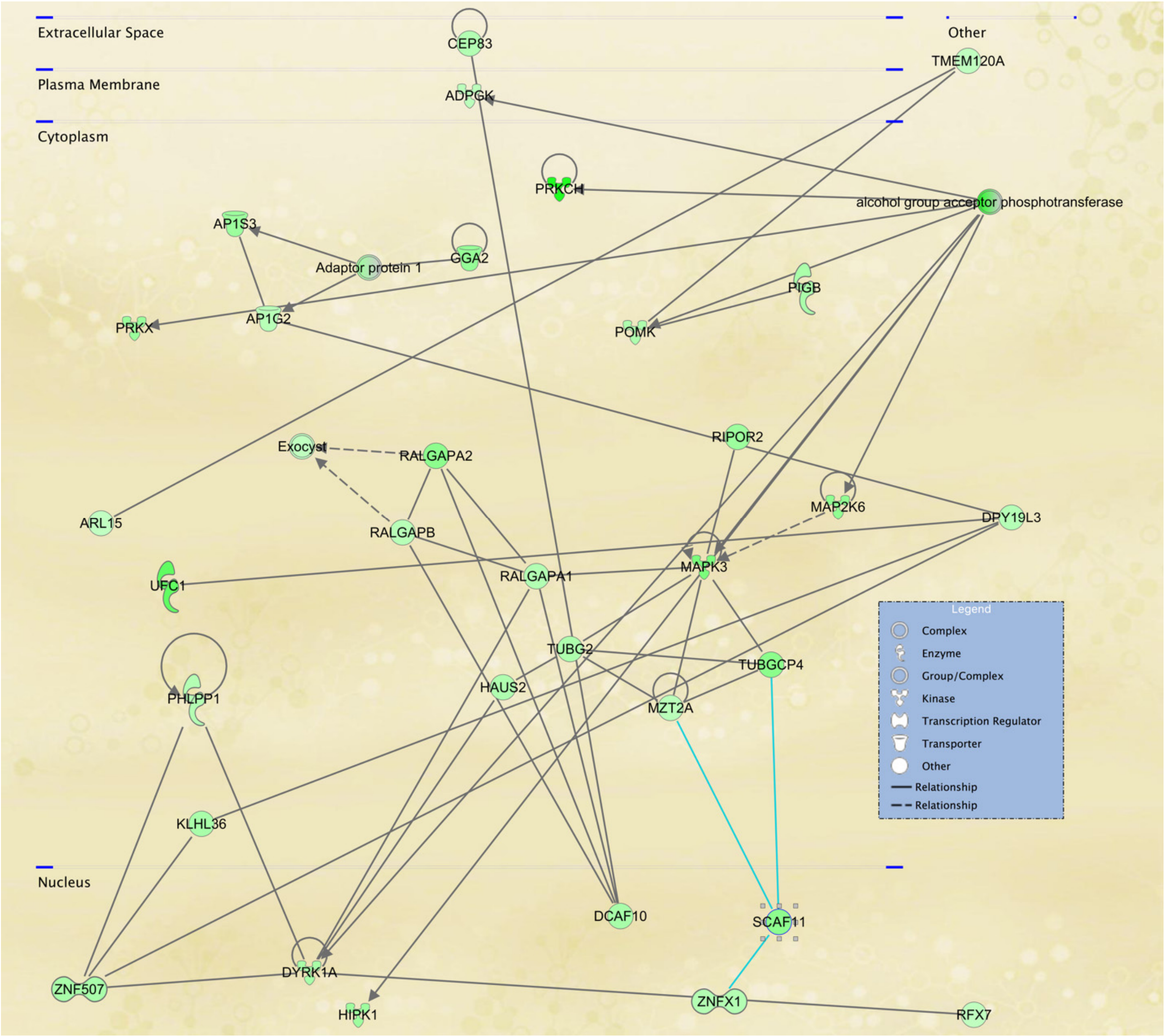

Supplementary Figure 18. Differentially regulated genes in SS Lymphoblasts in IPA network 5: Auditory disease, cellular development, cellular growth and proliferation.

Supplementary Table S1. Differentially-regulated genes in SS fibroblasts

Affymetrix ID	Setleis Avg (log2)	WT Avg (log2)	Setleis Std Dev	WT Std Dev	Fold Change	P-val	Gene Symbol	Description
**209763_at**	11.19	5.12	0.82	0.76	67.13	1.30E-05	CHRDL1	chordin-like 1
**235281_x_at**	9.61	5.09	2.68	0.03	22.94	0.045	AHNAK	AHNAK nucleoprotein
**209613_s_at**	11.32	7.2	1.49	0.66	17.35	0.0032	ADH1B	alcohol dehydrogenase 1B (class I), beta polypeptide
**235795_at**	7.43	3.36	2.11	0.47	16.72	0.0356	PAX6	paired box 6
**202203_s_at**	8.62	4.97	0.54	0.23	12.57	7.57E-06	AMFR	autocrine motility factor receptor, E3 ubiquitin protein ligase
**204324_s_at**	7.69	4.16	0.73	0.69	11.55	0.0008	GOLIM4	Golgi integral membrane protein 4
**209612_s_at**	11.47	7.95	1.34	0.55	11.48	0.0037	ADH1B	alcohol dehydrogenase 1B (class I), beta polypeptide
**1557181_s_at**	7.11	3.61	0.27	0.36	11.32	1.27E-06	C11orf87	chromosome 11 open reading frame 87
**207145_at**	6.38	2.91	1.76	0.24	11.01	0.0046	MSTN	myostatin
**1556579_s_at**	9.27	5.98	1.07	0.64	9.81	0.002	IGSF10	immunoglobulin superfamily, member 10
**205830_at**	11.03	7.85	1.02	0.96	9.09	0.0041	CLGN	calmegin
**221796_at**	6.88	3.73	2.37	0.4	8.9	0.0362	NTRK2	neurotrophic tyrosine kinase, receptor, type 2
**214156_at**	6.78	3.64	0.48	0.27	8.79	1.14E-05	MYRIP	myosin VIIA and Rab interacting protein
**205646_s_at**	8.49	5.46	1.67	0.5	8.16	0.0342	PAX6	paired box 6
**203440_at**	9.15	6.22	0.16	1.53	7.61	0.0207	CDH2	cadherin 2, type 1, N-cadherin (neuronal)
**206346_at**	6.74	3.81	0.92	0.34	7.58	0.0005	PRLR	prolactin receptor
**239439_at**	7.24	4.34	1.53	0.19	7.46	0.0262	AFF4	AF4/FMR2 family, member 4
**203184_at**	8.64	5.75	0.96	1.81	7.38	0.0454	FBN2	fibrillin 2
**228218_at**	7.55	4.68	0.45	0.74	7.35	0.0002	LSAMP	limbic system-associated membrane protein
**228108_at**	6.97	4.14	0.92	0.59	7.08	0.0066	PPM1L	protein phosphatase, Mg2+/Mn2+ dependent, 1L
**203788_s_at**	10.49	7.71	1.16	0.47	6.87	0.009	SEMA3C	sema domain, immunoglobulin domain (Ig), short basic domain, secreted, (semaphorin) 3C
**236129_at**	9.1	6.35	0.77	1.17	6.73	0.01	GALNT5	polypeptide N-acetylgalactosaminyltransferase 5
**227629_at**	7.64	4.89	0.77	1.06	6.72	0.0019	PRLR	prolactin receptor
**239572_at**	7.8	5.06	0.66	0.35	6.66	9.18E-05	GJA3	gap junction protein alpha 3
**206693_at**	7.72	5	0.2	0.72	6.59	0.0002	IL7	interleukin 7
**202016_at**	9.93	7.22	0.52	0.95	6.5	0.0005	MEST	mesoderm specific transcript

**228088_at**	6.96	4.26	0.5	1.04	6.5	0.0088	SESTD1	SEC14 and spectrin domains 1
**203820_s_at**	9.05	6.37	0.81	0.69	6.43	0.0032	IGF2BP3	insulin-like growth factor 2 mRNA binding protein 3
**219304_s_at**	9.98	7.31	1.03	1.33	6.35	0.0299	PDGFD	platelet derived growth factor D
**209614_at**	8.26	5.6	1.62	0.26	6.33	0.0156	ADH1B	alcohol dehydrogenase 1B (class I), beta polypeptide
**231981_at**	7.61	4.97	0.9	0.45	6.2	0.0003	PRLR	prolactin receptor
**212865_s_at**	8.34	5.79	0.65	1.25	5.86	0.0221	COL14A1	collagen, type XIV, alpha 1
**211917_s_at**	6.41	3.87	0.84	0.19	5.81	0.0005	PRLR	prolactin receptor
**236277_at**	5.9	3.37	0.9	0.33	5.76	0.0014	LOC104968398	adriamycin resistance-associated
**232110_at**	6.92	4.4	0.98	0.27	5.76	0.0047	GALNT5	polypeptide N-acetylgalactosaminyltransferase 5
**1558111_at**	6.57	4.06	1.38	0.2	5.71	0.0364	MBNL1	muscleblind like splicing regulator 1
**230720_at**	6.7	4.24	0.6	1.27	5.49	0.0157	RNF182	ring finger protein 182
**1554411_at**	7.69	5.25	1.82	0.27	5.45	0.0342	CTNNB1	catenin (cadherin-associated protein), beta 1
**222860_s_at**	8.34	5.92	1	0.84	5.36	0.027	PDGFD	platelet derived growth factor D
**202350_s_at**	9.77	7.35	0.51	0.19	5.36	0.0001	MATN2	matrilin 2
**206638_at**	7.17	4.75	0.8	0.16	5.32	0.0009	HTR2B	5-hydroxytryptamine (serotonin) receptor 2B, G protein-coupled
**228708_at**	9.55	7.15	1.16	0.6	5.29	0.0058	RAB27B	RAB27B, member RAS oncogene family
**236532_at**	6.43	4.05	0.59	0.1	5.19	4.63E-05	C11orf87	chromosome 11 open reading frame 87
**213446_s_at**	9.17	6.86	1.38	0.18	4.97	0.0478	IQGAP1	IQ motif containing GTPase activating protein 1
**218899_s_at**	8.37	6.09	0.15	0.53	4.86	0.0001	BAALC	brain and acute leukemia, cytoplasmic
**216638_s_at**	6.37	4.11	1.01	0.26	4.79	0.0032	PRLR	prolactin receptor
**215303_at**	6.78	4.54	0.34	1.09	4.75	0.0182	DCLK1	doublecortin-like kinase 1
**227803_at**	7.95	5.73	0.27	0.82	4.67	0.0011	ENPP5	ectonucleotide pyrophosphatase/phosphodiesterase 5 (putative)
**205200_at**	11.06	8.87	0.49	0.55	4.58	0.0004	CLEC3B; EXOSC7	C-type lectin domain family 3, member B; exosome component 7
**211668_s_at**	9.6	7.42	0.07	0.77	4.52	0.0007	PLAU	plasminogen activator, urokinase
**1557180_at**	5.18	3.01	0.86	0.18	4.5	0.0014	C11orf87	chromosome 11 open reading frame 87
**238320_at**	8.7	6.56	1.11	0.5	4.4	0.0128	NEAT1	nuclear paraspeckle assembly transcript 1 (non-protein coding)

**238429_at**	6.94	4.81	1.07	0.18	4.39	0.0033	TMEM71	transmembrane protein 71
**243487_at**	6.07	3.95	1.15	0.08	4.35	0.0295	AFF4	AF4/FMR2 family, member 4
**230482_at**	7.73	5.62	0.07	0.36	4.32	1.31E-05	ST6GALNAC5	ST6 (alpha-N-acetyl-neuraminyl-2,3-beta-galactosyl-1,3)-N-acetylgalactosaminide alpha-2,6-sialyltransferase 5
**215933_s_at**	5.92	3.82	1.15	0.14	4.29	0.0321	HHEX	hematopoietically expressed homeobox
**205818_at**	9.59	7.5	0.91	0.87	4.26	0.0397	BRINP1	bone morphogenetic protein/retinoic acid inducible neural-specific 1
**214895_s_at**	10.06	7.98	0.68	0.14	4.25	0.0015	ADAM10	ADAM metallopeptidase domain 10
**208609_s_at**	9.81	7.73	0.96	0.48	4.22	0.0055	TNXB	tenascin XB
**222780_s_at**	7.83	5.76	0.25	0.9	4.21	0.0023	BAALC	brain and acute leukemia, cytoplasmic
**226864_at**	5.86	3.79	0.84	0.48	4.21	0.0065	PKIA	protein kinase (cAMP-dependent, catalytic) inhibitor alpha
**213451_x_at**	10.29	8.24	0.85	0.74	4.14	0.0066	TNXA; TNXB	tenascin XA (pseudogene); tenascin XB
**204612_at**	7.56	5.51	0.78	0.73	4.13	0.0044	PKIA	protein kinase (cAMP-dependent, catalytic) inhibitor alpha
**225119_at**	8.81	6.76	0.51	1.15	4.13	0.0219	CHMP4B	charged multivesicular body protein 4B
**235852_at**	6.32	4.28	0.35	0.53	4.1	0.0008	STON2	stonin 2
**216339_s_at**	7.64	5.61	1.31	0.09	4.1	0.0157	TNXA; TNXB	tenascin XA (pseudogene); tenascin XB
**210155_at**	7.47	5.45	0.82	0.26	4.05	0.0013	MYOC	myocilin, trabecular meshwork inducible glucocorticoid response
**216333_x_at**	10.33	8.31	0.79	0.73	4.03	0.0058	TNXA; TNXB	tenascin XA (pseudogene); tenascin XB
**202921_s_at**	8.61	6.62	1.14	0.25	3.98	0.0454	ANK2	ankyrin 2, neuronal
**205732_s_at**	6.44	4.45	1.28	0.24	3.96	0.0415	NCOA2	nuclear receptor coactivator 2
**206093_x_at**	10.34	8.35	0.85	0.77	3.96	0.0083	TNXA; TNXB	tenascin XA (pseudogene); tenascin XB
**204160_s_at**	8.56	6.58	0.43	0.2	3.95	0.0001	ENPP4	ectonucleotide pyrophosphatase/phosphodiesterase 4 (putative)
**242474_s_at**	5.1	3.13	1.1	0.2	3.92	0.0394	VMA21	VMA21 vacuolar H+-ATPase homolog (S. cerevisiae)
**203819_s_at**	7.32	5.36	0.78	0.65	3.9	0.0022	IGF2BP3	insulin-like growth factor 2 mRNA binding protein 3
**229057_at**	5.01	3.05	0.32	0.58	3.9	0.0021	SCN2A	sodium channel, voltage gated, type II alpha subunit

**218273_s_at**	8.21	6.27	0.69	0.64	3.84	0.0145	PDP1	pyruvate dehyrogenase phosphatase catalytic subunit 1
**210286_s_at**	6.2	4.26	0.98	0.22	3.83	0.0077	SLC4A7	solute carrier family 4, sodium bicarbonate cotransporter, member 7
**242137_at**	6.84	4.91	1.06	0.34	3.82	0.0384	RBMS3	RNA binding motif, single stranded interacting protein 3
**205399_at**	9.67	7.74	0.21	1.03	3.82	0.028	DCLK1	doublecortin-like kinase 1
**215220_s_at**	6.69	4.76	1.12	0.41	3.81	0.0478	TPR	translocated promoter region, nuclear basket protein
**205352_at**	7.73	5.81	0.4	0.96	3.79	0.0113	SERPINI1	serpin peptidase inhibitor, clade I (neuroserpin), member 1
**219195_at**	7.06	5.15	0.68	0.63	3.77	0.0187	PPARGC1 A	peroxisome proliferator-activated receptor gamma, coactivator 1 alpha
**229555_at**	9.7	7.8	0.62	0.67	3.73	0.0185	GALNT5	polypeptide N-acetylgalactosaminyltransfera se 5
**227350_at**	6.31	4.42	0.87	0.96	3.71	0.0354	HELLS	helicase, lymphoid-specific
**210942_s_at**	6.76	4.87	0.24	0.55	3.71	0.0005	ST3GAL6	ST3 beta-galactoside alpha-2,3-sialyltransferase 6
**226931_at**	9.77	7.88	0.78	0.63	3.7	0.0241	TMTC1	transmembrane and tetratricopeptide repeat containing 1
**212831_at**	6.39	4.5	0.87	0.21	3.7	0.014	MEGF9	multiple EGF-like-domains 9
**229800_at**	10.83	8.95	0.34	0.8	3.69	0.0195	DCLK1	doublecortin-like kinase 1
**205372_at**	7.13	5.25	0.44	0.19	3.68	0.0002	PLAG1	pleiomorphic adenoma gene 1
**229552_at**	7.75	5.88	0.1	0.97	3.66	0.0103	HRK; LOC283454	harakiri, BCL2 interacting protein; uncharacterized LOC283454
**212257_s_at**	9.29	7.42	0.88	0.44	3.66	0.039	SMARCA2	SWI/SNF related, matrix associated, actin dependent regulator of chromatin, subfamily a, member 2
**205479_s_at**	10.23	8.36	0.21	0.69	3.65	0.0012	PLAU	plasminogen activator, urokinase
**230962_at**	6.84	4.99	0.33	0.65	3.6	0.0035	DCLK1	doublecortin-like kinase 1
**230867_at**	5.72	3.88	1.41	0.49	3.58	0.0085	COL6A6	collagen, type VI, alpha 6
**230670_at**	9.82	7.99	1.09	0.25	3.57	0.0099	IGSF10	immunoglobulin superfamily, member 10
**237187_at**	7.02	5.19	0.16	0.76	3.57	0.0053	HRK	harakiri, BCL2 interacting protein
**217504_at**	8.4	6.58	1.03	0.6	3.53	0.0243	ABCA6	ATP binding cassette subfamily A member 6
**242458_at**	7.38	5.56	0.42	0.13	3.52	0.0002	RALGPS2	Ral GEF with PH domain and SH3 binding motif 2
**205893_at**	5.48	3.66	0.68	0.28	3.51	0.0024	NLGN1	neuroligin 1

**200672_x_at**	9.21	7.4	1.14	0.32	3.5	0.0194	SPTBN1	spectrin, beta, non-erythrocytic 1
**210073_at**	7.25	5.46	0.9	1.14	3.47	0.0265	ST8SIA1	ST8 alpha-N-acetyl-neuraminide alpha-2,8-sialyltransferase 1
**230494_at**	6.69	4.9	0.45	0.94	3.45	0.0173	SLC20A1	solute carrier family 20 (phosphate transporter), member 1
**209663_s_at**	7.49	5.71	0.7	0.43	3.43	0.0215	ITGA7	integrin alpha 7
**206376_at**	7.59	5.81	0.52	0.79	3.42	0.0214	SLC6A15	solute carrier family 6 (neutral amino acid transporter), member 15
**230744_at**	7.5	5.73	0.33	0.74	3.41	0.0038	FSTL1	follistatin like 1
**235318_at**	9.57	7.8	0.67	0.41	3.41	0.0084	FBN1	fibrillin 1
**220979_s_at**	7.73	5.96	0.3	0.63	3.41	0.0004	ST6GALNAC5	ST6 (alpha-N-acetyl-neuraminyl-2,3-beta-galactosyl-1,3)-N-acetylgalactosaminide alpha-2,6-sialyltransferase 5
**204161_s_at**	6.94	5.17	0.43	0.32	3.4	0.0004	ENPP4	ectonucleotide pyrophosphatase/phosphodiesterase 4 (putative)
**222717_at**	6.27	4.5	0.84	0.87	3.4	0.0157	SDPR	serum deprivation response
**201299_s_at**	8.79	7.05	1.03	0.2	3.34	0.047	MOB1A	MOB kinase activator 1A
**232636_at**	6.34	4.6	1.03	0.61	3.34	0.017	SLITRK4	SLIT and NTRK-like family, member 4
**1554029_a_at**	7.42	5.68	0.89	0.53	3.34	0.0493	TTC37	tetratricopeptide repeat domain 37
**229943_at**	8.66	6.93	0.34	1.24	3.34	0.027	TRIM13	tripartite motif containing 13
**220945_x_at**	9.3	7.56	0.29	0.59	3.33	0.0013	MANSC1	MANSC domain containing 1
**212650_at**	8.11	6.37	0.83	0.28	3.32	0.032	EHBP1	EH domain binding protein 1
**242336_at**	6.12	4.39	1.03	0.61	3.32	0.0323	GSK3B	glycogen synthase kinase 3 beta
**217966_s_at**	8.25	6.53	0.32	1.28	3.29	0.0355	FAM129A	family with sequence similarity 129, member A
**228457_at**	6.97	5.25	0.79	0.25	3.29	0.0089	PPM1L	protein phosphatase, Mg2+/Mn2+ dependent, 1L
**236356_at**	6.24	4.52	0.44	0.3	3.28	0.0018	NDUFS1	NADH dehydrogenase (ubiquinone) Fe-S protein 1, 75kDa (NADH-coenzyme Q reductase)
**221590_s_at**	6.32	4.61	0.68	0.64	3.26	0.0212	ALDH6A1	aldehyde dehydrogenase 6 family, member A1
**207505_at**	8.86	7.15	0.66	0.22	3.26	0.0088	PRKG2	protein kinase, cGMP-dependent, type II
**211958_at**	8.61	6.91	1.16	0.69	3.25	0.0265	IGFBP5	insulin like growth factor binding protein 5

**238868_at**	6.11	4.41	0.15	1.04	3.25	0.0274	UACA	uveal autoantigen with coiled-coil domains and ankyrin repeats
**229506_at**	6.24	4.55	0.71	0.23	3.24	0.0068	PPM1L	protein phosphatase, Mg2+/Mn2+ dependent, 1L
**222837_s_at**	5.88	4.19	0.81	0.21	3.24	0.0163	NAA15	N(alpha)-acetyltransferase 15, NatA auxiliary subunit
**1558647_at**	8.03	6.34	0.91	0.11	3.23	0.0281	SH3D19	SH3 domain containing 19
**203000_at**	12.65	10.96	0.59	0.51	3.23	0.0043	STMN2	stathmin 2
**241869_at**	6.72	5.04	0.63	0.26	3.21	0.0029	APOL6	apolipoprotein L, 6
**235740_at**	5.53	3.85	0.77	0.34	3.2	0.0125	MCTP1	multiple C2 domains, transmembrane 1
**1558882_at**	4.74	3.06	0.65	0.2	3.2	0.0011	HTATSF1P2	HIV-1 Tat specific factor 1 pseudogene 2
**228749_at**	6.59	4.91	0.88	0.24	3.2	0.0378	ZDBF2	zinc finger, DBF-type containing 2
**231726_at**	6.23	4.57	0.73	0.38	3.17	0.0037	PCDHB14	protocadherin beta 14
**229352_at**	7.24	5.59	0.53	0.09	3.15	0.0015	SPESP1	sperm equatorial segment protein 1
**206170_at**	6.95	5.3	0.79	0.42	3.14	0.022	ADRB2	adrenoceptor beta 2, surface
**219778_at**	6.36	4.71	0.55	0.71	3.13	0.0394	ZFPM2	zinc finger protein, FOG family member 2
**202604_x_at**	9.55	7.91	0.49	0.2	3.12	0.0012	ADAM10	ADAM metallopeptidase domain 10
**231361_at**	5.58	3.94	0.7	0.38	3.12	0.0103	NLGN1	neuroligin 1
**217974_at**	7.12	5.49	0.26	0.65	3.1	0.0046	TM7SF3	transmembrane 7 superfamily member 3
**204642_at**	7.61	5.98	0.57	0.31	3.09	0.0014	S1PR1	sphingosine-1-phosphate receptor 1
**222734_at**	8.62	6.99	0.26	0.33	3.09	0.0002	WARS2	tryptophanyl tRNA synthetase 2, mitochondrial
**235333_at**	7.16	5.54	0.24	0.61	3.08	0.0037	B4GALT6	UDP-Gal:betaGlcNAc beta 1,4- galactosyltransferase, polypeptide 6
**209074_s_at**	6.37	4.75	1.41	0.06	3.08	0.0424	FAM107A	family with sequence similarity 107, member A
**202975_s_at**	9.98	8.36	0.6	0.68	3.08	0.0378	RHOBTB3	Rho-related BTB domain containing 3
**222846_at**	9.4	7.78	0.72	0.51	3.07	0.0345	RAB8B	RAB8B, member RAS oncogene family
**226478_at**	6.97	5.36	0.14	0.52	3.06	0.0012	TM7SF3	transmembrane 7 superfamily member 3
**212992_at**	11.62	10.01	0.48	0.85	3.05	0.0097	AHNAK2	AHNAK nucleoprotein 2
**213355_at**	6.58	4.97	0.23	0.52	3.05	0.0009	ST3GAL6	ST3 beta-galactoside alpha-2,3-sialyltransferase 6
**212446_s_at**	7.31	5.71	0.03	0.32	3.04	4.43E-05	CERS6	ceramide synthase 6
**237054_at**	6.31	4.71	0.19	0.37	3.03	0.0001	ENPP5	ectonucleotide pyrophosphatase/phosphodiesterase 5 (putative)

**235463_s_at**	5.72	4.12	0.33	0.72	3.03	0.0152	CERS6	ceramide synthase 6
**214877_at**	7.33	5.74	0.53	0.17	3.03	0.0019	CDKAL1	CDK5 regulatory subunit associated protein 1-like 1
**244190_at**	4.35	2.76	0.58	0.29	3.01	0.0068	THAP5	THAP domain containing 5
**200727_s_at**	8.91	7.33	0.97	0.83	3.01	0.0415	ACTR2	ARP2 actin-related protein 2 homolog (yeast)
**1555609_a_at**	8.63	7.04	0.46	0.37	3	0.0023	ZMAT3	zinc finger, matrin-type 3
**232112_at**	6.98	5.4	0.3	0.18	3	0.0001	RALGPS2	Ral GEF with PH domain and SH3 binding motif 2
**212385_at**	9.46	7.89	0.95	0.6	2.98	0.0494	TCF4	transcription factor 4
**238673_at**	7.56	5.99	0.49	0.45	2.97	0.0017	SAMD12	sterile alpha motif domain containing 12
**203765_at**	7.06	5.49	0.23	0.53	2.97	0.0034	GCA	grancalcin, EF-hand calcium binding protein
**1558015_s_at**	8.71	7.14	0.79	0.39	2.96	0.0291	ACTR2	ARP2 actin-related protein 2 homolog (yeast)
**210544_s_at**	9.69	8.14	0.7	0.56	2.93	0.0376	ALDH3A2	aldehyde dehydrogenase 3 family, member A2
**227059_at**	9.69	8.14	0.46	0.55	2.93	0.0022	GPC6	glypican 6
**208442_s_at**	7.82	6.28	0.68	0.46	2.92	0.0186	ATM	ATM serine/threonine kinase
**222771_s_at**	5.53	3.99	0.66	0.53	2.91	0.0315	MYEF2	myelin expression factor 2
**206233_at**	7.16	5.63	0.17	0.21	2.9	2.69E-05	B4GALT6	UDP-Gal:betaGlcNAc beta 1,4- galactosyltransferase, polypeptide 6
**237183_at**	6.18	4.65	0.86	0.08	2.89	0.031	GALNT5	polypeptide N-acetylgalactosaminyltransferase 5
**203001_s_at**	13.12	11.59	0.71	0.54	2.89	0.0112	STMN2	stathmin 2
**212105_s_at**	7.54	6.01	0.82	0.32	2.88	0.036	DHX9	DEAH (Asp-Glu-Ala-His) box helicase 9
**212239_at**	9.23	7.71	0.49	1.01	2.87	0.0243	PIK3R1	phosphoinositide-3-kinase, regulatory subunit 1 (alpha)
**204689_at**	5	3.48	0.82	0.07	2.86	0.0193	HHEX	hematopoietically expressed homeobox
**227941_at**	8.04	6.53	0.12	0.48	2.85	0.0018	LOC339803	uncharacterized LOC339803
**225408_at**	8.16	6.65	0.59	0.28	2.85	0.0025	MBP	myelin basic protein
**1553685_s_at**	7.21	5.7	0.79	0.52	2.84	0.0474	SP1	Sp1 transcription factor
**207604_s_at**	7.17	5.67	0.59	0.65	2.83	0.0359	SLC4A7	solute carrier family 4, sodium bicarbonate cotransporter, member 7
**221618_s_at**	7.33	5.83	0.03	0.65	2.82	0.0031	TAF9B	TAF9B RNA polymerase II, TATA box binding protein (TBP)-associated factor, 31kDa
**238756_at**	8.04	6.54	0.62	0.3	2.82	0.0031	GAS2L3	growth arrest-specific 2 like 3
**207724_s_at**	6.4	4.91	0.45	0.15	2.8	0.0012	SPAST	spastin
**242107_x_at**	5.27	3.8	1.01	0.32	2.78	0.048	TBX18	T-box 18

**227533_at**	9.06	7.59	0.14	0.25	2.78	6.33E-05	RALGPS2	Ral GEF with PH domain and SH3 binding motif 2
**204291_at**	6.12	4.64	0.52	0.35	2.77	0.0023	ZNF518A	zinc finger protein 518A
**221765_at**	8.62	7.15	0.52	0.48	2.77	0.0057	UGCG	UDP-glucose ceramide glucosyltransferase
**239352_at**	5.79	4.32	0.62	0.48	2.76	0.0162	SLC6A15	solute carrier family 6 (neutral amino acid transporter), member 15
**222438_at**	7.46	6	0.36	0.23	2.75	0.0007	MED4	mediator complex subunit 4
**220327_at**	10.76	9.31	0.37	0.3	2.74	0.0008	VGLL3	vestigial-like family member 3
**203789_s_at**	12.4	10.96	0.52	0.42	2.72	0.0127	SEMA3C	sema domain, immunoglobulin domain (Ig), short basic domain, secreted, (semaphorin) 3C
**227529_s_at**	9.12	7.68	0.7	0.42	2.72	0.0362	AKAP12	A kinase (PRKA) anchor protein 12
**228045_at**	6.51	5.06	0.17	0.54	2.72	0.0015	SUGT1	SGT1 homolog, MIS12 kinetochore complex assembly cochaperone
**218885_s_at**	8.71	7.27	0.06	0.81	2.71	0.0386	GALNT12	polypeptide N-acetylgalactosaminyltransfera se 12
**201939_at**	9.95	8.51	0.62	0.59	2.71	0.0195	PLK2	polo-like kinase 2
**87100_at**	5.61	4.17	0.57	0.28	2.7	0.0168	ABHD2	abhydrolase domain containing 2
**206614_at**	9.55	8.12	0.44	0.32	2.7	0.0006	GDF5	growth differentiation factor 5
**228654_at**	7.44	6.01	0.31	0.24	2.69	0.0003	SPIN4	spindlin family, member 4
**205112_at**	5.54	4.12	0.55	0.42	2.69	0.0121	PLCE1	phospholipase C, epsilon 1
**213469_at**	6.82	5.4	0.28	0.48	2.68	0.0034	PGAP1	post-GPI attachment to proteins 1
**239919_at**	8.73	7.31	0.4	0.42	2.67	0.0054	TBX5-AS1	TBX5 antisense RNA 1
**241669_x_at**	6.26	4.84	0.69	0.26	2.67	0.0407	PRKD2	protein kinase D2
**226269_at**	5.59	4.18	0.24	0.82	2.66	0.022	GDAP1	ganglioside induced differentiation associated protein 1
**221584_s_at**	10.22	8.81	0.52	0.79	2.66	0.0126	KCNMA1	potassium channel, calcium activated large conductance subfamily M alpha, member 1
**219371_s_at**	8.89	7.48	0.85	0.13	2.65	0.0157	KLF2	Kruppel-like factor 2
**235244_at**	6.91	5.5	0.47	0.4	2.65	0.0022	CCDC58	coiled-coil domain containing 58
**232431_at**	6.1	4.7	0.42	0.73	2.64	0.0088	NR3C1	nuclear receptor subfamily 3, group C, member 1 (glucocorticoid receptor)
**213022_s_at**	8.14	6.75	0.67	0.25	2.63	0.0372	UTRN	utrophin
**227752_at**	5.85	4.46	0.2	0.24	2.63	0.0003	SPTLC3	serine palmitoyltransferase, long chain base subunit 3

**205234_at**	8.33	6.94	0.3	0.61	2.62	0.001	SLC16A4	solute carrier family 16, member 4
**232825_s_at**	9.08	7.69	0.41	0.25	2.62	0.0011	DSEL	dermatan sulfate epimerase-like
**235088_at**	8.95	7.56	0.31	0.08	2.61	0.0002	C4orf46	chromosome 4 open reading frame 46
**232263_at**	5.92	4.54	0.13	0.75	2.61	0.0424	SLC6A15	solute carrier family 6 (neutral amino acid transporter), member 15
**228494_at**	4.73	3.34	0.6	0.13	2.61	0.0032	PPP1R9A	protein phosphatase 1, regulatory subunit 9A
**227041_at**	7.83	6.45	0.35	0.52	2.6	0.0056	SESTD1	SEC14 and spectrin domains 1
**223730_at**	7.73	6.35	0.56	0.34	2.6	0.0077	GPC6	glypican 6
**238909_at**	7.36	5.98	0.29	0.83	2.6	0.0132	S100A10	S100 calcium binding protein A10
**202820_at**	9.7	8.33	0.29	0.23	2.59	0.0003	AHR	aryl hydrocarbon receptor
**232535_at**	4.75	3.38	0.53	0.15	2.59	0.0038	RSBN1L	round spermatid basic protein 1-like
**210305_at**	9.08	7.71	0.73	0.56	2.59	0.0147	PDE4DIP	phosphodiesterase 4D interacting protein
**204749_at**	8.26	6.89	0.2	1.35	2.58	0.0248	NAP1L3	nucleosome assembly protein 1-like 3
**212229_s_at**	9.3	7.93	0.17	0.77	2.58	0.0389	FBXO21	F-box protein 21
**235570_at**	9.58	8.21	0.38	0.38	2.57	0.0021	RBMS3	RNA binding motif, single stranded interacting protein 3
**212442_s_at**	8.42	7.05	0.15	0.4	2.57	0.0008	CERS6	ceramide synthase 6
**215646_s_at**	11.61	10.26	0.59	0.35	2.56	0.0169	VCAN	versican
**238081_at**	7.31	5.95	0.25	0.49	2.56	0.0031	WDFY3-AS2	WDFY3 antisense RNA 2
**244779_at**	7.56	6.21	0.55	0.66	2.55	0.0061	ZDHHC2	zinc finger, DHHC-type containing 2
**212761_at**	11.22	9.87	0.48	0.42	2.55	0.01	TCF7L2	transcription factor 7-like 2 (T-cell specific, HMG-box)
**227040_at**	8.7	7.35	0.4	0.44	2.55	0.0126	NHLRC3	NHL repeat containing 3
**1554547_at**	5.92	4.58	0.55	0.42	2.54	0.0136	FAM13C	family with sequence similarity 13, member C
**229656_s_at**	5.62	4.28	0.91	0.09	2.53	0.0037	EML6	echinoderm microtubule associated protein like 6
**212801_at**	9.01	7.67	0.26	0.49	2.53	0.0038	CIT	citron rho-interacting serine/threonine kinase
**212806_at**	8.55	7.22	0.52	0.71	2.52	0.0105	PRUNE2	prune homolog 2 (Drosophila)
**204646_at**	10.2	8.87	0.54	0.46	2.52	0.0356	DPYD	dihydropyrimidine dehydrogenase
**221886_at**	7.09	5.75	0.29	0.57	2.52	0.007	DENND2A	DENN/MADD domain containing 2A
**212830_at**	9.43	8.1	0.15	0.33	2.51	0.0004	MEGF9	multiple EGF-like-domains 9
**221218_s_at**	7.29	5.97	0.24	0.45	2.51	0.0017	TPK1	thiamin pyrophosphokinase 1

**222036_s_at**	7.89	6.57	0.65	0.51	2.49	0.0272	MCM4	minichromosome maintenance complex component 4
**201222_s_at**	12.21	10.89	0.33	0.5	2.48	0.0109	RAD23B	RAD23 homolog B, nucleotide excision repair protein
**210543_s_at**	8.74	7.43	0.56	0.13	2.47	0.0133	PRKDC	protein kinase, DNA-activated, catalytic polypeptide
**210858_x_at**	8.59	7.28	0.57	0.15	2.47	0.0075	ATM	ATM serine/threonine kinase
**229498_at**	7.67	6.36	0.82	0.67	2.47	0.0244	MBNL3	muscleblind-like splicing regulator 3
**239761_at**	8.38	7.08	0.17	0.4	2.47	0.0003	GCNT1	glucosaminyl (N-acetyl) transferase 1, core 2
**209708_at**	10.74	9.44	0.77	0.5	2.46	0.0479	MOXD1	monooxygenase, DBH-like 1
**229715_at**	7.07	5.77	0.32	0.4	2.46	0.0102	NCR3LG1	natural killer cell cytotoxicity receptor 3 ligand 1
**228573_at**	9.7	8.4	0.52	0.14	2.46	0.0025	ANTXR2	anthrax toxin receptor 2
**226278_at**	7.85	6.56	0.25	0.67	2.45	0.0178	SVIP	small VCP/p97-interacting protein
**205091_x_at**	9.27	7.98	0.53	0.41	2.45	0.0184	RECQL	RecQ helicase-like
**226873_at**	9.39	8.09	0.32	0.28	2.45	0.0009	FAM63B	family with sequence similarity 63, member B
**225186_at**	7.76	6.47	0.47	0.55	2.45	0.0115	RAPH1	Ras association (RalGDS/AF-6) and pleckstrin homology domains 1
**243141_at**	8.07	6.78	0.62	0.17	2.45	0.029	SGMS2	sphingomyelin synthase 2
**214321_at**	9.97	8.68	0.34	0.59	2.45	0.007	NOV	nephroblastoma overexpressed
**207480_s_at**	10.08	8.79	0.34	0.08	2.44	0.0008	MEIS2	Meis homeobox 2
**220342_x_at**	8.03	6.75	0.54	0.43	2.44	0.021	EDEM3	ER degradation enhancer, mannosidase alpha-like 3
**225768_at**	9.86	8.57	0.5	0.33	2.44	0.0118	NR1D2	nuclear receptor subfamily 1, group D, member 2
**218674_at**	6.86	5.58	0.47	0.23	2.44	0.0046	TRAPPC13	trafficking protein particle complex 13
**205003_at**	8.66	7.38	0.6	0.61	2.44	0.0424	DOCK4	dedicator of cytokinesis 4
**235144_at**	7.54	6.25	0.43	0.38	2.44	0.003	RASEF	RAS and EF-hand domain containing
**235494_at**	5.89	4.61	0.49	0.19	2.43	0.0012	LSAMP	limbic system-associated membrane protein
**204666_s_at**	6	4.72	0.63	0.1	2.43	0.0179	SIKE1	suppressor of IKBKE 1
**209529_at**	7.12	5.85	0.55	0.86	2.43	0.0281	PLPP2	phospholipid phosphatase 2
**201733_at**	6.84	5.57	0.74	0.03	2.42	0.045	CLCN3	chloride channel, voltage-sensitive 3
**243755_at**	5.48	4.2	0.77	0.43	2.42	0.0076	PRLR	prolactin receptor
**238050_at**	6.27	5	0.52	0.55	2.41	0.0087	ANTXR2	anthrax toxin receptor 2
**229584_at**	6.67	5.4	0.64	0.41	2.41	0.0086	LRRK2	leucine-rich repeat kinase 2

**215210_s_at**	9.38	8.12	0.41	0.65	2.4	0.0145	DLST	dihydrolipoamide S-succinyltransferase (E2 component of 2-oxo-glutarate complex)
**1557174_a_at**	5.3	4.04	0.25	0.58	2.4	0.0173	IRAK1BP1	interleukin 1 receptor associated kinase 1 binding protein 1
**212762_s_at**	8.32	7.06	0.5	0.78	2.4	0.0248	TCF7L2	transcription factor 7-like 2 (T-cell specific, HMG-box)
**202202_s_at**	11.36	10.1	0.45	0.12	2.39	0.0044	LAMA4	laminin, alpha 4
**213802_at**	8.47	7.21	0.69	0.15	2.39	0.0111	PRSS12	protease, serine, 12 (neurotrypsin, motopsin)
**204237_at**	8.84	7.59	0.5	0.17	2.39	0.0064	GULP1	GULP, engulfment adaptor PTB domain containing 1
**235831_at**	4.94	3.69	0.42	0.3	2.38	0.0013	ZNF385D	zinc finger protein 385D
**241801_at**	4.88	3.63	0.27	0.34	2.38	0.0022	PGAP1	post-GPI attachment to proteins 1
**212901_s_at**	7.49	6.24	0.63	0.28	2.38	0.021	CSTF2T	cleavage stimulation factor, 3' pre-RNA, subunit 2, tau variant
**201008_s_at**	10.88	9.62	0.52	0.32	2.38	0.0147	TXNIP	thioredoxin interacting protein
**202766_s_at**	12.44	11.19	0.46	0.35	2.38	0.0257	FBN1	fibrillin 1
**227448_at**	5.79	4.54	0.14	0.45	2.38	0.0039	ARGLU1	arginine and glutamate rich 1
**228837_at**	7.24	5.99	0.8	0.23	2.38	0.0288	TCF4	transcription factor 4
**220039_s_at**	5.11	3.87	0.46	0.05	2.37	0.0038	CDKAL1	CDK5 regulatory subunit associated protein 1-like 1
**210089_s_at**	8.51	7.27	0.06	0.14	2.37	1.57E-05	LAMA4	laminin, alpha 4
**242422_at**	6.22	4.98	0.48	0.16	2.37	0.0061	G3BP1	GTPase activating protein (SH3 domain) binding protein 1
**226344_at**	4.64	3.4	0.46	0.14	2.37	0.0011	ZMAT1	zinc finger, matrin-type 1
**212107_s_at**	8.84	7.6	0.72	0.36	2.36	0.0295	DHX9	DEAH (Asp-Glu-Ala-His) box helicase 9
**209258_s_at**	6.96	5.72	0.49	0.36	2.36	0.0116	SMC3	structural maintenance of chromosomes 3
**233947_s_at**	8.58	7.35	0.51	0.63	2.36	0.0182	TBX5-AS1	TBX5 antisense RNA 1
**220298_s_at**	6.24	5	0.67	0.27	2.36	0.0218	SPATA6	spermatogenesis associated 6
**214691_x_at**	8.21	6.97	0.22	0.43	2.36	0.0014	FAM63B	family with sequence similarity 63, member B
**210568_s_at**	8.89	7.66	0.54	0.32	2.36	0.0171	RECQL	RecQ helicase-like
**222835_at**	7.6	6.37	0.48	0.62	2.35	0.0173	THSD4	thrombospondin type 1 domain containing 4
**214460_at**	6.95	5.72	0.54	0.61	2.35	0.0344	LSAMP	limbic system-associated membrane protein
**1554885_a_at**	6.65	5.43	0.44	0.49	2.34	0.013	PRIM2	primase, DNA, polypeptide 2 (58kDa)
**227461_at**	8.47	7.25	0.34	0.5	2.33	0.0128	STON2	stonin 2

**221922_at**	9.25	8.03	0.59	0.4	2.33	0.0081	GPSM2	G-protein signaling modulator 2
**213661_at**	11.12	9.9	0.57	0.44	2.33	0.0341	PAMR1	peptidase domain containing associated with muscle regeneration 1
**200612_s_at**	10.07	8.85	0.42	0.49	2.33	0.0088	AP2B1	adaptor-related protein complex 2, beta 1 subunit
**211571_s_at**	10.78	9.56	0.54	0.46	2.32	0.0183	VCAN	versican
**216048_s_at**	8.18	6.96	0.6	0.28	2.32	0.014	RHOBTB3	Rho-related BTB domain containing 3
**215708_s_at**	7.74	6.52	0.37	0.62	2.32	0.0213	PRIM2	primase, DNA, polypeptide 2 (58kDa)
**206767_at**	7.37	6.16	0.57	0.24	2.32	0.0372	RBMS3	RNA binding motif, single stranded interacting protein 3
**201009_s_at**	10.88	9.66	0.52	0.22	2.32	0.0122	TXNIP	thioredoxin interacting protein
**206263_at**	8.19	6.98	0.53	0.27	2.32	0.0168	FMO4	flavin containing monooxygenase 4
**231793_s_at**	8.99	7.78	0.53	0.4	2.31	0.0264	CAMK2D	calcium/calmodulin-dependent protein kinase II delta
**1558093_s_at**	9.17	7.96	0.67	0.18	2.31	0.0408	MATR3	matrin 3
**212142_at**	6.15	4.94	0.44	0.41	2.31	0.0115	MCM4	minichromosome maintenance complex component 4
**201718_s_at**	8.92	7.72	0.68	0.27	2.31	0.0115	EPB41L2	erythrocyte membrane protein band 4.1-like 2
**230194_at**	7.28	6.07	0.07	0.59	2.31	0.0035	LRPPRC	leucine-rich pentatricopeptide repeat containing
**223686_at**	6.47	5.26	0.19	0.43	2.3	0.001	TPK1	thiamin pyrophosphokinase 1
**216037_x_at**	9.11	7.91	0.48	0.43	2.3	0.0206	TCF7L2	transcription factor 7-like 2 (T-cell specific, HMG-box)
**232411_at**	5.26	4.06	0.49	0.9	2.3	0.026	KCNJ6	potassium channel, inwardly rectifying subfamily J, member 6
**207522_s_at**	7.21	6.01	0.45	0.25	2.29	0.0015	ATP2A3	ATPase, Ca++ transporting, ubiquitous
**244852_at**	8.29	7.1	0.35	0.21	2.29	0.0022	DSEL	dermatan sulfate epimerase-like
**242086_at**	6.55	5.35	0.67	0.5	2.29	0.0362	SPATA6	spermatogenesis associated 6
**219003_s_at**	7.74	6.54	0.36	0.19	2.29	0.0021	MANEA	mannosidase, endo-alpha
**1553108_at**	6.86	5.66	0.48	0.11	2.29	0.0048	C5orf24	chromosome 5 open reading frame 24
**238447_at**	9.61	8.41	0.43	0.61	2.29	0.0108	RBMS3	RNA binding motif, single stranded interacting protein 3
**222572_at**	10.45	9.25	0.27	0.38	2.29	0.0029	PDP1	pyruvate dehyrogenase phosphatase catalytic subunit 1

**235631_at**	9.69	8.5	0.58	0.13	2.28	0.0135	DDR2	discoidin domain receptor tyrosine kinase 2
**222731_at**	10.52	9.33	0.31	0.53	2.28	0.0196	ZDHHC2	zinc finger, DHHC-type containing 2
**238865_at**	5.43	4.25	0.18	0.44	2.28	0.0016	PABPC4L	poly(A) binding protein, cytoplasmic 4-like
**224486_s_at**	8.44	7.25	0.23	0.2	2.28	0.0003	C15orf41	chromosome 15 open reading frame 41
**209821_at**	5.83	4.64	0.83	0.61	2.28	0.0134	IL33	interleukin 33
**209376_x_at**	7.74	6.55	0.26	0.51	2.27	0.0307	SCAF11	SR-related CTD-associated factor 11
**235294_at**	6.84	5.66	0.54	0.19	2.27	0.0268	SIKE1	suppressor of IKBKE 1
**221841_s_at**	11.52	10.34	0.49	0.56	2.27	0.0216	KLF4	Kruppel-like factor 4 (gut)
**229430_at**	5.17	4	0.48	0.26	2.26	0.0123	C8orf46	chromosome 8 open reading frame 46
**226763_at**	8.98	7.81	0.45	0.49	2.25	0.0416	SESTD1	SEC14 and spectrin domains 1
**206865_at**	6.71	5.54	0.24	0.33	2.25	0.0024	HRK	harakiri, BCL2 interacting protein
**230492_s_at**	6.61	5.44	0.46	0.27	2.25	0.0092	GPCPD1	glycerophosphocholine phosphodiesterase 1
**214925_s_at**	5.89	4.72	0.33	0.48	2.25	0.0151	SPTAN1	spectrin, alpha, non-erythrocytic 1
**1555559_s_at**	6.82	5.65	0.63	0.29	2.25	0.04	USP25	ubiquitin specific peptidase 25
**223263_s_at**	6.92	5.75	0.63	0.24	2.25	0.0246	FGFR1OP2	FGFR1 oncogene partner 2
**214499_s_at**	8.38	7.22	0.64	0.17	2.25	0.046	BCLAF1	BCL2-associated transcription factor 1
**235051_at**	7.91	6.74	0.56	0.19	2.24	0.0375	CCDC50	coiled-coil domain containing 50
**202053_s_at**	10.64	9.47	0.43	0.28	2.24	0.0084	ALDH3A2	aldehyde dehydrogenase 3 family, member A2
**238459_x_at**	4.73	3.57	0.61	0.06	2.24	0.0207	SPATA6	spermatogenesis associated 6
**226561_at**	7.75	6.59	0.26	0.45	2.24	0.0051	AGFG1	ArfGAP with FG repeats 1
**205240_at**	9.11	7.95	0.63	0.43	2.24	0.0066	GPSM2	G-protein signaling modulator 2
**227550_at**	8.62	7.45	0.64	0.42	2.24	0.0369	GFRA1	GDNF family receptor alpha 1
**1554178_a_at**	4.21	3.05	0.13	0.64	2.23	0.0366	FAM126B	family with sequence similarity 126, member B
**242569_at**	6.54	5.38	0.45	0.24	2.23	0.0084	STAM2	signal transducing adaptor molecule (SH3 domain and ITAM motif) 2
**220299_at**	5.62	4.47	0.61	0.13	2.23	0.0087	SPATA6	spermatogenesis associated 6
**232506_s_at**	6.11	4.96	0.35	0.25	2.23	0.0022	C15orf41	chromosome 15 open reading frame 41
**225525_at**	8.12	6.97	0.18	0.31	2.22	0.0006	KIAA1671	KIAA1671

**240419_at**	5.54	4.38	0.12	0.33	2.22	0.0004	SLC6A15	solute carrier family 6 (neutral amino acid transporter), member 15
**241789_at**	7.59	6.43	0.83	0.93	2.22	0.0421	RBMS3	RNA binding motif, single stranded interacting protein 3
**221207_s_at**	7.54	6.39	0.71	0.21	2.22	0.0489	NBEA	neurobeachin
**217525_at**	10.11	8.97	0.61	0.64	2.21	0.0267	OLFML1	olfactomedin like 1
**229331_at**	10.08	8.93	0.63	0.33	2.21	0.0392	SPATA18	spermatogenesis associated 18
**228315_at**	9.88	8.73	0.56	0.58	2.21	0.0493	ZMAT3	zinc finger, matrin-type 3
**207564_x_at**	9.03	7.89	0.63	0.31	2.21	0.0248	OGT	O-linked N-acetylglucosamine (GlcNAc) transferase
**205296_at**	5.92	4.77	0.66	0.19	2.21	0.0066	RBL1	retinoblastoma-like 1
**243862_at**	4.79	3.65	0.46	0.17	2.21	0.0045	RASEF	RAS and EF-hand domain containing
**228695_at**	4.7	3.56	0.58	0.46	2.21	0.0347	C8orf46	chromosome 8 open reading frame 46
**1557370_s_at**	8.41	7.27	0.31	0.5	2.21	0.0404	MYCBP2	MYC binding protein 2, E3 ubiquitin protein ligase
**219023_at**	7.96	6.82	0.54	0.37	2.21	0.0289	AP1AR	adaptor-related protein complex 1 associated regulatory protein
**201085_s_at**	7.76	6.62	0.59	0.17	2.21	0.0488	SON	SON DNA binding protein
**230427_s_at**	6.25	5.11	0.22	0.28	2.2	0.0022	BAG5	BCL2-associated athanogene 5
**202054_s_at**	10.38	9.24	0.47	0.51	2.2	0.0395	ALDH3A2	aldehyde dehydrogenase 3 family, member A2
**205515_at**	7.71	6.57	0.77	0.03	2.2	0.0162	PRSS12	protease, serine, 12 (neurotrypsin, motopsin)
**215029_at**	4.7	3.57	0.44	0.38	2.2	0.0349	AKIRIN1	akirin 1
**217967_s_at**	10.46	9.32	0.07	0.95	2.2	0.0278	FAM129A	family with sequence similarity 129, member A
**1569348_at**	5.74	4.61	0.47	0.66	2.2	0.0195	TPTEP1	transmembrane phosphatase with tensin homology pseudogene 1
**238420_at**	7.24	6.11	0.66	0.23	2.2	0.0325	TAOK1	TAO kinase 1
**218396_at**	9.62	8.49	0.5	0.33	2.2	0.0268	VPS13C	vacuolar protein sorting 13 homolog C (S. cerevisiae)
**222037_at**	6.85	5.71	0.75	0.12	2.2	0.0318	MCM4	minichromosome maintenance complex component 4
**229657_at**	9.08	7.95	0.65	0.23	2.2	0.0302	THRB	thyroid hormone receptor, beta
**219949_at**	9.16	8.03	0.53	0.3	2.19	0.016	LRRC2	leucine rich repeat containing 2
**235698_at**	8.25	7.12	0.12	0.85	2.19	0.0376	ZFP90	ZFP90 zinc finger protein
**238020_at**	7.44	6.31	0.14	0.49	2.18	0.0053	PSMC2	proteasome 26S subunit, ATPase 2

**231929_at**	5.7	4.58	0.3	0.15	2.18	0.0012	IKZF2	IKAROS family zinc finger 2
**212577_at**	8.28	7.15	0.29	0.1	2.18	0.0008	SMCHD1	structural maintenance of chromosomes flexible hinge domain containing 1
**209060_x_at**	7.46	6.33	0.7	0.45	2.18	0.028	NCOA3	nuclear receptor coactivator 3
**213618_at**	4.07	2.95	0.74	0.33	2.18	0.0435	ARAP2	ArfGAP with RhoGAP domain, ankyrin repeat and PH domain 2
**205882_x_at**	11.5	10.38	0.31	0.42	2.18	0.0042	ADD3	adducin 3 (gamma)
**211959_at**	11.04	9.92	0.78	1.38	2.18	0.0344	IGFBP5	insulin like growth factor binding protein 5
**243031_at**	5.07	3.95	0.63	0.47	2.17	0.0212		
**241401_at**	6.4	5.28	0.4	0.4	2.17	0.0044	WDFY3-AS2	WDFY3 antisense RNA 2
**205794_s_at**	8.23	7.11	0.49	0.58	2.17	0.0183	NOVA1	neuro-oncological ventral antigen 1
**227132_at**	7.77	6.66	0.11	0.25	2.17	0.0005	ZNF706	zinc finger protein 706
**205010_at**	7.12	6.01	0.4	0.4	2.17	0.0193	GNL3L	guanine nucleotide binding protein-like 3 (nucleolar)-like
**216511_s_at**	8.92	7.8	0.51	0.45	2.17	0.0242	TCF7L2	transcription factor 7-like 2 (T-cell specific, HMG-box)
**203636_at**	9.48	8.37	0.39	0.3	2.16	0.0209	MID1	midline 1
**214647_s_at**	7.43	6.31	0.62	0.37	2.16	0.0252	HFE	hemochromatosis
**201753_s_at**	11.91	10.8	0.26	0.53	2.16	0.0062	ADD3	adducin 3 (gamma)
**220266_s_at**	8.94	7.83	0.69	0.34	2.16	0.0399	KLF4	Kruppel-like factor 4 (gut)
**227101_at**	6.45	5.34	0.59	0.17	2.16	0.039	ZNF800	zinc finger protein 800
**1552678_a_at**	7.07	5.96	0.49	0.25	2.16	0.0156	USP28	ubiquitin specific peptidase 28
**225886_at**	8.62	7.51	0.69	0.3	2.16	0.0407	DDX5	DEAD (Asp-Glu-Ala-Asp) box helicase 5
**212917_x_at**	9.83	8.72	0.42	0.34	2.15	0.0332	RECQL	RecQ helicase-like
**229442_at**	7.1	5.99	0.31	0.68	2.15	0.0177	C18orf54	chromosome 18 open reading frame 54
**225162_at**	10.78	9.68	0.55	0.21	2.15	0.0187	SH3D19	SH3 domain containing 19
**206316_s_at**	6.26	5.15	0.66	0.36	2.15	0.036	KNTC1	kinetochore associated 1
**212847_at**	5.62	4.52	0.12	0.24	2.15	0.0004	FUBP1	far upstream element (FUSE) binding protein 1
**202581_at**	8.47	7.37	0.9	0.56	2.15	0.0444	HSPA1A; HSPA1B	heat shock 70kDa protein 1A; heat shock 70kDa protein 1B
**235155_at**	6.71	5.61	0.42	0.24	2.15	0.0088	BDH2	3-hydroxybutyrate dehydrogenase, type 2
**216035_x_at**	9.1	8	0.54	0.42	2.15	0.0303	TCF7L2	transcription factor 7-like 2 (T-cell specific, HMG-box)
**203482_at**	7.43	6.33	0.16	0.25	2.14	0.0005	SLF2	SMC5-SMC6 complex localization factor 2
**220244_at**	7.55	6.45	0.71	0.23	2.14	0.021	LINC00312	long intergenic non-protein coding RNA 312

**221885_at**	7.64	6.54	0.39	0.44	2.14	0.0256	DENND2A	DENN/MADD domain containing 2A
**222414_at**	7.94	6.85	0.65	0.07	2.14	0.0426	KMT2C	lysine (K)-specific methyltransferase 2C
**201083_s_at**	8.3	7.21	0.66	0.14	2.14	0.032	BCLAF1	BCL2-associated transcription factor 1
**236922_at**	6.89	5.8	0.36	0.22	2.14	0.0049	NCK1	NCK adaptor protein 1
**225742_at**	5.7	4.61	0.48	0.4	2.14	0.0288	MDM4	MDM4, p53 regulator
**201752_s_at**	11.62	10.53	0.34	0.44	2.13	0.0056	ADD3	adducin 3 (gamma)
**241700_at**	7.15	6.06	0.41	0.33	2.13	0.0027	ZFHX4	zinc finger homeobox 4
**223614_at**	6.15	5.07	0.5	0.56	2.12	0.0318	MMP16	matrix metallopeptidase 16 (membrane-inserted)
**212579_at**	8.2	7.12	0.24	0.22	2.12	0.0009	SMCHD1	structural maintenance of chromosomes flexible hinge domain containing 1
**205540_s_at**	6.75	5.66	0.51	0.05	2.12	0.0164	RRAGB	Ras-related GTP binding B
**223842_s_at**	8.42	7.34	0.55	0.34	2.12	0.0092	SCARA3	scavenger receptor class A, member 3
**243759_at**	5.24	4.16	0.57	0.57	2.12	0.0265	SCAF4	SR-related CTD-associated factor 4
**219779_at**	7.65	6.57	0.33	0.16	2.12	0.0024	ZFHX4	zinc finger homeobox 4
**218711_s_at**	5.69	4.61	0.6	0.2	2.12	0.0112	SDPR	serum deprivation response
**232044_at**	6.71	5.63	0.53	0.27	2.12	0.0289	RBBP6	retinoblastoma binding protein 6
**221583_s_at**	5.77	4.68	0.38	0.27	2.12	0.0009	KCNMA1	potassium channel, calcium activated large conductance subfamily M alpha, member 1
**238963_at**	5.86	4.78	0.55	0.19	2.12	0.0099	RBM18	RNA binding motif protein 18
**222478_at**	8.8	7.72	0.17	0.23	2.11	0.0004	VPS36	vacuolar protein sorting 36 homolog (S. cerevisiae)
**228709_at**	5.96	4.88	0.29	0.58	2.11	0.0155	TPR	translocated promoter region, nuclear basket protein
**235956_at**	6.45	5.37	0.34	0.65	2.11	0.0183	CEP126	centrosomal protein 126kDa
**207808_s_at**	10.89	9.81	0.34	0.23	2.11	0.0036	PROS1	protein S (alpha)
**214843_s_at**	8.9	7.82	0.59	0.02	2.11	0.0372	USP33	ubiquitin specific peptidase 33
**204286_s_at**	7.21	6.14	0.82	0.77	2.11	0.0165	PMAIP1	phorbol-12-myristate-13-acetate-induced protein 1
**219355_at**	6.11	5.03	0.18	0.28	2.11	0.0003	CXorf57	chromosome X open reading frame 57
**211599_x_at**	8.09	7.01	0.08	0.65	2.1	0.0426	MET	MET proto-oncogene, receptor tyrosine kinase
**222310_at**	5.57	4.5	0.55	0.36	2.1	0.0057	SCAF4	SR-related CTD-associated factor 4
**243904_at**	7.53	6.46	0.56	0.31	2.1	0.0036	STXBP5	syntaxin binding protein 5 (tomosyn)

**218108_at**	8.78	7.71	0.09	0.18	2.1	0.0002	UBR7	ubiquitin protein ligase E3 component n-recognin 7 (putative)
**212086_x_at**	11.21	10.14	0.26	0.43	2.1	0.0129	LMNA	lamin A/C
**226114_at**	7.85	6.79	0.67	0.21	2.1	0.0313	ZNF436	zinc finger protein 436
**211828_s_at**	6.28	5.21	0.66	0.02	2.1	0.012	TNIK	TRAF2 and NCK interacting kinase
**235771_at**	5.17	4.1	0.76	0.15	2.1	0.0178	LINC00472	long intergenic non-protein coding RNA 472
**234980_at**	5.47	4.4	0.53	0.26	2.09	0.0134	TMEM56	transmembrane protein 56
**1553749_at**	7.1	6.03	0.4	0.5	2.09	0.0332	FAM76B	family with sequence similarity 76, member B
**213761_at**	6.51	5.44	0.3	0.13	2.09	0.0015	MDM1	Mdm1 nuclear protein
**241370_at**	6.91	5.85	0.28	0.54	2.09	0.0084	LOC286052 ; TMEM65	uncharacterized LOC286052; transmembrane protein 65
**230773_at**	6.56	5.5	0.52	0.35	2.08	0.013	ZNF385D	zinc finger protein 385D
**223843_at**	7.94	6.89	0.28	0.91	2.08	0.0277	SCARA3	scavenger receptor class A, member 3
**238736_at**	8.29	7.24	0.83	0.31	2.08	0.0239	REV3L	REV3 like, DNA directed polymerase zeta catalytic subunit
**213373_s_at**	7.63	6.58	0.28	0.04	2.08	0.0008	CASP8	caspase 8, apoptosis-related cysteine peptidase
**212730_at**	7.23	6.18	0.22	0.33	2.07	0.0072	SYNM	synemin, intermediate filament protein
**226071_at**	8.35	7.3	0.19	0.64	2.07	0.0054	ADAMTSL 4	ADAMTS like 4
**222834_s_at**	8.66	7.61	0.58	0.15	2.07	0.009	GNG12	guanine nucleotide binding protein (G protein), gamma 12
**235106_at**	6.73	5.68	0.56	0.44	2.07	0.0234	MAML2	mastermind-like transcriptional coactivator 2
**205083_at**	10.84	9.79	0.29	0.52	2.07	0.0413	AOX1	aldehyde oxidase 1
**211986_at**	11.41	10.36	0.51	0.44	2.06	0.0165	AHNAK	AHNAK nucleoprotein
**206271_at**	6.17	5.13	0.09	0.31	2.06	0.0008	TLR3	toll-like receptor 3
**235030_at**	6.44	5.4	0.56	0.58	2.06	0.0084	NXPE3	neurexophilin and PC-esterase domain family, member 3
**1552739_s_at**	5.76	4.72	0.07	0.4	2.06	0.0042	ST7L	suppression of tumorigenicity 7 like
**228960_at**	6.43	5.39	0.09	0.28	2.06	0.001	ICE2	interactor of little elongation complex ELL subunit 2
**227152_at**	5.58	4.54	0.61	0.18	2.06	0.0415	KIAA1551	KIAA1551
**217886_at**	8.93	7.89	0.4	0.2	2.06	0.0109	EPS15	epidermal growth factor receptor pathway substrate 15
**226713_at**	8.18	7.14	0.64	0.32	2.06	0.0198	CCDC50	coiled-coil domain containing 50
**204457_s_at**	11.94	10.9	0.5	0.45	2.06	0.018	GAS1	growth arrest-specific 1

**226432_at**	7.2	6.16	0.59	0.28	2.05	0.0289	ETNK1	ethanolamine kinase 1
**232060_at**	8.53	7.49	0.43	0.22	2.05	0.0068	ROR1	receptor tyrosine kinase-like orphan receptor 1
**227224_at**	9.26	8.22	0.12	0.18	2.05	8.77E-05	RALGPS2	Ral GEF with PH domain and SH3 binding motif 2
**207108_s_at**	7.26	6.22	0.58	0.21	2.05	0.041	NIPBL	Nipped-B homolog (Drosophila)
**213388_at**	8.25	7.21	0.35	0.4	2.05	0.0132	PDE4DIP	phosphodiesterase 4D interacting protein
**208016_s_at**	7.62	6.59	0.77	0.39	2.05	0.0184	AGTR1	angiotensin II receptor, type 1
**225639_at**	9.01	7.97	0.27	0.08	2.05	0.0005	SKAP2	src kinase associated phosphoprotein 2
**203378_at**	7.64	6.61	0.53	0.12	2.04	0.019	PCF11	PCF11 cleavage and polyadenylation factor subunit
**211327_x_at**	7.35	6.32	0.25	0.22	2.04	0.0021	HFE	hemochromatosis
**229222_at**	7.67	6.64	0.64	0.21	2.04	0.0358	ACSS3	acyl-CoA synthetase short-chain family member 3
**205357_s_at**	8.94	7.92	0.83	0.38	2.04	0.0334	AGTR1	angiotensin II receptor, type 1
**205447_s_at**	7.86	6.83	0.45	0.25	2.04	0.0385	MAP3K12	mitogen-activated protein kinase kinase kinase 12
**1554451_s_at**	7.46	6.43	0.5	0.36	2.04	0.0379	DNAJC14	DnaJ (Hsp40) homolog, subfamily C, member 14
**228693_at**	7.54	6.52	0.72	0.33	2.03	0.0441	CCDC50	coiled-coil domain containing 50
**224826_at**	8.61	7.59	0.28	0.36	2.03	0.0066	GPCPD1	glycerophosphocholine phosphodiesterase 1
**227025_at**	6.2	5.18	0.08	0.31	2.02	0.002	PPHLN1	periphilin 1
**233543_s_at**	7.08	6.06	0.37	0.4	2.02	0.019	FAM175A	family with sequence similarity 175, member A
**205731_s_at**	5.92	4.9	0.45	0.21	2.02	0.033	NCOA2	nuclear receptor coactivator 2
**228716_at**	7.53	6.51	0.46	0.11	2.02	0.0066	THRB	thyroid hormone receptor, beta
**228471_at**	4.69	3.68	0.33	0.33	2.02	0.0094	ANKRD44	ankyrin repeat domain 44
**223854_at**	5.54	4.53	0.74	0.36	2.02	0.0163	PCDHB10	protocadherin beta 10
**201010_s_at**	11.62	10.61	0.28	0.31	2.01	0.0087	TXNIP	thioredoxin interacting protein
**229431_at**	6.72	5.71	0.25	0.28	2.01	0.0014	RFXAP	regulatory factor X-associated protein
**209456_s_at**	7.71	6.7	0.65	0.16	2.01	0.0163	FBXW11	F-box and WD repeat domain containing 11
**236201_at**	4.32	3.31	0.08	0.16	2.01	0.0002	SPTLC3	serine palmitoyltransferase, long chain base subunit 3
**213106_at**	5.11	4.1	0.15	0.14	2.01	0.0001	ATP8A1	ATPase, aminophospholipid transporter (APLT), class I, type 8A, member 1
**233827_s_at**	8.19	7.18	0.51	0.25	2.01	0.0282	SUPT16H	SPT16 homolog, facilitates chromatin remodeling subunit

**232064_at**	5.88	4.88	0.23	0.4	2.01	0.0036	FER	fer (fps/fes related) tyrosine kinase
**238677_at**	5.38	4.38	0.25	0.31	2	0.0043	WDR36	WD repeat domain 36
**212569_at**	8.48	7.47	0.34	0.15	2	0.0023	SMCHD1	structural maintenance of chromosomes flexible hinge domain containing 1
**205961_s_at**	9.51	8.51	0.18	0.42	2	0.003	PSIP1	PC4 and SFRS1 interacting protein 1
**244563_at**	6.59	5.59	0.36	0.25	2	0.0064	QSER1	glutamine and serine rich 1
**202259_s_at**	7.49	6.48	0.22	0.21	2	0.0005	N4BP2L2	NEDD4 binding protein 2-like 2
**218284_at**	8.78	7.77	0.22	0.08	2	0.0005	SMAD3	SMAD family member 3
**230472_at**	9.81	8.81	0.08	0.36	2	0.0012	IRX1	iroquois homeobox 1
**239897_at**	6.5	5.5	0.22	0.42	2	0.0079	BCLAF1	BCL2-associated transcription factor 1
**226338_at**	9.26	8.26	0.4	0.23	2	0.0116	TMEM55A	transmembrane protein 55A
**202626_s_at**	4.85	5.85	0.3	0.55	-2	0.018	LYN	LYN proto-oncogene, Src family tyrosine kinase
**230309_at**	5.89	6.89	0.18	0.69	-2	0.0296	BHMT2	betaine--homocysteine S-methyltransferase 2
**236140_at**	6.86	7.86	0.32	0.17	-2.01	0.0056	GCLM	glutamate-cysteine ligase, modifier subunit
**207000_s_at**	7.46	8.46	0.26	0.46	-2.01	0.0279	PPP3CC	protein phosphatase 3, catalytic subunit, gamma isozyme
**209815_at**	4.45	5.46	0.5	0.27	-2.01	0.0146	PTCH1	patched 1
**200920_s_at**	10.23	11.24	0.06	0.67	-2.01	0.0166	BTG1	B-cell translocation gene 1, anti-proliferative
**209738_x_at**	10.19	11.2	0.4	0.3	-2.01	0.0037	PSG6	pregnancy specific beta-1-glycoprotein 6
**214797_s_at**	5.79	6.8	0.16	0.34	-2.01	0.0044	CDK18	cyclin-dependent kinase 18
**238025_at**	6.21	7.22	0.47	0.35	-2.02	0.0237	MLKL	mixed lineage kinase domain-like
**201037_at**	9.12	10.14	0.4	0.62	-2.02	0.0333	PFKP	phosphofructokinase, platelet
**214022_s_at**	10.27	11.29	0.37	0.43	-2.02	0.0161	IFITM1	interferon induced transmembrane protein 1
**1553141_at**	4.88	5.89	0.15	0.97	-2.02	0.0293	LACC1	laccase (multicopper oxidoreductase) domain containing 1
**1554443_s_at**	5.1	6.12	0.15	0.51	-2.02	0.0203	BEST1	bestrophin 1
**227179_at**	5.58	6.6	0.32	0.38	-2.02	0.0094	STAU2	staufen double-stranded RNA binding protein 2
**244444_at**	3.71	4.73	0.35	0.27	-2.03	0.0086	PKD1L2	polycystic kidney disease 1-like 2 (gene/pseudogene)
**225177_at**	5.36	6.38	0.48	0.83	-2.03	0.0424	RAB11FIP1	RAB11 family interacting protein 1 (class I)
**213285_at**	5.3	6.32	0.24	0.8	-2.03	0.039	TMEM30B	transmembrane protein 30B
**221602_s_at**	5.92	6.95	0.16	0.49	-2.04	0.0315	FCMR	Fc fragment of IgM receptor

**201490_s_at**	8.56	9.58	0.35	0.28	-2.04	0.0029	PPIF	peptidylprolyl isomerase F
**226924_at**	6.99	8.01	0.3	0.17	-2.04	0.0014	LINC00909	long intergenic non-protein coding RNA 909
**210426_x_at**	4.9	5.93	0.35	0.37	-2.04	0.004	RORA	RAR-related orphan receptor A
**46270_at**	7.31	8.34	0.38	0.17	-2.04	0.0071	UBAP1	ubiquitin associated protein 1
**214073_at**	7.11	8.14	0.38	0.31	-2.04	0.0143	CTTN	cortactin
**236193_at**	5.56	6.59	0.39	0.39	-2.05	0.0221	HIST1H2BC	histone cluster 1, H2bc
**209383_at**	8.5	9.53	0.48	0.19	-2.05	0.0335	DDIT3	DNA-damage-inducible transcript 3
**223690_at**	8.49	9.53	0.77	0.35	-2.05	0.0113	LTBP2	latent transforming growth factor beta binding protein 2
**209594_x_at**	9.8	10.84	0.37	0.25	-2.05	0.0025	PSG9	pregnancy specific beta-1-glycoprotein 9
**226129_at**	5.32	6.36	0.27	0.33	-2.06	0.0047	FAM83H	family with sequence similarity 83, member H
**1553572_a_at**	6.77	7.81	0.37	0.36	-2.06	0.0109	CYGB	cytoglobin
**205376_at**	6.5	7.54	0.5	0.35	-2.06	0.0027	INPP4B	inositol polyphosphate-4-phosphatase type II B
**239286_at**	6.07	7.11	0.11	0.48	-2.06	0.0053	CDH11	cadherin 11, type 2, OB-cadherin (osteoblast)
**208763_s_at**	7.69	8.74	0.13	0.2	-2.06	0.0001	TSC22D3	TSC22 domain family, member 3
**201842_s_at**	10.41	11.46	0.28	0.51	-2.07	0.005	EFEMP1	EGF containing fibulin-like extracellular matrix protein 1
**242037_at**	6.41	7.46	0.4	0.34	-2.07	0.0041	ASPH	aspartate beta-hydroxylase
**221933_at**	3.44	4.49	0.47	0.37	-2.07	0.0317	NLGN4X	neuroligin 4, X-linked
**237817_at**	4.62	5.67	0.18	0.39	-2.07	0.0093	SSR3	signal sequence receptor, gamma (translocon-associated protein gamma)
**204472_at**	8.28	9.33	1.09	0.54	-2.07	0.0433	GEM	GTP binding protein overexpressed in skeletal muscle
**200738_s_at**	11.86	12.91	0.2	0.56	-2.08	0.029	PGK1	phosphoglycerate kinase 1
**205185_at**	4.38	5.44	0.34	0.61	-2.08	0.0383	SPINK5	serine peptidase inhibitor, Kazal type 5
**203028_s_at**	8.87	9.93	1.13	0.41	-2.08	0.0388	CYBA	cytochrome b-245, alpha polypeptide
**219367_s_at**	5.43	6.49	0.36	0.35	-2.09	0.0033	NRP2	neuropilin 2
**210845_s_at**	8.24	9.3	0.42	0.23	-2.09	0.0045	PLAUR	plasminogen activator, urokinase receptor
**212659_s_at**	6.14	7.2	0.23	0.36	-2.09	0.0028	IL1RN	interleukin 1 receptor antagonist
**204217_s_at**	5.37	6.43	0.69	0.5	-2.09	0.0478	RTN2	reticulon 2
**225252_at**	8.61	9.68	0.29	0.13	-2.1	0.0012	SRXN1	sulfiredoxin 1
**204669_s_at**	6.58	7.65	0.46	0.37	-2.1	0.02	RNF24	ring finger protein 24

**224506_s_at**	6.25	7.32	0.56	0.41	-2.1	0.0334	PLPP7	phospholipid phosphatase 7 (inactive)
**232617_at**	7.03	8.1	0.33	0.39	-2.11	0.003	CTSS	cathepsin S
**203325_s_at**	8.23	9.31	0.23	0.73	-2.11	0.0282	COL5A1	collagen, type V, alpha 1
**204802_at**	4.49	5.57	0.36	0.4	-2.11	0.0053	RRAD	Ras-related associated with diabetes
**201262_s_at**	4.84	5.91	0.18	0.52	-2.11	0.0052	BGN	biglycan
**210215_at**	4.01	5.09	0.45	0.53	-2.11	0.0296	TFR2	transferrin receptor 2
**214632_at**	5.11	6.19	0.28	0.46	-2.11	0.0137	NRP2	neuropilin 2
**204059_s_at**	8.55	9.64	0.24	0.13	-2.12	0.0006	ME1	malic enzyme 1, NADP(+)-dependent, cytosolic
**223393_s_at**	7.03	8.11	0.59	0.31	-2.13	0.0496	TSHZ3	teashirt zinc finger homeobox 3
**218145_at**	8.19	9.28	0.08	0.1	-2.13	2.29E-05	TRIB3	tribbles pseudokinase 3
**206710_s_at**	8.21	9.3	0.3	0.34	-2.13	0.0086	EPB41L3	erythrocyte membrane protein band 4.1-like 3
**203925_at**	8.84	9.94	0.33	0.28	-2.14	0.0021	GCLM	glutamate-cysteine ligase, modifier subunit
**212110_at**	10.04	11.14	0.12	0.78	-2.14	0.0227	SLC39A14	solute carrier family 39 (zinc transporter), member 14
**239823_at**	5.48	6.58	0.16	0.75	-2.14	0.0178	LOC101927841	uncharacterized LOC101927841
**207733_x_at**	8.04	9.14	0.55	0.28	-2.14	0.0154	PSG9	pregnancy specific beta-1-glycoprotein 9
**214290_s_at**	10.55	11.65	0.2	0.42	-2.14	0.0043	HIST2H2AA3; HIST2H2AA4	histone cluster 2, H2aa3; histone cluster 2, H2aa4
**200921_s_at**	10.54	11.65	0.14	0.54	-2.15	0.0059	BTG1	B-cell translocation gene 1, anti-proliferative
**206552_s_at**	2.85	3.96	0.02	0.5	-2.15	0.0026	TAC1	tachykinin, precursor 1
**206237_s_at**	4.95	6.06	0.21	0.8	-2.15	0.0115	NRG1	neuregulin 1
**210749_x_at**	7.35	8.46	0.26	0.62	-2.15	0.0332	DDR1	discoidin domain receptor tyrosine kinase 1
**34408_at**	6.92	8.03	0.69	0.29	-2.16	0.0287	RTN2	reticulon 2
**208025_s_at**	6.39	7.5	0.67	0.35	-2.16	0.0255	HMGA2	high mobility group AT-hook 2
**210387_at**	4.65	5.76	0.13	0.05	-2.16	1.49E-05	HIST1H2BG	histone cluster 1, H2bg
**230799_at**	5.82	6.94	0.46	0.55	-2.16	0.034	LINC01119	long intergenic non-protein coding RNA 1119
**223276_at**	8.75	9.86	0.27	0.58	-2.16	0.0148	SMIM3	small integral membrane protein 3
**201502_s_at**	9.56	10.67	0.13	0.82	-2.17	0.0261	NFKBIA	nuclear factor of kappa light polypeptide gene enhancer in B-cells inhibitor, alpha
**225382_at**	6.38	7.5	0.21	0.41	-2.17	0.0026	ZNF275	zinc finger protein 275
**215977_x_at**	5.64	6.76	0.67	0.55	-2.17	0.0226	GK	glycerol kinase

**204347_at**	6.83	7.95	0.56	0.92	-2.17	0.026	AK4	adenylate kinase 4
**213789_at**	7.14	8.26	0.21	0.74	-2.17	0.0145	EBP	emopamil binding protein (sterol isomerase)
**232382_s_at**	7.7	8.82	0.6	0.33	-2.19	0.003	PCMTD1	protein-L-isoaspartate (D-aspartate) O-methyltransferase domain containing 1
**221865_at**	4.52	5.65	0.43	0.3	-2.19	0.0202	C9orf91	chromosome 9 open reading frame 91
**203399_x_at**	8.61	9.74	0.45	0.36	-2.19	0.0017	PSG3	pregnancy specific beta-1-glycoprotein 3
**204224_s_at**	6.08	7.21	0.65	1.07	-2.19	0.0168	GCH1	GTP cyclohydrolase 1
**212816_s_at**	5.22	6.35	0.93	0.59	-2.2	0.0425	CBS; CBSL	cystathionine-beta-synthase; cystathionine-beta-synthase like
**211924_s_at**	8.33	9.47	0.38	0.27	-2.2	0.0052	PLAUR	plasminogen activator, urokinase receptor
**204348_s_at**	7.82	8.96	0.57	0.81	-2.2	0.0089	AK4	adenylate kinase 4
**218280_x_at**	9.43	10.58	0.23	0.47	-2.21	0.0041	HIST2H2AA3; HIST2H2AA4	histone cluster 2, H2aa3; histone cluster 2, H2aa4
**231666_at**	3.52	4.67	0.1	0.56	-2.21	0.0043	PAX3	paired box 3
**229441_at**	7.64	8.79	0.68	0.68	-2.21	0.0397	PRSS23	protease, serine, 23
**210058_at**	5.21	6.35	0.32	0.14	-2.22	0.0006	MAPK13	mitogen-activated protein kinase 13
**226452_at**	6.86	8.01	0.14	0.95	-2.22	0.0366	PDK1	pyruvate dehydrogenase kinase, isozyme 1
**230665_at**	5.78	6.93	0.28	0.36	-2.23	0.0035	GUCY1A2	guanylate cyclase 1, soluble, alpha 2
**216603_at**	5.73	6.88	0.09	0.41	-2.23	0.0003	SLC7A8	solute carrier family 7 (amino acid transporter light chain, L system), member 8
**233177_s_at**	5.31	6.47	0.23	0.8	-2.23	0.0187	PNKD	paroxysmal nonkinesigenic dyskinesia
**224240_s_at**	5.75	6.91	0.29	0.52	-2.24	0.0034	CCL28	chemokine (C-C motif) ligand 28
**221249_s_at**	7.15	8.32	0.2	0.4	-2.25	0.0024	FAM117A	family with sequence similarity 117, member A
**227557_at**	6.51	7.68	0.17	0.83	-2.25	0.0194	SCARF2	scavenger receptor class F, member 2
**207469_s_at**	7.14	8.32	0.58	0.28	-2.26	0.0125	PIR	pirin
**203823_at**	9.04	10.22	0.45	0.3	-2.26	0.0031	RGS3	regulator of G-protein signaling 3
**227072_at**	7.54	8.72	0.6	0.39	-2.26	0.0113	RTTN	rotatin
**208916_at**	8.22	9.4	0.28	0.57	-2.26	0.0131	SLC1A5	solute carrier family 1 (neutral amino acid transporter), member 5

**1552508_at**	5.48	6.66	0.22	1.05	-2.27	0.0364	KCNE4	potassium channel, voltage gated subfamily E regulatory beta subunit 4
**233562_at**	4.98	6.17	0.14	0.33	-2.27	0.0006	LINC00839	long intergenic non-protein coding RNA 839
**226436_at**	4.97	6.16	0.43	0.51	-2.27	0.0339	RASSF4	Ras association (RalGDS/AF-6) domain family member 4
**230144_at**	6.3	7.49	0.32	0.73	-2.27	0.035	GRIA3	glutamate receptor, ionotropic, AMPA 3
**225626_at**	7.55	8.74	0.88	0.32	-2.27	0.0169	PAG1	phosphoprotein membrane anchor with glycosphingolipid microdomains 1
**209122_at**	11.29	12.48	0.51	0.56	-2.28	0.0071	PLIN2	perilipin 2
**218717_s_at**	4.78	5.97	0.28	0.36	-2.29	0.0026	P3H2	prolyl 3-hydroxylase 2
**201566_x_at**	8.5	9.69	0.38	0.86	-2.29	0.0403	ID2	inhibitor of DNA binding 2, dominant negative helix-loop-helix protein
**222646_s_at**	9.23	10.42	0.22	0.75	-2.29	0.0197	ERO1A	endoplasmic reticulum oxidoreductase alpha
**221903_s_at**	7.96	9.16	0.14	0.47	-2.29	0.0092	CYLD	cylindromatosis (turban tumor syndrome)
**223063_at**	9.8	10.99	0.4	0.81	-2.29	0.0266	C1orf198	chromosome 1 open reading frame 198
**210479_s_at**	4.46	5.66	0.55	0.42	-2.29	0.0095	RORA	RAR-related orphan receptor A
**203455_s_at**	10.45	11.65	0.16	0.31	-2.3	0.0008	SAT1	spermidine/spermine N1-acetyltransferase 1
**203835_at**	9.96	11.16	0.33	0.37	-2.3	0.0055	LRRC32	leucine rich repeat containing 32
**230259_at**	6.23	7.44	0.29	0.09	-2.31	0.0002	FUOM	fucose mutarotase
**238990_x_at**	3.74	4.95	0.47	0.54	-2.31	0.008	TRIM61	tripartite motif containing 61
**205117_at**	5.97	7.17	0.43	0.54	-2.31	0.015	FGF1	fibroblast growth factor 1 (acidic)
**223541_at**	4.33	5.54	0.03	0.55	-2.31	0.012	HAS3	hyaluronan synthase 3
**209911_x_at**	8.8	10.01	0.26	0.4	-2.32	0.0042	HIST1H2BD	histone cluster 1, H2bd
**204339_s_at**	6.21	7.43	0.24	0.96	-2.32	0.0294	RGS4	regulator of G-protein signaling 4
**232649_at**	6.14	7.36	0.42	0.36	-2.33	0.0095	GLDN	gliomedin
**204400_at**	7.55	8.78	0.87	0.45	-2.33	0.0493	EFS	embryonal Fyn-associated substrate
**202545_at**	5.92	7.15	0.33	0.44	-2.33	0.0085	PRKCD	protein kinase C, delta
**210367_s_at**	8.69	9.92	0.12	0.53	-2.34	0.0015	PTGES	prostaglandin E synthase
**217871_s_at**	11.39	12.61	0.31	0.47	-2.34	0.0071	MIF	macrophage migration inhibitory factor (glycosylation-inhibiting factor)

**201425_at**	7.12	8.36	0.33	0.28	-2.35	0.0009	ALDH2	aldehyde dehydrogenase 2 family (mitochondrial)
**227410_at**	7.1	8.33	0.74	0.79	-2.35	0.0155	FAM43A	family with sequence similarity 43, member A
**201565_s_at**	10.14	11.39	0.49	0.68	-2.37	0.0189	ID2	inhibitor of DNA binding 2, dominant negative helix-loop-helix protein
**224392_s_at**	5.04	6.28	0.44	0.47	-2.37	0.0044	OPN3	opsin 3
**207542_s_at**	6.17	7.41	0.12	0.87	-2.37	0.0044	AQP1	aquaporin 1 (Colton blood group)
**231755_at**	3.71	4.95	0.23	0.39	-2.37	0.0027	IL36B	interleukin 36, beta
**209720_s_at**	2.96	4.21	0.17	0.77	-2.37	0.0299	SERPINB3	serpin peptidase inhibitor, clade B (ovalbumin), member 3
**224942_at**	8.43	9.68	0.2	0.54	-2.38	0.0119	PAPPA	pregnancy-associated plasma protein A, pappalysin 1
**242064_at**	4.6	5.85	0.03	0.57	-2.38	0.0023	SDK2	sidekick cell adhesion molecule 2
**210396_s_at**	8.24	9.49	0.41	0.49	-2.38	0.0172	BOLA2; SMG1P2; SMG1P5	bolA family member 2; SMG1 pseudogene 2; SMG1 pseudogene 5
**209398_at**	8.39	9.65	0.34	0.45	-2.39	0.0028	HIST1H1C	histone cluster 1, H1c
**219257_s_at**	7.55	8.8	0.48	0.52	-2.39	0.0065	SPHK1	sphingosine kinase 1
**223062_s_at**	8.18	9.44	0.71	0.87	-2.39	0.0369	PSAT1	phosphoserine aminotransferase 1
**222668_at**	3.78	5.04	0.31	0.6	-2.4	0.0112	KCTD15	potassium channel tetramerization domain containing 15
**222062_at**	3.95	5.21	0.12	0.59	-2.41	0.0096	IL27RA	interleukin 27 receptor, alpha
**210732_s_at**	6.3	7.57	0.53	0.47	-2.42	0.0366	LGALS8	lectin, galactoside-binding, soluble, 8
**212946_at**	7.67	8.95	0.24	0.56	-2.42	0.0038	VWA8	von Willebrand factor A domain containing 8
**202052_s_at**	9.08	10.37	0.43	0.75	-2.43	0.0282	RAI14	retinoic acid induced 14
**221658_s_at**	4.17	5.45	0.15	0.67	-2.43	0.0073	IL21R	interleukin 21 receptor
**213100_at**	5.54	6.83	0.18	0.41	-2.44	0.0022	UNC5B	unc-5 netrin receptor B
**225817_at**	6.47	7.77	0.66	0.48	-2.45	0.0162	CGNL1	cingulin-like 1
**229748_x_at**	7.19	8.49	0.21	0.55	-2.46	0.0027	LOC100233156; LOC105379554; MAFIP; TEKT4P2	tektin 4 pseudogene; maFF-interacting protein; MAFF interacting protein (pseudogene); tektin 4 pseudogene 2
**225646_at**	8.2	9.5	0.67	0.07	-2.46	0.0019	CTSC	cathepsin C
**225020_at**	6.67	7.97	0.54	0.46	-2.47	0.0136	DAB2IP	DAB2 interacting protein
**218677_at**	4.78	6.09	0.15	1.11	-2.47	0.0367	S100A14	S100 calcium binding protein A14

**228937_at**	7.44	8.75	0.17	0.79	-2.47	0.002	LACC1	laccase (multicopper oxidoreductase) domain containing 1
**225285_at**	7.46	8.76	0.67	0.29	-2.47	0.0012	BCAT1	branched chain amino-acid transaminase 1, cytosolic
**202022_at**	7.66	8.97	0.29	1.06	-2.48	0.0209	ALDOC	aldolase C, fructose-bisphosphate
**202150_s_at**	5.44	6.75	0.23	0.4	-2.48	0.0017	NEDD9	neural precursor cell expressed, developmentally down-regulated 9
**206969_at**	5.01	6.33	0.8	0.71	-2.49	0.0255	KRT34; LOC100653 049	keratin 34, type I; keratin, type I cuticular Ha4
**229432_at**	5.36	6.68	0.36	0.35	-2.5	0.0046	NAGS	N-acetylglutamate synthase
**204338_s_at**	5.84	7.17	0.47	1.24	-2.5	0.0402	RGS4	regulator of G-protein signaling 4
**240572_s_at**	4.46	5.79	0.52	0.32	-2.5	0.0238	LOC374443	C-type lectin domain family 2, member D pseudogene
**208180_s_at**	4.76	6.09	0.17	1.01	-2.51	0.0328	HIST1H4H	histone cluster 1, H4h
**224817_at**	9.36	10.69	0.23	0.65	-2.51	0.0106	SH3PXD2A	SH3 and PX domains 2A
**208779_x_at**	6.67	8	0.24	0.61	-2.52	0.03	DDR1	discoidin domain receptor tyrosine kinase 1
**218484_at**	5.84	7.18	0.18	0.77	-2.54	0.0154	NDUFA4L 2	NADH dehydrogenase (ubiquinone) 1 alpha subcomplex, 4-like 2
**217356_s_at**	11.01	12.35	0.38	0.69	-2.54	0.0296	PGK1	phosphoglycerate kinase 1
**217739_s_at**	9.57	10.92	0.5	0.46	-2.54	0.0031	NAMPT	nicotinamide phosphoribosyltransferase
**221840_at**	4.4	5.75	0.34	0.7	-2.55	0.0147	PTPRE	protein tyrosine phosphatase, receptor type, E
**229225_at**	4.7	6.05	0.02	0.76	-2.55	0.0112	NRP2	neuropilin 2
**231202_at**	6.36	7.71	0.62	0.6	-2.55	0.0107	ALDH1L2	aldehyde dehydrogenase 1 family, member L2
**232693_s_at**	6.71	8.07	0.27	1.07	-2.56	0.0465	FBXO16; ZNF395	F-box protein 16; zinc finger protein 395
**219534_x_at**	5.53	6.89	0.54	0.59	-2.56	0.0292	CDKN1C	cyclin-dependent kinase inhibitor 1C (p57, Kip2)
**230748_at**	6.31	7.67	1.02	0.65	-2.57	0.0154	SLC16A6	solute carrier family 16, member 6
**226609_at**	5.97	7.33	0.62	0.41	-2.57	0.0101	DCBLD1	discoidin, CUB and LCCL domain containing 1
**225566_at**	5.69	7.05	0.34	0.68	-2.57	0.0084	NRP2	neuropilin 2
**237094_at**	4.07	5.43	0.2	0.62	-2.57	0.0034	FAM19A5	family with sequence similarity 19 (chemokine (C-C motif)-like), member A5
**227749_at**	6.17	7.54	0.22	0.3	-2.58	0.0004	POU2F2	POU class 2 homeobox 2
**203238_s_at**	5.16	6.52	0.34	0.65	-2.58	0.0018	NOTCH3	notch 3
**226769_at**	7.58	8.95	0.86	0.31	-2.59	0.0118	FIBIN	fin bud initiation factor homolog (zebrafish)

**227949_at**	3.5	4.87	0.15	1.08	-2.59	0.0298	PHACTR3	phosphatase and actin regulator 3
**227330_x_at**	6.76	8.13	0.15	0.54	-2.6	0.0023	LOC100233156; MAFIP; TEKT4P2	tektin 4 pseudogene; MAFF interacting protein (pseudogene); tektin 4 pseudogene 2
**200907_s_at**	9.24	10.62	0.36	1.01	-2.6	0.0246	PALLD	palladin, cytoskeletal associated protein
**219412_at**	5.19	6.58	0.7	0.39	-2.61	0.0175	RAB38	RAB38, member RAS oncogene family
**1553142_at**	4.77	6.16	0.07	1.03	-2.61	0.0049	LACC1	laccase (multicopper oxidoreductase) domain containing 1
**225647_s_at**	8.44	9.83	0.83	0.13	-2.64	0.0099	CTSC	cathepsin C
**204678_s_at**	5.24	6.64	0.16	0.22	-2.64	3.07E-05	KCNK1	potassium channel, two pore domain subfamily K, member 1
**213988_s_at**	9.6	11	0.16	0.58	-2.65	0.0026	SAT1	spermidine/spermine N1-acetyltransferase 1
**216243_s_at**	4.92	6.33	0.43	0.65	-2.65	0.0291	IL1RN	interleukin 1 receptor antagonist
**229802_at**	6.17	7.58	0.5	1.19	-2.66	0.0401	WISP1	WNT1 inducible signaling pathway protein 1
**204734_at**	4.29	5.7	0.09	1.36	-2.66	0.0479	KRT15	keratin 15, type I
**212488_at**	7.84	9.26	0.3	0.7	-2.66	0.0087	COL5A1	collagen, type V, alpha 1
**202952_s_at**	7.58	8.99	1.05	0.81	-2.67	0.04	ADAM12	ADAM metallopeptidase domain 12
**201858_s_at**	4.62	6.03	0.55	0.24	-2.67	0.0021	SRGN	serglycin
**227654_at**	5.75	7.17	0.91	0.83	-2.68	0.0454	FAM65C	family with sequence similarity 65, member C
**200737_at**	9.44	10.86	0.29	0.75	-2.69	0.0203	PGK1	phosphoglycerate kinase 1
**219282_s_at**	6.73	8.16	0.15	0.83	-2.69	0.0026	TRPV2	transient receptor potential cation channel, subfamily V, member 2
**218113_at**	5.9	7.33	0.62	0.04	-2.69	0.0101	TMEM2	transmembrane protein 2
**212281_s_at**	8.13	9.56	0.15	0.65	-2.7	0.004	TMEM97	transmembrane protein 97
**230147_at**	3.24	4.67	0.13	1.23	-2.7	0.0394	F2RL2	coagulation factor II (thrombin) receptor-like 2
**204830_x_at**	9.94	11.38	0.44	0.39	-2.7	0.0012	PSG5	pregnancy specific beta-1-glycoprotein 5
**224762_at**	6.98	8.41	0.3	0.07	-2.7	0.0001	SERINC2	serine incorporator 2
**228483_s_at**	5.34	6.79	0.31	0.93	-2.72	0.0194	TAF9B	TAF9B RNA polymerase II, TATA box binding protein (TBP)-associated factor, 31kDa
**1552721_a_at**	4.42	5.87	0.69	0.76	-2.72	0.017	FGF1	fibroblast growth factor 1 (acidic)
**203828_s_at**	6.2	7.65	0.21	1.2	-2.73	0.0262	IL32	interleukin 32

**214455_at**	4.47	5.92	0.2	0.5	-2.73	0.0011	HIST1H2B C	histone cluster 1, H2bc
**225293_at**	6.65	8.1	0.4	0.99	-2.74	0.0183	COL27A1	collagen, type XXVII, alpha 1
**242945_at**	5.36	6.82	0.61	0.68	-2.74	0.0313	FAM20A	family with sequence similarity 20, member A
**215014_at**	3.86	5.31	0.5	0.18	-2.74	0.0005	KCND3	potassium channel, voltage gated Shal related subfamily D, member 3
**210592_s_at**	10.41	11.87	0.09	0.34	-2.75	0.0002	SAT1	spermidine/spermine N1-acetyltransferase 1
**204879_at**	8.54	10	0.82	0.6	-2.75	0.0444	PDPN	podoplanin
**223278_at**	3.48	4.95	0.38	2.07	-2.76	0.0477	GJB2	gap junction protein beta 2
**242913_at**	4.96	6.43	0.33	0.49	-2.77	0.0009	CLIC6	chloride intracellular channel 6
**205668_at**	3.76	5.23	0.39	0.84	-2.77	0.0322	LY75	lymphocyte antigen 75
**221539_at**	7.9	9.38	0.52	0.29	-2.79	0.0026	EIF4EBP1	eukaryotic translation initiation factor 4E binding protein 1
**200897_s_at**	9.79	11.27	0.39	0.9	-2.79	0.0201	PALLD	palladin, cytoskeletal associated protein
**201487_at**	8.09	9.58	0.74	0.26	-2.8	0.0072	CTSC	cathepsin C
**202637_s_at**	7.08	8.56	0.55	0.65	-2.8	0.0148	ICAM1	intercellular adhesion molecule 1
**234973_at**	6.38	7.87	0.61	0.7	-2.8	0.0115	SLC38A5	solute carrier family 38, member 5
**214265_at**	3.98	5.47	0.29	0.53	-2.81	0.0015	ITGA8	integrin alpha 8
**237460_x_at**	5.06	6.56	0.12	1.1	-2.82	0.0289	LINC01588	long intergenic non-protein coding RNA 1588
**228523_at**	4.48	5.98	0.37	0.64	-2.83	0.0335	NANOS1	nanos homolog 1 (Drosophila)
**238669_at**	6.33	7.83	0.55	1.29	-2.83	0.0228	PTGS1	prostaglandin-endoperoxide synthase 1 (prostaglandin G/H synthase and cyclooxygenase)
**204337_at**	7.06	8.56	0.67	1.4	-2.83	0.0337	RGS4	regulator of G-protein signaling 4
**212282_at**	8.17	9.67	0.17	0.5	-2.83	0.0009	TMEM97	transmembrane protein 97
**205439_at**	5.42	6.92	0.59	0.63	-2.84	0.0195	GSTT2; GSTT2B	glutathione S-transferase theta 2 (gene/pseudogene); glutathione S-transferase theta 2B (gene/pseudogene)
**233868_x_at**	8.16	9.68	1.04	0.16	-2.86	0.0357	ADAM33	ADAM metallopeptidase domain 33
**226869_at**	7.01	8.53	0.33	0.57	-2.86	0.0042	MEGF6	multiple EGF-like-domains 6
**219117_s_at**	8.58	10.1	0.74	0.22	-2.87	0.018	FKBP11	FK506 binding protein 11
**202638_s_at**	6.58	8.1	0.94	0.95	-2.87	0.0476	ICAM1	intercellular adhesion molecule 1

**227868_at**	6.21	7.73	0.26	1.43	-2.87	0.0391	LOC154761	family with sequence similarity 115, member C pseudogene
**215223_s_at**	10.87	12.4	0.4	0.35	-2.88	0.0016	LOC100129518; SOD2	uncharacterized LOC100129518; superoxide dismutase 2, mitochondrial
**225721_at**	7.17	8.7	0.15	1.87	-2.88	0.0237	SYNPO2	synaptopodin 2
**213933_at**	4.7	6.23	0.48	0.78	-2.89	0.0083	PTGER3	prostaglandin E receptor 3 (subtype EP3)
**230966_at**	5.49	7.02	0.45	1.05	-2.89	0.0457	IL4I1	interleukin 4 induced 1
**209928_s_at**	5.75	7.28	0.22	0.73	-2.89	0.0042	MSC	musculin
**227467_at**	6.55	8.09	0.13	0.29	-2.9	6.46E-05	RDH10	retinol dehydrogenase 10 (all-trans)
**202219_at**	7.39	8.93	0.17	0.79	-2.91	0.0138	SLC6A8	solute carrier family 6 (neurotransmitter transporter), member 8
**229655_at**	6.39	7.94	0.36	0.62	-2.92	0.0071	FAM19A5	family with sequence similarity 19 (chemokine (C-C motif)-like), member A5
**229404_at**	10.59	12.14	0.3	0.27	-2.93	9.19E-05	TWIST2	twist family bHLH transcription factor 2
**242342_at**	4.42	5.99	0.49	0.76	-2.96	0.0074	GUCY1A2	guanylate cyclase 1, soluble, alpha 2
**219090_at**	4.56	6.15	0.23	0.69	-3.01	0.0028	SLC24A3	solute carrier family 24 (sodium/potassium/calcium exchanger), member 3
**204984_at**	4.85	6.44	0.4	0.46	-3.02	0.001	GPC4	glypican 4
**1555167_s_at**	7.42	9.01	0.53	0.6	-3.03	0.0037	NAMPT	nicotinamide phosphoribosyltransferase
**213832_at**	4.72	6.32	0.44	0.16	-3.03	0.0002	KCND3	potassium channel, voltage gated Shal related subfamily D, member 3
**229168_at**	5.04	6.64	0.27	0.56	-3.03	0.0017	COL23A1	collagen, type XXIII, alpha 1
**212314_at**	4.81	6.42	0.4	0.7	-3.03	0.0042	SEL1L3	sel-1 suppressor of lin-12-like 3 (C. elegans)
**219454_at**	5.64	7.24	0.38	1.69	-3.04	0.0153	EGFL6	EGF-like-domain, multiple 6
**208607_s_at**	4.91	6.52	0.15	1.03	-3.05	0.0128	SAA1; SAA2; SAA2-SAA4	serum amyloid A1; serum amyloid A2; SAA2-SAA4 readthrough
**204015_s_at**	6.36	7.97	0.85	0.54	-3.05	0.0241	DUSP4	dual specificity phosphatase 4
**201860_s_at**	6.38	7.99	0.43	0.79	-3.05	0.0243	PLAT	plasminogen activator, tissue
**206201_s_at**	2.97	4.58	0.17	1.18	-3.06	0.0059	MEOX2	mesenchyme homeobox 2
**222855_s_at**	5.11	6.73	0.19	0.75	-3.07	0.006	TRPV2	transient receptor potential cation channel, subfamily V, member 2
**214681_at**	5.69	7.31	0.72	0.46	-3.08	0.0497	GK	glycerol kinase

**209087_x_at**	5.39	7.02	0.14	1.39	-3.08	0.0485	MCAM	melanoma cell adhesion molecule
**221601_s_at**	5.07	6.7	0.3	0.71	-3.09	0.0066	FCMR	Fc fragment of IgM receptor
**204556_s_at**	6.52	8.16	0.94	0.11	-3.12	0.0405	DZIP1	DAZ interacting zinc finger protein 1
**205620_at**	5.84	7.49	0.33	0.26	-3.13	9.69E-05	F10	coagulation factor X
**222242_s_at**	4.75	6.4	0.22	1.44	-3.13	0.0464	KLK5	kallikrein related peptidase 5
**57588_at**	4.18	5.84	0.08	0.36	-3.17	0.0001	SLC24A3	solute carrier family 24 (sodium/potassium/calcium exchanger), member 3
**203921_at**	6.86	8.53	0.18	0.71	-3.18	0.0051	CHST2	carbohydrate (N-acetylglucosamine-6-O) sulfotransferase 2
**204341_at**	7.77	9.45	0.09	0.7	-3.2	0.007	TRIM16	tripartite motif containing 16
**215241_at**	3.8	5.48	0.05	0.6	-3.2	0.0024	ANO3	anoctamin 3
**228499_at**	6.37	8.05	0.2	1.02	-3.2	0.0136	PFKFB4	6-phosphofructo-2-kinase/fructose-2,6-biphosphatase 4
**206400_at**	4.6	6.31	0.07	0.89	-3.27	0.0199	LGALS7; LGALS7B	lectin, galactoside-binding, soluble, 7; lectin, galactoside-binding, soluble, 7B
**202796_at**	5.9	7.62	0.68	0.68	-3.27	0.0206	SYNPO	synaptopodin
**222877_at**	4.59	6.31	0.5	0.88	-3.29	0.0045	NRP2	neuropilin 2
**217738_at**	8.74	10.46	0.57	0.6	-3.29	0.0048	NAMPT	nicotinamide phosphoribosyltransferase
**219032_x_at**	6.52	8.24	0.64	0.41	-3.3	0.0029	OPN3	opsin 3
**204924_at**	5.22	6.95	0.28	0.89	-3.31	0.0094	TLR2	toll-like receptor 2
**213348_at**	6.97	8.71	0.81	0.57	-3.33	0.0415	CDKN1C	cyclin-dependent kinase inhibitor 1C (p57, Kip2)
**229910_at**	5.09	6.83	0.23	0.52	-3.34	0.0003	SHE	Src homology 2 domain containing E
**219118_at**	7.41	9.16	0.7	0.29	-3.35	0.0138	FKBP11	FK506 binding protein 11
**202936_s_at**	5.71	7.46	0.92	0.89	-3.36	0.0246	SOX9	SRY box 9
**202295_s_at**	7.27	9.02	0.52	0.43	-3.36	0.0039	CTSH	cathepsin H
**226517_at**	6.96	8.71	0.89	0.2	-3.37	0.0025	BCAT1	branched chain amino-acid transaminase 1, cytosolic
**200923_at**	7.52	9.27	0.73	0.66	-3.37	0.0232	LGALS3BP	lectin, galactoside-binding, soluble, 3 binding protein
**209126_x_at**	6.8	8.56	0.11	1.53	-3.39	0.0441	KRT6B	keratin 6B, type II
**230372_at**	6.47	8.23	0.96	0.41	-3.39	0.026	HAS2	hyaluronan synthase 2
**225720_at**	5.55	7.32	0.37	2.16	-3.41	0.0212	SYNPO2	synaptopodin 2
**210613_s_at**	7.08	8.85	0.76	0.3	-3.41	0.0129	SYNGR1	synaptogyrin 1
**217678_at**	7.37	9.14	0.81	0.63	-3.41	0.0028	SLC7A11	solute carrier family 7 (anionic amino acid transporter light chain, xc-system), member 11

**217272_s_at**	3.65	5.42	0.05	1.18	-3.41	0.0468	SERPINB13	serpin peptidase inhibitor, clade B (ovalbumin), member 13
**242414_at**	8.06	9.85	1.26	0.32	-3.45	0.0174	QPRT	quinolinate phosphoribosyltransferase
**204679_at**	7.05	8.83	0.77	0.37	-3.45	0.0011	KCNK1	potassium channel, two pore domain subfamily K, member 1
**205627_at**	5.3	7.09	0.49	1.16	-3.46	0.0336	CDA	cytidine deaminase
**214175_x_at**	7.67	9.46	0.46	0.58	-3.47	0.0049	PDLIM4	PDZ and LIM domain 4
**204682_at**	9.06	10.86	0.68	0.41	-3.47	0.0033	LTBP2	latent transforming growth factor beta binding protein 2
**225342_at**	7.87	9.66	0.78	1.04	-3.47	0.0203	AK4	adenylate kinase 4
**201849_at**	10.5	12.3	0.16	1.02	-3.48	0.0368	BNIP3	BCL2/adenovirus E1B 19kDa interacting protein 3
**230864_at**	5	6.8	0.2	1.44	-3.49	0.0339	NIM1K	NIM1 serine/threonine protein kinase
**202644_s_at**	7.08	8.89	0.07	1.08	-3.51	0.0093	TNFAIP3	tumor necrosis factor, alpha-induced protein 3
**227180_at**	3.4	5.22	0.14	0.64	-3.53	0.0006	ELOVL7	ELOVL fatty acid elongase 7
**201859_at**	5.36	7.19	0.92	0.63	-3.54	0.0154	SRGN	serglycin
**214452_at**	4.73	6.55	0.3	0.23	-3.55	6.83E-05	BCAT1	branched chain amino-acid transaminase 1, cytosolic
**214456_x_at**	5.51	7.35	0.34	1.07	-3.56	0.0117	SAA1; SAA2	serum amyloid A1; serum amyloid A2
**1552632_a_at**	5.94	7.78	0.79	1.01	-3.57	0.0128	ARSG	arylsulfatase G
**219410_at**	9.76	11.6	0.21	0.95	-3.59	0.0196	TMEM45A	transmembrane protein 45A
**203889_at**	7.73	9.57	0.86	0.16	-3.59	0.0031	SCG5	secretogranin V
**212171_x_at**	7.22	9.07	0.1	1.25	-3.61	0.0231	VEGFA	vascular endothelial growth factor A
**201673_s_at**	7.39	9.25	0.07	1.05	-3.62	0.0381	GYS1	glycogen synthase 1 (muscle)
**242680_at**	3.67	5.53	0.18	1.3	-3.63	0.0144	AVPR1A	arginine vasopressin receptor 1A
**202643_s_at**	6.03	7.91	0.08	1.24	-3.66	0.0129	TNFAIP3	tumor necrosis factor, alpha-induced protein 3
**1554500_a_at**	3.57	5.46	0.21	0.32	-3.71	6.74E-05	RGS7	regulator of G-protein signaling 7
**233555_s_at**	5.25	7.15	0.76	0.29	-3.72	0.0122	SULF2	sulfatase 2
**238750_at**	5.95	7.85	0.33	0.77	-3.73	0.0035	CCL28	chemokine (C-C motif) ligand 28
**202733_at**	9.88	11.78	0.45	0.78	-3.75	0.0104	P4HA2	prolyl 4-hydroxylase, alpha polypeptide II
**224215_s_at**	4.35	6.27	0.27	1.64	-3.78	0.0321	DLL1	delta-like 1 (Drosophila)
**229172_at**	5.28	7.2	0.39	0.03	-3.79	4.85E-05	HSPA12B	heat shock 70kD protein 12B
**224027_at**	3.68	5.61	0.32	1.02	-3.79	0.0121	CCL28	chemokine (C-C motif) ligand 28
**209082_s_at**	6.31	8.24	0.63	1.01	-3.79	0.0156	COL18A1	collagen, type XVIII, alpha 1

**223861_at**	3.21	5.16	0.28	0.72	-3.85	0.0044	HORMAD 1	HORMA domain containing 1
**222484_s_at**	5.1	7.05	0.22	1.66	-3.87	0.0302	CXCL14	chemokine (C-X-C motif) ligand 14
**203180_at**	8.89	10.85	0.69	0.59	-3.89	0.0125	ALDH1A3	aldehyde dehydrogenase 1 family, member A3
**206134_at**	3.48	5.45	0.25	1.3	-3.9	0.0063	ADAMDE C1	ADAM-like, decysin 1
**222449_at**	6.35	8.33	0.51	0.33	-3.94	0.0001	PMEPA1	prostate transmembrane protein, androgen induced 1
**209921_at**	7.32	9.3	0.77	0.57	-3.94	0.0031	SLC7A11	solute carrier family 7 (anionic amino acid transporter light chain, xc- system), member 11
**207992_s_at**	6.67	8.65	0.41	1.11	-3.96	0.0196	AMPD3	adenosine monophosphate deaminase 3
**204463_s_at**	3.2	5.21	0.45	1.14	-4.01	0.0133	EDNRA	endothelin receptor type A
**216604_s_at**	6.33	8.35	0.31	0.19	-4.05	2.47E-05	SLC7A8	solute carrier family 7 (amino acid transporter light chain, L system), member 8
**229390_at**	4.27	6.29	0.63	1.15	-4.05	0.0288	FAM26F	family with sequence similarity 26, member F
**204301_at**	4.78	6.81	0.24	1.03	-4.07	0.0056	KBTBD11	kelch repeat and BTB (POZ) domain containing 11
**204044_at**	7.21	9.25	1.3	0.44	-4.11	0.0129	LOC105369 247; QPRT	nicotinate-nucleotide pyrophosphorylase [carboxylating]-like; quinolinate phosphoribosyltransferase
**205128_x_at**	6.18	8.23	0.21	1.16	-4.13	0.0067	PTGS1	prostaglandin-endoperoxide synthase 1 (prostaglandin G/H synthase and cyclooxygenase)
**201195_s_at**	5.96	8.01	0.63	0.96	-4.15	0.0029	SLC7A5	solute carrier family 7 (amino acid transporter light chain, L system), member 5
**206118_at**	5.18	7.24	0.46	0.35	-4.17	0.0008	STAT4	signal transducer and activator of transcription 4
**210794_s_at**	5.46	7.52	0.23	0.59	-4.18	0.0004	MEG3	maternally expressed 3 (non-protein coding)
**222379_at**	6	8.07	0.32	0.65	-4.18	0.001	KCNE4	potassium channel, voltage gated subfamily E regulatory beta subunit 4
**226034_at**	5.92	8.01	1	0.84	-4.26	0.04	DUSP4	dual specificity phosphatase 4
**202718_at**	6.76	8.85	0.45	1.28	-4.26	0.003	IGFBP2	insulin like growth factor binding protein 2
**1558846_at**	4.16	6.25	0.37	0.69	-4.27	0.002	PNLIPRP3	pancreatic lipase-related protein 3
**206421_s_at**	5.48	7.57	0.63	1.04	-4.27	0.0033	SERPINB7	serpin peptidase inhibitor, clade B (ovalbumin), member 7

**218691_s_at**	4.45	6.58	0.54	0.94	-4.36	0.0105	PDLIM4	PDZ and LIM domain 4
**209436_at**	7.25	9.37	1.03	1.39	-4.36	0.0277	SPON1	spondin 1, extracellular matrix protein
**231233_at**	5.2	7.32	0.06	0.91	-4.37	0.0083	PCAT6	prostate cancer associated transcript 6 (non-protein coding)
**206336_at**	5.36	7.5	0.97	1.85	-4.4	0.0097	CXCL6	chemokine (C-X-C motif) ligand 6
**218507_at**	7.12	9.26	0.5	1.43	-4.42	0.0316	HILPDA	hypoxia inducible lipid droplet-associated
**210372_s_at**	4.53	6.67	0.09	1.7	-4.43	0.0445	TPD52L1	tumor protein D52-like 1
**244317_at**	6.38	8.53	1.46	0.75	-4.44	0.0498	KIAA1324L	KIAA1324-like
**229479_at**	3.44	5.59	0.08	1.22	-4.45	0.0379	LINC01614	long intergenic non-protein coding RNA 1614
**209772_s_at**	4.45	6.6	0.12	1.69	-4.45	0.0495	CD24	CD24 molecule
**205266_at**	5.76	7.92	0.48	0.91	-4.46	0.0017	LIF	leukemia inhibitory factor
**203896_s_at**	4.5	6.66	0.11	1.73	-4.46	0.0142	PLCB4	phospholipase C, beta 4
**238835_at**	3.15	5.32	0.09	1.42	-4.48	0.0148	AVPR1A	arginine vasopressin receptor 1A
**204681_s_at**	4.4	6.59	0.2	1.02	-4.57	0.0133	RAPGEF5	Rap guanine nucleotide exchange factor 5
**226021_at**	7.16	9.37	0.4	0.5	-4.61	0.0004	RDH10	retinol dehydrogenase 10 (all-trans)
**209277_at**	7.59	9.8	1.09	1.16	-4.62	0.0056	TFPI2	tissue factor pathway inhibitor 2
**201163_s_at**	9.56	11.78	1.14	0.43	-4.65	0.0036	IGFBP7	insulin like growth factor binding protein 7
**209016_s_at**	4.3	6.52	0.15	1.07	-4.65	0.0088	KRT7	keratin 7, type II
**209047_at**	5.47	7.7	0.2	1.07	-4.68	0.0018	AQP1	aquaporin 1 (Colton blood group)
**219049_at**	5.65	7.87	1.27	0.67	-4.68	0.0312	CSGALNACT1	chondroitin sulfate N-acetylgalactosaminyltransferase 1
**213823_at**	6.2	8.43	1.1	0.27	-4.7	0.0285	HOXA11	homeobox A11
**209278_s_at**	8.69	10.93	1.23	1.09	-4.73	0.0101	TFPI2	tissue factor pathway inhibitor 2
**209719_x_at**	3.9	6.15	0.23	1.23	-4.74	0.0448	SERPINB3	serpin peptidase inhibitor, clade B (ovalbumin), member 3
**218824_at**	4.66	6.92	0.98	0.45	-4.77	0.0108	PNMAL1	paraneoplastic Ma antigen family-like 1
**202411_at**	6.74	9	0.24	0.98	-4.79	0.0025	IFI27	interferon, alpha-inducible protein 27
**204597_x_at**	7.93	10.19	0.38	0.44	-4.8	0.0002	STC1	stanniocalcin 1
**227566_at**	5.23	7.5	1.12	0.34	-4.82	0.0281	NTM	neurotrimin
**201261_x_at**	5.98	8.26	0.64	0.54	-4.86	0.0008	BGN	biglycan
**202887_s_at**	8.59	10.87	0.29	0.91	-4.87	0.0011	DDIT4	DNA damage inducible transcript 4

**41469_at**	4.15	6.46	0.25	1.78	-4.97	0.0379	PI3	peptidase inhibitor 3, skin-derived
**214974_x_at**	4.54	6.85	0.41	1.51	-4.98	0.0135	CXCL5	chemokine (C-X-C motif) ligand 5
**204464_s_at**	5.24	7.57	0.49	1.13	-5.01	0.002	EDNRA	endothelin receptor type A
**203475_at**	5.23	7.56	0.31	0.84	-5.02	0.0012	CYP19A1	cytochrome P450, family 19, subfamily A, polypeptide 1
**209616_s_at**	5.42	7.75	0.13	0.27	-5.02	3.74E-06	CES1; CES1P1	carboxylesterase 1; carboxylesterase 1 pseudogene 1
**33322_i_at**	7.65	9.99	0.09	1.94	-5.03	0.0383	SFN	stratifin
**205021_s_at**	6.02	8.35	0.64	0.37	-5.04	0.0016	FOXN3	forkhead box N3
**218002_s_at**	4.53	6.87	0.1	1.44	-5.06	0.0101	CXCL14	chemokine (C-X-C motif) ligand 14
**203691_at**	4.82	7.17	0.1	1.78	-5.08	0.0484	PI3	peptidase inhibitor 3, skin-derived
**205098_at**	4.39	6.73	0.35	0.43	-5.09	0.0003	CCR1	chemokine (C-C motif) receptor 1
**227609_at**	5.8	8.15	0.42	0.52	-5.1	0.0004	EPSTI1	epithelial stromal interaction 1 (breast)
**201162_at**	9	11.35	0.91	0.67	-5.11	0.0012	IGFBP7	insulin like growth factor binding protein 7
**203895_at**	5.39	7.75	0.49	1.53	-5.11	0.0043	PLCB4	phospholipase C, beta 4
**203535_at**	3.89	6.25	0.13	1.23	-5.13	0.0224	S100A9	S100 calcium binding protein A9
**209774_x_at**	6.81	9.18	0.97	0.99	-5.17	0.0067	CXCL2	chemokine (C-X-C motif) ligand 2
**214954_at**	3.37	5.77	0.54	1.08	-5.29	0.0331	SUSD5	sushi domain containing 5
**222450_at**	5.58	7.99	0.26	0.39	-5.3	2.12E-05	PMEPA1	prostate transmembrane protein, androgen induced 1
**215813_s_at**	5.61	8.03	0.59	1.26	-5.34	0.0062	PTGS1	prostaglandin-endoperoxide synthase 1 (prostaglandin G/H synthase and cyclooxygenase)
**221009_s_at**	5.95	8.37	0.47	1.3	-5.36	0.0344	ANGPTL4	angiopoietin like 4
**211527_x_at**	5.44	7.87	0.13	1.58	-5.37	0.0197	VEGFA	vascular endothelial growth factor A
**211564_s_at**	6.78	9.21	0.69	0.82	-5.38	0.0079	PDLIM4	PDZ and LIM domain 4
**226804_at**	6.12	8.56	0.96	0.8	-5.4	0.0077	FAM20A	family with sequence similarity 20, member A
**206039_at**	6.22	8.66	1.17	0.33	-5.45	0.0166	RAB33A	RAB33A, member RAS oncogene family
**228143_at**	4.84	7.29	0.52	1.22	-5.46	0.0495	CP	ceruloplasmin (ferroxidase)
**235944_at**	4.24	6.7	1.48	1.73	-5.48	0.0418	HMCN1	hemicentin 1
**210512_s_at**	7.7	10.17	0.34	1.28	-5.54	0.0057	VEGFA	vascular endothelial growth factor A
**202086_at**	6.95	9.42	1.31	1	-5.57	0.0299	MX1	MX dynamin-like GTPase 1
**209081_s_at**	5.85	8.34	0.64	0.94	-5.63	0.0066	COL18A1	collagen, type XVIII, alpha 1

**1554163_at**	6.24	8.75	0.82	0.67	-5.67	0.002	TWIST2	twist family bHLH transcription factor 2
**202890_at**	5.41	7.92	0.95	0.88	-5.71	0.0107	MAP7	microtubule-associated protein 7
**235301_at**	6.47	8.99	1.66	0.79	-5.73	0.0332	KIAA1324L	KIAA1324-like
**207030_s_at**	8.57	11.15	1.1	1.33	-5.96	0.0357	CSRP2	cysteine and glycine-rich protein 2
**210195_s_at**	8.35	10.93	1.21	0.15	-5.98	0.0028	PSG1	pregnancy specific beta-1-glycoprotein 1
**227337_at**	5.99	8.58	0.47	1.24	-6.02	0.0083	ANKRD37	ankyrin repeat domain 37
**213905_x_at**	5.41	8.02	0.66	0.58	-6.1	0.0006	BGN	biglycan
**211126_s_at**	7.83	10.45	0.9	1.36	-6.14	0.0288	CSRP2	cysteine and glycine-rich protein 2
**225288_at**	4.36	6.98	0.29	1.05	-6.14	0.002	COL27A1	collagen, type XXVII, alpha 1
**1554997_a_at**	5.96	8.6	1.98	0.99	-6.24	0.0102	PTGS2	prostaglandin-endoperoxide synthase 2 (prostaglandin G/H synthase and cyclooxygenase)
**225895_at**	6.64	9.29	0.44	1.52	-6.26	0.0052	SYNPO2	synaptopodin 2
**1555673_at**	3.8	6.45	0.24	2.03	-6.27	0.0218	KRTAP2-3	keratin associated protein 2-3
**1554195_a_at**	4.85	7.52	0.12	1.57	-6.38	0.0497	C5orf46	chromosome 5 open reading frame 46
**205990_s_at**	6.71	9.42	1.2	1.12	-6.53	0.026	WNT5A	wingless-type MMTV integration site family, member 5A
**218974_at**	4.84	7.57	1.04	1.26	-6.61	0.0402	SOBP	sine oculis binding protein homolog
**1554452_a_at**	6.56	9.29	0.57	1.58	-6.61	0.0212	HILPDA	hypoxia inducible lipid droplet-associated
**224724_at**	5.65	8.4	1.28	0.29	-6.71	0.0145	SULF2	sulfatase 2
**210196_s_at**	7.65	10.42	1.25	0.39	-6.84	0.0028	PSG1	pregnancy specific beta-1-glycoprotein 1
**204475_at**	11	13.78	1.33	0.19	-6.88	0.0118	MMP1	matrix metallopeptidase 1
**230710_at**	5.1	7.95	0.3	1.69	-7.17	0.0277	MIR210HG	MIR210 host gene
**202709_at**	6.9	9.75	1.79	0.94	-7.21	0.0478	FMOD	fibromodulin
**201250_s_at**	7.03	9.88	0.35	1.28	-7.24	0.0048	SLC2A1	solute carrier family 2 (facilitated glucose transporter), member 1
**204197_s_at**	4.59	7.5	0.26	0.88	-7.5	0.0009	RUNX3	runt-related transcription factor 3
**213425_at**	5.53	8.46	0.99	1.03	-7.62	0.0324	WNT5A	wingless-type MMTV integration site family, member 5A
**204596_s_at**	6.04	9	0.45	1.02	-7.77	0.0011	STC1	stanniocalcin 1
**201650_at**	3.94	6.9	0.04	1.71	-7.8	0.0291	KRT19	keratin 19, type I
**204595_s_at**	7.59	10.57	0.55	0.78	-7.92	0.0005	STC1	stanniocalcin 1
**218723_s_at**	7.29	10.28	0.43	0.86	-7.97	0.0001	RGCC	regulator of cell cycle

**202620_s_at**	7.68	10.69	0.99	1.38	-8.07	0.0279	PLOD2	procollagen-lysine, 2-oxoglutarate 5-dioxygenase 2
**229459_at**	4.34	7.39	0.37	0.74	-8.29	0.0005	FAM19A5	family with sequence similarity 19 (chemokine (C-C motif)-like), member A5
**213994_s_at**	4.93	7.98	1.47	1.62	-8.31	0.0473	SPON1	spondin 1, extracellular matrix protein
**230360_at**	6.28	9.35	1.62	0.84	-8.44	0.0073	GLDN	gliomedin
**224341_x_at**	3.92	7.02	1.55	0.48	-8.57	0.0233	TLR4	toll-like receptor 4
**213524_s_at**	5.82	8.92	0.75	1.27	-8.59	0.0024	G0S2	G0/G1 switch 2
**222108_at**	6.65	9.78	0.79	1.61	-8.78	0.0056	AMIGO2	adhesion molecule with Ig-like domain 2
**206569_at**	4.42	7.56	0.18	0.82	-8.79	0.0002	IL24	interleukin 24
**204748_at**	7.82	10.98	2.09	0.88	-8.96	0.0088	PTGS2	prostaglandin-endoperoxide synthase 2 (prostaglandin G/H synthase and cyclooxygenase)
**221577_x_at**	7.46	10.63	1.03	0.45	-9.03	0.0017	GDF15	growth differentiation factor 15
**204198_s_at**	3.87	7.05	0.04	1.21	-9.04	0.0014	RUNX3	runt-related transcription factor 3
**205943_at**	3.57	6.76	0.06	1.95	-9.14	0.0079	TDO2	tryptophan 2,3-dioxygenase
**205204_at**	9.35	12.59	0.38	1.01	-9.44	0.0011	NMB	neuromedin B
**226899_at**	4.5	7.74	0.66	0.88	-9.47	0.0012	UNC5B	unc-5 netrin receptor B
**202464_s_at**	6.35	9.63	1.13	0.83	-9.69	0.005	PFKFB3	6-phosphofructo-2-kinase/fructose-2,6-biphosphatase 3
**209652_s_at**	5.21	8.6	0.66	0.96	-10.43	0.0004	PGF	placental growth factor
**213479_at**	3.85	7.23	0.23	0.43	-10.45	4.98E-06	NPTX2	neuronal pentraxin II
**204470_at**	6.72	10.17	1.22	1.25	-10.95	0.0032	CXCL1	chemokine (C-X-C motif) ligand 1 (melanoma growth stimulating activity, alpha)
**205476_at**	3.98	7.54	0.33	1.42	-11.77	0.0005	CCL20	chemokine (C-C motif) ligand 20
**203786_s_at**	5.05	8.66	0.16	2.1	-12.19	0.0425	TPD52L1	tumor protein D52-like 1
**206932_at**	5.59	9.23	1.39	0.91	-12.47	0.0061	CH25H	cholesterol 25-hydroxylase
**235666_at**	4.79	8.44	0.12	1.62	-12.51	0.0092	ITGA8	integrin alpha 8
**207092_at**	4.57	8.26	0.21	1.87	-12.96	0.0173	LEP	leptin
**204971_at**	4.68	8.4	0.57	0.75	-13.16	0.0001	CSTA	cystatin A (stefin A)
**206026_s_at**	8.43	12.2	0.98	0.95	-13.59	0.0006	TNFAIP6	tumor necrosis factor, alpha-induced protein 6
**206290_s_at**	4.31	8.13	1.04	0.47	-14.13	0.0008	RGS7	regulator of G-protein signaling 7
**226632_at**	4.9	8.76	1.9	0.77	-14.54	0.0221	CYGB	cytoglobin
**212657_s_at**	4.79	8.68	0.67	0.72	-14.77	0.0003	IL1RN	interleukin 1 receptor antagonist

**205713_s_at**	5.54	9.43	0.18	2.23	-14.8	0.037	COMP	cartilage oligomeric matrix protein
**223710_at**	5.13	9.05	0.74	1.69	-15.18	0.0201	CCL26	chemokine (C-C motif) ligand 26
**228885_at**	3.56	7.5	0.42	1.79	-15.39	0.0163	MAMDC2	MAM domain containing 2
**205064_at**	4.03	7.99	0.1	2.36	-15.56	0.046	SPRR1B	small proline-rich protein 1B
**206025_s_at**	8.34	12.37	0.85	0.78	-16.3	0.0001	TNFAIP6	tumor necrosis factor, alpha-induced protein 6
**202149_at**	3.97	8.14	1.43	0.3	-17.96	0.0016	NEDD9	neural precursor cell expressed, developmentally down-regulated 9
**226702_at**	5.9	10.11	0.58	0.63	-18.52	3.52E-05	CMPK2	cytidine monophosphate (UMP-CMP) kinase 2, mitochondrial
**219825_at**	3.96	8.18	1.32	0.96	-18.59	0.0024	CYP26B1	cytochrome P450, family 26, subfamily B, polypeptide 1
**205207_at**	7.89	12.24	1.62	1.62	-20.39	0.0068	IL6	interleukin 6
**207850_at**	4.84	9.32	0.23	1.36	-22.32	0.0003	CXCL3	chemokine (C-X-C motif) ligand 3
**213797_at**	4.24	8.76	0.36	1.52	-22.94	0.0013	RSAD2	radical S-adenosyl methionine domain containing 2
**223484_at**	5.36	9.92	1.51	0.24	-23.63	0.0017	C15orf48	chromosome 15 open reading frame 48
**202855_s_at**	5.45	10.09	0.46	1.88	-24.9	0.0054	SLC16A3	solute carrier family 16 (monocarboxylate transporter), member 3
**227742_at**	4.65	9.33	0.7	0.86	-25.61	0.0002	CLIC6	chloride intracellular channel 6
**1555778_a_at**	4.63	9.35	1.38	0.86	-26.35	0.0005	POSTN	periostin, osteoblast specific factor
**223121_s_at**	4.72	9.45	2.48	1.11	-26.53	0.0365	SFRP2	secreted frizzled-related protein 2
**210809_s_at**	6.03	10.86	2.12	0.73	-28.3	0.002	POSTN	periostin, osteoblast specific factor
**209351_at**	4.02	8.86	0.02	4.01	-28.77	0.0416	KRT14	keratin 14, type I
**203477_at**	4.24	9.1	0.89	0.9	-29.07	0.0002	COL15A1	collagen, type XV, alpha 1
**231240_at**	4.4	9.42	1.89	1.1	-32.33	0.0139	DIO2	deiodinase, iodothyronine, type II
**223122_s_at**	5.93	11	2.77	1.1	-33.66	0.0407	SFRP2	secreted frizzled-related protein 2
**213680_at**	4.05	9.23	0.11	3.76	-36.18	0.0411	KRT6B	keratin 6B, type II
**205916_at**	4.03	9.24	0.02	3.05	-36.98	0.042	S100A7	S100 calcium binding protein A7
**202856_s_at**	5.12	10.35	0.77	1.78	-37.44	0.0044	SLC16A3	solute carrier family 16 (monocarboxylate transporter), member 3
**205828_at**	8.15	13.38	2.45	0.18	-37.49	0.0125	MMP3	matrix metallopeptidase 3

**242625_at**	3.72	9.45	0.53	1.31	-52.99	0.0002	RSAD2	radical S-adenosyl methionine domain containing 2
**202859_x_at**	6.57	12.71	0.09	0.56	-70.81	2.35E-07	CXCL8	chemokine (C-X-C motif) ligand 8
**208650_s_at**	3.22	9.47	0.08	3.71	-76.35	0.0431	CD24	CD24 molecule
**209125_at**	3.49	10.52	0.23	4.25	-130.54	0.0453	KRT6A	keratin 6A, type II
**211506_s_at**	5.6	12.94	0.21	0.8	-161.96	8.66E-07	CXCL8	chemokine (C-X-C motif) ligand 8

Supplementary Table 2. Differentially-regulated genes in SS lymphoblastoid cells

Affymetrix ID	Setleis Avg (log2)	WT Avg (log2)	Setleis Std Dev	WT Std Dev	Fold Change	P-val	Gene Symbol	Description
**3555088**	5.09	8.73	0.15	0.65	-12.47	0.0001	KIAA0125	KIAA0125
**2940202**	4.84	8.22	1.83	0.47	-10.37	0.0293	F13A1	coagulation factor XIII, A1 polypeptide
**3538893**	5.43	8.63	0.58	0.72	-9.19	0.0006	PRKCH	protein kinase C, eta
**3982811**	5.82	8.92	1.43	0.44	-8.61	0.0244	SH3BGRL	SH3 domain binding glutamate-rich protein like
**2563785**	7.74	10.78	0.5	0.67	-8.24	0.0003	IGKV2-29; IGKV2-26; IGKC; IGK; IGKV1-5; IGKV7-3; IGKV2-4; IGKV1-6; IGKV3D-34; IGKV1D-33; IGKV2D-28; IGKV3-11; IGKV1-8; IGKV2-10; IGKV3-7; IGKV1-9; IGKV2-23; IGKV3-20; IGKV1-27; IGKV2-14; IGKV1-12; IGKV2-18; IGKV1-22; IGKV1-13; IGKV1-16; IGKV1-17; IGKV2-19; IGKV2-28; IGKV3-15; IGKV6-21; IGKV2-24; IGKJ5; IGKV1-39; IGKV1-33; IGKV3-31; IGKV1-37; IGKV2-30; IGKV3-34; IGKV1D-37; IGKV1D-39; IGKV2-40; IGKV1D-13;	immunoglobulin kappa variable 2-29, 2-26 immunoglobulin kappa constant; immunoglobulin kappa locus; immunoglobulin kappa variable 1-5; 7-3, 2-, 1-6; 3D-34, 1D-33; 2D-28; 3-11; 1-8; 2-10, 3-7 (non-functional); 1-9; 2-23, 3-20; 1-27; 2-14, 1-12; 2-18, 1-22 1-13, 1-16; 1-17; 2-19, 2-28; 3-15; 6-21 (non-functional); 2-24; immunoglobulin kappa joining 5; immunoglobulin kappa variable 1-39, 1-33; 3-31, 1-37 (non-functional); 2-30; 3-34 , 1D-37 (non-functional); 1D-39; 2-40; 1D-13; 1-35, 1D-12; 3D-15, 1D-8

							IGKV1-35; IGKV1D-12; IGKV3D-15; IGKV1D-8	
**3185205**	5.95	8.98	1.44	1.26	-8.13	0.0469	HSDL2	hydroxysteroid dehydrogenase like 2
**3203382**	4.83	7.8	2.15	1.32	-7.83	0.0399	SMU1	smu-1 suppressor of mec-8 and unc-52 homolog (C. elegans)
**3781980**	5.34	8.29	1.18	1	-7.74	0.0413	TTC39C	tetratricopeptide repeat domain 39C
**3925639**	4.04	6.89	0.15	0.62	-7.2	0.0003	NRIP1	nuclear receptor interacting protein 1
**3444525**	1.93	4.68	1.18	0.84	-6.76	0.0254	TAS2R46	taste receptor, type 2, member 46
**3447694**	5.94	8.69	1.11	1.07	-6.75	0.0339	BCAT1	branched chain amino-acid transaminase 1, cytosolic
**3156307**	5.18	7.9	0.4	0.97	-6.6	0.0048	PTK2	protein tyrosine kinase 2
**2651835**	3.34	6.03	1.08	0.84	-6.47	0.0229	GPR160	G protein-coupled receptor 160
**3374402**	6.89	9.56	1.6	0.14	-6.39	0.0105	LPXN	leupaxin
**3444472**	4.09	6.76	0.49	0.92	-6.35	0.0057	TAS2R50	taste receptor, type 2, member 50
**3277751**	4.8	7.43	1.1	0.71	-6.18	0.0479	NUDT5	nudix hydrolase 5
**3513514**	3.79	6.37	0.38	0.89	-5.96	0.0057	LPAR6	lysophosphatidic acid receptor 6
**3689922**	5.93	8.5	1.37	0.91	-5.94	0.0439	VPS35	VPS35 retromer complex component
**3257338**	3.75	6.31	0.9	0.81	-5.92	0.0368	KIF20B	kinesin family member 20B
**3778504**	6.24	8.8	0.69	0.46	-5.9	0.0011	RAB31	RAB31, member RAS oncogene family
**3301218**	7.96	10.51	0.95	0.78	-5.88	0.0422	PDLIM1	PDZ and LIM domain 1
**3224591**	5.2	7.76	0.92	0.97	-5.86	0.0427	STRBP	spermatid perinuclear RNA binding protein
**3909395**	5.55	8.06	1.2	0.57	-5.7	0.0316	DPM1	dolichyl-phosphate mannosyltransferase polypeptide 1, catalytic subunit
**3800619**	5.72	8.23	1.52	0.77	-5.69	0.0467	ROCK1	Rho-associated, coiled-coil containing protein kinase 1
**3743486**	6.81	9.3	1.74	0.75	-5.65	0.0336	GABARAP	GABA(A) receptor-associated protein
**3991889**	2.9	5.39	0.53	0.62	-5.62	0.0005	FAM127A	family with sequence similarity 127, member A
**3942954**	5.06	7.55	1.04	0.7	-5.61	0.0117	DRG1	developmentally regulated GTP binding protein 1
**3252170**	5.3	7.79	0.99	0.44	-5.61	0.021	ADK; MRPL35P3	adenosine kinase; mitochondrial ribosomal protein L35 pseudogene 3

**3429857**	6.05	8.54	1.22	0.01	-5.6	0.0256	C12orf75	chromosome 12 open reading frame 75
**3513549**	6.21	8.69	1.06	0.42	-5.56	0.0277	RCBTB2	regulator of chromosome condensation (RCC1) and BTB (POZ) domain containing protein 2
**3265133**	5.09	7.56	1.07	0.51	-5.54	0.038	NHLRC2	NHL repeat containing 2
**3449700**	6.04	8.5	1.96	0.22	-5.53	0.0374	FAM60A	family with sequence similarity 60, member A
**3843662**	5.29	7.76	1.12	0.13	-5.52	0.0098	ZNF587	zinc finger protein 587
**2363424**	6.2	8.66	1.41	0.27	-5.5	0.0371	UFC1	ubiquitin-fold modifier conjugating enzyme 1
**3396593**	4.73	7.17	0.28	1.01	-5.43	0.0176	FEZ1	fasciculation and elongation protein zeta 1
**2820925**	4.51	6.95	0.71	0.81	-5.42	0.0075	RHOBTB3	Rho-related BTB domain containing 3
**3145953**	8.84	11.27	1.25	0.29	-5.39	0.0143	RPL30; SNORA72	ribosomal protein L30; small nucleolar RNA, H/ACA box 72
**3277468**	6.46	8.88	1.41	0.45	-5.37	0.0153	USP6NL	USP6 N-terminal like
**3729294**	5.79	8.21	1.38	0.84	-5.35	0.0375	RPS6KB1	ribosomal protein S6 kinase, 70kDa, polypeptide 1
**3623424**	6.33	8.75	1.63	0.61	-5.33	0.0486	COPS2	COP9 signalosome subunit 2
**3125001**	4.85	7.27	1.17	0.83	-5.33	0.0349	LONRF1	LON peptidase N-terminal domain and ring finger 1
**3996815**	5.07	7.47	0.92	0.89	-5.26	0.0284	VBP1	von Hippel-Lindau binding protein 1
**3944046**	5.71	8.09	0.93	0.7	-5.22	0.0451	HMGXB4	HMG box domain containing 4
**3505319**	6.52	8.9	1.02	0.48	-5.22	0.0081	SACS	sacsin molecular chaperone
**3486383**	5.25	7.63	1.33	0.77	-5.18	0.0449	COG6	component of oligomeric golgi complex 6
**2935475**	5.85	8.21	0.93	0.54	-5.13	0.0292	QKI	QKI, KH domain containing, RNA binding
**3134922**	6.12	8.47	1.44	0.56	-5.12	0.0335	PCMTD1; PXDNL	protein-L-isoaspartate (D-aspartate) O-methyltransferase domain containing 1; peroxidasin like
**2421883**	4.87	7.22	1.18	0.61	-5.09	0.0371	GBP1	guanylate binding protein 1, interferon-inducible
**3591006**	6.61	8.96	1.28	0.38	-5.08	0.0174	SNAP23	synaptosome associated protein 23kDa
**3684100**	6.99	9.33	1.04	0.51	-5.08	0.0055	NPIPB4; NPIPB3; SLC7A5P2; SMG1P3; NPIPB5; SMG1P1	nuclear pore complex interacting protein family, member B4; nuclear pore complex interacting protein family, member B3; solute carrier family 7 (amino acid transporter light chain, L

								system), member 5 pseudogene 2; SMG1 pseudogene 3; nuclear pore complex interacting protein family, member B5; SMG1 pseudogene 1
**3364759**	7.13	9.47	1.64	0.98	-5.08	0.0391	PIK3C2A	phosphatidylinositol-4-phosphate 3-kinase, catalytic subunit type 2 alpha
**3336402**	5.78	8.12	1.11	0.53	-5.05	0.0152	RBM14	RNA binding motif protein 14
**3625271**	5.43	7.76	0.69	0.93	-5.05	0.0499	RAB27A	RAB27A, member RAS oncogene family
**2368180**	7.39	9.73	0.73	0.48	-5.04	0.0104	GPR52	G protein-coupled receptor 52
**3079103**	5.64	7.98	0.67	0.97	-5.04	0.0137	GIMAP6	GTPase, IMAP family member 6
**3485074**	6.63	8.96	1.47	0.81	-5.02	0.046	RFC3	replication factor C subunit 3
**3504791**	5.11	7.43	1.49	0.56	-4.99	0.038	MICU2; RNU6-59P	mitochondrial calcium uptake 2; RNA, U6 small nuclear 59, pseudogene
**2545653**	7.04	9.35	1.31	0.26	-4.96	0.0411	MPV17	MpV17 mitochondrial inner membrane protein
**3417161**	6.38	8.69	1.42	0.49	-4.96	0.0406	RAB5B	RAB5B, member RAS oncogene family
**3352070**	6.87	9.18	1.39	0.33	-4.95	0.0236	CBL	Cbl proto-oncogene, E3 ubiquitin protein ligase
**3282016**	6.66	8.97	1.52	0.57	-4.94	0.0337	ABI1	abl-interactor 1
**3535628**	6.41	8.71	0.77	0.4	-4.94	0.0105	GNG2	guanine nucleotide binding protein (G protein), gamma 2
**3555067**	6.02	8.31	0.51	0.43	-4.89	0.0004	KIAA0125	KIAA0125
**3325307**	4.75	7.04	0.65	0.63	-4.88	0.0156	ELP4	elongator acetyltransferase complex subunit 4
**3428845**	3.15	5.44	0.79	0.6	-4.87	0.012	PARPBP	PARP1 binding protein
**3717539**	5.3	7.58	0.99	0.91	-4.87	0.0366	RHOT1	ras homolog family member T1
**3337168**	8.58	10.86	1.73	0.26	-4.86	0.0425	GSTP1	glutathione S-transferase pi 1
**3326540**	5.59	7.86	1.04	0.8	-4.83	0.0447	PDHX; MIR1343	pyruvate dehydrogenase complex, component X; microRNA 1343
**3237548**	6.87	9.13	1.05	0.02	-4.79	0.0072	ARL5B	ADP-ribosylation factor like GTPase 5B
**4023467**	5.78	8.03	1.21	0.79	-4.77	0.0467	ARHGEF6	Rac/Cdc42 guanine nucleotide exchange factor 6
**3512948**	6.19	8.44	0.72	0.37	-4.75	0.0011	KIAA0226L	KIAA0226-like
**2356115**	8.49	10.72	1.25	0.59	-4.68	0.0275	TXNIP	thioredoxin interacting protein

**4044363**	6.02	8.23	1.13	0.15	-4.64	0.0114	CNR2	cannabinoid receptor 2
**4019486**	7.79	10	1.83	0.18	-4.63	0.0442	6-Sep	septin 6
**3431376**	7.46	9.67	1.63	0.62	-4.62	0.0399	ANKRD13A	ankyrin repeat domain 13A
**3840142**	3.74	5.95	0.58	0.54	-4.61	0.0037	ZNF480	zinc finger protein 480
**3406493**	5.43	7.63	0.71	0.71	-4.58	0.027	DERA	deoxyribose-phosphate aldolase (putative)
**3537747**	5.89	8.08	1.12	0.51	-4.58	0.0206	PSMA3	proteasome subunit alpha 3
**3205162**	5.95	8.15	1.08	0.14	-4.57	0.0111	RNF38	ring finger protein 38
**3687494**	6.12	8.31	0.98	0.43	-4.57	0.0384	MAPK3	mitogen-activated protein kinase 3
**3483159**	6.65	8.84	1.24	0.32	-4.57	0.0134	PAN3; RNU6-82P	PAN3 poly(A) specific ribonuclease subunit; RNA, U6 small nuclear 82, pseudogene
**3307939**	5.27	7.46	0.72	0.79	-4.56	0.0167	ABLIM1	actin binding LIM protein 1
**3448428**	5.81	8	1.23	0.65	-4.56	0.0342	ASUN	asunder spermatogenesis regulator
**3386217**	2.62	4.8	0.63	1.31	-4.56	0.0389	CHORDC1	cysteine and histidine rich domain containing 1
**2645275**	5.72	7.91	1.49	0.64	-4.54	0.0363	SLC25A36	solute carrier family 25 (pyrimidine nucleotide carrier), member 36
**2484358**	7.01	9.19	1.35	0.41	-4.53	0.0256	REL	v-rel avian reticuloendotheliosis viral oncogene homolog
**2704894**	7.54	9.72	1.35	0.32	-4.53	0.042	PHC3	polyhomeotic homolog 3 (Drosophila)
**3638665**	5.43	7.61	0.83	0.6	-4.52	0.039	C15orf38-AP3S2; AP3S2	C15orf38-AP3S2 readthrough; adaptor-related protein complex 3, sigma 2 subunit
**2473376**	5.75	7.92	0.38	0.98	-4.52	0.0195	EFR3B; DNAJC27-AS1	EFR3 homolog B; DNAJC27 antisense RNA 1
**3594986**	4.71	6.89	0.84	0.46	-4.51	0.0041	TEX9	testis expressed 9
**3347549**	4.85	7.02	0.74	0.82	-4.5	0.0324	CUL5	cullin 5
**2964231**	6.8	8.97	1.32	1.14	-4.5	0.0468	RRAGD	Ras-related GTP binding D
**2346074**	4.45	6.62	1.04	0.42	-4.5	0.0325	ZNF326	zinc finger protein 326
**3421897**	6.31	8.47	1.16	0.27	-4.49	0.014	CNOT2	CCR4-NOT transcription complex subunit 2
**3761164**	6.54	8.71	1.15	0.83	-4.48	0.0432	SKAP1	src kinase associated phosphoprotein 1
**3421446**	7.16	9.32	1.38	0.6	-4.47	0.0478	CPSF6	cleavage and polyadenylation specific factor 6
**3540353**	4.02	6.18	1.53	0.5	-4.47	0.0361	CHURC1-FNTB; CHURC1	CHURC1-FNTB readthrough; churchill domain containing 1

**4022106**	6.04	8.2	1.13	0.45	-4.45	0.0382	MBNL3	muscleblind-like splicing regulator 3
**3513883**	6.44	8.59	1.33	0.59	-4.44	0.045	KPNA3	karyopherin alpha 3 (importin alpha 4)
**3310757**	5.37	7.52	1.39	0.41	-4.43	0.0343	IKZF5	IKAROS family zinc finger 5
**3368748**	4.92	7.07	1.03	0.34	-4.43	0.0422	FBXO3	F-box protein 3
**3561952**	6.6	8.75	1.1	0.66	-4.42	0.0306	SEC23A	Sec23 homolog A, COPII coat complex component
**3303165**	6.56	8.7	1.23	0.45	-4.41	0.0168	DNMBP	dynamin binding protein
**3514488**	5.96	8.1	1.08	0.33	-4.41	0.0128	INTS6	integrator complex subunit 6
**3241316**	6.02	8.15	1.11	0.32	-4.4	0.0133	ZEB1	zinc finger E-box binding homeobox 1
**3437500**	6.19	8.33	0.46	0.81	-4.4	0.024	GLT1D1	glycosyltransferase 1 domain containing 1
**3244622**	7.4	9.53	1.05	0.31	-4.4	0.0084	ALOX5	arachidonate 5-lipoxygenase
**2509988**	5.95	8.09	0.9	0.73	-4.39	0.0359	LYPD6B	LY6/PLAUR domain containing 6B
**2991233**	5.16	7.3	0.94	0.45	-4.39	0.0194	AHR	aryl hydrocarbon receptor
**3195344**	5.34	7.47	1.38	0.14	-4.38	0.0185	MRPL41	mitochondrial ribosomal protein L41
**3763270**	6.77	8.9	0.92	0.24	-4.37	0.0037	MMD	monocyte to macrophage differentiation-associated
**2864849**	6.95	9.08	1.24	0.14	-4.36	0.0233	SSBP2	single-stranded DNA binding protein 2
**3362263**	6.5	8.61	1.19	0.7	-4.34	0.028	DENND5A	DENN/MADD domain containing 5A
**3948754**	7.64	9.75	1.24	0.54	-4.33	0.0279	ATXN10	ataxin 10
**3444476**	5.54	7.65	1.23	0.72	-4.32	0.0236	TAS2R20; TAS2R19	taste receptor, type 2, member 20; taste receptor, type 2, member 19
**3917155**	5.93	8.04	0.94	0.55	-4.32	0.0164	USP16	ubiquitin specific peptidase 16
**3942161**	5.88	7.99	1.06	0.39	-4.31	0.0359	UQCR10	ubiquinol-cytochrome c reductase, complex III subunit X
**3199431**	6.32	8.43	0.99	0.11	-4.31	0.0059	ZDHHC21	zinc finger, DHHC-type containing 21
**2663083**	4.87	6.97	0.78	0.41	-4.3	0.0092	TAMM41	TAM41 mitochondrial translocator assembly and maintenance homolog
**3717635**	7.95	10.05	1.47	0.32	-4.29	0.0206	ZNF207; MIR632	zinc finger protein 207; microRNA 632
**3475717**	5.99	8.09	1.34	0.45	-4.28	0.0245	RSRC2	arginine/serine-rich coiled-coil 2
**3445786**	8.42	10.52	1.57	0.32	-4.28	0.0302	ARHGDIB	Rho GDP dissociation inhibitor (GDI) beta
**3528115**	6.88	8.97	1.04	0.26	-4.28	0.0113	TOX4	TOX high mobility group box family member 4

**3636522**	5.98	8.07	0.5	0.56	-4.26	0.0042	HDGFRP3	hepatoma-derived growth factor, related protein 3
**3383322**	6.04	8.11	1.28	0.33	-4.22	0.027	NARS2	asparaginyl-tRNA synthetase 2, mitochondrial (putative)
**3537884**	4.66	6.73	0.95	0.41	-4.21	0.0123	ARID4A	AT rich interactive domain 4A (RBP1-like)
**3840372**	4.02	6.1	0.59	0.89	-4.2	0.0301	ZNF701	zinc finger protein 701
**2672096**	3.65	5.72	0.5	1.21	-4.2	0.0286	CCR1	chemokine (C-C motif) receptor 1
**3972025**	5.4	7.47	0.83	0.82	-4.18	0.0403	PDK3	pyruvate dehydrogenase kinase, isozyme 3
**3629350**	7.4	9.46	1.12	0.62	-4.17	0.0282	SPG21	spastic paraplegia 21 (autosomal recessive, Mast syndrome)
**2530539**	6.36	8.42	0.99	0.24	-4.17	0.0379	MFF	mitochondrial fission factor
**4002011**	3.97	6.03	0.78	0.38	-4.16	0.0097	CXorf23	chromosome X open reading frame 23
**2403335**	5.74	7.79	0.97	0.38	-4.16	0.0248	EYA3	EYA transcriptional coactivator and phosphatase 3
**3136178**	5.74	7.79	0.3	0.42	-4.14	0.0015	PLAG1	pleiomorphic adenoma gene 1
**2992963**	5.2	7.25	0.72	0.71	-4.13	0.0222	CCDC126	coiled-coil domain containing 126
**3138464**	6.81	8.86	0.69	0.68	-4.12	0.0057	PDE7A	phosphodiesterase 7A
**3631794**	5.23	7.27	0.98	0.75	-4.12	0.0397	MYO9A	myosin IXA
**3335089**	6.14	8.18	0.99	0.37	-4.12	0.036	DPF2	D4, zinc and double PHD fingers family 2
**3449910**	5.39	7.43	0.94	0.8	-4.11	0.0463	AMN1; STMN1P1	antagonist of mitotic exit network 1 homolog; stathmin 1 pseudogene 1
**3449760**	5.32	7.36	0.49	0.64	-4.1	0.0035	DENND5B	DENN/MADD domain containing 5B
**3627076**	6.03	8.07	1.29	0.85	-4.1	0.0422	BNIP2	BCL2/adenovirus E1B 19kDa interacting protein 2
**2366581**	4.28	6.31	0.99	0.35	-4.1	0.0437	C1orf112	chromosome 1 open reading frame 112
**3642162**	4.82	6.85	0.63	0.48	-4.08	0.0051	SNRPA1	small nuclear ribonucleoprotein polypeptide A
**3954238**	7.54	9.57	1.22	0.41	-4.08	0.0322	MAPK1	mitogen-activated protein kinase 1
**3659931**	5.59	7.62	1.03	0.49	-4.08	0.0251	PAPD5	PAP associated domain containing 5
**3343546**	4.59	6.61	0.74	0.57	-4.07	0.0149	TMEM135	transmembrane protein 135
**3182019**	5.7	7.73	1.19	0.49	-4.07	0.0344	STX17	syntaxin 17
**3927105**	5.73	7.75	0.92	0.67	-4.06	0.0487	MRPL39	mitochondrial ribosomal protein L39
**3778601**	5.88	7.91	1.1	0.57	-4.06	0.0331	VAPA	VAMP associated protein A

**3593652**	5.28	7.3	1.05	0.47	-4.05	0.0231	USP8	ubiquitin specific peptidase 8
**3830353**	7.24	9.25	0.99	0.39	-4.03	0.01	CD22	CD22 molecule
**3864725**	4.17	6.17	0.8	0.53	-4.02	0.0353	ZNF45	zinc finger protein 45
**2881554**	6.02	8.02	1	0.22	-4.01	0.0322	DCTN4	dynactin 4 (p62)
**2819044**	6.61	8.61	1.4	0.25	-4.01	0.0359	RASA1	RAS p21 protein activator (GTPase activating protein) 1
**3462693**	5.62	7.62	1.09	0.61	-4.01	0.028	KRR1	KRR1, small subunit (SSU) processome component, homolog (yeast)
**3988874**	5.9	7.9	1.28	0.19	-4	0.0191	UBE2A	ubiquitin conjugating enzyme E2A
**3624145**	5.16	7.15	0.83	0.53	-3.99	0.008	DMXL2	Dmx-like 2
**3707199**	6.1	8.09	0.81	0.58	-3.99	0.0161	PSMB6	proteasome subunit beta 6
**3560575**	5.16	7.15	0.85	0.65	-3.98	0.0257	EAPP	E2F-associated phosphoprotein
**3191589**	5.46	7.45	0.88	0.69	-3.98	0.0441	FUBP3	far upstream element (FUSE) binding protein 3
**3591400**	6.18	8.17	1.24	0.38	-3.97	0.03	TUBGCP4	tubulin, gamma complex associated protein 4
**3321269**	5.99	7.98	1.15	0.64	-3.97	0.0458	FAR1	fatty acyl CoA reductase 1
**3573229**	4.83	6.81	0.65	0.39	-3.96	0.0126	ALKBH1	alkB homolog 1, histone H2A dioxygenase
**3409081**	6.89	8.88	1.18	0.27	-3.96	0.014	STK38L	serine/threonine kinase 38 like
**2320048**	6.33	8.32	1.2	0.69	-3.96	0.048	TARDBP	TAR DNA binding protein
**3320169**	6.54	8.53	0.63	0.57	-3.95	0.009	AMPD3	adenosine monophosphate deaminase 3
**3505937**	5.69	7.67	1.08	0.54	-3.95	0.0325	CENPJ	centromere protein J
**3548152**	6.6	8.58	1.25	0.46	-3.95	0.039	TDP1	tyrosyl-DNA phosphodiesterase 1
**3896370**	8.02	10	1.16	0.26	-3.95	0.0188	GPCPD1	glycerophosphocholine phosphodiesterase 1
**3504760**	4.1	6.07	0.77	0.81	-3.94	0.0168	ZDHHC20	zinc finger, DHHC-type containing 20
**3900091**	5.97	7.95	1	0.65	-3.94	0.0144	RALGAPA2	Ral GTPase activating protein, alpha subunit 2 (catalytic)
**3527418**	5.54	7.51	1.01	0.58	-3.94	0.0278	PARP2	poly(ADP-ribose) polymerase 2
**2350922**	5.43	7.41	0.91	0.12	-3.93	0.0247	GSTM4; GSTM2	glutathione S-transferase mu 4; glutathione S-transferase mu 2 (muscle)
**3452145**	5.61	7.58	1.31	0.26	-3.92	0.0221	SCAF11	SR-related CTD-associated factor 11
**3482219**	6.32	8.29	1.14	0.76	-3.92	0.0434	NUP58	nucleoporin 58kDa
**3765580**	5.62	7.59	1	0.63	-3.92	0.0429	BRIP1	BRCA1 interacting protein C-terminal helicase 1

**3269373**	4.18	6.14	0.76	0.33	-3.91	0.0056	ZRANB1	zinc finger, RAN-binding domain containing 1
**3329983**	5.67	7.63	0.48	0.61	-3.9	0.0069	OR4B1; PTPRJ; OR4B2P	olfactory receptor, family 4, subfamily B, member 1; protein tyrosine phosphatase, receptor type, J; olfactory receptor, family 4, subfamily B, member 2 pseudogene
**3628469**	3.19	5.15	0.63	0.91	-3.9	0.0368	RPS27L	ribosomal protein S27-like
**3423184**	5.41	7.37	0.57	0.43	-3.89	0.0043	ZDHHC17	zinc finger, DHHC-type containing 17
**3563734**	5.92	7.87	1.43	0.15	-3.88	0.0365	SOS2	SOS Ras/Rho guanine nucleotide exchange factor 2
**3456630**	7.79	9.75	1.43	0.29	-3.87	0.036	CBX5	chromobox homolog 5
**3445643**	4.94	6.9	0.77	0.56	-3.87	0.0148	HIST4H4	histone cluster 4, H4
**3960782**	6.56	8.51	1.03	0.63	-3.87	0.0371	JOSD1	Josephin domain containing 1
**3159040**	9.75	11.7	1.14	0.12	-3.86	0.0139	RPL8; MIR6850	ribosomal protein L8; microRNA 6850
**3400384**	7.78	9.73	0.9	0.56	-3.86	0.0168	WNK1	WNK lysine deficient protein kinase 1
**3576704**	4.74	6.69	0.33	0.41	-3.86	0.0012	TC2N; CATSPERB	tandem C2 domains, nuclear; catsper channel auxiliary subunit beta
**3489957**	5.91	7.85	0.79	0.74	-3.85	0.0411	RNASEH2B	ribonuclease H2, subunit B
**3733065**	4.88	6.82	0.22	1.48	-3.84	0.045	MAP2K6	mitogen-activated protein kinase kinase 6
**3570454**	5.83	7.77	0.79	0.14	-3.84	0.0098	SYNJ2BP-COX16; COX16	SYNJ2BP-COX16 readthrough; COX16 cytochrome c oxidase assembly homolog
**3707715**	6.19	8.13	1.39	0.02	-3.84	0.029	RPAIN	RPA interacting protein
**3429754**	6.97	8.9	1.44	0.31	-3.84	0.0435	KIAA1033	KIAA1033
**3291682**	7.02	8.95	1.53	0.32	-3.82	0.0436	JMJD1C	jumonji domain containing 1C
**3975467**	5.52	7.45	0.96	0.13	-3.82	0.0088	KDM6A	lysine (K)-specific demethylase 6A
**3984655**	5.51	7.44	0.82	0.83	-3.81	0.0481	CENPI	centromere protein I
**3468009**	5.24	7.17	1.26	0.48	-3.81	0.026	ARL1	ADP-ribosylation factor like GTPase 1
**2331727**	8.5	10.42	1.3	0.36	-3.8	0.0494	CAP1	CAP, adenylate cyclase-associated protein 1 (yeast)
**2478928**	5.55	7.48	0.88	0.3	-3.8	0.0225	MTA3	metastasis associated 1 family member 3
**3807370**	6.39	8.32	1.31	0.18	-3.8	0.034	DYM	dymeclin
**3282213**	7.61	9.53	1.45	0.53	-3.79	0.0405	YME1L1	YME1-like 1 ATPase
**3597603**	7.15	9.07	1.28	0.33	-3.78	0.0339	USP3	ubiquitin specific peptidase 3

**3249738**	6.42	8.33	1.18	0.29	-3.77	0.0193	HNRNPH3	heterogeneous nuclear ribonucleoprotein H3 (2H9)
**3599280**	7.67	9.58	1.02	0.44	-3.77	0.01	SKOR1; PIAS1	SKI family transcriptional corepressor 1; protein inhibitor of activated STAT 1
**3367183**	4.95	6.86	0.51	0.77	-3.76	0.0159	LIN7C	lin-7 homolog C (C. elegans)
**3430389**	5.38	7.29	0.66	0.48	-3.75	0.0101	TMEM263	transmembrane protein 263
**3412008**	6.06	7.96	1.03	0.46	-3.74	0.0294	PPHLN1	periphilin 1
**3421579**	5.49	7.39	1.08	0.11	-3.73	0.0118	FRS2	fibroblast growth factor receptor substrate 2
**3331903**	5.8	7.7	1.06	0.71	-3.73	0.0439	FAM111B	family with sequence similarity 111, member B
**3374746**	6.79	8.69	1.14	0.15	-3.73	0.016	PATL1	protein associated with topoisomerase II homolog 1 (yeast)
**3627363**	5.54	7.44	1.22	0.41	-3.72	0.0245	ICE2	interactor of little elongation complex ELL subunit 2
**3141857**	6.18	8.08	1.07	0.57	-3.72	0.0498	TPD52	tumor protein D52
**3197318**	7	8.9	0.82	0.36	-3.72	0.0258	AK3; CDC37L1-AS1	adenylate kinase 3; CDC37L1 antisense RNA 1 (head to head)
**3031573**	6.28	8.17	0.41	0.86	-3.72	0.0059	GIMAP1-GIMAP5; GIMAP5; GIMAP1	GIMAP1-GIMAP5 readthrough; GTPase, IMAP family member 5; GTPase, IMAP family member 1
**3353335**	5.39	7.28	0.06	0.83	-3.71	0.0166	UBASH3B	ubiquitin associated and SH3 domain containing B
**3136129**	8.83	10.72	1.06	0.44	-3.71	0.0443	RPS20; SNORD54	ribosomal protein S20; small nucleolar RNA, C/D box 54
**3834089**	7.48	9.36	1.51	0.26	-3.7	0.0439	HNRNPUL1	heterogeneous nuclear ribonucleoprotein U-like 1
**3661065**	5.71	7.59	0.86	0.77	-3.69	0.0447	RBL2	retinoblastoma-like 2
**3748323**	5.73	7.62	0.56	0.79	-3.69	0.0303	SHMT1	serine hydroxymethyltransferase 1 (soluble)
**3544905**	6.37	8.26	0.98	0.41	-3.69	0.0073	GPATCH2L	G-patch domain containing 2 like
**2414998**	6.67	8.55	1.2	0.48	-3.69	0.0295	MYSM1	Myb-like, SWIRM and MPN domains 1
**3532393**	5.77	7.65	1.02	0.58	-3.69	0.0412	KIAA0391; RPL9P3	KIAA0391; ribosomal protein L9 pseudogene 3
**3716481**	6.32	8.2	1.16	0.13	-3.66	0.0202	GOSR1; ALOX12P1	golgi SNAP receptor complex member 1; arachidonate 12-lipoxygenase pseudogene 1
**3371339**	6.79	8.66	1.12	0.32	-3.66	0.0206	PHF21A	PHD finger protein 21A
**3832643**	8.26	10.13	1.18	0.35	-3.66	0.0314	ACTN4	actinin, alpha 4
**3307795**	3.83	5.7	0.83	0.51	-3.66	0.0117	CCDC186; MIR2110	coiled-coil domain containing 186; microRNA 2110

**3217807**	6.65	8.52	1.12	0.54	-3.65	0.033	TEX10	testis expressed 10
**3290785**	6.8	8.67	1.15	0.27	-3.64	0.0278	CCDC6	coiled-coil domain containing 6
**3422326**	6.15	8.02	0.83	0.55	-3.64	0.0201	TBC1D15	TBC1 domain family, member 15
**3989180**	5.19	7.06	1.28	0.21	-3.64	0.0425	MCTS1	malignant T-cell amplified sequence 1
**3167220**	7.36	9.22	1.23	0.21	-3.63	0.0285	UBE2R2	ubiquitin-conjugating enzyme E2R 2
**3409006**	6	7.86	1.48	0.12	-3.63	0.0379	MED21	mediator complex subunit 21
**3454680**	6.25	8.11	0.92	0.69	-3.63	0.0428	TFCP2; GALNT6	transcription factor CP2; polypeptide N-acetylgalactosaminyltransferase 6
**3340697**	7.86	9.72	1.47	0.34	-3.63	0.0315	UVRAG	UV radiation resistance associated
**2826064**	5.16	7.02	1.07	0.31	-3.62	0.0461	SRFBP1	serum response factor binding protein 1
**3685183**	7.41	9.27	0.92	0.34	-3.62	0.0163	GGA2	golgi-associated, gamma adaptin ear containing, ARF binding protein 2
**3752709**	6.32	8.18	0.99	0.44	-3.62	0.0139	MYO1D	myosin ID
**3417309**	10.13	11.99	1.29	0.66	-3.62	0.0409	PA2G4	proliferation-associated 2G4
**3875195**	5.22	7.07	1.24	0.34	-3.61	0.0284	MCM8	minichromosome maintenance 8 homologous recombination repair factor
**4027769**	4.11	5.96	0.63	0.74	-3.61	0.0178	CLIC2	chloride intracellular channel 2
**2860666**	2.66	4.52	1.28	0.33	-3.61	0.0405	AK6; TAF9	adenylate kinase 6; TAF9 RNA polymerase II, TATA box binding protein (TBP)-associated factor, 32kDa
**3422855**	6.53	8.38	1.24	0.32	-3.61	0.0092	GLIPR1	GLI pathogenesis-related 1
**3536663**	8.1	9.95	1.23	0.62	-3.6	0.0324	MAPK1IP1L	mitogen-activated protein kinase 1 interacting protein 1-like
**3410445**	5.56	7.41	1.53	0.17	-3.6	0.0468	BICD1	bicaudal D homolog 1 (Drosophila)
**3163136**	6.23	8.08	1.18	0.28	-3.6	0.0164	SNAPC3	small nuclear RNA activating complex polypeptide 3
**2825514**	6.36	8.2	1.2	0.54	-3.59	0.045	DMXL1	Dmx-like 1
**3212143**	7.53	9.37	1.27	0.25	-3.58	0.0353	UBQLN1	ubiquilin 1
**3915087**	6.13	7.97	1.34	0.04	-3.58	0.0312	USP25	ubiquitin specific peptidase 25
**3451670**	5.18	7.02	0.72	0.64	-3.58	0.0339	PUS7L	pseudouridylate synthase 7-like
**2432851**	5.18	7.02	1.05	0.24	-3.58	0.0486	NBPF11; NBPF20;	neuroblastoma breakpoint family, member 11;

							NBPF8; NBPF25P	neuroblastoma breakpoint family, member 20; neuroblastoma breakpoint family, member 8; neuroblastoma breakpoint family, member 25, pseudogene
**3635776**	4.08	5.91	0.81	0.71	-3.57	0.0378	EFTUD1	elongation factor Tu GTP binding domain containing 1
**3594031**	5.25	7.08	0.74	0.33	-3.57	0.0039	TMOD2	tropomodulin 2 (neuronal)
**3529156**	5.63	7.46	0.86	0.7	-3.56	0.0371	NGDN	neuroguidin, EIF4E binding protein
**2363042**	6.91	8.74	1	0.13	-3.55	0.0405	PEA15	phosphoprotein enriched in astrocytes 15
**3096214**	7.9	9.72	0.83	0.32	-3.55	0.0248	VDAC3	voltage-dependent anion channel 3
**3419849**	6.19	8.02	1.04	0.69	-3.54	0.0301	TBK1	TANK-binding kinase 1
**3360622**	5.8	7.61	1.16	0.61	-3.53	0.0342	TRIM5; TRIM22	tripartite motif containing 5; tripartite motif containing 22
**3651018**	4.7	6.52	0.66	0.74	-3.53	0.0396	CCP110	centriolar coiled coil protein 110kDa
**3321150**	5.28	7.09	0.92	0.43	-3.52	0.02	ARNTL	aryl hydrocarbon receptor nuclear translocator-like
**3150663**	5.96	7.78	0.94	0.2	-3.52	0.0104	TAF2	TAF2 RNA polymerase II, TATA box binding protein (TBP)-associated factor, 150kDa
**3445670**	6.68	8.5	1.2	0.09	-3.52	0.0205	WBP11	WW domain binding protein 11
**3405531**	7.15	8.97	1.18	0.36	-3.52	0.0213	DDX47; APOLD1	DEAD (Asp-Glu-Ala-Asp) box polypeptide 47; apolipoprotein L domain containing 1
**3405032**	7.63	9.44	1.51	0.19	-3.5	0.0418	ETV6	ets variant 6
**3067478**	3.34	5.15	0.15	0.11	-3.5	7.28E-05	NRCAM	neuronal cell adhesion molecule
**2738146**	6.44	8.24	1.05	0.46	-3.5	0.0407	TET2	tet methylcytosine dioxygenase 2
**3976124**	6.52	8.33	0.89	0.03	-3.49	0.0301	CDK16	cyclin-dependent kinase 16
**3595846**	4.29	6.09	0.61	0.82	-3.49	0.0423	FAM63B	family with sequence similarity 63, member B
**3790259**	7.09	8.89	1.53	0.34	-3.48	0.0477	MALT1	MALT1 paracaspase
**3319137**	6.23	8.03	0.9	0.4	-3.48	0.0116	PPFIBP2	PTPRF interacting protein, binding protein 2 (liprin beta 2)
**3997946**	6.97	8.76	1.32	0.24	-3.48	0.0318	PRKX	protein kinase, X-linked
**3223646**	5.47	7.27	1.15	0.62	-3.48	0.0451	PSMD5	proteasome 26S subunit, non-ATPase 5

**3564071**	6.8	8.6	1.29	0.12	-3.47	0.028	NIN; PYGL	ninein (GSK3B interacting protein); phosphorylase, glycogen, liver
**3483348**	6.93	8.72	1.1	0.2	-3.47	0.0212	POMP	proteasome maturation protein
**3468345**	6.1	7.9	0.29	0.2	-3.47	0.0002	IGF1	insulin-like growth factor 1 (somatomedin C)
**3333603**	5.77	7.56	0.75	0.54	-3.46	0.0285	TTC9C	tetratricopeptide repeat domain 9C
**3813604**	6.23	8.02	1.08	0.53	-3.45	0.046	ZADH2	zinc binding alcohol dehydrogenase domain containing 2
**2569908**	4.64	6.42	1.01	0.61	-3.45	0.0106	10-Sep	septin 10
**3303109**	5.95	7.74	1.09	0.25	-3.44	0.0271	COX15	cytochrome c oxidase assembly homolog 15 (yeast)
**3159946**	7.2	8.98	1.27	0.56	-3.44	0.039	SMARCA2	SWI/SNF related, matrix associated, actin dependent regulator of chromatin, subfamily a, member 2
**3540398**	5.59	7.37	0.68	0.25	-3.44	0.0109	CHURC1-FNTB; FNTB	CHURC1-FNTB readthrough; farnesyltransferase, CAAX box, beta
**3728325**	4.47	6.25	1.19	0.77	-3.44	0.0336	FLJ11710	uncharacterized protein FLJ11710
**3234760**	7.28	9.05	0.94	0.14	-3.43	0.0124	CELF2	CUGBP, Elav-like family member 2
**2945741**	6.32	8.1	0.82	0.3	-3.43	0.0102	FAM65B	family with sequence similarity 65, member B
**3403092**	9.07	10.85	1.45	0.27	-3.43	0.0481	PTPN6	protein tyrosine phosphatase, non-receptor type 6
**3360587**	4.04	5.82	0.85	0.43	-3.43	0.017	OR52H1	olfactory receptor, family 52, subfamily H, member 1
**3409432**	4.83	6.61	0.89	0.43	-3.43	0.0208	CCDC91	coiled-coil domain containing 91
**3591963**	5.31	7.08	0.91	0.46	-3.42	0.0402	EIF3J	eukaryotic translation initiation factor 3, subunit J
**4027828**	5.2	6.97	0.81	0.39	-3.42	0.0365	TMLHE	trimethyllysine hydroxylase, epsilon
**2601287**	5.74	7.51	0.62	0.64	-3.42	0.011	AP1S3	adaptor-related protein complex 1 sigma 3 subunit
**3629698**	5.77	7.55	0.75	0.49	-3.42	0.0136	DPP8	dipeptidyl-peptidase 8
**3286975**	5.7	7.47	0.59	0.62	-3.41	0.0157	ZFAND4	zinc finger, AN1-type domain 4
**2554975**	6.22	7.99	0.49	0.43	-3.41	0.0035	BCL11A	B-cell CLL/lymphoma 11A (zinc finger protein)
**3568310**	6.52	8.29	0.59	0.48	-3.41	0.0113	ZBTB25	zinc finger and BTB domain containing 25
**3518169**	5.5	7.27	1.26	0.38	-3.4	0.0404	COMMD6	COMM domain containing 6
**3135184**	4.49	6.25	0.66	0.58	-3.4	0.0401	RB1CC1	RB1-inducible coiled-coil 1

**2330133**	5.69	7.45	0.95	0.17	-3.4	0.0363	AGO3	argonaute RISC catalytic component 3
**3843275**	2.85	4.61	0.45	0.37	-3.39	0.0072	ZNF749; ZNF419	zinc finger protein 749; zinc finger protein 419
**2506903**	7.22	8.98	1.23	0.55	-3.39	0.0289	MGAT5	mannosyl (alpha-1,6-)-glycoprotein beta-1,6-N-acetyl-glucosaminyltransferase
**3564997**	7.36	9.12	1.21	0.28	-3.39	0.0188	DDHD1	DDHD domain containing 1
**3255402**	5.41	7.17	0.34	0.44	-3.39	0.0028	CCSER2; TNPO1P1	coiled-coil serine rich protein 2; transportin 1 pseudogene 1
**2536183**	6.7	8.46	1.03	0.4	-3.38	0.0472	PPP1R7	protein phosphatase 1, regulatory subunit 7
**3519119**	7.34	9.1	1.31	0.18	-3.38	0.0222	RBM26	RNA binding motif protein 26
**3881651**	6.64	8.4	0.51	0.84	-3.38	0.0058	HCK	HCK proto-oncogene, Src family tyrosine kinase
**2556529**	6.02	7.78	0.7	0.67	-3.38	0.0256	SERTAD2	SERTA domain containing 2
**3526425**	7.03	8.79	0.39	0.51	-3.37	0.0113	PCID2	PCI domain containing 2
**3099750**	6.9	8.66	0.89	0.74	-3.37	0.0398	SDCBP	syndecan binding protein
**3577256**	3.72	5.47	0.54	1.07	-3.37	0.0433	C14orf142	chromosome 14 open reading frame 142
**2496536**	5.25	7	0.54	0.16	-3.37	0.0015	RPL31; TBC1D8	ribosomal protein L31; TBC1 domain family, member 8 (with GRAM domain)
**3622934**	6.36	8.11	1.19	0.35	-3.37	0.027	MYEF2	myelin expression factor 2
**3919278**	6.4	8.15	1.01	0.09	-3.37	0.0145	CLIC6	chloride intracellular channel 6
**3447129**	5.96	7.71	1.17	0.33	-3.36	0.0463	C2CD5	C2 calcium-dependent domain containing 5
**3545311**	6.07	7.82	0.66	0.36	-3.36	0.025	CIPC	CLOCK-interacting pacemaker
**3359751**	6.55	8.3	0.97	0.78	-3.36	0.023	ZNF195	zinc finger protein 195
**2378662**	5.87	7.62	0.83	0.35	-3.36	0.0296	TRAF5	TNF receptor-associated factor 5
**3901191**	5.41	7.16	1.23	0.6	-3.35	0.0472	NAPB	N-ethylmaleimide-sensitive factor attachment protein, beta
**3387033**	5.27	7.02	1.04	0.43	-3.35	0.0213	MRE11A	MRE11 homolog A, double strand break repair nuclease
**2444790**	5.54	7.29	1.03	0.22	-3.35	0.0399	MRPS14	mitochondrial ribosomal protein S14
**2612813**	6.07	7.81	0.99	0.66	-3.34	0.0363	PLCL2; MIR3714	phospholipase C-like 2; microRNA 3714
**3031517**	5.37	7.11	0.26	0.96	-3.34	0.0099	GIMAP7	GTPase, IMAP family member 7
**2352758**	6.59	8.33	0.88	0.32	-3.34	0.0401	HIPK1	homeodomain interacting protein kinase 1
**3145801**	5.69	7.43	0.08	0.44	-3.34	0.0012	TSPYL5	TSPY-like 5

**3562746**	5.41	7.15	0.73	0.29	-3.34	0.0054	MIS18BP1	MIS18 binding protein 1
**3918696**	6.44	8.18	0.75	0.19	-3.33	0.0152	SON	SON DNA binding protein
**3938817**	6.7	8.43	0.87	0.51	-3.33	0.0173	BMS1P20	BMS1 ribosome biogenesis factor pseudogene 20
**3815649**	8.09	9.82	1.39	0.18	-3.33	0.0402	CIRBP	cold inducible RNA binding protein
**3412296**	5.55	7.28	0.91	0.2	-3.33	0.0123	IRAK4	interleukin 1 receptor associated kinase 4
**2714644**	6.05	7.78	1.08	0.31	-3.32	0.0456	CTBP1-AS2	CTBP1 antisense RNA 2 (head to head)
**3027956**	3.84	5.57	1.05	1.27	-3.31	0.0453	TAS2R4	taste receptor, type 2, member 4
**3322251**	4.71	6.43	0.34	0.88	-3.31	0.0442	NUCB2	nucleobindin 2
**3962587**	5.76	7.49	0.93	0.76	-3.31	0.0484	ARFGAP3; PACSIN2	ADP-ribosylation factor GTPase activating protein 3; protein kinase C and casein kinase substrate in neurons 2
**3701384**	5.78	7.51	1.4	0.28	-3.31	0.046	CMC2	C-x(9)-C motif containing 2
**3626704**	6.07	7.8	1.18	0.63	-3.3	0.0446	SLTM	SAFB-like, transcription modulator
**3608298**	5.46	7.18	0.99	0.38	-3.3	0.024	BLM	Bloom syndrome, RecQ helicase-like
**2835006**	6.74	8.46	0.97	0.25	-3.3	0.0426	GRPEL2	GrpE-like 2, mitochondrial (E. coli)
**3561321**	6.32	8.04	0.58	0.56	-3.3	0.0137	MBIP	MAP3K12 binding inhibitory protein 1
**3390067**	6.74	8.46	1.26	0.33	-3.3	0.0346	NPAT	nuclear protein, ataxia-telangiectasia locus
**2743029**	5.17	6.89	1.15	0.19	-3.3	0.0444	ABHD18	abhydrolase domain containing 18
**3131916**	6.88	8.6	1.28	0.63	-3.29	0.0385	WHSC1L1	Wolf-Hirschhorn syndrome candidate 1-like 1
**3515009**	5.97	7.69	1.04	0.67	-3.29	0.042	VPS36	vacuolar protein sorting 36 homolog (S. cerevisiae)
**2330002**	4.92	6.64	0.68	0.5	-3.28	0.0273	AGO4	argonaute RISC catalytic component 4
**3439178**	7.11	8.82	0.71	0.68	-3.28	0.034	PXMP2	peroxisomal membrane protein 2
**3232349**	7.98	9.69	1.12	0.41	-3.27	0.0422	PFKP	phosphofructokinase, platelet
**2911257**	4.98	6.69	0.82	0.52	-3.27	0.0494	KIAA1586; ZNF451	KIAA1586; zinc finger protein 451
**3595096**	6.76	8.46	1.34	0.07	-3.26	0.0428	TCF12	transcription factor 12
**2388085**	4.51	6.21	0.34	0.82	-3.26	0.0289	KMO	kynurenine 3-monooxygenase (kynurenine 3-hydroxylase)
**3476012**	5.43	7.14	1.14	0.4	-3.26	0.0378	MPHOSPH9	M-phase phosphoprotein 9
**3919952**	5.85	7.55	0.52	0.73	-3.26	0.0288	MORC3	MORC family CW-type zinc finger 3

**3556888**	6.43	8.13	1.26	0.53	-3.26	0.0476	RBM23	RNA binding motif protein 23
**3323443**	5.09	6.8	0.99	0.28	-3.26	0.0303	PRMT3	protein arginine methyltransferase 3
**3301011**	5.31	7.01	0.93	0.35	-3.25	0.0277	NOC3L	NOC3-like DNA replication regulator
**3989089**	6.33	8.03	0.54	0.24	-3.25	0.0031	ZBTB33	zinc finger and BTB domain containing 33
**3620457**	6.9	8.6	1.27	0.38	-3.25	0.0424	VPS39	vacuolar protein sorting 39 homolog (S. cerevisiae)
**3556386**	5.56	7.26	0.73	0.23	-3.25	0.0168	RAB2B	RAB2B, member RAS oncogene family
**3387537**	5.73	7.43	0.83	0.34	-3.25	0.0152	MAML2	mastermind-like transcriptional coactivator 2
**3365437**	6.09	7.79	0.59	0.77	-3.24	0.0498	TSG101	tumor susceptibility 101
**3311775**	5.39	7.09	0.62	0.59	-3.24	0.023	DHX32	DEAH (Asp-Glu-Ala-His) box polypeptide 32
**3765689**	7.51	9.21	1.07	0.42	-3.24	0.0354	MED13	mediator complex subunit 13
**3402978**	4.01	5.71	0.79	0.85	-3.24	0.0311	DSTNP2	destrin (actin depolymerizing factor) pseudogene 2
**3463522**	4.97	6.66	0.81	0.33	-3.24	0.034	PAWR	PRKC, apoptosis, WT1, regulator
**3978819**	4.93	6.62	0.42	0.56	-3.23	0.0098	RRAGB	Ras-related GTP binding B
**2378121**	6.11	7.81	1.41	0.32	-3.23	0.0478	TRAF3IP3	TRAF3 interacting protein 3
**3928866**	6.76	8.45	0.92	0.09	-3.23	0.0098	SCAF4	SR-related CTD-associated factor 4
**3715109**	8.51	10.2	1.22	0.37	-3.22	0.0273	WSB1	WD repeat and SOCS box containing 1
**2405364**	5.76	7.45	0.98	0.36	-3.22	0.0257	AK2	adenylate kinase 2
**3451264**	5.23	6.92	0.6	0.28	-3.22	0.0107	YAF2	YY1 associated factor 2
**3891006**	6.95	8.63	1.31	0.06	-3.21	0.0346	STX16; NPEPL1; STX16-NPEPL1	syntaxin 16; aminopeptidase-like 1; STX16-NPEPL1 readthrough (NMD candidate)
**3250204**	6.35	8.04	1.22	0.52	-3.21	0.0472	SUPV3L1	SUV3-like helicase
**3131741**	5.96	7.64	0.78	0.26	-3.21	0.0102	RAB11FIP1	RAB11 family interacting protein 1 (class I)
**2468376**	6.07	7.76	0.33	0.6	-3.21	0.0208	RNF144A	ring finger protein 144A
**4011889**	6.82	8.51	0.95	0.49	-3.21	0.0415	ZMYM3	zinc finger, MYM-type 3
**2633691**	4.99	6.67	0.09	1.06	-3.2	0.0423	TMEM45A	transmembrane protein 45A
**3620880**	5.89	7.57	0.83	0.48	-3.2	0.0271	UBR1; EPB42; FDPSP4	ubiquitin protein ligase E3 component n-recognin 1; erythrocyte membrane protein band 4.2; farnesyl diphosphate synthase pseudogene 4

**3962260**	6.75	8.42	1.16	0.37	-3.19	0.0482	NDUFA6	NADH dehydrogenase (ubiquinone) 1 alpha subcomplex, 6, 14kDa
**2953852**	5.83	7.5	0.84	0.32	-3.19	0.0422	MED20	mediator complex subunit 20
**2852591**	6.2	7.87	0.03	0.43	-3.19	0.0007	ADAMTS12	ADAM metallopeptidase with thrombospondin type 1 motif 12
**3463571**	6.26	7.93	1.23	0.03	-3.19	0.0312	PPP1R12A	protein phosphatase 1, regulatory subunit 12A
**4016001**	3.64	5.31	0.69	0.7	-3.19	0.0449	ZMAT1	zinc finger, matrin-type 1
**3990512**	7.33	9	1.29	0.22	-3.19	0.0399	SASH3	SAM and SH3 domain containing 3
**3693083**	6.54	8.22	0.72	0.31	-3.19	0.0205	FAM192A	family with sequence similarity 192, member A
**2486927**	5.49	7.16	0.37	0.3	-3.19	0.0018	ARHGAP25	Rho GTPase activating protein 25
**3188050**	6.17	7.84	1.18	0.35	-3.18	0.0389	MRRF	mitochondrial ribosome recycling factor
**3457614**	8.03	9.7	1.39	0.5	-3.18	0.0488	CS	citrate synthase
**3480129**	7.08	8.75	1.5	0.27	-3.18	0.0343	ZMYM2	zinc finger, MYM-type 2
**3569754**	8.75	10.42	1.06	0.28	-3.18	0.0202	ZFP36L1	ZFP36 ring finger protein-like 1
**3365776**	5.61	7.28	0.72	0.31	-3.18	0.038	E2F8	E2F transcription factor 8
**3133465**	5.36	7.03	0.83	0.62	-3.18	0.0406	THAP1	THAP domain containing, apoptosis associated protein 1
**2619120**	6.39	8.06	1.09	0.26	-3.18	0.0499	TRAK1	trafficking protein, kinesin binding 1
**3448975**	6.11	7.77	1.15	0.08	-3.17	0.0204	ERGIC2	ERGIC and golgi 2
**3085990**	7.99	9.65	1.2	0.51	-3.17	0.0387	BLK	BLK proto-oncogene, Src family tyrosine kinase
**3347658**	6.72	8.38	1.36	0.39	-3.17	0.0298	ATM	ATM serine/threonine kinase
**3329649**	7.42	9.09	1.23	0.19	-3.17	0.0489	DDB2	damage-specific DNA binding protein 2
**3165780**	3.61	5.27	0.58	0.48	-3.16	0.0249	IFT74	intraflagellar transport 74
**3161566**	6.67	8.33	1.25	0.54	-3.16	0.0313	KDM4C	lysine (K)-specific demethylase 4C
**3420442**	4.56	6.22	0.6	0.25	-3.16	0.0052	IRAK3	interleukin 1 receptor associated kinase 3
**3925473**	5.33	6.99	1.1	0.71	-3.16	0.0364	SAMSN1	SAM domain, SH3 domain and nuclear localization signals 1
**3589697**	5.87	7.53	1.15	0.27	-3.16	0.0327	PAK6; BUB1B	p21 protein (Cdc42/Rac)-activated kinase 6; BUB1 mitotic checkpoint serine/threonine kinase B
**2769947**	4.57	6.22	0.86	0.08	-3.15	0.0395	CLOCK	clock circadian regulator

**3452622**	4.19	5.85	0.48	0.59	-3.15	0.0213	RPAP3	RNA polymerase II associated protein 3
**3011250**	6.89	8.55	1.27	0.08	-3.15	0.0409	DMTF1	cyclin D binding myb-like transcription factor 1
**3336378**	7.1	8.76	1.11	0.35	-3.15	0.0405	RBM14; RBM14-RBM4	RNA binding motif protein 14; RBM14-RBM4 readthrough
**3471005**	6.65	8.31	1.3	0.1	-3.15	0.0378	GIT2	G protein-coupled receptor kinase interacting ArfGAP 2
**3510362**	6.2	7.86	1.38	0.55	-3.15	0.0473	PROSER1	proline and serine rich 1
**3192117**	7.38	9.03	0.97	0.16	-3.15	0.0182	PRRC2B; SNORD62A; SNORD62B	proline-rich coiled-coil 2B; small nucleolar RNA, C/D box 62A; small nucleolar RNA, C/D box 62B
**3428131**	5.71	7.37	1.16	0.36	-3.15	0.0311	SCYL2	SCY1-like, kinase-like 2
**2995320**	4.4	6.05	0.49	0.47	-3.14	0.023	DKFZP586I1420	uncharacterized protein DKFZp586I1420
**3600744**	7.04	8.7	1.18	0.35	-3.14	0.0378	ARIH1; MIR630	ariadne RBR E3 ubiquitin protein ligase 1; microRNA 630
**3549033**	5.11	6.76	1.13	0.25	-3.14	0.0324	GOLGA5	golgin A5
**3811086**	5.51	7.16	0.81	0.48	-3.14	0.0297	PIGN	phosphatidylinositol glycan anchor biosynthesis class N
**3522662**	6.53	8.18	0.29	0.29	-3.13	0.0003	GPR183	G protein-coupled receptor 183
**3747199**	6.08	7.73	0.63	0.45	-3.13	0.0432	CENPV	centromere protein V
**3235932**	5.62	7.26	0.71	0.62	-3.13	0.0261	PRPF18	pre-mRNA processing factor 18
**3304116**	6.19	7.84	0.84	0.11	-3.13	0.0085	C10orf76	chromosome 10 open reading frame 76
**3471300**	6.97	8.61	0.8	0.72	-3.13	0.0452	PPTC7	PTC7 protein phosphatase homolog
**3778823**	6.36	8	0.94	0.59	-3.13	0.0481	NAPG	N-ethylmaleimide-sensitive factor attachment protein, gamma
**3755359**	6.58	8.22	1.01	0.46	-3.12	0.0441	PIP4K2B	phosphatidylinositol-5-phosphate 4-kinase, type II, beta
**3454006**	7.1	8.74	0.83	0.52	-3.12	0.0297	FMNL3	formin like 3
**3860552**	5.85	7.49	0.88	0.43	-3.12	0.0314	ZNF529	zinc finger protein 529
**3591909**	6.42	8.05	0.97	0.63	-3.11	0.0337	CTDSPL2	CTD small phosphatase like 2
**3904566**	5.97	7.61	0.62	0.17	-3.11	0.0104	DSN1	DSN1 homolog, MIS12 kinetochore complex component
**3836044**	5.94	7.57	0.65	0.37	-3.11	0.012	GEMIN7; MARK4; PPP1R37	gem nuclear organelle associated protein 7; MAP/microtubule affinity-regulating kinase 4; protein

								phosphatase 1, regulatory subunit 37
**3803882**	4.81	6.44	0.89	0.33	-3.1	0.0291	ZSCAN30	zinc finger and SCAN domain containing 30
**3464912**	4.77	6.4	1.17	0.33	-3.1	0.0475	POC1B; POC1B-GALNT4	POC1 centriolar protein B; POC1B-GALNT4 readthrough
**3904119**	7.38	9.01	0.94	0.43	-3.09	0.0359	RBM12; CPNE1	RNA binding motif protein 12; copine I
**2458701**	6.9	8.53	0.9	0.34	-3.09	0.0395	ACBD3	acyl-CoA binding domain containing 3
**3784670**	4.67	6.29	0.88	0.04	-3.09	0.0132	C18orf21	chromosome 18 open reading frame 21
**3715368**	6.67	8.29	0.91	0.13	-3.08	0.0125	NLK	nemo-like kinase
**3504054**	3.15	4.77	0.41	0.34	-3.08	0.0018	ZMYM5	zinc finger, MYM-type 5
**3732448**	6.65	8.27	1.21	0.17	-3.08	0.0358	BPTF	bromodomain PHD finger transcription factor
**3783749**	5.24	6.86	0.53	0.68	-3.08	0.0345	RNF138	ring finger protein 138, E3 ubiquitin protein ligase
**3332403**	9.35	10.97	0.74	0.78	-3.08	0.0169	MS4A1	membrane-spanning 4-domains, subfamily A, member 1
**2444842**	6.94	8.56	0.82	0.35	-3.08	0.0489	KIAA0040	KIAA0040
**3833214**	5.2	6.83	0.75	0.59	-3.08	0.0142	LGALS17A	Charcot-Leyden crystal protein pseudogene
**3105777**	6.39	8.01	0.91	0.11	-3.07	0.0459	WWP1	WW domain containing E3 ubiquitin protein ligase 1
**3850501**	3.95	5.57	0.86	0.77	-3.07	0.008	ILF3-AS1	ILF3 antisense RNA 1 (head to head)
**2532894**	7.53	9.14	1.37	0.31	-3.07	0.0464	DGKD	diacylglycerol kinase, delta 130kDa
**3873389**	7.19	8.81	1.22	0.18	-3.07	0.0452	PSMF1	proteasome inhibitor subunit 1
**3227696**	7.72	9.34	1.05	0.34	-3.07	0.0402	RAPGEF1	Rap guanine nucleotide exchange factor 1
**3916576**	6.06	7.67	0.71	0.94	-3.07	0.0459	GABPA	GA binding protein transcription factor alpha subunit
**3452970**	6.12	7.74	1.08	0.13	-3.07	0.0274	SENP1	SUMO1/sentrin specific peptidase 1
**3408573**	4.38	6	0.97	0.72	-3.07	0.047	LYRM5	LYR motif containing 5
**3576812**	5.27	6.88	0.61	0.49	-3.07	0.0177	TRIP11; ATXN3	thyroid hormone receptor interactor 11; ataxin 3
**3325768**	5.22	6.84	0.94	0.36	-3.07	0.029	QSER1	glutamine and serine rich 1
**2633587**	5.14	6.76	0.75	0.4	-3.06	0.0346	TBC1D23	TBC1 domain family, member 23
**3442205**	6.78	8.39	1.13	0.34	-3.06	0.0266	ZNF384	zinc finger protein 384
**3563459**	5.66	7.28	0.92	0.19	-3.06	0.0174	NEMF	nuclear export mediator factor

**3690550**	5.61	7.23	0.77	0.53	-3.06	0.0368	SIAH1	siah E3 ubiquitin protein ligase 1
**2365675**	6.44	8.04	1.07	0.54	-3.05	0.0498	POU2F1	POU class 2 homeobox 1
**3786039**	5.97	7.57	1.14	0.39	-3.04	0.032	PIK3C3	phosphatidylinositol 3-kinase, catalytic subunit type 3
**3347831**	6.38	7.98	1.39	0.31	-3.04	0.0495	DDX10	DEAD (Asp-Glu-Ala-Asp) box polypeptide 10
**4017519**	6.09	7.69	1.12	0.35	-3.04	0.0446	PSMD10	proteasome 26S subunit, non-ATPase 10
**3615985**	5.68	7.29	0.81	0.17	-3.04	0.0138	MTMR10	myotubularin related protein 10
**3946095**	5.78	7.39	0.1	0.55	-3.04	0.002	GRAP2	GRB2-related adaptor protein 2
**3304215**	7.29	8.89	1.16	0.25	-3.04	0.0304	LDB1	LIM domain binding 1
**3920566**	6.59	8.19	1.11	0.08	-3.03	0.0299	DYRK1A	dual specificity tyrosine-(Y)-phosphorylation regulated kinase 1A
**3983962**	5.09	6.69	0.9	0.46	-3.03	0.0217	DIAPH2	diaphanous-related formin 2
**3327906**	7.24	8.84	0.95	0.74	-3.03	0.0326	API5	apoptosis inhibitor 5
**3497586**	6.55	8.15	1.05	0.35	-3.03	0.0347	MBNL2	muscleblind-like splicing regulator 2
**3676669**	7.6	9.19	0.94	0.19	-3.03	0.0198	RNPS1	RNA binding protein S1, serine-rich domain
**3407229**	6.75	8.35	0.62	0.28	-3.02	0.0108	AEBP2	AE binding protein 2
**3558012**	6.89	8.49	0.9	0.46	-3.02	0.0375	TINF2	TERF1 (TRF1)-interacting nuclear factor 2
**3744217**	6.06	7.66	0.77	0.25	-3.02	0.0211	VAMP2	vesicle associated membrane protein 2
**3303339**	5.92	7.51	1	0.73	-3.02	0.0481	CWF19L1; SNORA12	CWF19-like 1, cell cycle control (S. pombe); small nucleolar RNA, H/ACA box 12
**2449619**	5.08	6.68	0.76	0.21	-3.02	0.016	ZBTB41	zinc finger and BTB domain containing 41
**3806905**	6.34	7.94	0.99	0.32	-3.02	0.0156	SMAD2	SMAD family member 2
**3308967**	5.5	7.09	0.39	0.38	-3.02	0.0092	FAM204A	family with sequence similarity 204, member A
**3590422**	6.14	7.73	0.91	0.3	-3.01	0.028	RTF1	RTF1 homolog, Paf1/RNA polymerase II complex component
**2866590**	6.37	7.95	1.34	0.41	-3.01	0.0458	LYSMD3	LysM, putative peptidoglycan-binding, domain containing 3
**2711034**	6.18	7.77	1.29	0.15	-3.01	0.0489	MB21D2	Mab-21 domain containing 2
**4013730**	6.28	7.86	0.98	0.27	-3.01	0.0142	BRWD3	bromodomain and WD repeat domain containing 3
**3819880**	5.89	7.47	0.52	0.3	-3	0.0151	ZNF317	zinc finger protein 317

**3543411**	6.41	7.99	0.92	0.51	-3	0.0433	RBM25	RNA binding motif protein 25
**3560864**	4.69	6.28	0.54	0.53	-3	0.0468	PPP2R3C	protein phosphatase 2, regulatory subunit B, gamma
**2530425**	5.45	7.03	0.58	0.36	-3	0.0093	COL4A3	collagen, type IV, alpha 3 (Goodpasture antigen)
**3332334**	4.3	5.88	0.21	0.39	-2.99	0.0011	MS4A14; MS4A7	membrane-spanning 4-domains, subfamily A, member 14; membrane-spanning 4-domains, subfamily A, member 7
**3243078**	6.13	7.71	0.81	0.41	-2.99	0.0235	ZNF33A	zinc finger protein 33A
**2451309**	6.6	8.18	0.57	1.03	-2.99	0.0211	KDM5B	lysine (K)-specific demethylase 5B
**3375648**	5.93	7.51	0.77	0.44	-2.99	0.0328	FTH1	ferritin, heavy polypeptide 1
**3169094**	5.82	7.4	0.92	0.57	-2.99	0.0472	DCAF10	DDB1 and CUL4 associated factor 10
**3439305**	5.68	7.26	0.77	0.03	-2.99	0.0076	ZNF84	zinc finger protein 84
**3359910**	7.03	8.61	1.18	0.22	-2.98	0.0494	NUP98	nucleoporin 98kDa
**3498476**	5.06	6.64	1.34	0.41	-2.98	0.0229	LOC100132099	FRSS1829
**3671850**	6.47	8.05	0.73	0.44	-2.98	0.041	KLHL36	kelch-like family member 36
**3970642**	4.97	6.54	0.06	0.46	-2.98	0.0011	CDKL5	cyclin-dependent kinase-like 5
**3779684**	6.62	8.2	0.91	0.15	-2.98	0.0157	PSMG2	proteasome (prosome, macropain) assembly chaperone 2
**2485406**	5.37	6.94	0.52	0.65	-2.97	0.0235	LGALSL	lectin, galactoside-binding-like
**2750594**	7.24	8.81	0.79	0.59	-2.97	0.0191	MSMO1	methylsterol monooxygenase 1
**4026722**	7.23	8.81	0.73	0.26	-2.97	0.0157	IDH3G	isocitrate dehydrogenase 3 (NAD+) gamma
**3142217**	6.82	8.39	0.76	0.22	-2.97	0.0063	PAG1	phosphoprotein membrane anchor with glycosphingolipid microdomains 1
**3444368**	3.08	4.65	0.52	0.46	-2.97	0.003	PRH1; TAS2R14; PRR4	proline-rich protein HaeIII subfamily 1; taste receptor, type 2, member 14; proline rich 4 (lacrimal)
**3853814**	7.05	8.62	0.97	0.41	-2.97	0.025	EPS15L1	epidermal growth factor receptor pathway substrate 15-like 1
**3551303**	6.75	8.32	1.17	0.27	-2.97	0.0375	CCNK	cyclin K
**3188299**	6.36	7.93	1.09	0.32	-2.97	0.0363	RABGAP1; GPR21	RAB GTPase activating protein 1; G protein-coupled receptor 21

**3977067**	8.54	10.11	1.1	0.17	-2.97	0.0376	PLP2	proteolipid protein 2 (colonic epithelium-enriched)
**3828887**	5.61	7.18	1.21	0.26	-2.96	0.0219	ZNF507	zinc finger protein 507
**3740487**	5.41	6.98	1.18	0.47	-2.96	0.0498		
**3509910**	6.28	7.84	1.15	0.24	-2.96	0.0352	SUPT20H	SPT20 homolog, SAGA complex component
**3929821**	5.34	6.9	0.67	0.36	-2.96	0.0108	CRYZL1	crystallin zeta like 1
**2603897**	4.23	5.8	0.61	0.54	-2.96	0.0137	TIGD1	tigger transposable element derived 1
**3932148**	5.45	7.02	0.8	0.5	-2.96	0.0149	BRWD1	bromodomain and WD repeat domain containing 1
**3240340**	6.81	8.38	1.33	0.22	-2.96	0.0404	WAC	WW domain containing adaptor with coiled-coil
**3904527**	5.24	6.8	0.81	0.42	-2.96	0.0445	NDRG3	NDRG family member 3
**3568667**	6.26	7.82	0.76	0.05	-2.95	0.0086	MAX	MYC associated factor X
**3588658**	5.83	7.39	0.69	0.05	-2.95	0.0193	C15orf41; LARP4P	chromosome 15 open reading frame 41; La ribonucleoprotein domain family, member 4 pseudogene
**3507003**	5.6	7.17	0.93	0.36	-2.95	0.0265	LNX2	ligand of numb-protein X 2
**3563922**	5.59	7.15	0.82	0.11	-2.95	0.0086	MAP4K5	mitogen-activated protein kinase kinase kinase kinase 5
**2558511**	7.23	8.79	1.2	0.22	-2.95	0.0363	TIA1; C2orf42	TIA1 cytotoxic granule-associated RNA binding protein; chromosome 2 open reading frame 42
**3238231**	6.15	7.71	1.06	0.18	-2.95	0.0257	MLLT10	myeloid/lymphoid or mixed-lineage leukemia; translocated to, 10
**3759186**	6.25	7.81	0.92	0.35	-2.95	0.0306	GPATCH8	G-patch domain containing 8
**3375545**	7.74	9.3	0.79	0.38	-2.95	0.0306	FADS1	fatty acid desaturase 1
**3755903**	6.31	7.87	1.05	0.3	-2.94	0.0402	GSDMB	gasdermin B
**3839818**	5.14	6.69	0.68	0.35	-2.94	0.023	ZNF175	zinc finger protein 175
**3493448**	4.3	5.86	0.96	0.32	-2.94	0.0239	PIBF1	progesterone immunomodulatory binding factor 1
**3182957**	4.39	5.95	0.63	0.5	-2.94	0.0346	NIPSNAP3A; NIPSNAP3B	nipsnap homolog 3A (C. elegans); nipsnap homolog 3B (C. elegans)
**3481410**	6.79	8.34	0.36	0.55	-2.93	0.0068	TNFRSF19	tumor necrosis factor receptor superfamily, member 19
**3351931**	5.97	7.52	1.06	0.02	-2.93	0.0285	HINFP	histone H4 transcription factor
**3832383**	6.98	8.53	1.03	0.45	-2.93	0.0446	PSMD8	proteasome 26S subunit, non-ATPase 8

**3929775**	5.73	7.28	0.77	0.49	-2.93	0.0415	DONSON	downstream neighbor of SON
**3472000**	6.39	7.94	1.1	0.36	-2.93	0.035	HECTD4; RPL7AP60	HECT domain containing E3 ubiquitin protein ligase 4; ribosomal protein L7a pseudogene 60
**3457947**	7.43	8.98	1.23	0.11	-2.93	0.0447	BAZ2A	bromodomain adjacent to zinc finger domain 2A
**3781531**	6.74	8.28	0.97	0.69	-2.92	0.0444	CABLES1	Cdk5 and Abl enzyme substrate 1
**2638676**	5.97	7.51	1.15	0.36	-2.92	0.0364	EAF2	ELL associated factor 2
**3108489**	6.05	7.59	0.1	0.51	-2.92	0.0015	LAPTM4B	lysosomal protein transmembrane 4 beta
**3594825**	4.84	6.39	0.39	0.18	-2.92	0.0023	PIGB	phosphatidylinositol glycan anchor biosynthesis class B
**3617757**	6.2	7.74	1.02	0.34	-2.92	0.0295	AQR	aquarius intron-binding spliceosomal factor
**3256689**	8.66	10.21	1.13	0.33	-2.92	0.0405	PTEN	phosphatase and tensin homolog
**2922840**	4.41	5.96	0.87	0.39	-2.91	0.0378	KPNA5	karyopherin alpha 5 (importin alpha 6)
**3698055**	5.5	7.04	0.66	0.6	-2.91	0.0418	TXNL4B	thioredoxin-like 4B
**3814734**	5.61	7.15	0.72	0.37	-2.91	0.0179	TXNL4A	thioredoxin-like 4A
**3628650**	7.08	8.62	1.14	0.56	-2.91	0.0384	HERC1	HECT and RLD domain containing E3 ubiquitin protein ligase family member 1
**3559497**	5.55	7.09	0.83	0.22	-2.91	0.017	STRN3	striatin, calmodulin binding protein 3
**3987492**	5.25	6.79	1	0.38	-2.9	0.0471	ALG13	ALG13, UDP-N-acetylglucosaminyltransfera se subunit
**3379390**	5.66	7.2	0.83	0.44	-2.9	0.0323	KMT5B	lysine (K)-specific methyltransferase 5B
**3591281**	5.78	7.32	1.03	0.26	-2.9	0.0283	TMEM62	transmembrane protein 62
**3318390**	4.98	6.51	0.41	0.51	-2.9	0.0097	TRIM6; TRIM6-TRIM34; TRIM34; OR52B5P; TRIM78P	tripartite motif containing 6; TRIM6-TRIM34 readthrough; tripartite motif containing 34; olfactory receptor, family 52, subfamily B, member 5 pseudogene; tripartite motif containing 78, pseudogene
**3202421**	3.44	4.97	0.74	0.28	-2.9	0.0335	C9orf72	chromosome 9 open reading frame 72
**3553872**	7.35	8.88	0.99	0.44	-2.89	0.0408	KLC1	kinesin light chain 1
**3475679**	5.58	7.11	1.03	0.27	-2.89	0.0288	ZCCHC8	zinc finger, CCHC domain containing 8
**3913544**	7.2	8.73	0.91	0.36	-2.89	0.0298	DIDO1	death inducer-obliterator 1
**3978999**	7.23	8.76	0.85	0.27	-2.89	0.0213	UBQLN2	ubiquilin 2

**3866958**	7.05	8.59	1.08	0.29	-2.89	0.0425	CARD8	caspase recruitment domain family, member 8
**4014251**	5.34	6.87	0.63	0.57	-2.89	0.0367	CHM	choroideremia (Rab escort protein 1)
**3756344**	7.57	9.1	0.73	1.01	-2.89	0.0494	SMARCE1	SWI/SNF related, matrix associated, actin dependent regulator of chromatin, subfamily e, member 1
**3381817**	7.76	9.29	1.19	0.16	-2.89	0.0455	UCP2	uncoupling protein 2 (mitochondrial, proton carrier)
**3228884**	6.81	8.34	0.89	0.37	-2.89	0.0354	VAV2	vav 2 guanine nucleotide exchange factor
**3153428**	6.36	7.89	0.76	0.14	-2.89	0.0028	ASAP1; ASAP1-IT2	ArfGAP with SH3 domain, ankyrin repeat and PH domain 1; ASAP1 intronic transcript 2
**3672830**	7.93	9.46	1.02	0.16	-2.89	0.0286	MAP1LC3B	microtubule-associated protein 1 light chain 3 beta
**3473480**	5.63	7.16	0.64	0.63	-2.88	0.0492	FBXO21	F-box protein 21
**3617920**	3.63	5.15	0.7	0.19	-2.88	0.0125	DPH6	diphthamine biosynthesis 6
**3357723**	6.38	7.91	0.96	0.41	-2.88	0.0276	BET1L	Bet1 golgi vesicular membrane trafficking protein-like
**3130244**	3.33	4.85	0.31	0.7	-2.88	0.0083	TEX15	testis expressed 15
**3653317**	6.97	8.5	1.08	0.32	-2.88	0.0222	RBBP6	retinoblastoma binding protein 6
**3536650**	5.58	7.1	0.7	0.34	-2.87	0.0175	SOCS4	suppressor of cytokine signaling 4
**2443450**	8.71	10.23	0.58	0.07	-2.87	0.01	SELL	selectin L
**3466318**	4.34	5.86	0.4	0.07	-2.87	0.001	NR2C1; FGD6	nuclear receptor subfamily 2, group C, member 1; FYVE, RhoGEF and PH domain containing 6
**3651588**	5.61	7.13	0.96	0.38	-2.87	0.0423	LYRM1	LYR motif containing 1
**4012204**	6.23	7.75	0.89	0.35	-2.87	0.0334	HDAC8	histone deacetylase 8
**3340913**	6.25	7.76	0.9	0.18	-2.87	0.0183	EMSY	EMSY BRCA2-interacting transcriptional repressor
**3488253**	5.83	7.34	0.66	0.23	-2.86	0.0076	COG3	component of oligomeric golgi complex 3
**3322958**	6.26	7.77	1.15	0.22	-2.86	0.0291	ZDHHC13	zinc finger, DHHC-type containing 13
**3181302**	5.98	7.5	1.22	0.35	-2.85	0.0369	NCBP1	nuclear cap binding protein subunit 1
**3175494**	5.99	7.51	0.28	0.36	-2.85	0.0015	GCNT1	glucosaminyl (N-acetyl) transferase 1, core 2
**3768103**	5.22	6.73	0.59	0.69	-2.85	0.0359	PSMD12	proteasome 26S subunit, non-ATPase 12
**3861413**	7.55	9.07	1.06	0.21	-2.85	0.0292	MAP4K1	mitogen-activated protein kinase kinase kinase kinase 1

**2910236**	4.37	5.88	0.4	0.29	-2.85	0.006	EFHC1	EF-hand domain (C-terminal) containing 1
**3656527**	5.86	7.37	0.89	0.42	-2.85	0.0479	PHKG2	phosphorylase kinase, gamma 2 (testis)
**3466284**	7.08	8.58	0.6	0.46	-2.84	0.0153	NDUFA12	NADH dehydrogenase (ubiquinone) 1 alpha subcomplex, 12
**4021508**	5.26	6.77	1.07	0.19	-2.84	0.0291	ZNF280C	zinc finger protein 280C
**3588069**	7.07	8.58	0.66	0.43	-2.84	0.0318	EMC4	ER membrane protein complex subunit 4
**3721956**	6.41	7.92	0.66	0.28	-2.84	0.0127	TUBG2	tubulin, gamma 2
**2771654**	7.31	8.81	1.01	0.31	-2.84	0.0272	CENPC	centromere protein C
**3289031**	6.78	8.29	0.73	0.31	-2.83	0.0215	TIMM23; TIMM23B	translocase of inner mitochondrial membrane 23 homolog (yeast); translocase of inner mitochondrial membrane 23 homolog B (yeast)
**4009062**	7.57	9.07	0.97	0.37	-2.83	0.0427	KDM5C; MIR6894	lysine (K)-specific demethylase 5C; microRNA 6894
**3096545**	6.29	7.79	0.68	0.11	-2.83	0.0083	POMK	protein-O-mannose kinase
**3362934**	6.55	8.05	1.16	0.55	-2.83	0.024	ZBED5	zinc finger, BED-type containing 5
**2341565**	7.55	9.05	1.34	0.55	-2.83	0.0461	SRSF11	serine/arginine-rich splicing factor 11
**3226253**	7.22	8.71	0.74	0.39	-2.82	0.0121	FAM102A	family with sequence similarity 102, member A
**3647993**	7.67	9.16	0.67	0.16	-2.82	0.0069	CIITA	class II, major histocompatibility complex, transactivator
**3385769**	7.96	9.45	1.14	0.37	-2.82	0.0253	CTSC	cathepsin C
**3755714**	6.62	8.11	1.07	0.08	-2.82	0.0267	MED1	mediator complex subunit 1
**2463515**	5.11	6.6	0.75	0.37	-2.81	0.0146	CHML	choroideremia-like (Rab escort protein 2)
**2877171**	6.19	7.68	1.04	0.17	-2.81	0.04	FAM13B	family with sequence similarity 13, member B
**3748659**	6.77	8.26	0.91	0.53	-2.81	0.0373	GRAP; SNORD3B-1; SNORD3B-2; GRAPL	GRB2-related adaptor protein; small nucleolar RNA, C/D box 3B-1; small nucleolar RNA, C/D box 3B-2; GRB2-related adaptor protein-like
**3608220**	6.33	7.82	1.01	0.15	-2.81	0.0289	CRTC3	CREB regulated transcription coactivator 3
**3181240**	6.82	8.31	0.18	0.33	-2.81	0.001	TMOD1	tropomodulin 1
**3462094**	6.49	7.98	0.94	0.16	-2.81	0.0155	ZFC3H1	zinc finger, C3H1-type containing
**3063337**	6.37	7.86	0.98	0.27	-2.8	0.0398	ZNF394	zinc finger protein 394

**3572235**	5.57	7.05	0.64	0.2	-2.8	0.0061	MLH3	mutL homolog 3
**3361381**	5.03	6.51	0.25	0.44	-2.8	0.0021	CYB5R2	cytochrome b5 reductase 2
**3242353**	4.16	5.64	0.36	0.56	-2.8	0.0371	CREM	cAMP responsive element modulator
**2682436**	5.66	7.14	0.87	0.55	-2.79	0.043	RYBP	RING1 and YY1 binding protein
**3648412**	6.44	7.92	0.65	0.6	-2.79	0.0147	SNX29	sorting nexin 29
**3443206**	7.24	8.73	0.86	0.47	-2.79	0.0106	AICDA	activation-induced cytidine deaminase
**3375340**	6.72	8.2	0.84	0.22	-2.79	0.0215	CPSF7	cleavage and polyadenylation specific factor 7
**3798829**	7.32	8.8	0.62	0.43	-2.79	0.0197	PIEZO2	piezo-type mechanosensitive ion channel component 2
**3478457**	5.9	7.38	0.51	0.18	-2.78	0.0051	STX2	syntaxin 2
**3474228**	7.16	8.63	1.11	0.19	-2.78	0.033	RAB35	RAB35, member RAS oncogene family
**3174224**	6.42	7.9	1.15	0.22	-2.78	0.0463	SMC5	structural maintenance of chromosomes 5
**3994100**	5.8	7.27	0.83	0.29	-2.78	0.0215	FMR1	fragile X mental retardation 1
**3935243**	7.19	8.66	0.72	0.52	-2.77	0.0209	LSS	lanosterol synthase (2,3-oxidosqualene-lanosterol cyclase)
**3538703**	5.01	6.47	0.7	0.3	-2.77	0.0224	MNAT1	MNAT CDK-activating kinase assembly factor 1
**3791168**	6.19	7.66	0.8	0.42	-2.76	0.0233	KIAA1468	KIAA1468
**3726992**	6.26	7.72	0.97	0.6	-2.76	0.0416	UTP18	UTP18 small subunit (SSU) processome component
**2462511**	6.85	8.31	0.91	0.16	-2.76	0.0437	HEATR1	HEAT repeat containing 1
**3331487**	7.1	8.57	0.9	0.15	-2.76	0.015	CTNND1; TMX2; TMX2-CTNND1	catenin (cadherin-associated protein), delta 1; thioredoxin-related transmembrane protein 2; TMX2-CTNND1 readthrough (NMD candidate)
**3946510**	6.12	7.58	0.45	0.53	-2.76	0.0264	XPNPEP3	X-prolyl aminopeptidase 3, mitochondrial
**3826504**	4.62	6.09	0.54	0.52	-2.76	0.0171	ZNF431; RPL36AP51; VN1R82P	zinc finger protein 431; ribosomal protein L36a pseudogene 51; vomeronasal 1 receptor 82 pseudogene
**3534201**	6.36	7.82	0.94	0.54	-2.76	0.0289	PRPF39	pre-mRNA processing factor 39
**3890913**	5.73	7.2	0.84	0.46	-2.76	0.0409	VAPB	VAMP (vesicle-associated membrane protein)-associated protein B and C
**2488038**	7.28	8.74	1	0.31	-2.76	0.0394	NAGK	N-acetylglucosamine kinase

**3699178**	7.03	8.49	0.92	0.4	-2.76	0.0451	WDR59	WD repeat domain 59
**3591044**	3.74	5.2	0.55	0.39	-2.75	0.0163	HAUS2	HAUS augmin like complex subunit 2
**3013178**	5.31	6.77	0.68	0.33	-2.75	0.0466	CASD1	CAS1 domain containing 1
**3236538**	5	6.46	0.83	0.24	-2.75	0.015	RPP38	ribonuclease P/MRP 38kDa subunit
**3239760**	6.9	8.36	0.95	0.26	-2.75	0.0328	APBB1IP	amyloid beta (A4) precursor protein-binding, family B, member 1 interacting protein
**3393200**	6.94	8.4	0.76	0.11	-2.74	0.0113	PCSK7	proprotein convertase subtilisin/kexin type 7
**3267036**	5.85	7.3	0.54	0.49	-2.74	0.012	GRK5; GRK5-IT1	G protein-coupled receptor kinase 5; GRK5 intronic transcript 1
**3954989**	5.34	6.8	1.21	0.54	-2.74	0.0388	DDT; DDTL	D-dopachrome tautomerase; D-dopachrome tautomerase-like
**3758928**	5.54	6.99	0.48	0.39	-2.74	0.0129	ASB16-AS1	ASB16 antisense RNA 1
**3945396**	7.52	8.97	1.17	0.1	-2.74	0.0441	GTPBP1	GTP binding protein 1
**3457696**	6.71	8.16	0.87	0.25	-2.73	0.0129	PAN2	PAN2 poly(A) specific ribonuclease subunit
**4027176**	8.15	9.6	1.08	0.53	-2.73	0.0458	FLNA	filamin A, alpha
**2372141**	4.54	5.99	0.75	0.2	-2.73	0.0371	C1orf27	chromosome 1 open reading frame 27
**3725517**	6.04	7.49	0.16	0.45	-2.73	0.0033	IGF2BP1	insulin-like growth factor 2 mRNA binding protein 1
**2779434**	6.91	8.35	0.96	0.15	-2.73	0.0271	DNAJB14	DnaJ (Hsp40) homolog, subfamily B, member 14
**3946192**	7.33	8.78	0.87	0.29	-2.73	0.0224	TNRC6B	trinucleotide repeat containing 6B
**3383081**	5.36	6.81	0.54	0.34	-2.73	0.0073	INTS4	integrator complex subunit 4
**3513794**	5.72	7.17	0.72	0.2	-2.72	0.0146	RCBTB1	regulator of chromosome condensation (RCC1) and BTB (POZ) domain containing protein 1
**2454485**	6.82	8.26	1.22	0.27	-2.72	0.0406	LPGAT1	lysophosphatidylglycerol acyltransferase 1
**3927226**	7.84	9.29	0.93	0.37	-2.72	0.0292	APP	amyloid beta (A4) precursor protein
**3664952**	5.57	7.02	0.64	0.46	-2.72	0.0199	PDP2	pyruvate dehyrogenase phosphatase catalytic subunit 2
**3300350**	6.33	7.77	0.92	0.47	-2.72	0.0318	IDE	insulin-degrading enzyme
**3884922**	5.86	7.31	0.82	0.46	-2.72	0.0318	DHX35	DEAH (Asp-Glu-Ala-His) box polypeptide 35
**3760268**	5.58	7.03	0.53	1.33	-2.72	0.0177	ARL17B; ARL17A; NBR2	ADP-ribosylation factor like GTPase 17B; ADP-ribosylation factor like GTPase 17A; neighbor of

								BRCA1 gene 2 (non-protein coding)
**3666282**	5.45	6.89	1.11	0.44	-2.72	0.0496	ZFP90	ZFP90 zinc finger protein
**3322775**	11.51	12.95	0.92	0.32	-2.72	0.0438	LDHA	lactate dehydrogenase A
**3754736**	5.41	6.85	0.95	0.2	-2.72	0.0251	DDX52	DEAD (Asp-Glu-Ala-Asp) box polypeptide 52
**4015602**	6.18	7.62	0.77	0.42	-2.72	0.0439	TRMT2B	tRNA methyltransferase 2 homolog B
**2379754**	6.06	7.5	1.02	0.24	-2.71	0.0349	SMYD2	SET and MYND domain containing 2
**3766960**	5.97	7.41	0.97	0.13	-2.71	0.0258	SMURF2	SMAD specific E3 ubiquitin protein ligase 2
**2531310**	6.09	7.53	1.14	0.12	-2.7	0.0435	SP140L; SP140	SP140 nuclear body protein-like; SP140 nuclear body protein
**2947040**	1.97	3.4	0.09	1.12	-2.7	0.0118	HIST1H2AJ	histone cluster 1, H2aj
**2583014**	5.7	7.14	1.01	0.33	-2.7	0.0383	BAZ2B	bromodomain adjacent to zinc finger domain 2B
**3414561**	7.15	8.58	1.02	0.21	-2.7	0.0446	DIP2B	disco-interacting protein 2 homolog B
**3245682**	5.7	7.13	0.91	0.6	-2.7	0.0165	MAPK8	mitogen-activated protein kinase 8
**3565739**	5.44	6.87	0.6	0.41	-2.7	0.0189	ATG14	autophagy related 14
**3933331**	6.92	8.35	0.85	0.38	-2.7	0.0125	C2CD2	C2 calcium-dependent domain containing 2
**2608156**	5.09	6.51	0.84	0.03	-2.69	0.0393	TRNT1	tRNA nucleotidyl transferase, CCA-adding, 1
**3303530**	7.29	8.72	0.47	0.35	-2.69	0.0103	NDUFB8	NADH dehydrogenase (ubiquinone) 1 beta subcomplex, 8, 19kDa
**3469180**	6.18	7.61	0.51	0.26	-2.69	0.0025	SLC41A2	solute carrier family 41 (magnesium transporter), member 2
**3178611**	6.57	8	0.86	0.18	-2.69	0.0191	SECISBP2	SECIS binding protein 2
**3400625**	6.68	8.1	0.42	0.17	-2.69	0.0046	ADIPOR2	adiponectin receptor 2
**3152220**	5.68	7.11	0.98	0.25	-2.68	0.0296	KIAA0196	KIAA0196
**3666779**	7.27	8.69	1.2	0.29	-2.68	0.041	NFAT5	nuclear factor of activated T-cells 5, tonicity-responsive
**3140640**	5.4	6.81	0.58	0.39	-2.67	0.0207	STAU2	staufen double-stranded RNA binding protein 2
**3304012**	7.65	9.07	1.72	0.29	-2.67	0.0465	MGEA5	meningioma expressed antigen 5 (hyaluronidase)
**3840224**	4.16	5.57	0.24	0.21	-2.67	0.0006	ZNF528	zinc finger protein 528
**3522398**	4.98	6.4	0.21	0.31	-2.67	0.0023	DOCK9	dedicator of cytokinesis 9
**3350850**	5.72	7.13	1.04	0.3	-2.67	0.0477	RNF214	ring finger protein 214
**3597977**	5.62	7.03	0.84	0.65	-2.66	0.0466	TRIP4	thyroid hormone receptor interactor 4

**3286921**	5.93	7.34	1.32	0.42	-2.66	0.0473	MARCH-VIII	membrane associated ring finger 8
**3389745**	4.53	5.94	0.63	0.45	-2.66	0.04	CWF19L2	CWF19-like 2, cell cycle control (S. pombe)
**3334137**	7.46	8.87	1.04	0.09	-2.66	0.0354	OTUB1	OTU deubiquitinase, ubiquitin aldehyde binding 1
**3450655**	5.31	6.72	1.17	0.1	-2.65	0.0497	CPNE8	copine VIII
**3361116**	7.8	9.21	0.88	0.36	-2.65	0.0279	MRPL17	mitochondrial ribosomal protein L17
**3466110**	3.65	5.06	0.54	0.24	-2.65	0.0043	CEP83; RBMS2P1	centrosomal protein 83kDa; RNA binding motif, single stranded interacting protein 2 pseudogene 1
**3768015**	6.58	7.98	1.07	0.37	-2.65	0.0384	HELZ	helicase with zinc finger
**3561110**	3.76	5.17	0.5	0.46	-2.65	0.0301	RALGAPA1	Ral GTPase activating protein, alpha subunit 1 (catalytic)
**3832978**	7.22	8.62	0.77	0.55	-2.65	0.0339	ZFP36	ZFP36 ring finger protein
**2774971**	5.73	7.13	0.45	0.54	-2.65	0.0094	ANTXR2	anthrax toxin receptor 2
**3841574**	7.87	9.27	0.89	0.42	-2.65	0.0091	LILRB1	leukocyte immunoglobulin-like receptor, subfamily B (with TM and ITIM domains), member 1
**3154317**	6.22	7.62	0.18	0.54	-2.64	0.0097	NDRG1	N-myc downstream regulated 1
**3662774**	6.77	8.17	0.21	0.55	-2.64	0.019	ADGRG5	adhesion G protein-coupled receptor G5
**3419239**	6.55	7.95	1.07	0.16	-2.64	0.0327	MON2	MON2 homolog, regulator of endosome-to-Golgi trafficking
**3767339**	8.02	9.42	0.73	0.79	-2.64	0.0476	GNA13	guanine nucleotide binding protein (G protein), alpha 13
**3196842**	5.62	7.02	0.76	0.32	-2.64	0.0172	RFX3	regulatory factor X, 3 (influences HLA class II expression)
**2443989**	5.13	6.53	0.64	0.31	-2.64	0.0121	VAMP4	vesicle associated membrane protein 4
**3757770**	7.1	8.5	0.74	0.44	-2.63	0.0401	STAT5B	signal transducer and activator of transcription 5B
**2779823**	7.67	9.07	0.87	0.4	-2.63	0.0489	SLC39A8	solute carrier family 39 (zinc transporter), member 8
**3260829**	5.08	6.47	0.61	0.38	-2.63	0.0333	SLF2	SMC5-SMC6 complex localization factor 2
**3376193**	6.66	8.05	1.03	0.46	-2.63	0.0421	STX5	syntaxin 5
**3625823**	5.18	6.58	0.44	0.22	-2.63	0.0027	ZNF280D	zinc finger protein 280D
**3378411**	7.01	8.4	0.54	0.27	-2.63	0.0053	RBM4B	RNA binding motif protein 4B

**3591674**	5.54	6.93	0.48	0.16	-2.63	0.0122	HYPK; SERF2	huntingtin interacting protein K; small EDRK-rich factor 2
**3269662**	5.38	6.77	0.28	0.57	-2.63	0.0184	BCCIP	BRCA2 and CDKN1A interacting protein
**3628832**	5.28	6.68	0.21	0.19	-2.63	0.0014	DAPK2	death-associated protein kinase 2
**3490251**	6.34	7.74	0.63	0.48	-2.63	0.0496	WDFY2	WD repeat and FYVE domain containing 2
**3458451**	6.67	8.06	1	0.24	-2.62	0.0358	R3HDM2	R3H domain containing 2
**2346863**	5.45	6.85	0.6	0.34	-2.62	0.0174	RPL5; SNORD21; SNORA66	ribosomal protein L5; small nucleolar RNA, C/D box 21; small nucleolar RNA, H/ACA box 66
**2687739**	8.52	9.91	1.02	0.22	-2.62	0.0333	CD47	CD47 molecule
**3136782**	7.03	8.42	1.12	0.38	-2.62	0.0252	NSMAF	neutral sphingomyelinase activation associated factor
**2444283**	5.82	7.21	0.36	0.55	-2.62	0.0143	TNFSF4	tumor necrosis factor (ligand) superfamily, member 4
**3791482**	5.67	7.06	0.47	0.8	-2.62	0.0388	PHLPP1	PH domain and leucine rich repeat protein phosphatase 1
**2377332**	5.47	6.86	0.28	0.47	-2.61	0.0032	CR1	complement component (3b/4b) receptor 1 (Knops blood group)
**3961622**	6.28	7.66	0.73	0.41	-2.61	0.0183	SLC25A17	solute carrier family 25 (mitochondrial carrier; peroxisomal membrane protein, 34kDa), member 17
**3590853**	5.62	7.01	0.49	0.21	-2.61	0.0068	CAPN3; GANC	calpain 3; glucosidase, alpha; neutral C
**3709685**	7.57	8.96	0.48	0.29	-2.61	0.0098	NDEL1	nudE neurodevelopment protein 1-like 1
**3016692**	6.37	7.76	0.55	0.34	-2.61	0.0089	PRKRIP1	PRKR interacting protein 1 (IL11 inducible)
**3843566**	5.39	6.77	0.69	0.27	-2.61	0.0119	ZNF587; ZNF587B; ZSCAN1; ZNF586; UBE2CP5	zinc finger protein 587; zinc finger protein 587B; zinc finger and SCAN domain containing 1; zinc finger protein 586; ubiquitin conjugating enzyme E2C pseudogene 5
**3908831**	7.38	8.76	1.06	0.41	-2.6	0.0482	ZNFX1; KCNB1	zinc finger, NFX1-type containing 1; potassium channel, voltage gated Shab related subfamily B, member 1
**3185522**	7.11	8.49	0.5	0.16	-2.6	0.0054	SLC31A1	solute carrier family 31 (copper transporter), member 1
**4000839**	6.37	7.75	0.77	0.47	-2.6	0.0363	CTPS2	CTP synthase 2

**3458337**	8.24	9.61	1.02	0.28	-2.6	0.0351	STAT6	signal transducer and activator of transcription 6, interleukin-4 induced
**2580943**	3.86	5.24	0.6	0.56	-2.6	0.0302	RBM43	RNA binding motif protein 43
**2659918**	6.85	8.22	1	0.22	-2.59	0.0463	LRCH3	leucine-rich repeats and calponin homology (CH) domain containing 3
**3319898**	5.04	6.41	0.65	0.32	-2.59	0.0208	ZNF143	zinc finger protein 143
**2692136**	4.9	6.28	0.7	0.17	-2.59	0.0421	HSPBAP1	HSPB (heat shock 27kDa) associated protein 1
**3709327**	6.31	7.69	0.55	0.32	-2.59	0.0454	CNTROB	centrobin, centrosomal BRCA2 interacting protein
**2367963**	6.54	7.91	1.08	0.31	-2.59	0.0312	RABGAP1L	RAB GTPase activating protein 1-like
**3418610**	5.25	6.62	0.71	0.16	-2.59	0.04	XRCC6BP1	XRCC6 binding protein 1
**2458513**	7.19	8.56	0.79	0.41	-2.59	0.024	TMEM63A	transmembrane protein 63A
**3283378**	5.9	7.27	0.25	0.36	-2.59	0.005	MTPAP; GOLGA2P6	mitochondrial poly(A) polymerase; golgin A2 pseudogene 6
**3184408**	6.32	7.69	0.86	0.49	-2.58	0.0413	AKAP2; PALM2; PALM2-AKAP2	A kinase (PRKA) anchor protein 2; paralemmin 2; PALM2-AKAP2 readthrough
**3227574**	6.2	7.57	0.58	0.35	-2.58	0.0299	FAM78A	family with sequence similarity 78, member A
**3191900**	7.19	8.56	0.85	0.08	-2.58	0.0204	NUP214	nucleoporin 214kDa
**3473083**	6.39	7.75	0.53	0.38	-2.58	0.0106	MED13L	mediator complex subunit 13-like
**3927949**	5.39	6.75	0.91	0.16	-2.58	0.0225	LTN1	listerin E3 ubiquitin protein ligase 1
**3691967**	4.84	6.2	0.27	0.64	-2.57	0.0203	AKTIP	AKT interacting protein
**3224556**	6.21	7.57	0.58	0.15	-2.57	0.0064	MIR600HG; STRBP	MIR600 host gene; spermatid perinuclear RNA binding protein
**2708066**	6.52	7.88	0.38	0.68	-2.57	0.0437	KLHL6	kelch-like family member 6
**3759540**	6.73	8.09	0.73	0.26	-2.56	0.0362	DCAKD	dephospho-CoA kinase domain containing
**3257559**	6.41	7.76	0.8	0.47	-2.56	0.0393	RPP30	ribonuclease P/MRP 30kDa subunit
**2462160**	6.53	7.89	0.77	0.38	-2.56	0.0349	NID1	nidogen 1
**3685131**	6.18	7.53	0.82	0.31	-2.56	0.0441	COG7	component of oligomeric golgi complex 7
**3139035**	6.25	7.6	0.96	0.3	-2.56	0.0399	ARFGEF1	ADP-ribosylation factor guanine nucleotide-exchange factor 1 (brefeldin A-inhibited)
**3731228**	5.8	7.15	0.9	0.06	-2.55	0.0211	CEP95	centrosomal protein 95kDa

**2950145**	8.15	9.5	1.25	0.16	-2.55	0.0171	HLA-DOB	major histocompatibility complex, class II, DO beta
**3277662**	6.73	8.08	1.03	0.23	-2.55	0.0427	UPF2	UPF2 regulator of nonsense transcripts homolog (yeast)
**3484005**	6.04	7.39	0.82	0.29	-2.55	0.0185	USPL1	ubiquitin specific peptidase like 1
**3716337**	5.6	6.95	0.48	0.07	-2.55	0.0095	NSRP1; MIR423	nuclear speckle splicing regulatory protein 1; microRNA 423
**3806689**	6.77	8.12	0.89	0.27	-2.55	0.0387	HDHD2	haloacid dehalogenase-like hydrolase domain containing 2
**3231846**	5.96	7.31	0.5	0.51	-2.55	0.0404	WDR37; LINC00200	WD repeat domain 37; long intergenic non-protein coding RNA 200
**3711165**	6.31	7.66	0.72	0.25	-2.54	0.0336	COX10	COX10 heme A:farnesyltransferase cytochrome c oxidase assembly factor
**3487220**	6.6	7.95	0.73	0.43	-2.54	0.0428	AKAP11	A kinase (PRKA) anchor protein 11
**3513953**	5.27	6.61	0.49	0.61	-2.54	0.0385	SPRYD7	SPRY domain containing 7
**3828949**	5.15	6.5	0.66	0.16	-2.54	0.0099	DPY19L3	dpy-19-like 3 (C. elegans)
**3772525**	7.97	9.32	1.34	0.42	-2.54	0.0463	CYTH1	cytohesin 1
**3751463**	7.49	8.84	0.98	0.31	-2.54	0.0359	NUFIP2	nuclear fragile X mental retardation protein interacting protein 2
**3771037**	6.71	8.06	0.82	0.11	-2.54	0.0232	WBP2	WW domain binding protein 2
**3453218**	6.59	7.94	0.92	0.12	-2.54	0.0271	CCNT1	cyclin T1
**3662650**	6.2	7.54	0.91	0.29	-2.54	0.0438	ARL2BP	ADP-ribosylation factor like GTPase 2 binding protein
**3527662**	6.52	7.86	0.33	0.69	-2.53	0.0177	RNASE6	ribonuclease, RNase A family, k6
**3942384**	6.19	7.53	0.58	0.55	-2.53	0.0484	MTFP1	mitochondrial fission process 1
**3190035**	7.86	9.2	0.87	0.46	-2.53	0.0353	CDK9; MIR2861; MIR3960	cyclin-dependent kinase 9; microRNA 2861; microRNA 3960
**3853345**	7.47	8.8	0.86	0.3	-2.52	0.0314	AKAP8L	A kinase (PRKA) anchor protein 8-like
**3957003**	6.59	7.92	0.96	0.24	-2.52	0.0261	ASCC2	activating signal cointegrator 1 complex subunit 2
**2523213**	6.57	7.91	0.92	0.21	-2.52	0.0322	BMPR2	bone morphogenetic protein receptor type II
**3582745**	5.5	6.83	0.24	0.74	-2.52	0.0041	MIR5195; IGHV5-78	microRNA 5195; immunoglobulin heavy variable 5-78 (pseudogene)
**3762625**	5.24	6.58	0.56	0.34	-2.52	0.0069	MBTD1	mbt domain containing 1

**3804143**	6.27	7.6	0.92	0.18	-2.52	0.0327	RPRD1A	regulation of nuclear pre-mRNA domain containing 1A
**3408018**	6.76	8.09	0.69	0.35	-2.52	0.0169	ETNK1	ethanolamine kinase 1
**3354174**	7.14	8.47	0.75	0.14	-2.52	0.0128	TBRG1	transforming growth factor beta regulator 1
**3768969**	5.62	6.95	0.53	0.43	-2.51	0.0038	ABCA5	ATP binding cassette subfamily A member 5
**2646327**	6.85	8.17	0.37	0.61	-2.51	0.049	C3orf58	chromosome 3 open reading frame 58
**4003954**	6.42	7.75	0.98	0.12	-2.51	0.0351	TAB3	TGF-beta activated kinase 1/MAP3K7 binding protein 3
**3544678**	5.99	7.32	0.59	0.28	-2.51	0.0117	TTLL5; FLVCR2; IFT43	tubulin tyrosine ligase-like family member 5; feline leukemia virus subgroup C cellular receptor family, member 2; intraflagellar transport 43
**3831774**	5.44	6.77	0.82	0.15	-2.51	0.0211	ZNF383	zinc finger protein 383
**2357996**	6.17	7.5	0.84	0.33	-2.51	0.0477	VPS45	vacuolar protein sorting 45 homolog (S. cerevisiae)
**3379326**	6.26	7.59	0.8	0.27	-2.51	0.0146	CHKA	choline kinase alpha
**2654306**	5.97	7.29	0.98	0.24	-2.51	0.0465	TTC14	tetratricopeptide repeat domain 14
**3784344**	6.15	7.48	0.8	0.37	-2.51	0.0467	MAPRE2	microtubule-associated protein, RP/EB family, member 2
**3948047**	7.6	8.92	0.99	0.35	-2.5	0.0368	PARVG; PARVB	parvin, gamma; parvin, beta
**3899346**	7.52	8.85	0.57	0.27	-2.5	0.0478	SNX5; OVOL2	sorting nexin 5; ovo-like zinc finger 2
**3306299**	6.36	7.68	0.51	0.43	-2.5	0.0447	XPNPEP1	X-prolyl aminopeptidase (aminopeptidase P) 1, soluble
**4019160**	3.88	5.2	0.23	0.75	-2.5	0.0179	KLHL13	kelch-like family member 13
**3560673**	5.58	6.9	0.53	0.71	-2.5	0.0207	CFL2; SEPT7P1	cofilin 2 (muscle); septin 7 pseudogene 1
**3577870**	7.2	8.52	1.03	0.12	-2.5	0.0383	DICER1	dicer 1, ribonuclease type III
**3923436**	6.86	8.18	0.96	0.23	-2.5	0.0313	TRAPPC10	trafficking protein particle complex 10
**3567469**	4.85	6.17	0.6	0.27	-2.5	0.0204	TRMT5	tRNA methyltransferase 5
**3872521**	5.61	6.93	0.67	0.95	-2.5	0.0363	ZNF417	zinc finger protein 417
**3405396**	5.79	7.11	0.88	0.27	-2.49	0.0482	CREBL2	cAMP responsive element binding protein-like 2
**3010082**	6.14	7.46	0.9	0.26	-2.49	0.0439	PHTF2	putative homeodomain transcription factor 2
**3580498**	5.9	7.22	0.56	0.32	-2.49	0.0389	CDC42BPB	CDC42 binding protein kinase beta (DMPK-like)

**2416522**	7.42	8.74	0.92	0.66	-2.49	0.0469	JAK1; LINC01359	Janus kinase 1; long intergenic non-protein coding RNA 1359
**4000132**	2.48	3.8	0.58	0.5	-2.49	0.0443	TRAPPC2	trafficking protein particle complex 2
**3288707**	5.4	6.72	0.45	0.18	-2.49	0.004	ERCC6; ERCC6-PGBD3; PGBD3	excision repair cross-complementation group 6; ERCC6-PGBD3 readthrough; piggyBac transposable element derived 3
**3884640**	6.97	8.28	1.05	0.31	-2.48	0.0444	RALGAPB; RPS3P2	Ral GTPase activating protein, beta subunit (non-catalytic); ribosomal protein S3 pseudogene 2
**3665288**	6.89	8.2	1.21	0.37	-2.48	0.0478	E2F4	E2F transcription factor 4, p107/p130-binding
**3261165**	6.04	7.35	0.49	0.56	-2.48	0.0444	BTRC	beta-transducin repeat containing E3 ubiquitin protein ligase
**3303913**	7.28	8.59	0.43	0.39	-2.48	0.04	FBXW4	F-box and WD repeat domain containing 4
**3376235**	6.89	8.2	1.15	0.46	-2.48	0.049	WDR74; RNU2-1	WD repeat domain 74; RNA, U2 small nuclear 1
**3454296**	6.71	8.02	0.63	0.3	-2.48	0.0201	CERS5	ceramide synthase 5
**3708462**	7.56	8.87	1.01	0.07	-2.47	0.0431	ACAP1	ArfGAP with coiled-coil, ankyrin repeat and PH domains 1
**3081613**	4.75	6.06	0.85	0.5	-2.47	0.0169	LMBR1	limb development membrane protein 1
**3322717**	5.35	6.65	0.48	0.29	-2.47	0.0045	GTF2H1	general transcription factor IIH subunit 1
**3494137**	5.15	6.46	0.47	0.59	-2.47	0.0145	LMO7	LIM domain 7
**3557614**	7.73	9.03	0.75	0.39	-2.47	0.0205	AP1G2	adaptor-related protein complex 1, gamma 2 subunit
**3527493**	7.46	8.77	0.76	0.24	-2.47	0.0394	APEX1	APEX nuclease (multifunctional DNA repair enzyme) 1
**3214984**	6.31	7.61	0.6	0.43	-2.47	0.0426	BICD2	bicaudal D homolog 2 (Drosophila)
**3362795**	5.29	6.6	0.51	0.58	-2.47	0.0474	RNF141	ring finger protein 141
**3855410**	7.04	8.34	1.23	0.11	-2.47	0.0449	SUGP2	SURP and G-patch domain containing 2
**3224366**	6.07	7.37	1.21	0.29	-2.47	0.042	RC3H2	ring finger and CCCH-type domains 2
**3835467**	6.05	7.36	0.88	0.18	-2.47	0.0281	ZNF234	zinc finger protein 234
**3969455**	6.29	7.59	0.79	0.26	-2.47	0.0436	OFD1	oral-facial-digital syndrome 1
**4041113**	4.27	5.58	0.47	0.6	-2.46	0.0379	KPNA2	karyopherin alpha 2 (RAG cohort 1, importin alpha 1)
**3896034**	7.07	8.37	0.98	0.45	-2.46	0.0431	RASSF2	Ras association (RalGDS/AF-6) domain family member 2

**3572461**	8.15	9.45	0.7	0.6	-2.46	0.0125	C14orf1	chromosome 14 open reading frame 1
**2653673**	4.72	6.02	0.31	0.35	-2.46	0.0053	KCNMB2	potassium channel subfamily M regulatory beta subunit 2
**3471819**	6.32	7.62	0.84	0.02	-2.46	0.022	NAA25	N(alpha)-acetyltransferase 25, NatB auxiliary subunit
**2882098**	6.73	8.03	0.34	0.29	-2.46	0.006	SPARC	secreted protein, acidic, cysteine-rich (osteonectin)
**2922631**	7.06	8.36	0.95	0.25	-2.46	0.0459	DSE	dermatan sulfate epimerase
**4020444**	6.58	7.87	1.16	0.21	-2.46	0.0421	THOC2	THO complex 2
**3233322**	6.32	7.61	0.58	0.17	-2.46	0.011	FAM208B	family with sequence similarity 208, member B
**3639406**	7.57	8.86	0.15	0.41	-2.45	0.0101	FAM174B	family with sequence similarity 174, member B
**2609960**	6.43	7.73	0.81	0.15	-2.45	0.036	ARPC4; TTLL3; ARPC4-TTLL3	actin related protein 2/3 complex subunit 4; tubulin tyrosine ligase-like family member 3; ARPC4-TTLL3 readthrough
**2954025**	6.45	7.74	0.38	0.2	-2.45	0.0042	TRERF1	transcriptional regulating factor 1
**3471588**	6.63	7.92	0.9	0.21	-2.44	0.0336	ATXN2	ataxin 2
**2544484**	7.87	9.16	0.87	0.37	-2.44	0.045	ADCY3	adenylate cyclase 3
**3831588**	2.73	4.02	0.26	0.09	-2.44	0.0014	ZNF345; RPL31P61	zinc finger protein 345; ribosomal protein L31 pseudogene 61
**2749380**	3.51	4.79	0.36	0.19	-2.44	0.0084	TMEM144	transmembrane protein 144
**4013460**	5.68	6.97	0.06	0.64	-2.43	0.0065	CYSLTR1	cysteinyl leukotriene receptor 1
**3913483**	6.1	7.38	0.85	0.34	-2.43	0.0358	TCFL5	transcription factor-like 5 (basic helix-loop-helix)
**3525538**	7.08	8.36	0.73	0.57	-2.43	0.0356	CARS2	cysteinyl-tRNA synthetase 2, mitochondrial (putative)
**3145149**	6.9	8.18	0.93	0.19	-2.43	0.0175	TP53INP1	tumor protein p53 inducible nuclear protein 1
**3444195**	7.34	8.62	0.63	0.37	-2.43	0.022	MAGOHB	mago homolog B, exon junction complex core component
**3593339**	5.37	6.65	0.8	0.58	-2.43	0.0471	GALK2; NDUFAF4P1	galactokinase 2; NADH dehydrogenase (ubiquinone) complex I, assembly factor 4 pseudogene 1
**3487432**	5.66	6.94	0.79	0.34	-2.43	0.0385	DNAJC15	DnaJ (Hsp40) homolog, subfamily C, member 15
**2560286**	6.1	7.38	0.19	0.09	-2.43	0.0012	LOXL3	lysyl oxidase-like 3
**3823982**	7.75	9.03	0.97	0.11	-2.42	0.0374	MYO9B	myosin IXB
**3470193**	4.38	5.65	0.3	0.45	-2.42	0.0082	CMKLR1	chemerin chemokine-like receptor 1

**2690850**	7.11	8.38	0.65	0.3	-2.41	0.0236	TMEM39A	transmembrane protein 39A
**3727510**	4.47	5.74	0.69	0.24	-2.41	0.0295	STXBP4	syntaxin binding protein 4
**3739962**	7	8.27	0.89	0.16	-2.41	0.0334	ABR	active BCR-related
**2886595**	6.67	7.94	0.64	0.15	-2.41	0.0181	LCP2	lymphocyte cytosolic protein 2
**3896078**	6.34	7.6	0.4	0.39	-2.4	0.0126	SLC23A2	solute carrier family 23 (ascorbic acid transporter), member 2
**3929664**	5.61	6.87	0.35	0.4	-2.4	0.0325	TMEM50B	transmembrane protein 50B
**3331730**	6.39	7.65	0.73	0.18	-2.4	0.0219	CNTF; ZFP91; ZFP91-CNTF	ciliary neurotrophic factor; ZFP91 zinc finger protein; ZFP91-CNTF readthrough (NMD candidate)
**3464622**	4.01	5.27	0.31	0.69	-2.4	0.0434	CEP290	centrosomal protein 290kDa
**3235414**	6.17	7.43	0.69	0.19	-2.4	0.0102	SEC61A2	Sec61 translocon alpha 2 subunit
**3766284**	5.97	7.23	0.66	0.17	-2.39	0.0213	STRADA	STE20-related kinase adaptor alpha
**3812385**	8.2	9.46	0.96	0.2	-2.39	0.0335	CD226	CD226 molecule
**2576554**	7.69	8.95	0.78	0.46	-2.39	0.0416	MZT2A	mitotic spindle organizing protein 2A
**3808854**	6.6	7.85	0.78	0.26	-2.39	0.0165	TCF4	transcription factor 4
**2780099**	6.39	7.65	0.82	0.07	-2.39	0.038	SLC9B2	solute carrier family 9, subfamily B (NHA2, cation proton antiporter 2), member 2
**3256074**	6.84	8.09	0.68	0.36	-2.39	0.0118	BMPR1A	bone morphogenetic protein receptor type IA
**2957227**	6.86	8.11	0.6	0.5	-2.38	0.0214	TRAM2	translocation associated membrane protein 2
**3774535**	7.47	8.72	0.66	0.21	-2.38	0.0489	DCXR	dicarbonyl/L-xylulose reductase
**3468743**	6.16	7.41	0.67	0.45	-2.38	0.0378	NT5DC3	5-nucleotidase domain containing 3
**3741875**	6.86	8.11	0.81	0.26	-2.38	0.0327	ZZEF1	zinc finger, ZZ-type with EF-hand domain 1
**3593452**	2.51	3.76	0.36	0.11	-2.38	0.0069	DTWD1	DTW domain containing 1
**2324634**	6.75	8	0.74	0.18	-2.38	0.0143	CDC42	cell division cycle 42
**3607698**	6.13	7.38	0.68	0.15	-2.38	0.0159	TICRR	TOPBP1-interacting checkpoint and replication regulator
**3354443**	6.64	7.89	0.34	0.51	-2.38	0.0105	SLC37A2	solute carrier family 37 (glucose-6-phosphate transporter), member 2
**3500772**	6.29	7.54	0.72	0.31	-2.38	0.0198	ABHD13	abhydrolase domain containing 13
**3980758**	7.43	8.68	1.02	0.12	-2.37	0.0434	MED12	mediator complex subunit 12
**2940145**	5.19	6.43	0.29	0.86	-2.37	0.0367	NRN1	neuritin 1

**2579439**	5.41	6.66	0.52	0.44	-2.37	0.0414	GTDC1	glycosyltransferase like domain containing 1
**3849797**	4.79	6.03	0.57	0.33	-2.37	0.0428	ZNF561	zinc finger protein 561
**2499234**	3.86	5.1	0.49	0.43	-2.37	0.037	CCDC138	coiled-coil domain containing 138
**3336422**	7.41	8.65	0.96	0.38	-2.37	0.0465	RBM4; RBM14-RBM4	RNA binding motif protein 4; RBM14-RBM4 readthrough
**2461999**	5.32	6.56	0.78	0.22	-2.37	0.0276	LYST	lysosomal trafficking regulator
**3318712**	6.98	8.22	1.12	0.29	-2.36	0.0408	TIMM10B	translocase of inner mitochondrial membrane 10 homolog B (yeast)
**3752271**	5.17	6.41	0.28	0.14	-2.36	0.0017	EVI2A	ecotropic viral integration site 2A
**3417075**	7.12	8.36	0.68	0.18	-2.36	0.0074	DGKA	diacylglycerol kinase alpha
**2915268**	5.95	7.19	0.78	0.25	-2.36	0.0378	DOPEY1	dopey family member 1
**3534128**	4.84	6.08	0.32	0.27	-2.36	0.0022	FAM179B	family with sequence similarity 179, member B
**3902682**	6.17	7.41	0.86	0.19	-2.36	0.034	PLAGL2	pleiomorphic adenoma gene-like 2
**3507465**	4.88	6.11	0.29	0.59	-2.36	0.0027	SLC46A3; RNU6-53P	solute carrier family 46, member 3; RNA, U6 small nuclear 53, pseudogene
**3317915**	7.76	9	0.99	0.14	-2.36	0.0322	STIM1; RPS29P20	stromal interaction molecule 1; ribosomal protein S29 pseudogene 20
**3869379**	4.79	6.02	0.69	0.35	-2.36	0.0348	ZNF614	zinc finger protein 614
**3484060**	7.93	9.16	0.5	0.77	-2.35	0.0191	ALOX5AP; LINC00398	arachidonate 5-lipoxygenase-activating protein; long intergenic non-protein coding RNA 398
**3181728**	7.02	8.25	0.76	0.47	-2.35	0.0216	TGFBR1	transforming growth factor, beta receptor 1
**3966225**	7.25	8.49	0.79	0.47	-2.35	0.013	RABL2B	RAB, member of RAS oncogene family-like 2B
**3529701**	8	9.23	0.96	0.26	-2.35	0.0344	IRF9	interferon regulatory factor 9
**3642815**	6.91	8.15	0.8	0.28	-2.35	0.0357	NME4	NME/NM23 nucleoside diphosphate kinase 4
**3216476**	4.79	6.02	0.38	0.18	-2.35	0.0041	ZNF510	zinc finger protein 510
**3764066**	7.23	8.46	0.55	0.37	-2.35	0.013	VEZF1	vascular endothelial zinc finger 1
**3154700**	6.7	7.93	0.77	0.13	-2.35	0.0254	ZFAT; MIR30D; MIR30B	zinc finger and AT hook domain containing; microRNA 30d; microRNA 30b
**3743119**	5.34	6.57	0.77	0.41	-2.35	0.0351	KIAA0753	KIAA0753
**3766861**	6.45	7.68	0.47	0.52	-2.35	0.0269	POLG2	polymerase (DNA directed), gamma 2, accessory subunit

**3559794**	5.33	6.56	0.49	0.23	-2.34	0.0177	DTD2	D-tyrosyl-tRNA deacylase 2 (putative)
**3257031**	5.5	6.73	0.54	0.29	-2.34	0.0091	STAMBPL1; FAS	STAM binding protein-like 1; Fas cell surface death receptor
**3887165**	6.58	7.8	0.61	0.25	-2.34	0.0188	PCIF1	PDX1 C-terminal inhibiting factor 1
**3833040**	6.6	7.83	0.74	0.34	-2.33	0.0397	SUPT5H	SPT5 homolog, DSIF elongation factor subunit
**3386737**	5.8	7.03	0.5	0.38	-2.33	0.0159	SMCO4	single-pass membrane protein with coiled-coil domains 4
**3947310**	6.43	7.65	0.99	0.12	-2.33	0.0488	SMDT1; NDUFA6-AS1	single-pass membrane protein with aspartate-rich tail 1; NDUFA6 antisense RNA 1 (head to head)
**3674840**	8.07	9.29	0.58	0.34	-2.33	0.017	POLR3K	polymerase (RNA) III (DNA directed) polypeptide K, 12.3 kDa
**3655140**	7	8.23	0.63	0.3	-2.33	0.0323	NFATC2IP	nuclear factor of activated T-cells, cytoplasmic, calcineurin-dependent 2 interacting protein
**3944543**	6.11	7.33	0.33	0.29	-2.33	0.003	NCF4	neutrophil cytosolic factor 4
**3601675**	5.95	7.17	0.38	0.3	-2.33	0.0164	ARID3B	AT rich interactive domain 3B (BRIGHT-like)
**3632298**	7.1	8.32	0.89	0.23	-2.33	0.0325	ADPGK	ADP-dependent glucokinase
**3426502**	7.39	8.61	0.85	0.25	-2.33	0.0216	PLXNC1	plexin C1
**3816153**	7.89	9.1	0.97	0.24	-2.33	0.035	CSNK1G2	casein kinase 1, gamma 2
**3699581**	7.26	8.48	0.38	0.32	-2.32	0.008	TMEM170A; CHST6	transmembrane protein 170A; carbohydrate (N-acetylglucosamine 6-O) sulfotransferase 6
**3535125**	5.8	7.02	0.29	0.25	-2.32	0.0037	ATP5S	ATP synthase, H+ transporting, mitochondrial Fo complex subunit s (factor B)
**3707258**	7.07	8.28	0.76	0.14	-2.32	0.0331	MINK1	misshapen-like kinase 1
**3975762**	6.43	7.65	1.33	0.51	-2.32	0.0339	KRBOX4	KRAB box domain containing 4
**3259631**	6.14	7.36	0.35	0.33	-2.32	0.0089	LCOR	ligand dependent nuclear receptor corepressor
**3006133**	5.31	6.53	0.53	0.42	-2.32	0.0301	STAG3L4	stromal antigen 3-like 4 (pseudogene)
**3903525**	6.37	7.58	0.42	0.13	-2.32	0.0038	NCOA6; PIGU	nuclear receptor coactivator 6; phosphatidylinositol glycan anchor biosynthesis class U
**3325263**	5.19	6.4	0.43	0.16	-2.32	0.0053	DNAJC24	DnaJ (Hsp40) homolog, subfamily C, member 24

**3709244**	7.16	8.37	0.83	0.22	-2.32	0.049	CHD3; SCARNA21	chromodomain helicase DNA binding protein 3; small Cajal body-specific RNA 21
**3387413**	6.22	7.43	0.5	0.23	-2.31	0.0138	FAM76B	family with sequence similarity 76, member B
**3883941**	7.32	8.53	0.7	0.33	-2.31	0.0465	TGIF2; TGIF2-C20orf24	TGFB-induced factor homeobox 2; TGIF2-C20orf24 readthrough
**3641871**	5.83	7.04	0.44	0.26	-2.31	0.0315	LINS1	lines homolog 1
**3845944**	8.02	9.23	0.58	0.11	-2.31	0.0104	GNG7	guanine nucleotide binding protein (G protein), gamma 7
**3450180**	5.01	6.21	0.36	0.57	-2.31	0.03	YARS2	tyrosyl-tRNA synthetase 2, mitochondrial
**3721718**	3.45	4.66	0.71	0.3	-2.31	0.0289	ATP6V0A1	ATPase, H+ transporting, lysosomal V0 subunit a1
**2328990**	3.72	4.93	0.27	0.32	-2.31	0.0047	RBBP4	retinoblastoma binding protein 4
**3343202**	6.79	7.99	0.82	0.36	-2.31	0.0381	EED; MIR6755	embryonic ectoderm development; microRNA 6755
**3511698**	6.77	7.97	1.39	0.34	-2.31	0.0489	EPSTI1	epithelial stromal interaction 1 (breast)
**3882533**	5.2	6.4	0.72	0.19	-2.31	0.03	CBFA2T2	core-binding factor, runt domain, alpha subunit 2; translocated to, 2
**3487095**	6.21	7.41	0.85	0.5	-2.31	0.0369	DGKH	diacylglycerol kinase, eta
**3308864**	6.19	7.39	1.06	0.21	-2.3	0.0116	RAB11FIP2	RAB11 family interacting protein 2 (class I)
**3589212**	4.43	5.63	0.33	0.5	-2.3	0.0358	FAM98B	family with sequence similarity 98, member B
**3309124**	6.82	8.02	0.56	0.24	-2.3	0.0111	CACUL1	CDK2-associated, cullin domain 1
**3384417**	5.35	6.56	0.42	0.5	-2.3	0.016	PCF11-AS1	PCF11 anstisense RNA 1
**3828032**	5.59	6.8	0.88	0.16	-2.3	0.0466	POP4	POP4 homolog, ribonuclease P/MRP subunit
**3557268**	6.03	7.23	0.8	0.17	-2.3	0.0217	PPP1R3E; HOMEZ	protein phosphatase 1, regulatory subunit 3E; homeobox and leucine zipper encoding
**2440295**	6.76	7.96	0.29	0.49	-2.3	0.026	CD84	CD84 molecule
**2877028**	6.1	7.3	0.53	0.25	-2.3	0.0195	KLHL3	kelch-like family member 3
**3508644**	5.29	6.49	0.57	0.64	-2.3	0.0377	N4BP2L1	NEDD4 binding protein 2-like 1
**3960478**	7.57	8.77	0.65	0.21	-2.3	0.0152	CSNK1E	casein kinase 1, epsilon
**2358171**	7.57	8.77	1.03	0.5	-2.29	0.0448	PRPF3	pre-mRNA processing factor 3
**3377044**	8.33	9.52	0.86	0.3	-2.29	0.0424	SF1	splicing factor 1

**3716579**	5.12	6.32	0.79	0.13	-2.29	0.0259	LRRC37BP1; SH3GL1P2	leucine rich repeat containing 37B pseudogene 1; SH3-domain GRB2-like 1 pseudogene 2
**3525679**	7.49	8.69	0.9	0.28	-2.29	0.0443	ANKRD10; ANKRD10-IT1	ankyrin repeat domain 10; ANKRD10 intronic transcript 1
**3957589**	6.11	7.3	0.84	0.11	-2.29	0.0296	MORC2	MORC family CW-type zinc finger 2
**3937587**	7.35	8.55	0.7	0.18	-2.29	0.024	MED15	mediator complex subunit 15
**3233605**	6.69	7.89	0.66	0.47	-2.29	0.0408	PFKFB3	6-phosphofructo-2-kinase/fructose-2,6-biphosphatase 3
**3762519**	6.35	7.54	0.98	0.39	-2.29	0.0432	SPAG9	sperm associated antigen 9
**2716467**	5.7	6.89	0.22	0.3	-2.28	0.001	NSG1; LOC105374360	neuron specific gene family member 1; uncharacterized LOC105374360
**3352948**	8.86	10.05	1.43	0.16	-2.28	0.0466	SORL1	sortilin-related receptor, L(DLR class) A repeats containing
**3174429**	5.22	6.4	0.8	0.13	-2.28	0.0258	C9orf85	chromosome 9 open reading frame 85
**3879372**	5.08	6.27	0.82	0.25	-2.28	0.0338	KIZ	kizuna centrosomal protein
**3530982**	5.23	6.41	0.6	0.68	-2.27	0.0406	G2E3	G2/M-phase specific E3 ubiquitin protein ligase
**3337516**	6.55	7.74	0.46	0.2	-2.27	0.0311	LRP5	LDL receptor related protein 5
**4024160**	6.69	7.87	1.13	0.34	-2.27	0.0457	ATP11C	ATPase, class VI, type 11C
**3630701**	7.13	8.31	0.37	0.26	-2.27	0.0145	CLN6	ceroid-lipofuscinosis, neuronal 6, late infantile, variant
**3823583**	7.38	8.57	1.08	0.3	-2.27	0.0484	HSH2D	hematopoietic SH2 domain containing
**2841491**	6.04	7.22	0.9	0.36	-2.27	0.0322	CREBRF	CREB3 regulatory factor
**2321238**	6.61	7.79	0.9	0.04	-2.27	0.0477	PRDM2; KAZN	PR domain containing 2, with ZNF domain; kazrin, periplakin interacting protein
**3860208**	7.24	8.43	0.64	0.12	-2.27	0.0245	ALKBH6	alkB homolog 6
**3961664**	5.72	6.9	0.74	0.41	-2.27	0.027	ST13	suppression of tumorigenicity 13 (colon carcinoma) (Hsp70 interacting protein)
**3077573**	2.82	4	0.22	0.36	-2.27	0.0176	ARHGEF35	Rho guanine nucleotide exchange factor 35
**3750842**	5.85	7.03	0.69	0.1	-2.26	0.0256	SGK494	uncharacterized serine/threonine-protein kinase SgK494
**3562671**	6.11	7.29	0.43	0.51	-2.26	0.0139	KLHL28	kelch-like family member 28

**2497119**	4.39	5.57	0.36	0.1	-2.26	0.0067	IL18R1	interleukin 18 receptor 1
**2958861**	5.44	6.62	0.84	0.24	-2.26	0.018	LINC00680; GUSBP4; GUSBP1	long intergenic non-protein coding RNA 680; glucuronidase, beta pseudogene 4; glucuronidase, beta pseudogene 1
**3352130**	6.89	8.06	0.68	0.15	-2.26	0.0175	RNF26	ring finger protein 26
**3843848**	5.78	6.95	0.47	0.29	-2.26	0.0148	ZNF544	zinc finger protein 544
**3996404**	8.3	9.47	1.1	0.14	-2.25	0.034	GDI1	GDP dissociation inhibitor 1
**3456353**	6.5	7.67	0.55	0.33	-2.25	0.0302	CALCOCO1; CISTR	calcium binding and coiled-coil domain 1; chondrogenesis-associated transcript
**3838067**	7.22	8.39	0.68	0.31	-2.25	0.0462	BAX	BCL2-associated X protein
**3547375**	6.07	7.24	0.71	0.39	-2.25	0.0162	GPR65	G protein-coupled receptor 65
**3653398**	6.58	7.75	0.9	0.1	-2.25	0.0267	TNRC6A	trinucleotide repeat containing 6A
**3789442**	5.74	6.91	0.75	0.36	-2.25	0.0338	WDR7	WD repeat domain 7
**3491948**	5.71	6.88	0.69	0.35	-2.25	0.0256	TDRD3	tudor domain containing 3
**3382319**	6.71	7.87	0.28	0.03	-2.24	0.0016	GDPD5; KLHL35	glycerophosphodiester phosphodiesterase domain containing 5; kelch-like family member 35
**3414512**	6.67	7.84	0.76	0.55	-2.24	0.0489	LARP4	La ribonucleoprotein domain family, member 4
**3686587**	6.88	8.04	0.75	0.45	-2.24	0.0464	SGF29	SAGA complex associated factor 29
**3881874**	7.43	8.6	0.83	0.37	-2.24	0.0468	ASXL1	additional sex combs like transcriptional regulator 1
**3755396**	6.35	7.52	0.74	0.38	-2.24	0.0467	CWC25; C17orf98	CWC25 spliceosome-associated protein homolog; chromosome 17 open reading frame 98
**3853453**	8.02	9.18	0.79	0.16	-2.24	0.0328	RASAL3	RAS protein activator like 3
**3524618**	7.78	8.94	1.19	0.16	-2.24	0.032	ARGLU1	arginine and glutamate rich 1
**2350952**	5.96	7.12	0.84	0.36	-2.24	0.0399	GSTM2; GSTM4; GSTM1; ERVK11-1	glutathione S-transferase mu 2 (muscle); glutathione S-transferase mu 4; glutathione S-transferase mu 1; endogenous retrovirus group K11, member 1
**3645816**	5.66	6.82	0.69	0.12	-2.24	0.0176	ZNF75A	zinc finger protein 75a
**3820370**	7.2	8.36	0.38	0.76	-2.23	0.0383	PPAN-P2RY11; P2RY11	PPAN-P2RY11 readthrough; purinergic receptor P2Y, G-protein coupled, 11

**2986999**	5.01	6.17	0.4	0.44	-2.23	0.0186	GPR146	G protein-coupled receptor 146
**2735459**	5.95	7.11	0.29	0.43	-2.23	0.0098	HERC3	HECT and RLD domain containing E3 ubiquitin protein ligase 3
**3416996**	6.45	7.61	0.46	0.49	-2.23	0.0394	TMEM198B	transmembrane protein 198B, pseudogene
**3132016**	6.76	7.92	0.55	0.4	-2.23	0.0357	FGFR1; RPS20P22	fibroblast growth factor receptor 1; ribosomal protein S20 pseudogene 22
**3014904**	7.45	8.61	0.86	0.09	-2.23	0.0344	ZNF655	zinc finger protein 655
**3958045**	6.66	7.82	1.09	0.19	-2.23	0.046	PRR14L	proline rich 14-like
**3432138**	6.95	8.1	0.93	0.14	-2.23	0.0377	MAPKAPK5	mitogen-activated protein kinase-activated protein kinase 5
**3430086**	4.93	6.09	0.36	0.25	-2.23	0.0085	TCP11L2	t-complex 11, testis-specific-like 2
**3570475**	5.17	6.32	0.7	0.16	-2.23	0.0322	SYNJ2BP-COX16; SYNJ2BP	SYNJ2BP-COX16 readthrough; synaptojanin 2 binding protein
**3695199**	6.31	7.46	0.78	0.53	-2.23	0.0368	DYNC1LI2	dynein, cytoplasmic 1, light intermediate chain 2
**3543355**	6.06	7.22	0.46	0.31	-2.23	0.0224	DCAF4	DDB1 and CUL4 associated factor 4
**2760632**	5.06	6.21	0.54	0.21	-2.22	0.0348	CLNK	cytokine-dependent hematopoietic cell linker
**2803329**	7.52	8.67	0.54	0.25	-2.22	0.0233	BASP1	brain abundant, membrane attached signal protein 1
**3386814**	6.18	7.33	1.17	0.13	-2.22	0.0427	TAF1D; SNORA8; SNORA18; SNORA40; SNORA1; SNORA25; SNORA32; SNORD5; SNORD6	TATA box binding protein associated factor 1D; small nucleolar RNA, H/ACA box 8; small nucleolar RNA, H/ACA box 18; small nucleolar RNA, H/ACA box 40; small nucleolar RNA, H/ACA box 1; small nucleolar RNA, H/ACA box 25; small nucleolar RNA, H/ACA box 32; small nucleolar RNA, C/D box 5; small nucleolar RNA, C/D box 6
**3544251**	6.27	7.42	0.89	0.23	-2.22	0.045	YLPM1	YLP motif containing 1
**3557106**	6.71	7.86	0.84	0.26	-2.22	0.0468	ACIN1	apoptotic chromatin condensation inducer 1
**3511189**	5.19	6.34	0.83	0.37	-2.22	0.0331	MTRF1	mitochondrial translational release factor 1
**3717452**	4.31	5.46	0.25	0.27	-2.22	0.003	LRRC37B; SH3GL1P1	leucine rich repeat containing 37B; SH3-domain GRB2-like 1 pseudogene 1
**3954525**	5.07	6.22	0.21	0.26	-2.22	0.0056	ZNF280B	zinc finger protein 280B

**2621583**	6.56	7.71	0.91	0.33	-2.22	0.0387	ZNF589	zinc finger protein 589
**3463112**	6.91	8.06	0.51	0.38	-2.22	0.0212	E2F7	E2F transcription factor 7
**3841474**	8.02	9.17	1.02	0.7	-2.21	0.0317	LENG8	leukocyte receptor cluster (LRC) member 8
**3477917**	9.27	10.41	1.12	0.08	-2.21	0.0186	SLC15A4	solute carrier family 15 (oligopeptide transporter), member 4
**3851651**	7.19	8.33	0.81	0.08	-2.21	0.035	TNPO2; SNORD41; SNORD135	transportin 2; small nucleolar RNA, C/D box 41; small nucleolar RNA, C/D box 135
**3524999**	5.65	6.79	0.33	0.24	-2.21	0.0035	LIG4	ligase IV, DNA, ATP-dependent
**3254337**	5.56	6.7	0.43	0.57	-2.21	0.0325	TMEM254	transmembrane protein 254
**2831875**	7.51	8.65	0.75	0.54	-2.21	0.0379	SLC35A4	solute carrier family 35, member A4
**3735623**	5.72	6.86	0.38	0.37	-2.21	0.0252	MFSD11	major facilitator superfamily domain containing 11
**3049840**	6.62	7.76	0.64	0.18	-2.2	0.0434	HUS1; PKD1L1	HUS1 checkpoint clamp component; polycystic kidney disease 1 like 1
**3044938**	5.27	6.41	0.36	0.13	-2.2	0.0041	RP9P; KBTBD2	retinitis pigmentosa 9 pseudogene; kelch repeat and BTB (POZ) domain containing 2
**3917204**	5.98	7.12	0.41	0.23	-2.2	0.0033	MAP3K7CL	MAP3K7 C-terminal like
**3291601**	7.51	8.65	0.21	0.68	-2.2	0.0241	EGR2	early growth response 2
**3529082**	6.55	7.69	0.57	0.3	-2.2	0.0244	BCL2L2-PABPN1; PABPN1	BCL2L2-PABPN1 readthrough; poly(A) binding protein, nuclear 1
**3778252**	5.83	6.97	0.69	0.53	-2.2	0.0181	ANKRD12	ankyrin repeat domain 12
**3625674**	6.81	7.95	0.87	0.36	-2.2	0.0198	RFX7	regulatory factor X, 7
**3947258**	4.53	5.67	0.24	0.55	-2.2	0.0225	WBP2NL	WBP2 N-terminal like
**3178416**	6.13	7.27	0.55	0.23	-2.2	0.027	SPIN1	spindlin 1
**2974671**	5.05	6.19	0.35	0.7	-2.2	0.029	SLC18B1	solute carrier family 18, subfamily B, member 1
**3874751**	7.01	8.15	0.66	0.23	-2.2	0.0044	PRNP	prion protein
**2992197**	7.38	8.51	0.86	0.27	-2.2	0.0319	SP4	Sp4 transcription factor
**3864597**	6.45	7.59	0.74	0.11	-2.2	0.0326	SMG9	SMG9 nonsense mediated mRNA decay factor
**3657041**	6.3	7.43	0.3	0.3	-2.19	0.0048	ITGAX	integrin alpha X
**2903401**	9.83	10.97	0.86	0.4	-2.19	0.0226	HLA-DPB1; RPL32P1	major histocompatibility complex, class II, DP beta 1; ribosomal protein L32 pseudogene 1
**3161113**	6.8	7.93	0.63	0.31	-2.19	0.0115	PDCD1LG2	programmed cell death 1 ligand 2
**3551566**	7.26	8.39	0.59	0.18	-2.19	0.012	EVL	Enah/Vasp-like

**3253683**	6.72	7.85	0.66	0.18	-2.19	0.0236	ZMIZ1	zinc finger, MIZ-type containing 1
**3441941**	6.51	7.63	0.94	0.81	-2.19	0.0412	VAMP1	vesicle associated membrane protein 1
**3426828**	6.05	7.18	0.7	0.08	-2.19	0.0232	VEZT; MIR331	vezatin, adherens junctions transmembrane protein; microRNA 331
**3739679**	6.38	7.51	0.93	0.28	-2.19	0.0385	VPS53; RPS4XP17	vacuolar protein sorting 53 homolog (S. cerevisiae); ribosomal protein S4X pseudogene 17
**4009506**	6.63	7.76	0.86	0.23	-2.19	0.0441	PHF8	PHD finger protein 8
**3835494**	4.74	5.87	0.3	0.2	-2.19	0.0074	ZNF226	zinc finger protein 226
**3239380**	3.44	4.57	0.23	0.3	-2.18	0.0074	THNSL1	threonine synthase-like 1
**3771336**	6.53	7.65	0.59	0.31	-2.18	0.0494	EXOC7	exocyst complex component 7
**3747812**	6.07	7.19	0.55	0.01	-2.18	0.0366	PEMT	phosphatidylethanolamine N-methyltransferase
**3057520**	6.94	8.06	0.45	0.58	-2.18	0.0349	TMEM120A; STYXL1	transmembrane protein 120A; serine/threonine/tyrosine interacting-like 1
**3844486**	6.08	7.2	0.47	0.23	-2.18	0.0409	MIER2	mesoderm induction early response 1, family member 2
**3146012**	6.46	7.59	0.59	0.48	-2.18	0.0152	NIPAL2	NIPA-like domain containing 2
**3454576**	5.32	6.44	0.48	0.23	-2.18	0.0187	SLC11A2	solute carrier family 11 (proton-coupled divalent metal ion transporter), member 2
**3869396**	4.33	5.45	0.24	0.28	-2.18	0.0036	ZNF841; ZNF432	zinc finger protein 841; zinc finger protein 432
**3943101**	6.36	7.48	0.66	0.17	-2.18	0.0115	DEPDC5	DEP domain containing 5
**3292735**	4.74	5.86	0.36	0.58	-2.17	0.0464	SLC25A16	solute carrier family 25 (mitochondrial carrier), member 16
**3778372**	7.93	9.05	0.39	0.63	-2.17	0.0462	TWSG1	twisted gastrulation BMP signaling modulator 1
**3812426**	6.25	7.37	0.97	0.38	-2.17	0.0407	RTTN	rotatin
**3285926**	4.25	5.37	0.53	0.24	-2.17	0.0136	ZNF33B; ZNF37BP	zinc finger protein 33B; zinc finger protein 37B, pseudogene
**4019465**	5.51	6.63	0.52	0.36	-2.17	0.0151	NKRF	NFKB repressing factor
**2409970**	6.65	7.76	0.75	0.39	-2.17	0.0489	HECTD3	HECT domain containing E3 ubiquitin protein ligase 3
**3726772**	7.41	8.52	1.33	0.45	-2.17	0.0355	LUC7L3	LUC7-like 3 pre-mRNA splicing factor
**3248999**	6.87	7.99	0.8	0.47	-2.17	0.0375	REEP3	receptor accessory protein 3
**3368520**	5.87	6.98	0.89	0.18	-2.17	0.0464	CSTF3; RPL29P22	cleavage stimulation factor, 3 pre-RNA, subunit 3;

								ribosomal protein L29 pseudogene 22
**3929325**	4.99	6.1	0.55	0.26	-2.17	0.0254	SYNJ1	synaptojanin 1
**3420854**	6.84	7.96	0.2	0.61	-2.17	0.0112	DYRK2	dual specificity tyrosine-(Y)-phosphorylation regulated kinase 2
**3572782**	6.67	7.78	0.41	0.12	-2.16	0.0096	ANGEL1	angel homolog 1 (Drosophila)
**3913712**	7.1	8.22	0.72	0.44	-2.16	0.0418	YTHDF1	YTH N(6)-methyladenosine RNA binding protein 1
**3829638**	6.06	7.18	0.36	0.18	-2.16	0.0153	KIAA0355	KIAA0355
**3563687**	5.76	6.87	0.78	0.24	-2.16	0.0386	VCPKMT	valosin containing protein lysine (K) methyltransferase
**3822322**	6.28	7.39	0.58	0.22	-2.16	0.0267	MRI1; C19orf53	methylthioribose-1-phosphate isomerase 1; chromosome 19 open reading frame 53
**2978989**	6.51	7.62	0.77	0.08	-2.16	0.0408	LATS1; PPIL4	large tumor suppressor kinase 1; peptidylprolyl isomerase (cyclophilin)-like 4
**3903481**	6.78	7.88	0.56	0.13	-2.16	0.0144	PIGU	phosphatidylinositol glycan anchor biosynthesis class U
**3602526**	6.67	7.77	0.83	0.09	-2.16	0.0434	FBXO22	F-box protein 22
**2829542**	6.85	7.96	0.8	0.26	-2.15	0.0448	C5orf24; DDX46	chromosome 5 open reading frame 24; DEAD (Asp-Glu-Ala-Asp) box polypeptide 46
**3733938**	6.18	7.29	0.72	0.14	-2.15	0.031	COG1	component of oligomeric golgi complex 1
**3831475**	5.32	6.42	1.08	0.47	-2.15	0.0358	ZNF382	zinc finger protein 382
**4010768**	5.33	6.43	0.4	0.38	-2.15	0.0121	ZC4H2	zinc finger, C4H2 domain containing
**3850166**	6.55	7.65	0.41	0.26	-2.15	0.0219	S1PR2; DNMT1	sphingosine-1-phosphate receptor 2; DNA (cytosine-5-)-methyltransferase 1
**3434142**	6.66	7.77	0.52	0.21	-2.15	0.0454	PRKAB1	protein kinase, AMP-activated, beta 1 non-catalytic subunit
**3894047**	6.62	7.72	0.56	0.45	-2.15	0.0252	PCMTD2; LINC00266-1	protein-L-isoaspartate (D-aspartate) O-methyltransferase domain containing 2; long intergenic non-protein coding RNA 266-1
**2785035**	5.92	7.03	0.68	0.39	-2.15	0.02	MFSD8	major facilitator superfamily domain containing 8
**3763687**	7.23	8.34	0.77	0.53	-2.15	0.0391	COIL	coilin
**3835983**	6.35	7.45	0.49	0.29	-2.15	0.041	CLASRP	CLK4-associating serine/arginine rich protein

**3440998**	5.73	6.83	0.11	0.56	-2.14	0.0131	LOC100128816	ACAH3104
**2615600**	8.52	9.62	0.9	0.29	-2.14	0.0464	STT3B	STT3B, subunit of the oligosaccharyltransferase complex (catalytic)
**3878429**	4.91	6.01	0.45	0.32	-2.14	0.0114	POLR3F	polymerase (RNA) III (DNA directed) polypeptide F, 39 kDa
**3821727**	5.9	7	0.68	0.29	-2.14	0.0204	ZNF136	zinc finger protein 136
**3237088**	6.37	7.47	0.8	0.01	-2.14	0.0467	STAM	signal transducing adaptor molecule (SH3 domain and ITAM motif) 1
**3818648**	5.34	6.44	0.44	0.37	-2.14	0.012	ZNF557	zinc finger protein 557
**3835544**	5.28	6.37	0.51	0.31	-2.14	0.026	ZNF227	zinc finger protein 227
**3823842**	7.19	8.29	0.46	0.39	-2.14	0.0369	TMEM38A	transmembrane protein 38A
**3145240**	3.99	5.09	0.32	0.31	-2.14	0.0146	C8orf37	chromosome 8 open reading frame 37
**3724591**	4.03	5.13	0.4	0.17	-2.14	0.0115	EFCAB13	EF-hand calcium binding domain 13
**3201277**	7.07	8.16	0.98	0.31	-2.13	0.035	KLHL9	kelch-like family member 9
**2634091**	6.98	8.07	0.62	0.29	-2.13	0.0377	NFKBIZ; NXPE3	nuclear factor of kappa light polypeptide gene enhancer in B-cells inhibitor, zeta; neurexophilin and PC-esterase domain family, member 3
**2675925**	6.1	7.19	0.81	0.14	-2.13	0.0321	DUSP7	dual specificity phosphatase 7
**3762473**	6.11	7.19	0.48	0.56	-2.12	0.0311	TOB1	transducer of ERBB2, 1
**3317868**	6.99	8.07	0.6	0.03	-2.12	0.0178	PGAP2	post-GPI attachment to proteins 2
**3654175**	7.44	8.52	0.61	0.24	-2.12	0.03	IL4R	interleukin 4 receptor
**3840194**	5.23	6.31	0.48	0.35	-2.12	0.0179	ZNF880	zinc finger protein 880
**3864445**	6.94	8.02	0.78	0.06	-2.12	0.0456	IRGQ; XRCC1	immunity-related GTPase family, Q; X-ray repair complementing defective repair in Chinese hamster cells 1
**2401643**	5.56	6.64	0.27	0.42	-2.12	0.0282	FUCA1	fucosidase, alpha-L- 1, tissue
**3303255**	5.61	6.69	0.75	0.22	-2.12	0.0242	ERLIN1; CHUK	ER lipid raft associated 1; conserved helix-loop-helix ubiquitous kinase
**3458216**	6.2	7.28	0.58	0.22	-2.11	0.0471	ZBTB39	zinc finger and BTB domain containing 39
**2856634**	4.06	5.14	0.58	0.07	-2.11	0.0177	ARL15	ADP-ribosylation factor like GTPase 15
**2440476**	7.51	8.58	0.86	0.22	-2.11	0.0412	TSTD1; F11R	thiosulfate sulfurtransferase (rhodanese)-like domain containing 1; F11 receptor

**3326635**	7.94	9.02	0.73	0.35	-2.11	0.0067	CD44	CD44 molecule (Indian blood group)
**3719210**	5.72	6.79	0.51	0.33	-2.11	0.0356	DHRS11	dehydrogenase/reductase (SDR family) member 11
**3376512**	4.69	5.77	0.17	0.47	-2.1	0.0495	HRASLS2	HRAS-like suppressor 2
**2710474**	6.47	7.54	0.32	0.38	-2.1	0.0047	P3H2	prolyl 3-hydroxylase 2
**3944690**	6.73	7.8	0.73	0.3	-2.1	0.0396	CYTH4	cytohesin 4
**3740838**	6.45	7.52	0.72	0.25	-2.1	0.042	SMG6; SMG6-IT1	SMG6 nonsense mediated mRNA decay factor; SMG6 intronic transcript 1
**3146661**	5.77	6.84	0.67	0.44	-2.1	0.0359	ANKRD46; GAPDHP62	ankyrin repeat domain 46; glyceraldehyde 3 phosphate dehydrogenase pseudogene 62
**3274173**	6.96	8.03	0.85	0.11	-2.1	0.0223	PITRM1	pitrilysin metallopeptidase 1
**3592054**	4.78	5.85	0.43	0.33	-2.1	0.0299	TRIM69	tripartite motif containing 69
**3470037**	6.38	7.44	0.7	0.19	-2.09	0.0369	PRDM4	PR domain containing 4
**3456260**	6.56	7.62	0.81	0.16	-2.09	0.0485	ATF7	activating transcription factor 7
**3647827**	5.37	6.43	0.73	0.24	-2.09	0.0209	ATF7IP2	activating transcription factor 7 interacting protein 2
**3854000**	8	9.06	0.54	0.15	-2.09	0.0083	SLC35E1	solute carrier family 35, member E1
**2636483**	7.83	8.89	0.76	0.06	-2.09	0.0431	SIDT1	SID1 transmembrane family, member 1
**3742783**	6.79	7.85	0.77	0.21	-2.09	0.0321	NLRP1	NLR family, pyrin domain containing 1
**3720739**	7.55	8.61	0.64	0.13	-2.09	0.0421	MSL1; RAPGEFL1; CASC3	male-specific lethal 1 homolog (Drosophila); Rap guanine nucleotide exchange factor like 1; cancer susceptibility candidate 3
**3278977**	5.73	6.79	0.88	0.35	-2.08	0.0268	DCLRE1C	DNA cross-link repair 1C
**3276421**	5.45	6.51	0.58	0.14	-2.08	0.0197	KIN	Kin17 DNA and RNA binding protein
**3284188**	5.83	6.89	0.43	0.69	-2.08	0.0351	ITGB1	integrin beta 1
**3909843**	6.51	7.57	0.59	0.24	-2.08	0.0256	ZFP64	ZFP64 zinc finger protein
**3869650**	5.79	6.85	0.67	0.33	-2.08	0.0397	ZNF83	zinc finger protein 83
**3428671**	6.15	7.21	0.24	0.22	-2.08	0.0059	CHPT1	choline phosphotransferase 1
**3352904**	6.59	7.64	0.7	0.48	-2.08	0.0178	SC5D	sterol-C5-desaturase
**3557811**	7.19	8.25	0.34	0.19	-2.08	0.002	PSME2; MIR7703	proteasome activator subunit 2; microRNA 7703
**3744680**	6.55	7.6	0.37	0.08	-2.08	0.0051	PIK3R5	phosphoinositide-3-kinase, regulatory subunit 5
**3954331**	6.4	7.46	0.6	0.29	-2.08	0.0352	TOP3B	topoisomerase (DNA) III beta

**3869312**	4.59	5.65	0.1	0.38	-2.08	0.0056	ZNF649	zinc finger protein 649
**3454662**	6.07	7.12	0.34	0.45	-2.07	0.029	CSRNP2	cysteine-serine-rich nuclear protein 2
**3761737**	5.68	6.73	0.33	0.29	-2.07	0.0216	ZNF652	zinc finger protein 652
**3402506**	9.43	10.48	0.55	0.5	-2.07	0.0227	CD27; TAPBPL	CD27 molecule; TAP binding protein-like
**3486025**	6.79	7.84	0.36	0.44	-2.07	0.0164	UFM1	ubiquitin-fold modifier 1
**3393834**	4.87	5.92	0.47	0.09	-2.07	0.0289	IFT46	intraflagellar transport 46
**3284073**	5.84	6.89	0.5	0.14	-2.07	0.0108	EPC1	enhancer of polycomb homolog 1 (Drosophila)
**3261886**	5.57	6.62	0.76	0.28	-2.07	0.0432	WBP1L; CYP17A1-AS1	WW domain binding protein 1-like; CYP17A1 antisense RNA 1
**3731543**	5.41	6.45	0.15	0.22	-2.06	0.0026	RGS9	regulator of G-protein signaling 9
**3398076**	6.37	7.42	0.52	0.34	-2.06	0.0386	NFRKB	nuclear factor related to kappaB binding protein
**3860596**	4.73	5.77	0.1	0.32	-2.06	0.0107	ZNF461	zinc finger protein 461
**2676319**	7.27	8.31	0.99	0.22	-2.06	0.0491	GLT8D1	glycosyltransferase 8 domain containing 1
**3139950**	3.62	4.67	0.62	0.33	-2.06	0.0271	LACTB2	lactamase, beta 2
**3510925**	5.39	6.44	0.47	0.27	-2.06	0.0455	MRPS31	mitochondrial ribosomal protein S31
**3252071**	6.32	7.36	0.63	0.51	-2.06	0.0494	VCL	vinculin
**3439063**	4.69	5.73	0.39	0.47	-2.06	0.0249	ZNF26	zinc finger protein 26
**3314040**	7.45	8.49	1.08	0.31	-2.05	0.0211	BNIP3	BCL2/adenovirus E1B 19kDa interacting protein 3
**3380697**	8.07	9.11	0.79	0.49	-2.05	0.0383	DHCR7	7-dehydrocholesterol reductase
**2955061**	7.09	8.13	0.75	0.13	-2.05	0.0466	SLC35B2	solute carrier family 35 (adenosine 3-phospho 5-phosphosulfate transporter), member B2
**3339261**	6.24	7.28	0.3	0.38	-2.05	0.023	IL18BP	interleukin 18 binding protein
**3221916**	7.21	8.25	0.78	0.25	-2.05	0.0385	AKNA	AT-hook transcription factor
**3969713**	4.59	5.63	0.32	0.52	-2.05	0.0445	MOSPD2	motile sperm domain containing 2
**3725602**	7.5	8.54	0.59	0.3	-2.05	0.0223	ABI3	ABI family, member 3
**3183305**	5.07	6.1	0.55	0.17	-2.05	0.0206	FKTN; FSD1L	fukutin; fibronectin type III and SPRY domain containing 1-like
**3352813**	6.09	7.13	0.73	0.19	-2.05	0.0338	TBCEL	tubulin folding cofactor E-like
**3444436**	5.19	6.23	0.4	0.36	-2.04	0.0324	TAS2R14	taste receptor, type 2, member 14
**3310725**	5.15	6.18	0.65	0.08	-2.04	0.0231	C10orf88	chromosome 10 open reading frame 88

**3847906**	7.59	8.62	0.65	0.13	-2.04	0.037	DENND1C	DENN/MADD domain containing 1C
**3553228**	6.13	7.16	0.66	0.41	-2.04	0.0461	RCOR1	REST corepressor 1
**2351854**	6.14	7.17	0.49	0.36	-2.04	0.0232	C1orf162	chromosome 1 open reading frame 162
**3608466**	7.14	8.17	0.42	0.34	-2.04	0.0077	MAN2A2	mannosidase, alpha, class 2A, member 2
**3996430**	6.74	7.76	0.81	0.18	-2.04	0.0419	FAM50A	family with sequence similarity 50, member A
**3501661**	7.87	8.89	0.96	0.5	-2.04	0.033	ARHGEF7	Rho guanine nucleotide exchange factor 7
**3729052**	3.76	4.78	0.64	0.27	-2.04	0.0234	YPEL2	yippee like 2
**3371544**	6.27	7.29	0.52	0.16	-2.03	0.0392	AMBRA1	autophagy/beclin-1 regulator 1
**3870135**	3.29	4.32	0.5	0.47	-2.03	0.0352	ZNF347	zinc finger protein 347
**3429406**	5.39	6.41	0.62	0.48	-2.03	0.0424	HCFC2	host cell factor C2
**3422231**	7.58	8.6	0.31	0.72	-2.03	0.0327	TMEM19	transmembrane protein 19
**3962469**	7.64	8.66	0.56	0.12	-2.03	0.0456	RRP7BP	ribosomal RNA processing 7 homolog B, pseudogene
**3846926**	6.95	7.97	0.7	0.39	-2.03	0.046	DPP9	dipeptidyl-peptidase 9
**2487696**	5.15	6.17	0.34	0.18	-2.03	0.0267	PCYOX1	prenylcysteine oxidase 1
**3375091**	7.8	8.82	0.48	0.34	-2.03	0.0193	SLC15A3	solute carrier family 15 (oligopeptide transporter), member 3
**3456805**	4.54	5.56	0.52	0.27	-2.02	0.0181	GTSF1	gametocyte specific factor 1
**3846742**	7.33	8.34	0.56	0.24	-2.02	0.039	SH3GL1	SH3-domain GRB2-like 1
**3840944**	3.94	4.96	0.44	0.38	-2.02	0.0183	ZNF813; ZNF845; ZNF525; ZNF765	zinc finger protein 813; zinc finger protein 845; zinc finger protein 525; zinc finger protein 765
**3734797**	7.46	8.48	0.7	0.18	-2.02	0.0346	TMEM94; MIR6785	transmembrane protein 94; microRNA 6785
**3537264**	6.43	7.45	0.76	0.1	-2.02	0.0189	TMEM260	transmembrane protein 260
**3910360**	6.32	7.34	0.62	0.34	-2.02	0.0374	BCAS1	breast carcinoma amplified sequence 1
**3704376**	7.63	8.65	0.58	0.22	-2.02	0.0377	PIEZO1	piezo-type mechanosensitive ion channel component 1
**3204648**	6.07	7.08	0.39	0.28	-2.02	0.0101	CD72	CD72 molecule
**3259253**	9.46	10.47	1.08	0.13	-2.01	0.02	ENTPD1; C10orf131	ectonucleoside triphosphate diphosphohydrolase 1; chromosome 10 open reading frame 131
**3699080**	5.34	6.35	0.68	0.48	-2.01	0.0271	MLKL	mixed lineage kinase domain-like
**2596514**	6.01	7.02	0.27	0.06	-2.01	0.004	KLF7	Kruppel-like factor 7 (ubiquitous)
**3603436**	5.78	6.79	0.24	0.16	-2.01	0.0029	CHRNA5	cholinergic receptor, nicotinic alpha 5

**3167511**	6.25	7.25	0.56	0.07	-2.01	0.0216	GALT	galactose-1-phosphate uridylyltransferase
**3544562**	6.46	7.46	0.3	0.28	-2	0.0181	JDP2	Jun dimerization protein 2
**3623771**	7.18	8.18	1.12	0.14	-2	0.0266	TRPM7	transient receptor potential cation channel, subfamily M, member 7
**3976766**	6.68	7.69	0.63	0.1	-2	0.0315	WAS	Wiskott-Aldrich syndrome
**3869714**	4.73	5.73	0.66	0.32	-2	0.033	ZNF611; ZNF320	zinc finger protein 611; zinc finger protein 320
**2854327**	4.25	5.25	0.14	0.56	-2	0.0049	FYB	FYN binding protein
**3401217**	5.39	6.39	0.36	0.12	-2	0.0059	TULP3	tubby like protein 3
**3203311**	5.98	6.98	0.33	0.35	-2	0.0418	APTX	aprataxin
**3360350**	3.7	2.7	0.44	0.09	2	0.0306	OR52E2	olfactory receptor, family 52, subfamily E, member 2
**3867693**	6.81	5.8	0.46	0.18	2.01	0.0435	C19orf73	chromosome 19 open reading frame 73
**2947877**	5.31	4.3	0.21	0.07	2.01	0.0021	UBD; GABBR1	ubiquitin D; gamma-aminobutyric acid (GABA) B receptor, 1
**3252690**	5.43	4.43	0.22	0.08	2.01	0.0058	C10orf11	chromosome 10 open reading frame 11
**3199207**	5.17	4.16	0.65	0.44	2.01	0.0117	NFIB	nuclear factor I/B
**2781138**	5.94	4.93	0.26	0.1	2.01	0.0092	LEF1	lymphoid enhancer-binding factor 1
**3845581**	9.63	8.62	0.55	0.39	2.02	0.0465	ABHD17A	abhydrolase domain containing 17A
**3653266**	4.83	3.81	0.38	0.38	2.03	0.0142	CACNG3	calcium channel, voltage-dependent, gamma subunit 3
**3655621**	5.64	4.62	0.13	0.34	2.03	0.0135	ZG16	zymogen granule protein 16
**3822949**	4.39	3.37	0.48	0.87	2.04	0.0348	OR7C2	olfactory receptor, family 7, subfamily C, member 2
**3250438**	6.75	5.72	0.22	0.35	2.04	0.0259	C10orf35	chromosome 10 open reading frame 35
**2947703**	3.5	2.47	0.24	0.08	2.05	0.0023	OR2B3	olfactory receptor, family 2, subfamily B, member 3
**2855285**	3.34	2.29	0.12	0.27	2.07	0.0055	SEPP1	selenoprotein P, plasma, 1
**2776126**	4.53	3.48	0.44	0.21	2.08	0.0174	OK/SW-CL.36	OK/SW-CL.36
**3819870**	5.43	4.37	0.07	0.03	2.09	0.0009	OR1M1	olfactory receptor, family 1, subfamily M, member 1
**2718259**	5.28	4.22	0.32	0.31	2.09	0.0086	DRD5	dopamine receptor D5
**3373346**	3.8	2.74	0.22	0.36	2.1	0.0236	OR8K5	olfactory receptor, family 8, subfamily K, member 5
**3188200**	3.82	2.73	0.55	0.15	2.13	0.0195	OR1L1	olfactory receptor, family 1, subfamily L, member 1
**3602390**	5.64	4.55	0.37	0.31	2.13	0.0219	SNX33	sorting nexin 33

**3080437**	6.55	5.46	0.45	0.21	2.13	0.0282	LINC01287; ERVFC1-1	long intergenic non-protein coding RNA 1287; endogenous retrovirus group FC1, member 1
**2906720**	5.49	4.4	0.55	0.12	2.13	0.0144	TREML4; TREML5P	triggering receptor expressed on myeloid cells-like 4; triggering receptor expressed on myeloid cells-like 5, pseudogene
**3399398**	3.91	2.79	0.32	0.28	2.17	0.0159	MIR4697HG	MIR4697 host gene
**3028934**	4.25	3.13	0.73	0.11	2.17	0.0456	PIP	prolactin-induced protein
**2794584**	5.58	4.45	0.48	0.12	2.18	0.0226	GPM6A	glycoprotein M6A
**2466379**	4.08	2.94	0.36	0.24	2.2	0.0124	LOC100128185	PNAS-19
**2325877**	2.89	1.75	0.55	0.07	2.2	0.0088	RHD	Rh blood group, D antigen
**3360702**	4.7	3.56	0.43	0.13	2.2	0.0091	OR52L1	olfactory receptor, family 52, subfamily L, member 1
**4007376**	3	1.86	0.91	0.24	2.22	0.0387	SSX3	synovial sarcoma, X breakpoint 3
**2669979**	5.22	4.04	0.32	0.73	2.26	0.0269	CX3CR1	chemokine (C-X3-C motif) receptor 1
**3216736**	5.31	4.12	0.68	0.11	2.29	0.0146	LOC100130916	HSAL5836
**3017068**	5.18	3.99	0.65	0.67	2.29	0.0455	NFE4	nuclear factor, erythroid 4
**2578610**	5	3.79	0.56	0.25	2.32	0.0174	NXPH2	neurexophilin 2
**3416740**	4.78	3.57	0.23	0.33	2.32	0.0081	OR10A7; OR6C74; OR9R1P; OR9K1P	olfactory receptor, family 10, subfamily A, member 7; olfactory receptor, family 6, subfamily C, member 74; olfactory receptor, family 9, subfamily R, member 1 pseudogene; olfactory receptor, family 9, subfamily K, member 1 pseudogene
**3756709**	3.66	2.45	0.29	0.53	2.32	0.0367	KRTAP2-2; KRTAP2-4; KRTAP2-1	keratin associated protein 2-2; keratin associated protein 2-4; keratin associated protein 2-1
**3151086**	3.77	2.55	0.41	0.74	2.33	0.038	HAS2	hyaluronan synthase 2
**3362791**	8.33	7.1	0.38	0.39	2.35	0.0048		
**3595594**	4.31	3.07	0.1	0.07	2.36	0.0004	AQP9	aquaporin 9
**3889624**	5.77	4.53	0.14	0.18	2.37	0.0017	TSHZ2	teashirt zinc finger homeobox 2
**2359282**	9.36	8.11	0.34	0.2	2.39	0.0032	LCE5A	late cornified envelope 5A
**3976450**	4.44	3.16	0.74	0.57	2.42	0.0328	SPACA5B; SPACA5	sperm acrosome associated 5B; sperm acrosome associated 5
**2688070**	3.37	2.09	0.33	0.46	2.42	0.0184	GUCA1C	guanylate cyclase activator 1C

**2804085**	3.47	2.18	0.36	0.18	2.44	0.004	PMCHL1	pro-melanin-concentrating hormone-like 1, pseudogene
**3332131**	4.73	3.41	0.12	0.24	2.49	0.0005	STX3; OR10V2P; OR10Y1P	syntaxin 3; olfactory receptor, family 10, subfamily V, member 2 pseudogene; olfactory receptor, family 10, subfamily Y, member 1 pseudogene
**2668132**	5.29	3.97	0.14	0.28	2.5	0.0019	LOC105377021	putative uncharacterized protein UNQ6490/PRO21339
**3881236**	3.85	2.52	0.2	0.7	2.51	0.0449	DEFB118; DEFB117	defensin, beta 118; defensin, beta 117 (pseudogene)
**2974576**	4.56	3.22	0.88	0.33	2.54	0.0307	TAAR1	trace amine associated receptor 1
**2926147**	5.71	4.35	0.9	0.49	2.57	0.0263	TAAR6	trace amine associated receptor 6
**3915479**	2.82	1.43	0.45	0.82	2.62	0.0477	CXADR; BTG3	coxsackie virus and adenovirus receptor; BTG family, member 3
**3416852**	5.03	3.61	0.69	0.64	2.67	0.016	OR6C76	olfactory receptor, family 6, subfamily C, member 76
**3823379**	5.14	3.7	0.4	0.19	2.72	0.0017	OR10H2	olfactory receptor, family 10, subfamily H, member 2
**3410056**	6.66	5.21	0.66	0.21	2.74	0.0085	TSPAN11	tetraspanin 11
**3318517**	6.1	4.65	0.85	0.22	2.74	0.0465	OR52N2	olfactory receptor, family 52, subfamily N, member 2
**3612166**	8.76	7.28	1.07	0.13	2.79	0.0284	WASH3P; MIR6859-1; MIR6859-2; MIR6859-3; MIR6859-4; WASH5P; WASH1	WAS protein family homolog 3 pseudogene; microRNA 6859-1; microRNA 6859-2; microRNA 6859-3; microRNA 6859-4; WAS protein family homolog 5 pseudogene; WAS protein family homolog 1
**3416834**	4.54	2.97	0.31	0.09	2.98	0.0007	OR6C3	olfactory receptor, family 6, subfamily C, member 3
**3331047**	3.67	2.08	1.12	0.8	3.01	0.0389	OR9G1; OR9G9	olfactory receptor, family 9, subfamily G, member 1; olfactory receptor, family 9, subfamily G, member 9
**2671728**	7.65	6	0.31	0.17	3.13	0.0006	CDCP1	CUB domain containing protein 1
**2390253**	6.07	4.42	0.41	0.48	3.15	0.0046	OR2L8	olfactory receptor, family 2, subfamily L, member 8 (gene/pseudogene)
**3527348**	5.35	3.64	0.58	0.27	3.27	0.0011	OR4N5	olfactory receptor, family 4, subfamily N, member 5
**3333425**	3.84	2.11	0.46	0.37	3.32	0.0068	SCGB1D2	secretoglobin, family 1D, member 2

**2742009**	4.47	2.72	0.22	0.57	3.35	0.0019	ADAD1	adenosine deaminase domain containing 1
**3138204**	7.7	5.94	0.66	0.23	3.4	0.0146	CYP7B1	cytochrome P450, family 7, subfamily B, polypeptide 1
**3207241**	5.09	3.17	0.65	0.62	3.78	0.0033	LOC403323; LOC105379440	uncharacterized LOC403323; uncharacterized LOC105379440
**3206317**	3.51	1.57	1.11	0.12	3.82	0.0123	ZNF658B	zinc finger protein 658B, pseudogene
**3173479**	6.31	4.36	1.02	0.52	3.86	0.0222	FOXD4L3	forkhead box D4-like 3
**2482683**	5.34	3.28	0.23	0.93	4.16	0.0196	RPL23AP32; SPTBN1	ribosomal protein L23a pseudogene 32; spectrin, beta, non-erythrocytic 1
**3527290**	4.51	2.06	0.91	0.5	5.46	0.001	OR4N2	olfactory receptor, family 4, subfamily N, member 2
**2692883**	9.37	6.37	1.27	0.47	8.01	0.0156	MUC13	mucin 13, cell surface associated
**2359352**	5.75	2.5	1.11	1.39	9.51	0.0086	LCE2D	late cornified envelope 2D
**3143112**	7.33	3.62	2.68	0.52	13.07	0.0417	REXO1L1P; REXO1L3P	REX1, RNA exonuclease 1 homolog-like 1, pseudogene; REX1, RNA exonuclease 1 homolog-like 3, pseudogene

Supplementary Table S3. Differentially expressed genes detected in both skin fibroblast and lymphoblastoid cell lines*.

Fibroblasts Fold Change	Lymphoblasts Fold Change	Gene Symbol	Protein Product
**2.59**	**-4.39**	**AHR**	**aryl hydrocarbon receptor**
-3.96	*-3.95*	*AMPD3*	*adenosine monophosphate deaminase 3*
**2.41**	**-2.65**	**ANTXR2**	**anthrax toxin receptor 2**
**2.38**	**-2.24**	**ARGLU1**	**arginine and glutamate rich 1**
**2.47**	**-3.17**	**ATM**	**ATM serine/threonine kinase**
-3.55	*-6.75*	*BCAT1*	*branched chain amino-acid transaminase 1, cytosolic*
-3.48	*-2.05*	*BNIP3*	*BCL2/adenovirus E1B 19kDa interacting protein 3*
-5.09	*-4.2*	*CCR1*	*chemokine (C-C motif) receptor 1*
-25.61	*-3.37*	*CLIC6*	*chloride intracellular channel 6*
-2.8	*-2.82*	*CTSC*	*cathepsin C*
-5.1	*-2.31*	*EPSTI1*	*epithelial stromal interaction 1 (breast)*
2.05	*-2.52*	*ETNK1*	*ethanolamine kinase 1*
**2.36**	**-3.49**	**FAM63B**	**family with sequence similarity 63, member B**
**2.09**	**-2.31**	**FAM76B**	**family with sequence similarity 76, member B**
-2.56	*-2.88*	*FBXO21*	*F-box protein 21*
**2.47**	**-2.85**	**GCNT1**	**glucosaminyl (N-acetyl) transferase 1, core 2**
**2.03**	**-3.95**	**GPCPD1**	**glycerophosphocholine phosphodiesterase 1**
**-3.39**	**2.33**	**HAS2**	**hyaluronan synthase 2**
**2.06**	**-3.72**	**ICE2**	**interactor of little elongation complex ELL subunit 2**
**2.07**	**-3.25**	**MAML2**	**mastermind-like transcriptional coactivator 2**
**2.47**	**-4.45**	**MBNL3**	**muscleblind-like splicing regulator 3**
-2.02	*-2.01*	*MLKL*	*mixed lineage kinase domain-like*
2.91	-3.37	MYEF2	myelin expression factor 2
-2.29	*-2.1*	*P3H2*	*prolyl 3-hydroxylase 2*
-2.27	*-2.97*	*PAG1*	*phosphoprotein membrane anchor with glycosphingolipid m 1*
-9.69	*-2.29*	*PFKFB3*	*6-phosphofructo-2-kinase/fructose-2,6-biphosphatase 3*
-2.02	*-3.27*	*PFKP*	*phosphofructokinase, platelet*
**3.68**	**-4.14**	**PLAG1**	**pleiomorphic adenoma gene 1**
**2.02**	**-3.74**	**PPHLN1**	**periphilin 1**
**2**	**-3.07**	**QSER1**	**glutamine and serine rich 1**
-2.03	*-3.21*	*RAB11FIP1*	*RAB11 family interacting protein 1 (class I)*
**2.12**	**-2.88**	**RBBP6**	**retinoblastoma binding protein 6**
**2.32**	**-5.42**	**RHOBTB3**	**Rho-related BTB domain containing 3**
**2.12**	**-3.23**	**RRAGB**	**Ras-related GTP binding B**
-2.26	*-2.17*	*RTTN*	*rotatin*
**2.27**	**-3.92**	**SCAF11**	**SR-related CTD-associated factor 11**
**2.1**	**-3.23**	**SCAF4**	**SR-related CTD-associated factor 4**

**2.14**	**-2.63**	**SLF2**	**SMC5-SMC6 complex localization factor 2**
**3.66**	**-3.44**	**SMARCA2**	**SWI/SNF related, regulator of chromatin structure**
**2.21**	**-3.33**	**SON**	**SON DNA and RNA binding protein**
-2.02	*-2.67*	*STAU2*	*staufen double-stranded RNA binding protein 2*
**2.38**	**-2.39**	**TCF4**	**transcription factor 4**
-3.59	*-3.2*	*TMEM45A*	*transmembrane protein 45A*
**2.01**	**-4.68**	**TXNIP**	**thioredoxin interacting protein**
**2.25**	**-3.58**	**USP25**	**ubiquitin specific peptidase 25**
**2.11**	**-3.29**	**VPS36**	**vacuolar protein sorting 36 homolog (S. cerevisiae)**
**2.19**	**-2.72**	**ZFP90**	**ZFP90 zinc finger protein**
**2.37**	**-3.19**	**ZMAT1**	**zinc finger, matrin-type 1**

*Those genes that exhibited the same changes in gene expression in both cell types are indicated in italics and underlined, while those that had opposite gene expression changes are indicated in bold letters.

Supplementary Table S4. Wiki Pathways affected in SS fibroblasts

Pathway	#Total	Up List	*Down List*	Significance	p-value
VEGFA-VEGFR2 Signaling Pathway	20	**TXNIP,NCK1,PLAU, EPS15,TNXB,SSR3, ADAM10,S1PR1**	*VEGFA,SPHK1,NRP2,CXCL8,PTGS2,PRKCD,PLAUR, PGF,SOD2,IGFBP7,PDE4DIP,P4HA2*	4.13	0.000074
Nuclear Receptors Meta-Pathway	18	**NR3C1,SDPR,AHR, SLC6A15**	*SLC7A11,TSC22D3, CES1,SLC2A1,SRGN, SLC7A5,TNFAIP3,GCLM, PTGS2,NRG1,CCL20,ME1, SRXN1,AMIGO2*	4.93	0.000012
PodNet: protein-protein interactions in the podocyte	13	**MYOC,ADAM10, PLCE1,NCK1**	*KRT7,NOTCH3, KCNMA1,IGFBP7,PLAUR PTGS2,VEGFA,IGFBP2, COL18A1*	2.66	0.002203
PI3K-Akt Signaling Pathway	13	**TNXB,GNG12, COL6A6,IL7,LAMA4 ,PRLR**	*DDIT4,EIF4EBP1,IL6, ITGA8,TLR2,VEGFA,PGF*	2.07	0.008601
Focal Adhesion-PI3K-Akt-mTOR-signaling pathway	12	**PRLR,GNG12,LAMA4TNXB**	*COL5A1,DDIT4,PGF, SLC2A1,EIF4EBP1,ITGA8, VEGFA,PFKFB3*	2.23	0.005875
Adipogenesis	11	**NR3C1,RBL1,PRLR, AHR,LMNA**	*LIF,CYP26B1,MIF,IL6, TRIB3,RORA*	4.81	0.000015
Endothelin Pathway	10	**CASP8,GULP1**	*RGS3,CXCL8,PTGS2, EDNRA,COL5A1,CXCL1,VEGFA,IL6*	2.15	0.007033
Sudden Infant Death Syndrome (SIDS) Susceptibility Pathways	9	**NR3C1,THRB**	*POU2F2,IL6,RORA, RUNX3,VEGFA,TAC1, IL1RN*	2.76	0.001727
Circadian rythm related genes	9	**PRKG2,NR1D2,AHR ,NLGN1**	*IL6,RORA,KCNMA1, NAMPT,OPN3*	2.07	0.008474
Glucocorticoid Receptor Pathway	8	**NR3C1,SDPR**	*AMIGO2,TSC22D3,PTGS2 TNFAIP3,CCL20,SRGN*	4.56	0.000027
Spinal Cord Injury	8	**MBP**	*MIF,AQP1,IL6,CXCL8, CXCL1,CXCL2,PTGS2*	3.05	0.000891
NRF2 pathway	8	**SLC6A15**	*SLC7A11,SLC2A1,NRG1, ME1,SRXN1,CES1,GCLM*	2.5	0.003175
Myometrial Relaxation and Contraction Pathways	8	**ATP2A3,GNG12,PKIA**	*IL6,RGS7,IGFBP2,PRKCD, RGS3*	2.27	0.005362
Chemokine signaling pathway	8	**GNG12**	*PLCB4,CCL28,CCR1, PRKCD,CXCL14,CXCL3, CCL20*	2.11	0.00771
Non-genomic actions of 1,25 dihydroxyvitamin D3	7	**PLCE1**	*PLCB4,PRKCD,TLR2, MAPK13,RSAD2,CXCL8*	3.44	0.000367
Regulation of toll-like receptor signaling pathway	7	**CASP8,TLR3**	*CYLD,TLR2,MAPK13, TNFAIP3,IL6*	1.85	0.014031
Hepatitis B infection	7	**CASP8,TLR3**	*STAT4,CXCL8,TLR2,IL6,MAPK13*	1.82	0.015

Calcium Regulation in the Cardiac Cell	7	**GNG12,ATP2A3,PKIA**	*PRKCD,GJA3,RGS7,RGS3*	1.78	0.016543
EGF/EGFR Signaling Pathway	7	**PLCE1,STAM2,NCK1AP2B1,EPS15**	*EIF4EBP1,PRKCD*	1.67	0.021201
Senescence and Autophagy in Cancer	6	**PLAU**	*CXCL14,IGFBP7,IL24,IL6, CXCL1*	1.86	0.013943
Genes involved in male infertility	6	**AHR,ATM**	*SOD2,CYP26B1,EPSTI1, HORMAD1*	1.42	0.03798
Nuclear Receptors	5	**THRB,NR1D2,ROR1, NR3C1**	*RORA*	3.36	0.00044
Amino Acid metabolism	5	**PPM1L**	*GCLM,P4HA2,BCAT1,TDO2*	1.65	0.022273
Toll-like Receptor Signaling Pathway	5	**CASP8,TLR3**	*TLR2,IL6,MAPK13*	1.5	0.031447
Photodynamic therapy-induced NFE2L2 (NRF2) survival signaling	4		*CES1,GCLM,MAPK13,SRXN1*	3.25	0.000566
Cytokines and Inflammatory Response	4	**IL7**	*CXCL2,CXCL1,IL6*	2.8	0.001595
Prostaglandin Synthesis and Regulation	4		*PTGS1,PTGS2,PTGER3,EDNRA*	2.74	0.001807
Oligodendrocyte Specification and differentiation(including remyelination), leading to Myelin Components for CNS	4	**MBP**	*CXCL2,CXCL1,LIF*	2.74	0.001807
Photodynamic therapy-induced NF-kB survival signaling	4		*IL6,VEGFA,CXCL2,PTGS2*	2.55	0.00285
Photodynamic therapy-induced HIF-1 survival signaling	4		*VEGFA,SLC2A1,PTGS2,IGFBP2*	2.46	0.003501
Splicing factor NOVA regulated synaptic proteins	4	**EPB41L2**	*KCNMA1,PLCB4,EPB41L3*	2.26	0.005551
Tryptophan metabolism	4	**ALDH3A2**	*TDO2,CYP19A1,ALDH2*	2.11	0.007675
Complement and Coagulation Cascades	4	**PLAU,PROS1**	*F10,PLAUR*	1.72	0.019067
RIG-I-like Receptor Signaling	4	**CASP8**	*CYLD,MAPK13,CXCL8*	1.72	0.019067
Dengue-2 Interactions with Complement and Coagulation Cascades	4	**PROS1,PLAU**	*F10,PLAUR*	1.7	0.020139
Lung fibrosis	4	**PLAU**	*CXCL2,IL6,CXCL8*	1.63	0.023575
Head and Neck Squamous Cell Carcinoma	4	**CASP8**	*VEGFA,DDIT4,EIF4EBP1*	1.41	0.03907
Nucleotide-binding domain, leucine rich repeat containing	4	**TXNIP**	*TNFAIP3,CYLD, SUGT1*	1.34	0.045855

receptor (NLR) signaling pathways					
miR-509-3p alteration of YAP1/ECM axis	3	**GPC6**	*EDNRA,COL5A1*	2.56	0.002785
Sphingolipid Metabolism (general overview)	3	**CERS6,UGCG**	*SPHK1*	2.17	0.006727
Sphingolipid Metabolism (integrated pathway)	3	**CERS6,UGCG**	*SPHK1*	2.12	0.007592
Hypothesized Pathways in Pathogenesis of Cardiovascular Disease	3	**FBN1**	*POSTN,LTBP2*	2.07	0.00852
Eicosanoid Synthesis	3		*PTGS1,PTGES,PTGS2*	2.07	0.00852
Regulatory circuits of the STAT3 signaling pathway	3		*IL27RA,MAPK13,IL21R*	1.89	0.012876
Eicosanoid metabolism via Cyclo Oxygenases (COX)	3		*PTGS1,PTGS2,PTGES*	1.77	0.016837
miRNA regulation of p53 pathway in prostate cancer	3	**ATM,CASP8,ZMAT3**		1.77	0.016837
Development and heterogeneity of the ILC family	3	**AHR**	*RORA,IL6*	1.77	0.016837
Ovarian Infertility Genes	3	**PRLR,ATM**	*CYP19A1*	1.77	0.016837
Resistin as a regulator of inflammation	3	**PLCE1**	*PLCB4,IL6*	1.74	0.018291
Genes controlling renal nephrogenesis	3	**NCK1**	*VEGFA,ITGA8*	1.58	0.026582
Fibrin Complement Receptor 3 Signaling Pathway	3	**TLR3**	*IL6,CXCL3*	1.58	0.026582
Ferroptosis	3		*SLC7A11,GCLM,SAT1*	1.55	0.028444
miRNA targets in ECM and membrane receptors	3	**LAMA4,TNXB**	*COL5A1*	1.36	0.043344
Exercise-induced Circadian Regulation	3	**NR1D2**	*G0S2,BTG1*	1.32	0.04819
Differentiation Pathway	3	**GDF5**	*IL6,VEGFA*	1.32	0.04819
Thymic Stromal LymphoPoietin (TSLP) Signaling Pathway	3		*STAT4,EIF4EBP1,IL6*	1.32	0.04819
Gastric ulcer formation	2		*PTGS1,PTGS2*	2.55	0.002792
mRNA, protein, and metabolite inducation pathway by cyclosporin A	2		*SLC7A11,SLC7A5*	2.24	0.005732
COVID-19 AOP	2	**IL7**	*IL6*	1.58	0.026203
Transcriptional activation by NRF2	2		*GCLM,SLC7A11*	1.58	0.026203

Fatty Acid Omega Oxidation	2	**ADH1B**	*ALDH2*	1.58	0.026203
Deregulation of Rab and Rab Effector Genes in Bladder Cancer	2	**MYRIP,RAB27B**		1.53	0.029614
Amplification and Expansion of Oncogenic Pathways as Metastatic Traits	2	**TCF7L2**	*VEGFA*	1.48	0.033191
LTF danger signal response pathway	2		*IL6,TLR2*	1.39	0.040817
Hypertrophy Model	2	**ADAM10**	*EIF4EBP1*	1.35	0.044852
Sphingolipid Metabolism	2	**B4GALT6**	*SPHK1*	1.31	0.049028

Supplementary Table S5. Wiki Pathways affected in SS lymphoblasts

Pathway	#Total	**Up List**	*Down List*	Significance	p-value
EGF/EGFR Signaling Pathway	23		*MAPK1,MAPK8,EPS15L1,RASA1,VAV2, SOS2,MAPK3,JAK1,CDC42,ATXN2, RPS6KB1,ASAP1,SYNJ1,MAP4K1,USP6NL,ABI1,ROCK1,USP8,PTEN,STAM, STAT5B,CBL,PTK2*	2.37	0.004236
TGF-beta Signaling Pathway	22	**SPTBN1**	*MAPK1,MAPK8,WWP1,NUP214,TGFBR1,RBL2,MAPK3,CDC42,SMAD2, STAMBPL1,BTRC,SMURF2,E2F4,ITGB1 MAP4K1,APP,MAP2K6,PIAS1,ROCK1,ZEB1,PTK2*	3.07	0.000854
JAK/STAT	19		*PTPN6,MAPK3,FLNA,STAT5B,VAV2, CBL,CHUK,RPS6KB1,PTK2,MAPK1,REL BAX,ITGB1,IGF1,JAK1,CDC42,ROCK1,CFL2,MAPK8*	2.84	0.001456
Olfactory receptor activity	19	**OR6C76, OR6C74, OR9G1,OR52L1,OR2L8,OR7C2,OR2B3, OR10H2, OR52N2, OR6C3,OR4N2,OR8K5,OR52E2,OR4N5,OR10A7, OR1M1,OR1L1**	*OR52H1,OR4B1*	1.38	0.041643
Mesodermal Commitment Pathway	19	**LEF1**	*PIAS1,NLK,WDFY2,BMPR2,CCDC6, ELP4,C9orf72,TRIM5,FGFR1,LATS1, KDM6A,MBTD1,TCF4,BMPR1A, TRERF1,TWSG1,SMAD2,AEBP2*	1.34	0.045833
Genes involved in male infertility	18	**UBD**	*ABLIM1,AHR,ARNTL,ATM,CLOCK, EPSTI1,FAS,LIG4,MLH3,PEMT,RGS9, SHMT1,TEX15,USP8,CCNT1,CCNK, CDK9*	1.39	0.040353
Endoderm Differentiation	18	**LEF1**	*RTF1,RFX7,BPTF,MBTD1,SP4,APP, AEBP2,BMPR1A,SMAD2,PIAS1,NLK, WDFY2,ELP4,TRIM5,TCF4,EMSY, TRERF1*	1.39	0.040353
miR-targeted genes in epithelium - TarBase	17	**CDCP1**	*GNA13,ADPGK,CTSC,DMTF1, TP53INP1,GRPEL2,CPNE8,NUFIP2, VPS39,FADS1,GEMIN7,PPP1R7,GSTM4 SDCBP,MIR30B,MIR30D*	1.37	0.042799
B Cell Receptor Signaling Pathway	16		*CHUK,REL,MAP2K6,VAV2,MALT1, PTPN6,MAPK3,RAPGEF1,MAX,CDC42, CBL,MAPK8,BLK,MAPK1,CD22, MAP4K1*	2.38	0.004197

T-Cell antigen Receptor (TCR) Signaling Pathway	15		*MAP4K1,MAPK8,LCP2,WAS,REL,CHUK,FYB,CDC42,GRAP2,MAPK3,MAPK1,MALT1,CBL,SKAP1,FAS*	2.31	0.004854
Integrin-mediated Cell Adhesion	15	**SEPP1**	*CDC42,ARHGEF7,VAV2,ROCK1,GIT2,PTK2,CAPN3,PAK6,MAP2K6,RAPGEF1,MAPK1,ITGAX,ITGB1,VCL*	1.89	0.012959
Leptin signaling pathway	14		*CHUK,MAPK8,STAT5B,PTEN,BAX,PTK2,CFL2,MAPK1,MAPK3,REL,RPS6KB1,JAK1,ROCK1,CDC42*	2.64	0.002271
IL-18 signaling pathway	14		*BAX,IL18R1,MAPK1,IL18BP,FAS,PTEN,REL,NFKBIZ,MAPK3,MAPK8,CHUK,KLC1,IRAK4,RPS6KB1*	1.27	0.054269
Nuclear Receptors Meta-Pathway	13		*ARL5B,NCOA6,SERTAD2,DNAJC15,FTH1,ALOX5AP,GSTP1,AHR,NRIP1,GSTM1,GSTM2,GSTM4,SLC39A8*	1.96	0.010912
Pancreatic adenocarcinoma pathway	13		*ARHGEF6,PAK6,CHUK,MAPK1,MAPK3,CDC42,MAPK8,RPS6KB1,JAK1,TGFBR1,SMAD2,BAX,DDB2*	1.6	0.024853
RNA pol II transcription (Initiation and elongation)	12		*CDK9,SUPT5H,RTF1,GTF2H1,TAF2,TAF9,MED1,MED12,MED13,MED15,MED20,MED21*	1.36	0.04363
IL-4 Signaling Pathway	11		*MAPK3,STAT6,RPS6KB1,CBL,PTPN6,JAK1,STAT5B,IL4R,MAPK1,RASA1,CHUK*	2.44	0.00364
Prolactin Signaling Pathway	11		*JAK1,PTPN6,STAT5B,MAPK3,CBL,MAPK1,ITGB1,VAV2,FLNA,RPS6KB1,PTK2*	1.32	0.048291
Processing of Capped Intron-Containing Pre-mRNA	9		*HNRNPUL1,CWC25,CASC3,PABPN1,NCBP1,AQR,SRSF11,RNPS1,SF1*	3.65	0.000226
Signaling of Hepatocyte Growth Factor Receptor	9		*MAP4K1,MAPK1,MAPK8,PTEN,PTK2,RAPGEF1,RASA1,MAPK3,ITGB1*	3.09	0.000817
IL-3 Signaling Pathway	9		*MAPK1,HCK,MAPK8,STAT5B,PTPN6,CBL,MAPK3,JAK1,RAPGEF1*	1.95	0.011338
RANKL/RANK (Receptor activator of NFKB (ligand)) Signaling Pathway	9		*MAP2K6,TRAF5,PTK2,MAPK8,MAPK3,MAPK1,CHUK,CBL,CDC42*	1.45	0.035541
IL-1 signaling pathway	9		*REL,MAPK3,CHUK,MAPK1,TAB3,MAP2K6,MAPK8,IRAK4,IRAK3*	1.45	0.035541
Interferon type I signaling pathways	9		*CBL,RAPGEF1,RPS6KB1,PTPN6,JAK1,PIAS1,IRF9,REL,MAP2K6*	1.38	0.041436
MET in type 1 papillary renal cell carcinoma	9		*MAPK8,CBL,SOS2,RAPGEF1,CDC42,PAK6,MAPK1,MAPK3,PTK2*	1.36	0.043956
Thymic Stromal LymphoPoietin (TSLP) Signaling	8		*MAPK3,MAPK1,JAK1,MAPK8,STAT6,STAT5B,TNFSF4,HCK*	1.54	0.028517
Cilium Assembly	7		*IFT46,CEP83,TRIP11,MARK4,IFT43,*	2.38	0.004163

			IFT74,CCP110		
Assembly of the primary cilium	7		*TRIP11,IFT46,IFT74,IFT43,CEP83, CCP110,MARK4*	2.38	0.004163
Autophagy	7		*ATG14,PIK3C3,MAP1LC3B,AMBRA1, UVRAG,RB1CC1,PRKAB1*	2.18	0.00658
Interleukin-1 family signaling	7		*IL18R1,IRAK3,MAP2K6,MAPK8,IRAK4, IL18BP,TBK1*	2.13	0.00737
G13 Signaling Pathway	7		*CFL2,GNA13,ARHGDIB,WAS,ROCK1, CDC42,RPS6KB1*	1.62	0.023906
Intra-Golgi and retrograde Golgi-to-ER traffic	6		*STX5,VAMP4,GOSR1,BET1L,USP6NL STX16*	2.12	0.00752
Pathogenesis of Cardiovascular Disease	6		*MAPK3,MAPK8,MAPK1,TGFBR1, SMAD2,FLNA*	2	0.010105
Metapathway biotransformation Phase I and II	6	**CYP7B1**	*GSTM4,GSTM2,GSTM1,GSTP1,CHST6*	1.74	0.018182
Type I Interferon Induction and Signaling During SARS-CoV-2 Infection	6	**TREML4**	*CHUK,TBK1,JAK1,MAPK8,IRF9*	1.68	0.020857
Prion disease pathway	6		*FGFR1,BCL11A,MAPK3,PRNP,MAPK1, PTK2*	1.32	0.048299
Class A/1 (Rhodopsin-like receptors)	5	**CX3CR1**	*LPAR6,GPR183,P2RY11,CMKLR1*	11.07	0
Deubiquitination	5		*ATXN3,STAMBPL1,STAM,PTEN, ZRANB1*	8.82	0
Generic Transcription Pathway	5		*MED1,MED15,MED13,MED20,MED12*	10.59	0
Chromatin organization	5		*PRMT3,BRWD1,PHF8,HDAC8,CLOCK*	5.85	0.000001
TCF dependent signaling in response to WNT	5		*CSNK1G2,USP8,BTRC,SMURF2,CSNK1 E*	5.56	0.000003
Cell surface interactions at the vascular wall	5	**CXADR**	*CD47,CD84,PTPN6,F11R*	4.96	0.000011
TCR signaling	5		*WAS,PTEN,GRAP2,LCP2,FYB*	2.25	0.005621
Hematopoietic Stem Cell Gene Regulation by GABP alpha/beta Complex	5		*GABPA,ETV6,DNMT1,PTEN,ATM*	1.81	0.015535
Hippo-Yap signaling pathway	5		*STK38L,LATS1,MINK1,NDRG1,MAP4K 1*	1.56	0.027858
The effect of progerin on the involved genes in	5	**LEF1**	*CHD3,RBBP4,MTA3,CBX5*	1.35	0.045062

Hutchinson-Gilford Progeria Syndrome					
ESR-mediated signaling	4		*MED1,POU2F1,NRIP1,KPNA2*	3.13	0.00075
Host Interactions of HIV factors	4		*BTRC,CCNT1,CDK9,HCK*	2.68	0.002103
EPH-Ephrin signaling	4		*ARHGEF7,PTK2,SDCBP,RASA1*	1.86	0.013929
Kennedy pathway from Sphingolipids	4		*PEMT,CHKA,ETNK1,CHPT1*	1.73	0.018583
Interactome of polycomb repressive complex 2 (PRC2)	4		*MORC3,AEBP2,EED,RBBP4*	1.52	0.029889
22q11.2 copy number variation syndrome	4		*MED15,CBX5,FGFR1,CDC42*	1.46	0.034636
IL-9 Signaling Pathway	4		*STAT5B,MAPK3,MAPK1,JAK1*	1.43	0.036773
MFAP5 effect on permeability and motility of endothelial cells via cytoskeleton rearrangement	4		*PTK2,MAPK1,VCL,MAPK3*	1.35	0.044492
Inhibition of exosome biogenesis and secretion by Manumycin A in CRPC cells	4		*RAB27A,MAPK1,MAPK3,RAB5B*	1.35	0.044492
Immunoregulatory interactions between a Lymphoid and a non-Lymphoid cell	3	**CXADR**	*SELL,CD226*	6.95	0
Mitotic G1 phase and G1/S transition	3		*DYRK1A,RBL2,CABLES1*	4.72	0.000019
Signaling by the B Cell Receptor (BCR)	3		*CD22,MALT1,PTPN6*	4.41	0.000039
Major pathway of rRNA processing in the nucleolus and cytosol	3		*RPS27L,UTP18,CSNK1E*	3.65	0.000222
Interferon gamma signaling	3		*PIAS1,PTPN6,CIITA*	3.34	0.000459
RHO GTPases Activate Formins	3		*EVL,FMNL3,ITGB1*	3.14	0.000727
Sudden Infant Death Syndrome (SIDS) Susceptibility Pathways	3	**SPTBN1**	*CREM,VAMP2*	2.6	0.002512
Human Complement System	3		*WAS,CR1,SELL*	2.17	0.006836
L1CAM interactions	3		*VAV2,NRCAM,SDCBP*	2.02	0.009531

Apoptotic execution phase	3	*ROCK1,PTK2,ACIN1*	2.02	0.009531
Regulation of mRNA stability by proteins that bind AU-rich elements	3	*ZFP36,NUP214,ZFP36L1*	1.88	0.013262
Regulation of TP53 Activity through Phosphorylation	3	*DYRK2,HIPK1,TP53INP1*	1.59	0.025497
Signaling by EGFR	3	*CBL,EPS15L1,PAG1*	1.31	0.048452
Class I MHC mediated antigen processing & presentation	2	*SNAP23,SEC23A*	13.13	0
Interleukin-4 and Interleukin-13 signaling	2	*JAK1,IL4R*	5.39	0.000004
Transcriptional regulation by RUNX1	2	*BLK,SOCS4*	5.2	0.000006
Integrin cell surface interactions	2	*CD44,F11R*	4.75	0.000018
Ion channel transport	2	*SLC9B2,CLIC2*	4.58	0.000026
Signaling by FGFR2	2	*FRS2,CBL*	4.1	0.000079
PIP3 activates AKT signaling	2	*CHUK,PTEN*	3.94	0.000114
Regulation of lipid metabolism by PPARalpha	2	*FADS1,GLIPR1*	3.79	0.000164
RNA Polymerase II Transcription	2	*EAF2,CDK9*	3.78	0.000167
Degradation of the extracellular matrix	2	*CD44,NID1*	3.63	0.000236
Signaling by VEGF	2	*PTK2,RASA1*	3.62	0.000238
Interferon alpha/beta signaling	2	*PTPN6,IRF9*	3.31	0.000495
Post-translational modification: synthesis of GPI-anchored proteins	2	*PIGB,PIGN*	3.29	0.000514
MAPK6/MAPK4 signaling	2	*IGF2BP1,MAPKAPK5*	2.99	0.001012
Oxidative Stress Induced Senescence	2	*RBBP4,EED*	2.97	0.001069
Integration of energy metabolism	2	*ARL2BP,VAMP2*	2.84	0.001442
Glycerophospholipid biosynthesis	2	*PEMT,GPCPD1*	2.68	0.002082
p75 NTR receptor-mediated signalling	2	*PRDM4,MAPK8*	2.68	0.002113

Signaling by FGFR1	2	*CBL,FRS2*	2.68	0.002113
Signaling by FGFR3	2	*CBL,FRS2*	2.38	0.004186
Hypoxia-mediated EMT and Stemness	2	*DICER1,ZEB1*	2.23	0.005894
Signaling by NTRK1 (TRKA)	2	*RAPGEF1,FRS2*	2.22	0.006004
DNA Double Strand Break Response	2	*KPNA2,MRE11A*	2.07	0.008546
Semaphorin interactions	2	*PLXNC1,CD72*	1.92	0.011948
SUMOylation of transcription cofactors	2	*PIAS1,NRIP1*	1.92	0.012143
MicroRNAs in cardiomyocyte hypertrophy	2	*LRP5,ROCK1*	1.77	0.016864
E3 ubiquitin ligases ubiquitinate target proteins	2	*WAC,RNF144A*	1.76	0.017511
Amyloid fiber formation	2	*SORL1,SIAH1*	1.62	0.023758
Signaling by PTK6	2	*RASA1,CBL*	1.62	0.023848
SUMOylation of intracellular receptors	2	*NR2C1,PIAS1*	1.48	0.033367
TP53 Regulates Transcription of DNA Repair Genes	2	*DDB2,CCNK*	1.47	0.03358
Transcriptional regulation by RUNX2	2	*RBM14,BAX*	1.46	0.035036
Signaling by FGFR4	2	*FRS2,CBL*	1.33	0.046707
Gamma carboxylation, hypusine formation and arylsulfatase activation	2	*DNAJC24,DPH6*	1.33	0.046835
O-linked glycosylation	1	*POMK*	14.96	0
Fc epsilon receptor (FCERI) signaling	1	*MALT1*	8.39	0
Mitotic Metaphase and Anaphase	1	*HDAC8*	7.33	0
RAF/MAP kinase cascade	1	*PEA15*	9.42	0
Mitotic Prometaphase	1	*HDAC8*	6.61	0
Signaling by ROBO receptors	1	*MYO9B*	8.21	0
Cell Cycle Checkpoints	1	*BUB1B*	9.77	0

C-type lectin receptors (CLRs)	1	*MALT1*	5.99	0.000001
Complement cascade	1	*CR1*	5.99	0.000001
Beta-catenin independent WNT signaling	1	*NLK*	4.98	0.00001
PTEN Regulation	1	*PTEN*	4.82	0.000015
G alpha (q) signalling events	1	*GRK5*	4.81	0.000016
Nucleotide Excision Repair	1	*ERCC6*	4.32	0.000048
Cardiac conduction	1	*CLIC2*	3.99	0.000103
Activation of anterior HOX genes in hindbrain development during early embryogenesis	1	*EGR2*	3.66	0.000221
Synthesis of DNA	1	*MCM8*	3.66	0.000221
Respiratory electron transport, ATP synthesis by chemiosmotic coupling, and heat production by uncoupling proteins.	1	*NDUFA12*	3.63	0.000236
Transcriptional Regulation by MECP2	1	*PTEN*	3.49	0.000326
Asparagine N-linked glycosylation	1	*MGAT5*	3.49	0.000326
Extracellular matrix organization	1	*SPARC*	3.32	0.000484
Hedgehog 'off' state	1	*TULP3*	3.29	0.00051
SUMOylation of DNA damage response and repair proteins	1	*BLM*	3.16	0.000699
S Phase	1	*CABLES1*	3.14	0.000727
Clathrin-mediated endocytosis	1	*PIK3C2A*	2.99	0.001024
Nonsense-Mediated Decay (NMD)	1	*SMG6*	2.98	0.001041
Selenoamino acid metabolism	1	*SECISBP2*	2.82	0.001498
ABC-family proteins mediated transport	1	*ABCA5*	2.81	0.001563
TP53 Regulates Metabolic Genes	1	*PTEN*	2.67	0.002159
Sphingolipid metabolism	1	*CSNK1G2*	2.66	0.002167

Gene Silencing by RNA	1		*TNRC6A*	2.66	0.002191
Mitochondrial biogenesis	1		*GABPA*	2.66	0.002191
Transcriptional regulation of white adipocyte differentiation	1		*EGR2*	2.66	0.002191
Extra-nuclear estrogen signaling	1		*PTK2*	2.65	0.002232
GPCRs, Other	1		*P2RY11*	2.65	0.002232
Costimulation by the CD28 family	1		*GRAP2*	2.64	0.00229
Regulation of mitotic cell cycle	1		*BUB1B*	2.47	0.003352
Keratinization	1		*KAZN*	2.47	0.003352
Regulation of RUNX2 expression and activity	1		*WWP1*	2.32	0.004772
NR1H2 and NR1H3-mediated signaling	1		*NRIP1*	2.31	0.004902
DNA Replication Pre-Initiation	1		*MCM8*	2.31	0.004902
M/G1 Transition	1		*MCM8*	2.31	0.004902
RNA polymerase II transcribes snRNA genes	1		*ZNF143*	2.18	0.006664
Metabolism of water-soluble vitamins and cofactors	1		*SLC25A16*	2.17	0.006823
Mitochondrial protein import	1		*PITRM1*	2.16	0.006969
Mitochondrial translation	1		*MRRF*	2.14	0.007163
Cell Differentiation - Index expanded	1		*MIR2861*	2.02	0.009561
RNA Polymerase I Transcription	1		*TAF1D*	2.01	0.0097
Platelet homeostasis	1		*PTPN6*	2.01	0.00979
Macroautophagy	1		*AMBRA1*	2	0.009945
Transcriptional regulation of granulopoiesis	1	**LEF1**		1.99	0.010167
tRNA processing in the mitochondrion	1		*TRNT1*	1.85	0.014033
Transcriptional regulation by the AP-2 (TFAP2) family of transcription factors	1		*KDM5B*	1.85	0.01424

Metabolism of polyamines	1	*MRI1*	1.85	0.01424
TNF signaling	1	*NSMAF*	1.68	0.02106
Prader-Willi and Angelman Syndrome	1	*FEZ1*	1.67	0.021558
NoRC negatively regulates rRNA expression	1	*DNMT1*	1.67	0.021558
Iron uptake and transport	1	*SLC11A2*	1.67	0.021558
Cell Differentiation - Index	1	*MIR2861*	1.65	0.022563
Transcriptional activity of SMAD2/SMAD3:SMAD4 heterotrimer	1	*SMURF2*	1.5	0.031322
Parkinsons Disease Pathway	1	*ATXN2*	1.37	0.042897
Nonhomologous End-Joining (NHEJ)	1	*DCLRE1C*	1.36	0.043502

Supplementary Table S6. IPA canonical pathways affected in SS fibroblasts

Ingenuity Canonical Pathways	-log(p-value)	Molecules
Role of IL-17A in Psoriasis	9.10E+00	CCL20,CXCL1,CXCL3,CXCL5,CXCL6,CXCL8,S100A7,S100A9
Osteoarthritis Pathway	8.14E+00	CASP8,CCN4,CTNNB1,CXCL8,DDIT4,DDR2,GDF5,LEP,MMP1,MMP3, NAMPT,PGF,PPARGC1A,PTCH1,PTGS2,S100A9,SMAD3,SOX9,SP1, SPHK1,TCF4,TCF7L2,TLR2,TLR4,VEGFA
Role of Macrophages, Fibroblasts and Endothelial Cells in Rheumatoid Arthritis	7.50E+00	CAMK2D,CTNNB1,CXCL8,GSK3B,ICAM1,IL1RN,IL32,IL33,IL36B,IL6,IL7, MIF,MMP1,MMP3,NFKBIA,PDGFD,PGF,PIK3R1,PLCB4,PLCE1,PPP3CC, PRKCD,SFRP2,TCF4,TCF7L2,TLR2,TLR3,TLR4,VEGFA,WNT5A
Hepatic Fibrosis / Hepatic Stellate Cell Activation	7.23E+00	AGTR1,COL15A1,COL18A1,COL23A1,COL27A1,COL5A1,COL6A6,CXCL3,CXCL8,EDNRA,FGF1,ICAM1,IGFBP5,IL6,LEP,MET,MMP1,PDGFD,PGF, SMAD3,TLR4,VEGFA
Glucocorticoid Receptor Signaling	6.79E+00	ADRB2,CDKN1C,CXCL3,CXCL8,HSPA1A/HSPA1B,ICAM1,IL1RN,IL6,KRT14,KRT15,KRT19,KRT34,KRT6A,KRT6B,KRT7,MAPK13,MMP1,NCOA2, NCOA3,NFKBIA,NR3C1,PIK3R1,PLAU,POU2F2,PPP3CC,PTGS2,SMAD3, SMARCA2,TAF9B,TSC22D3
Role of IL-17A in Arthritis	6.25E+00	CCL20,CXCL1,CXCL3,CXCL5,CXCL6,CXCL8,MAPK13,MMP1,NFKBIA, PIK3R1,PTGS2
Dendritic Cell Maturation	5.47E+00	COL18A1,DDR2,ICAM1,IL1RN,IL32,IL33,IL36B,IL6,LEP,LY75,MAPK13, NFKBIA,PIK3R1,PLCB4,PLCE1,STAT4,TLR2,TLR3,TLR4
Hepatic Fibrosis Signaling Pathway	5.02E+00	AGTR1,COL18A1,CTNNB1,CXCL8,EDNRA,GSK3B,ICAM1,IL1RN,IL33, IL36B,LEP,MAPK13,MMP1,NFKBIA,PDGFD,PDK1,PGF,PIK3R1,PRKCD, PTCH1,SMAD3,SOD2,SP1,TCF4,TCF7L2,TLR4,VEGFA,WNT5A
IL-17A Signaling in Airway Cells	4.66E+00	CCL20,CXCL1,CXCL3,CXCL5,CXCL6,GSK3B,IL6,MAPK13,NFKBIA,PIK3R1
Granulocyte Adhesion and Diapedesis	4.42E+00	CCL20,CCL26,CCL28,CXCL1,CXCL14,CXCL2,CXCL3,CXCL5,CXCL6, CXCL8,ICAM1,IL1RN,IL33,IL36B,MMP1,MMP16,MMP3
TR/RXR Activation	4.33E+00	DIO2,F10,ME1,NCOA2,NCOA3,PFKP,PIK3R1,PPARGC1A,SLC16A3, SLC2A1,THRB
Agranulocyte Adhesion and Diapedesis	4.04E+00	CCL20,CCL26,CCL28,CXCL1,CXCL14,CXCL2,CXCL3,CXCL5,CXCL6, CXCL8,ICAM1,IL1RN,IL33,IL36B,MMP1,MMP16,MMP3
GP6 Signaling Pathway	3.55E+00	ADAM10,COL15A1,COL18A1,COL23A1,COL27A1,COL5A1,COL6A6, GSK3B,LAMA4,LYN,PIK3R1,PRKCD
Colorectal Cancer Metastasis Signaling	3.53E+00	CTNNB1,GNG12,GSK3B,IL6,MMP1,MMP16,MMP3,PGF,PIK3R1,PTGER3, PTGS2,SMAD3,TCF4,TCF7L2,TLR2,TLR3,TLR4,VEGFA,WNT5A
Putrescine Degradation III	3.46E+00	ALDH1A3,ALDH2,ALDH3A2,IL4I1,SAT1
HOTAIR Regulatory Pathway	3.40E+00	CTNNB1,ICAM1,KMT2C,MET,MMP1,MMP16,MMP3,NFKBIA,PIK3R1, TCF4,TCF7L2,TLR4,TWIST2,WNT5A
Aryl Hydrocarbon Receptor Signaling	3.36E+00	AHR,ALDH1A3,ALDH1L2,ALDH2,ALDH3A2,ALDH6A1,ATM, GSTT2/GSTT2B,IL6,NCOA2,NCOA3,RBL1,SP1
Axonal Guidance Signaling	3.34E+00	ACTR2,ADAM10,ADAM12,ADAM33,ADAMDEC1,GNG12,GSK3B,MET, MMP1,MMP16,MMP3,NCK1,NRP2,NTRK2,PAK3,PAPPA,PDGFD,PGF, PIK3R1,PLCB4,PLCE1,PPP3CC,PRKCD,PTCH1,RGS3,SEMA3C,UNC5B, VEGFA,WNT5A
Toll-like Receptor Signaling	3.31E+00	IL1RN,IL33,IL36B,MAPK13,NFKBIA,TLR2,TLR3,TLR4,TNFAIP3

Estrogen Receptor Signaling	3.31E+00	DDX5,EIF4EBP1,GSK3B,LEP,MED4,MMP1,MMP16,MMP3,NCOA2,NCOA3,NR3C1,PGF,PIK3R1,PLCB4,PLCE1,PPARGC1A,PRKCD,PRKDC,SHE,SOD2,SP1,VEGFA
Ethanol Degradation IV	3.26E+00	ACSS3,ALDH1A3,ALDH2,ALDH3A2,CYGB
IL-17A Signaling in Fibroblasts	3.23E+00	CXCL5,GSK3B,IL6,MAPK13,MMP1,NFKBIA
BEX2 Signaling Pathway	3.18E+00	CDH2,CTNNB1,GSK3B,NFKBIA,PGF,PPM1L,TCF4,TCF7L2,VEGFA
IL-17 Signaling	3.14E+00	CXCL1,CXCL5,CXCL8,GSK3B,IL6,MAPK13,MMP3,PIK3R1,PTGS2
Regulation of the Epithelial-Mesenchymal Transition Pathway	3.08E+00	CDH2,CTNNB1,FGF1,GSK3B,HMGA2,ID2,MET,NOTCH3,PDGFD,PIK3R1,SMAD3,TCF4,TCF7L2,TWIST2,WNT5A
Airway Pathology in Chronic Obstructive Pulmonary Disease	3.02E+00	CCL20,CXCL1,CXCL3,CXCL8,FGF1,IL33,IL36B,IL6,LEP,LIF,MMP1
Inhibition of Matrix Metalloproteases	2.97E+00	ADAM10,ADAM12,MMP1,MMP16,MMP3,TFPI2
GŒ±q Signaling	2.95E+00	AGTR1,AVPR1A,GNG12,GSK3B,GYS1,HTR2B,NFKBIA,PIK3R1,PLCB4,PPP3CC,PRKCD,RGS4,RGS7
Xenobiotic Metabolism AHR Signaling Pathway	2.95E+00	AHR,ALDH1A3,ALDH1L2,ALDH2,ALDH3A2,ALDH6A1,GSTT2/GSTT2B,IL6,NCOA2
IL-10 Signaling	2.94E+00	CCR1,IL1RN,IL33,IL36B,IL6,MAPK13,NFKBIA,SP1
Role of Cytokines in Mediating Communication between Immune Cells	2.92E+00	CXCL8,IL1RN,IL24,IL32,IL33,IL36B,IL6
Role of Osteoblasts, Osteoclasts and Chondrocytes in Rheumatoid Arthritis	2.92E+00	CTNNB1,GSK3B,IL1RN,IL33,IL36B,IL6,IL7,MMP1,MMP3,NFKBIA,PIK3R1,PPP3CC,SFRP2,TCF4,TCF7L2,WNT5A
Xenobiotic Metabolism Signaling	2.87E+00	AHR,ALDH1A3,ALDH1L2,ALDH2,ALDH3A2,ALDH6A1,CAMK2D,CES1,CHST2,FMO4,GSTT2/GSTT2B,IL4I1,IL6,MAP3K12,MAPK13,PIK3R1,PPARGC1A,PPM1L,PRKCD
IL-6 Signaling	2.81E+00	CXCL8,CYP19A1,IL1RN,IL33,IL36B,IL6,MAPK13,NFKBIA,PIK3R1,TNFAIP6,VEGFA
HIF1Œ± Signaling	2.80E+00	CAMK2D,EIF4EBP1,HSPA1A/HSPA1B,IL6,MET,MMP1,MMP16,MMP3,PGF,PIK3R1,PPP3CC,PRKCD,SAT1,SLC2A1,VEGFA
Role of Hypercytokinemia/hyperchemokinemia in the Pathogenesis of Influenza	2.74E+00	CCR1,CXCL8,IL1RN,IL33,IL36B,IL6
Cardiac Hypertrophy Signaling (Enhanced)	2.67E+00	ADRB2,AGTR1,ATP2A3,CAMK2D,CTNNB1,CXCL8,EDNRA,EIF4EBP1,FGF1,GSK3B,IL21R,IL27RA,IL33,IL36B,IL6,LEP,LIF,MAP3K12,MAPK13,PDK1,PIK3R1,PLCB4,PLCE1,PPP3CC,PRKCD,PTGS2,WNT5A
Oxidative Ethanol Degradation III	2.64E+00	ACSS3,ALDH1A3,ALDH2,ALDH3A2

FAT10 Cancer Signaling Pathway	2.59E+00	CTNNB1,GSK3B,IL6,NFKBIA,SMAD3,TCF4
Ethanol Degradation II	2.58E+00	ACSS3,ADH1B,ALDH1A3,ALDH2,ALDH3A2
Thyroid Cancer Signaling	2.56E+00	CTNNB1,CXCL8,GSK3B,NTRK2,PDK1,PIK3R1,TCF4,TCF7L2
Fatty Acid Œ±-oxidation	2.55E+00	ALDH1A3,ALDH2,ALDH3A2,PTGS2
Human Embryonic Stem Cell Pluripotency	2.55E+00	CTNNB1,GSK3B,NTRK2,PDGFD,PIK3R1,S1PR1,SMAD3,SPHK1,TCF4,TCF7L2,WNT5A
Prostanoid Biosynthesis	2.54E+00	PTGES,PTGS1,PTGS2
p53 Signaling	2.52E+00	ATM,CTNNB1,GSK3B,MDM4,PIK3R1,PMAIP1,PRKDC,SFN,THBS1
Role of PKR in Interferon Induction and Antiviral Response	2.51E+00	CASP8,HSPA1A/HSPA1B,IL24,MAPK13,NFKBIA,PDGFD,SCARA3,SP1,TLR3,TLR4
Gap Junction Signaling	2.51E+00	CCN3,CTNNB1,GJA3,GJB2,GRIA3,GUCY1A2,HTR2B,PIK3R1,PLCB4,PLCE1,PPP3CC,PRKCD,PRKG2,SP1
MIF-mediated Glucocorticoid Regulation	2.46E+00	MIF,NFKBIA,NR3C1,PTGS2,TLR4
Synaptogenesis Signaling Pathway	2.46E+00	ACTR2,AP2B1,CAMK2D,CDH11,CDH2,COMP,CTNNB1,EIF4EBP1,GRIA3,GSK3B,LYN,NLGN1,NLGN4X,NTRK2,PIK3R1,PRKCD,SHE,STXBP5,THBS1
CD40 Signaling	2.45E+00	ICAM1,MAPK13,NFKBIA,PIK3R1,PTGS1,PTGS2,TNFAIP3
Ovarian Cancer Signaling	2.45E+00	CTNNB1,EDNRA,GSK3B,PGF,PIK3R1,PTGS1,PTGS2,TCF4,TCF7L2,VEGFA,WNT5A
Coagulation System	2.41E+00	F10,PLAT,PLAU,PLAUR,PROS1
Noradrenaline and Adrenaline Degradation	2.41E+00	ADH1B,ALDH1A3,ALDH2,ALDH3A2,IL4I1
Endocannabinoid Cancer Inhibition Pathway	2.36E+00	CASP8,CTNNB1,DDIT3,GSK3B,PGF,PIK3R1,TCF4,TCF7L2,TRIB3,TWIST2,VEGFA
HMGB1 Signaling	2.33E+00	CXCL8,ICAM1,IL33,IL36B,IL6,LEP,LIF,MAPK13,PIK3R1,PLAT,SP1,TLR4
Regulation Of The Epithelial Mesenchymal Transition By Growth Factors Pathway	2.29E+00	CDH2,FGF1,GSK3B,HMGA2,ID2,IL6,MAPK13,MEST,MET,MMP1,PDGFD,PIK3R1,SMAD3
Phenylethylamine Degradation I	2.27E+00	ALDH2,ALDH3A2
Atherosclerosis Signaling	2.26E+00	COL18A1,CXCL8,ICAM1,IL1RN,IL33,IL36B,IL6,MMP1,MMP3,PDGFD
AMPK Signaling	2.21E+00	ADRB2,AK4,EIF4EBP1,GYS1,LEP,MAPK13,PFKFB3,PFKFB4,PFKP,PIK3R1,PPARGC1A,PPM1L,SLC2A1,SMARCA2
Th1 and Th2 Activation Pathway	2.20E+00	CCR1,DLL1,ICAM1,IL24,IL27RA,IL33,IL6,NOTCH3,PIK3R1,RUNX3,S1PR1,STAT4
IL-17A Signaling in Gastric Cells	2.19E+00	CCL20,CXCL1,CXCL8,MAPK13

Tryptophan Degradation X (Mammalian, via Tryptamine)	2.19E+00	ALDH1A3,ALDH2,ALDH3A2,IL4I1
Epithelial Adherens Junction Signaling	2.16E+00	ACTR2,CDH2,CTNNB1,DLL1,FER,FGF1,IQGAP1,MET,NOTCH3,TCF4,TCF7L2
PI3K/AKT Signaling	2.13E+00	CTNNB1,EIF4EBP1,GDF15,GSK3B,GYS1,IL21R,IL27RA,NFKBIA,PIK3R1,PPM1L,PTGS2,SFN
Role of Pattern Recognition Receptors in Recognition of Bacteria and Viruses	2.12E+00	CXCL8,IL33,IL36B,IL6,LEP,LIF,PIK3R1,PRKCD,TLR2,TLR3,TLR4
TREM1 Signaling	2.11E+00	CXCL3,CXCL8,ICAM1,IL6,TLR2,TLR3,TLR4
Phenylalanine Degradation IV (Mammalian, via Side Chain)	2.10E+00	ALDH2,ALDH3A2,IL4I1
Sirtuin Signaling Pathway	2.09E+00	ADAM10,CXCL8,GSK3B,H1-2,NAMPT,NDUFA4L2,NDUFS1,PDK1,PFKFB3,PGK1,PPARGC1A,PPID,PPIF,PRKDC,SLC2A1,SOD2,SP1
Role of IL-17F in Allergic Inflammatory Airway Diseases	2.06E+00	CXCL1,CXCL5,CXCL6,CXCL8,IL6
Th2 Pathway	2.06E+00	CCR1,DLL1,ICAM1,IL24,IL33,NOTCH3,PIK3R1,RUNX3,S1PR1,STAT4
Acute Phase Response Signaling	2.05E+00	CP,IL1RN,IL33,IL36B,IL6,MAPK13,NFKBIA,NR3C1,PIK3R1,SAA2,SOD2,TCF4
NF-Œ∫B Signaling	2.05E+00	CASP8,GSK3B,IL1RN,IL33,IL36B,NFKBIA,NTRK2,PIK3R1,TLR2,TLR3,TLR4,TNFAIP3
LPS/IL-1 Mediated Inhibition of RXR Function	2.05E+00	ALDH1A3,ALDH1L2,ALDH2,ALDH3A2,ALDH6A1,CHST2,FMO4,GSTT2/GSTT2B,IL1RN,IL33,IL36B,IL4I1,PPARGC1A,TLR4
Communication between Innate and Adaptive Immune Cells	2.05E+00	CXCL8,IL1RN,IL33,IL36B,IL6,TLR2,TLR3,TLR4
Bladder Cancer Signaling	2.02E+00	CXCL8,FGF1,MMP1,MMP16,MMP3,PGF,THBS1,VEGFA
Role of Tissue Factor in Cancer	2.02E+00	CXCL1,CXCL8,F10,LYN,MAPK13,MMP1,PIK3R1,PLAUR,VEGFA
Role of Wnt/GSK-3Œ≤ Signaling in the Pathogenesis of Influenza	2.02E+00	CTNNB1,GSK3B,NCOA2,NCOA3,TCF4,TCF7L2,WNT5A
IL-7 Signaling Pathway	2.02E+00	GSK3B,IL7,LYN,MAPK13,MET,PIK3R1,SLC2A1
Protein Kinase A Signaling	1.95E+00	ADD3,AKAP12,CAMK2D,CTNNB1,DUSP4,GNG12,GSK3B,GYS1,H1-2,NFKBIA,PLCB4,PLCE1,PPP3CC,PRKCD,PTCH1,PTGS2,PTPRE,SFN,SMAD3,TCF4,TCF7L2
Neuropathic Pain Signaling In Dorsal Horn Neurons	1.92E+00	CAMK2D,GRIA3,NTRK2,PIK3R1,PLCB4,PLCE1,PRKCD,TAC1

Dopamine Degradation	1.91E+00	ALDH1A3,ALDH2,ALDH3A2,IL4I1
PEDF Signaling	1.91E+00	CASP8,MAPK13,NFKBIA,PIK3R1,SOD2,TCF4,TCF7L2
Mouse Embryonic Stem Cell Pluripotency	1.87E+00	CTNNB1,GSK3B,ID2,LIF,MAPK13,PIK3R1,TCF4,TCF7L2
Histamine Degradation	1.86E+00	ALDH1A3,ALDH2,ALDH3A2
Xenobiotic Metabolism PXR Signaling Pathway	1.83E+00	ALDH1A3,ALDH1L2,ALDH2,ALDH3A2,ALDH6A1,CAMK2D,CES1,CHST2,GSTT2/GSTT2B,IL4I1,PPARGC1A,PRKCD
Clathrin-mediated Endocytosis Signaling	1.81E+00	ACTR2,AP2B1,CTTN,EPS15,FGF1,MET,PDGFD,PGF,PIK3R1,PPP3CC,STON2,VEGFA
Serotonin Degradation	1.79E+00	ADH1B,ALDH1A3,ALDH2,ALDH3A2,CSGALNACT1,IL4I1
Coronavirus Pathogenesis Pathway	1.78E+00	AGTR1,CASP8,CXCL8,DDIT3,IL6,MAPK13,NFKBIA,PTGS2,RBL1,SMAD3
p70S6K Signaling	1.76E+00	AGTR1,F2RL2,LYN,PIK3R1,PLCB4,PLCE1,PPM1L,PRKCD,SFN
Inhibition of Angiogenesis by TSP1	1.72E+00	GUCY1A2,MAPK13,THBS1,VEGFA
Altered T Cell and B Cell Signaling in Rheumatoid Arthritis	1.70E+00	IL1RN,IL33,IL36B,IL6,TLR2,TLR3,TLR4
Neuroinflammation Signaling Pathway	1.67E+00	CASP8,CTNNB1,CXCL8,GSK3B,ICAM1,IL6,KCNJ6,MAPK13,MMP3,PIK3R1,PPP3CC,PTGS2,SOD2,TLR2,TLR3,TLR4
Adipogenesis pathway	1.67E+00	CTNNB1,DDIT3,FGF1,LEP,NR1D2,SMAD3,SOX9,TXNIP,WNT5A
Systemic Lupus Erythematosus In B Cell Signaling Pathway	1.66E+00	CTNNB1,CXCL8,GSK3B,IL33,IL36B,IL6,LEP,LIF,LYN,PAG1,PIK3R1,PPP3CC,PRKCD,SHE,TLR3
Necroptosis Signaling Pathway	1.66E+00	CAMK2D,CASP8,CYLD,MLKL,PPID,PPIF,PPP3CC,RBL1,TLR3,TLR4
Basal Cell Carcinoma Signaling	1.65E+00	CTNNB1,GSK3B,PTCH1,TCF4,TCF7L2,WNT5A
Superoxide Radicals Degradation	1.64E+00	CYGB,SOD2
ErbB Signaling	1.61E+00	GSK3B,MAPK13,NCK1,NRG1,PAK3,PIK3R1,PRKCD
Polyamine Regulation in Colon Cancer	1.55E+00	CTNNB1,SAT1,TCF4
Endothelin-1 Signaling	1.53E+00	CASP8,EDNRA,GUCY1A2,MAPK13,PIK3R1,PLCB4,PLCE1,PRKCD,PTGS1,PTGS2,SHE
Thiamin Salvage III	1.52E+00	TPK1
Xenobiotic Metabolism CAR Signaling Pathway	1.52E+00	ALDH1A3,ALDH1L2,ALDH2,ALDH3A2,ALDH6A1,CHST2,FMO4,GSTT2/GSTT2B,PPARGC1A,PPM1L,PRKCD
Molecular Mechanisms of Cancer	1.50E+00	ATM,CAMK2D,CASP8,CDK18,CTNNB1,GSK3B,MAPK13,NFKBIA,PAK3,PIK3R1,PLCB4,PMAIP1,PRKCD,PRKDC,PTCH1,RBL1,SMAD3,TCF4,WNT5A
VDR/RXR Activation	1.50E+00	IGFBP5,KLF4,NCOA2,NCOA3,PRKCD,SP1
Nitric Oxide Signaling in the Cardiovascular System	1.50E+00	ATP2A3,GUCY1A2,PGF,PIK3R1,PRKCD,PRKG2,VEGFA

Apelin Cardiac Fibroblast Signaling Pathway	1.50E+00	AGTR1,IL6,SPHK1
LXR/RXR Activation	1.50E+00	IL1RN,IL33,IL36B,IL6,PTGS2,SAA2,TLR3,TLR4
IL-15 Production	1.50E+00	DDR1,DDR2,FER,IL6,LYN,MET,NTRK2,ROR1
Th1 Pathway	1.50E+00	DLL1,ICAM1,IL27RA,IL6,NOTCH3,PIK3R1,RUNX3,STAT4
Tumoricidal Function of Hepatic Natural Killer Cells	1.45E+00	CASP8,ICAM1,SRGN
RAR Activation	1.45E+00	ADH1B,ALDH1A3,MAPK13,MMP1,PIK3R1,PPARGC1A,PRKCD,RDH10, SMAD3,SMARCA2,VEGFA
Insulin Secretion Signaling Pathway	1.45E+00	CAMK2D,CLCN3,EIF4EBP1,LYN,MAPK13,PIK3R1,PLCB4,PLCE1,PRKCD ,PRLR,SLC2A1,SSR3,STAT4
Phagosome Formation	1.43E+00	PIK3R1,PLCB4,PLCE1,PRKCD,SCARA3,TLR2,TLR3,TLR4
MIF Regulation of Innate Immunity	1.42E+00	MIF,NFKBIA,PTGS2,TLR4
Intrinsic Prothrombin Activation Pathway	1.42E+00	COL18A1,F10,KLK5,PROS1
Pyrimidine Ribonucleotides Interconversion	1.42E+00	AK4,CMPK2,DHX9,RECQL
Leukocyte Extravasation Signaling	1.41E+00	CTNNB1,CTTN,CYBA,FER,ICAM1,MAPK13,MMP1,MMP16,MMP3,PIK3 R1,PRKCD
Adrenomedullin signaling pathway	1.41E+00	GSK3B,GUCY1A2,IL1RN,IL33,IL36B,MAPK13,PIK3R1,PLCB4,PLCE1,PRK G2,SHE
PPAR Signaling	1.40E+00	IL1RN,IL33,IL36B,NFKBIA,PDGFD,PPARGC1A,PTGS2
Opioid Signaling Pathway	1.40E+00	AP2B1,CAMK2D,CTNNB1,GSK3B,KCNJ6,LYN,NFKBIA,PDK1,PPP3CC,P RKCD,RGS3,RGS4,RGS7
Wnt/Ca+ pathway	1.40E+00	GSK3B,PLCB4,PLCE1,ROR1,WNT5A
Factors Promoting Cardiogenesis in Vertebrates	1.39E+00	CAMK2D,CTNNB1,GSK3B,PLCB4,PLCE1,PRKCD,TCF4,TCF7L2,WNT5A
Sperm Motility	1.39E+00	DDR1,DDR2,FER,GUCY1A2,LYN,MET,NTRK2,PLCB4,PLCE1,PRKCD,PR KG2,ROR1
Regulation Of The Epithelial Mesenchymal Transition In Development Pathway	1.37E+00	CTNNB1,GSK3B,PTCH1,TCF4,TCF7L2,WNT5A
IL-8 Signaling	1.37E+00	CXCL1,CXCL8,EIF4EBP1,GNG12,ICAM1,IQGAP1,PGF,PIK3R1,PRKCD,P TGS2,VEGFA
Glycolysis I	1.36E+00	ALDOC,PFKP,PGK1
Gluconeogenesis I	1.36E+00	ALDOC,ME1,PGK1
White Adipose Tissue Browning Pathway	1.36E+00	DIO2,GUCY1A2,LEP,MAPK13,PPARGC1A,PRKG2,THRB,VEGFA
Pyrimidine Ribonucleotides De Novo Biosynthesis	1.35E+00	AK4,CMPK2,DHX9,RECQL

IL-23 Signaling Pathway	1.35E+00	NFKBIA,PIK3R1,RORA,STAT4
Semaphorin Neuronal Repulsive Signaling Pathway	1.34E+00	GSK3B,GUCY1A2,NRP2,PAK3,PIK3R1,PRKG2,SMC3,VCAN
Ephrin Receptor Signaling	1.31E+00	ACTR2,ADAM10,FGF1,GNG12,NCK1,PAK3,PDGFD,PGF,RGS3,VEGFA
NAD biosynthesis II (from tryptophan)	1.30E+00	QPRT,TDO2
Cell Cycle: G1/S Checkpoint Regulation	1.27E+00	ATM,GSK3B,NRG1,RBL1,SMAD3
Hepatic Cholestasis	1.24E+00	CXCL8,IL1RN,IL33,IL36B,IL6,LEP,LIF,NFKBIA,PRKCD,TLR4
B Cell Receptor Signaling	1.24E+00	CAMK2D,GSK3B,LYN,MAP3K12,MAPK13,NFKBIA,PAG1,PIK3R1,POU2F2,PPP3CC
Graft-versus-Host Disease Signaling	1.24E+00	IL1RN,IL33,IL36B,IL6
Th17 Activation Pathway	1.23E+00	AHR,CCL20,IL21R,IL6,RORA,STAT4
Œ≤-alanine Degradation I	1.22E+00	ALDH6A1
Cysteine Biosynthesis/Homocysteine Degradation	1.22E+00	CBS/CBSL
PI3K Signaling in B Lymphocytes	1.22E+00	CAMK2D,LYN,NFKBIA,PIK3R1,PLCB4,PLCE1,PPP3CC,TLR4
Hematopoiesis from Pluripotent Stem Cells	1.21E+00	CXCL8,IL6,IL7,LIF
Cell Cycle: G2/M DNA Damage Checkpoint Regulation	1.21E+00	ATM,MDM4,PRKDC,SFN
Apelin Endothelial Signaling Pathway	1.21E+00	ICAM1,KLF2,PIK3R1,PLCB4,PRKCD,SMAD3,SP1
Dopamine-DARPP32 Feedback in cAMP Signaling	1.21E+00	ATP2A3,GUCY1A2,KCNJ6,PLCB4,PLCE1,PPM1L,PPP3CC,PRKCD,PRKG2
Role of NFAT in Cardiac Hypertrophy	1.20E+00	CAMK2D,GNG12,GSK3B,IL6,LIF,MAPK13,PIK3R1,PLCB4,PLCE1,PPP3CC,PRKCD
fMLP Signaling in Neutrophils	1.19E+00	ACTR2,GNG12,NFKBIA,PIK3R1,PLCB4,PPP3CC,PRKCD
TNFR1 Signaling	1.19E+00	CASP8,NFKBIA,PAK3,TNFAIP3
DNA Double-Strand Break Repair by Non-Homologous End Joining	1.18E+00	ATM,PRKDC
Sphingosine-1-phosphate Signaling	1.18E+00	CASP8,PDGFD,PIK3R1,PLCB4,PLCE1,S1PR1,SPHK1
Melatonin Signaling	1.17E+00	CAMK2D,PLCB4,PLCE1,PRKCD,RORA
UVB-Induced MAPK Signaling	1.14E+00	EIF4EBP1,MAPK13,PIK3R1,PRKCD
G-Protein Coupled Receptor Signaling	1.13E+00	ADRB2,AGTR1,AVPR1A,CAMK2D,DUSP4,HTR2B,NFKBIA,PIK3R1,PLCB4,PTGER3,RGS4,RGS7,S1PR1

ATM Signaling	1.12E+00	ATM,MAPK13,MDM4,NFKBIA,PPM1L,SMC3
Salvage Pathways of Pyrimidine Ribonucleotides	1.10E+00	AK4,CDA,CDK18,CMPK2,PAK3,PRKCD
Senescence Pathway	1.10E+00	ATM,CXCL8,EIF4EBP1,IL6,PDK1,PIK3R1,PPM1L,PPP3CC,RBL1,SAA2,SMAD3,SOD2,TLR2
Apelin Cardiomyocyte Signaling Pathway	1.09E+00	ATP2A3,MAPK13,PIK3R1,PLCB4,PLCE1,PRKCD
Wnt/Œ≤-catenin Signaling	1.08E+00	CDH2,CTNNB1,GSK3B,PPM1L,SFRP2,SOX9,TCF4,TCF7L2,WNT5A
Granzyme B Signaling	1.08E+00	CASP8,PRKDC
Extrinsic Prothrombin Activation Pathway	1.08E+00	F10,PROS1
Retinoate Biosynthesis I	1.08E+00	ADH1B,ALDH1A3,RDH10
CCR3 Signaling in Eosinophils	1.07E+00	CCL26,GNG12,MAPK13,PAK3,PIK3R1,PLCB4,PRKCD
Uracil Degradation II (Reductive)	1.05E+00	DPYD
Thyronamine and Iodothyronamine Metabolism	1.05E+00	DIO2
Glutathione Biosynthesis	1.05E+00	GCLM
Tetrahydrobiopterin Biosynthesis I	1.05E+00	GCH1
Methionine Salvage II (Mammalian)	1.05E+00	BHMT2
Thymine Degradation	1.05E+00	DPYD
Thyroid Hormone Metabolism I (via Deiodination)	1.05E+00	DIO2
Tetrahydrobiopterin Biosynthesis II	1.05E+00	GCH1
cAMP-mediated signaling	1.05E+00	ADRB2,AGTR1,AKAP12,CAMK2D,DUSP4,PKIA,PPP3CC,PTGER3,RGS4,RGS7,S1PR1
Endocannabinoid Neuronal Synapse Pathway	1.02E+00	GRIA3,KCNJ6,MAPK13,PLCB4,PLCE1,PPP3CC,PTGS2
Renal Cell Carcinoma Signaling	1.01E+00	MET,PAK3,PIK3R1,SLC2A1,VEGFA
IGF-1 Signaling	1.01E+00	CCN3,IGFBP2,IGFBP5,IGFBP7,PIK3R1,SFN
Reelin Signaling in Neurons	1.00E+00	ACTR2,CAMK2D,CDH2,GSK3B,LYN,PDK1,PIK3R1
MSP-RON Signaling Pathway	1.00E+00	KLK5,PIK3R1,TLR2,TLR4
Mitochondrial Dysfunction	0.00E+00	CASP8,LRRK2,NDUFA4L2,NDUFS1,SOD2
Tight Junction Signaling	0.00E+00	CTNNB1,F2RL2,PPM1L,SPTAN1

Regulation of Actin-based Motility by Rho	0.00E+00	ACTR2,PAK3
Caveolar-mediated Endocytosis Signaling	0.00E+00	ITGA7,ITGA8
FcŒ≥RIIB Signaling in B Lymphocytes	0.00E+00	LYN,PIK3R1
Cytotoxic T Lymphocyte-mediated Apoptosis of Target Cells	0.00E+00	CASP8
IL-9 Signaling	0.00E+00	PIK3R1
Virus Entry via Endocytic Pathways	0.00E+00	AP2B1,PIK3R1,PRKCD
CNTF Signaling	0.00E+00	PIK3R1
CDK5 Signaling	0.00E+00	MAPK13,NTRK2,PPM1L
Maturity Onset Diabetes of Young (MODY) Signaling	0.00E+00	APOL6
Melanocyte Development and Pigmentation Signaling	0.00E+00	PAX3,PIK3R1
DNA Methylation and Transcriptional Repression Signaling	0.00E+00	H4C8
Androgen Signaling	0.00E+00	GNG12,NCOA2,PRKCD,SMAD3
Growth Hormone Signaling	0.00E+00	PIK3R1,PRKCD
Melanoma Signaling	0.00E+00	PIK3R1
Type I Diabetes Mellitus Signaling	0.00E+00	CASP8,MAPK13,NFKBIA
Primary Immunodeficiency Signaling	0.00E+00	RFXAP
Type II Diabetes Mellitus Signaling	0.00E+00	NFKBIA,PIK3R1,PRKCD
Chronic Myeloid Leukemia Signaling	0.00E+00	PIK3R1,RBL1,SMAD3
Non-Small Cell Lung Cancer Signaling	0.00E+00	PIK3R1
ERK5 Signaling	0.00E+00	LIF,SFN
G Beta Gamma Signaling	0.00E+00	GNG12,KCNJ6,PRKCD
Systemic Lupus Erythematosus Signaling	0.00E+00	IL1RN,IL33,IL36B,IL6,LYN,PIK3R1
Cdc42 Signaling	0.00E+00	ACTR2,GSK3B,IQGAP1,MAPK13,PAK3
FAK Signaling	0.00E+00	PAK3,PIK3R1
EIF2 Signaling	0.00E+00	DDIT3,GSK3B,PIK3R1,TRIB3,VEGFA

Retinoic acid Mediated Apoptosis Signaling	0.00E+00	CASP8
Hereditary Breast Cancer Signaling	0.00E+00	ATM,PIK3R1,SFN,SMARCA2
RhoA Signaling	0.00E+00	ACTR2,CIT,NRP2
Phospholipase C Signaling	0.00E+00	AHNAK,GNG12,LYN,PLCB4,PLCE1,PPP3CC,PRKCD
Regulation of eIF4 and p70S6K Signaling	0.00E+00	EIF4EBP1,MAPK13,PIK3R1,PPM1L
B Cell Development	0.00E+00	IL7
Breast Cancer Regulation by Stathmin1	0.00E+00	ADRB2,AGTR1,AVPR1A,CAMK2D,CCR1,EDNRA,F2RL2,GNG12,HTR2B, PGF,PIK3R1,PLCB4,PPM1L,PRKCD,PTGER3,S1PR1,VEGFA
Role of MAPK Signaling in the Pathogenesis of Influenza	0.00E+00	MAPK13,PTGS2
Antiproliferative Role of TOB in T Cell Signaling	0.00E+00	SMAD3
OX40 Signaling Pathway	0.00E+00	NFKBIA
Assembly of RNA Polymerase II Complex	0.00E+00	TAF9B
Role of JAK2 in Hormone-like Cytokine Signaling	0.00E+00	PRLR
Actin Nucleation by ARP-WASP Complex	0.00E+00	ACTR2,NCK1
NGF Signaling	0.00E+00	MAP3K12,PIK3R1,PRKCD
RhoGDI Signaling	0.00E+00	ACTR2,CDH11,CDH2,GNG12,PAK3
Telomerase Signaling	0.00E+00	PIK3R1,PPM1L,SP1
Heparan Sulfate Biosynthesis	0.00E+00	CHST2,XYLT2
Heparan Sulfate Biosynthesis (Late Stages)	0.00E+00	CHST2
D-myo-inositol-5-phosphate Metabolism	0.00E+00	PIP4P2,PLCB4,PLCE1,PLPP7
Estrogen Biosynthesis	0.00E+00	CYP19A1
tRNA Charging	0.00E+00	WARS2
D-myo-inositol (1,4,5,6)-Tetrakisphosphate Biosynthesis	0.00E+00	PLPP7
Superpathway of Inositol Phosphate Compounds	0.00E+00	PIK3R1,PIP4P2,PLCB4,PLCE1,PLPP7

D-myo-inositol (3,4,5,6)-tetrakisphosphate Biosynthesis	0.00E+00	PLPP7
3-phosphoinositide Degradation	0.00E+00	INPP4B,PIP4P2,PLPP7
3-phosphoinositide Biosynthesis	0.00E+00	PIK3R1,PLPP7
Triacylglycerol Biosynthesis	0.00E+00	PLPP2
Stearate Biosynthesis I (Animals)	0.00E+00	BDH2
GŒ±s Signaling	0.00E+00	ADD3,ADRB2,GNG12
Oxidative Phosphorylation	0.00E+00	NDUFS1
PCP pathway	0.00E+00	WNT5A
Nucleotide Excision Repair Pathway	0.00E+00	RAD23B
SAPK/JNK Signaling	0.00E+00	DUSP4,MAP3K12,PIK3R1
PTEN Signaling	0.00E+00	GSK3B,NTRK2,PIK3R1
IL-2 Signaling	0.00E+00	PIK3R1
GABA Receptor Signaling	0.00E+00	AP2B1
IL-4 Signaling	0.00E+00	NR3C1,PIK3R1
Neurotrophin/TRK Signaling	0.00E+00	NTRK2,PIK3R1
Glutamate Receptor Signaling	0.00E+00	GRIA3
BMP signaling pathway	0.00E+00	MAPK13
Phagosome Maturation	0.00E+00	CTSC,CTSH,CTSS
PD-1, PD-L1 cancer immunotherapy pathway	0.00E+00	GSK3B,PIK3R1,SMAD3
Sumoylation Pathway	0.00E+00	NFKBIA,NR3C1,SP1
NER Pathway	0.00E+00	H4C8,PRIM2,RAD23B
SPINK1 General Cancer Pathway	0.00E+00	IL6,PIK3R1
Systemic Lupus Erythematosus In T Cell Signaling Pathway	0.00E+00	CASP8,IL6,LEP,PDK1,PIK3R1,PPM1L,PPP3CC,SP1
Inhibition of ARE-Mediated mRNA Degradation Pathway	0.00E+00	MAPK13,PPM1L,SFN
Kinetochore Metaphase Signaling Pathway	0.00E+00	H2AC18/H2AC19,KNTC1,SMC3

Calcium Signaling	0.00E+00	ASPH,ATP2A3,CAMK2D,GRIA3,PPP3CC

Supplementary Table S7. IPA canonical pathways affected in SS lymphoblasts

Ingenuity Canonical Pathways	p-value	Molecules
FAK Signaling	2.95121E-05	ARHGEF6,ARHGEF7,ASAP1,CAPN3,GIT2,ITGB1,MAPK1,MAPK3, PIK3C2A,PIK3C3,PIK3R5,PTEN,PTK2,SOS2,VCL,WAS
Ephrin B Signaling	7.58578E-05	ABI1,CAP1,CBL,CDC42,CFL2,GNA13,GNG2,GNG7,MAPK1,MAPK3, PTK2,ROCK1,VAV2
Molecular Mechanisms of Cancer	7.94328E-05	ADCY3,ARHGEF6,ARHGEF7,ATM,BAX,BMPR1A,BMPR2,CBL,CDC42, CDK16,CDK9,CTNND1,E2F4,E2F7,E2F8,GNA13,ITGB1,JAK1,LEF1,LRP5, MAP2K6,MAPK1,MAPK3,MAPK8,MAX,NLK,PA2G4,PIK3C2A,PIK3C3, PIK3R5,PRKCH,PTK2,RAPGEF1,RASA1,RHOT1,SMAD2,SOS2,TCF4, TGFBR1
Reelin Signaling in Neurons	0.000125893	APP,ARHGEF6,BLK,CDC42,CNR2,HCK,ITGB1,MAP2K6,MAP4K1, MAPK1,MAPK3,MAPK8,NDEL1,PDK3,PIK3C2A,PIK3C3,PIK3R5, RAPGEF1
B Cell Receptor Signaling	0.00025704	APBB1IP,CD22,CDC42,CFL2,IGHG1,MALT1,MAP2K6,MAPK1,MAPK3, MAPK8,NFAT5,PAG1,PIK3C2A,PIK3C3,PIK3R5,PTEN,PTK2,PTPN6, RPS6KB1,SOS2,SYNJ1,VAV2
Huntington's Disease Signaling	0.000275423	BAX,BET1L,CAPN3,GNG2,GNG7,GOSR1,HDAC8,IGF1,MAPK1,MAPK3, MAPK8,NAPB,NAPG,PIK3C2A,PIK3C3,PIK3R5,PRKCH,PSME2,PSMF1, RASA1,RCOR1,SOS2,STX16,UBD,VAMP2,ZDHHC17
T Cell Receptor Signaling	0.000346737	CBL,GRAP2,LCP2,MALT1,MAPK1,MAPK3,MAPK8,NFAT5,PAG1, PIK3C2A,PIK3C3,PIK3R5,RASA1,SOS2,VAV2
Paxillin Signaling	0.000467735	ACTN4,ARHGEF6,ARHGEF7,CDC42,GIT2,ITGAX,ITGB1,MAPK1, MAPK8,PIK3C2A,PIK3C3,PIK3R5,PTK2,SOS2,VCL
EGF Signaling	0.00047863	JAK1,MAPK1,MAPK3,MAPK8,PIK3C2A,PIK3C3,PIK3R5,RASA1,RPS6KB1 SOS2
DNA Double-Strand Break Repair by Non-Homologous End Joining	0.000512861	ATM,DCLRE1C,LIG4,MRE11,XRCC1
Pyridoxal 5'-phosphate Salvage Pathway	0.000549541	ADPGK,DYRK1A,GRK5,HIPK1,MAP2K6,MAPK1,MAPK3,MAPK8,POMK PRKCH,PRKX
Glioma Signaling	0.00057544	E2F4,E2F7,E2F8,IDH3G,IGF1,MAPK1,MAPK3,PA2G4,PIK3C2A,PIK3C3, PIK3R5,PRKCH,PTEN,RBL2,SOS2
Role of BRCA1 in DNA Damage Response	0.000831764	ATM,BLM,BRIP1,E2F4,E2F7,E2F8,MRE11,POU2F1,RBL2,RFC3,SMARCA2, SMARCE1
JAK/Stat Signaling	0.000831764	JAK1,MAPK1,MAPK3,PIAS1,PIK3C2A,PIK3C3,PIK3R5,PTPN6,SOCS4, SOS2,STAT5B,STAT6
Chronic Myeloid Leukemia Signaling	0.000891251	E2F4,E2F7,E2F8,HDAC8,MAPK1,MAPK3,PA2G4,PIK3C2A,PIK3C3, PIK3R5,RBL2,SOS2,STAT5B,TGFBR1
Tec Kinase Signaling	0.000912011	BLK,CDC42,GNA13,GNG2,GNG7,HCK,ITGB1,JAK1,MAPK8,PIK3C2A, PIK3C3,PIK3R5,PRKCH,PTK2,RHOT1,STAT5B,STAT6,VAV2,WAS
Insulin Receptor Signaling	0.000977237	CBL,JAK1,MAPK1,MAPK3,MAPK8,PIK3C2A,PIK3C3,PIK3R5,PPP1R12A, PPP1R7,PTEN,RAPGEF1,RPS6KB1,SOS2,STXBP4,SYNJ1,VAMP2
Growth Hormone Signaling	0.001047129	IGF1,MAPK1,MAPK3,PIK3C2A,PIK3C3,PIK3R5,PRKCH,PTPN6,RPS6KB1, SOCS4,STAT5B

IL-4 Signaling	0.00144544	HLA-DOB,IL4R,JAK1,NFAT5,PIK3C2A,PIK3C3,PIK3R5,PTPN6,RPS6KB1,SOS2,STAT6,SYNJ1
PAK Signaling	0.001513561	ARHGEF6,ARHGEF7,CDC42,CFL2,ITGB1,MAPK1,MAPK3,MAPK8,PIK3C2A,PIK3C3,PIK3R5,PTK2,SOS2
Pancreatic Adenocarcinoma Signaling	0.001548817	CDC42,E2F4,E2F7,E2F8,JAK1,MAPK1,MAPK3,MAPK8,PA2G4,PIK3C2A,PIK3C3,PIK3R5,SMAD2,TGFBR1
Salvage Pathways of Pyrimidine Ribonucleotides	0.001659587	ADPGK,AICDA,DYRK1A,GRK5,HIPK1,MAP2K6,MAPK1,MAPK3,MAPK8,NME4,POMK,PRKCH,PRKX
Integrin Signaling	0.001737801	ACTN4,ARHGEF7,ASAP1,CAPN3,CDC42,ITGAX,ITGB1,MAPK1,MAPK3,MAPK8,PIK3C2A,PIK3C3,PIK3R5,PPP1R12A,PTEN,PTK2,RAPGEF1,RHOT1,ROCK1,SOS2,VCL,WAS
Erythropoietin Signaling	0.001819701	CBL,MAPK1,MAPK3,PIK3C2A,PIK3C3,PIK3R5,PRKCH,PTPN6,RPS6KB1,SOS2,STAT5B
Acute Myeloid Leukemia Signaling	0.002137962	IDH3G,LEF1,MAP2K6,MAPK1,MAPK3,PIK3C2A,PIK3C3,PIK3R5,RPS6KB1,SOS2,STAT5B,TCF4
IL-7 Signaling Pathway	0.002290868	BAX,IGHG1,JAK1,MAPK1,MAPK3,PIK3C2A,PIK3C3,PIK3R5,PTK2,SOS2,STAT5B
PTEN Signaling	0.002290868	BMPR1A,BMPR2,CBL,CDC42,FGFR1,ITGB1,MAPK1,MAPK3,PIK3R5,PTEN,PTK2,RPS6KB1,SOS2,SYNJ1,TGFBR1
Cell Cycle: G1/S Checkpoint Regulation	0.002290868	ATM,BTRC,E2F4,E2F7,E2F8,HDAC8,MAX,PA2G4,RBL2,RPL5
Senescence Pathway	0.002454709	ASXL1,ATM,BMPR2,CAPN3,DMTF1,E2F4,E2F7,E2F8,EED,MAP2K6,MAPK1,MAPK3,MAPKAPK5,MRE11,NFAT5,PDHX,PDK3,PIK3C2A,PIK3C3,PIK3R5,PTEN,RBL2,SMAD2,TBK1,TGFBR1,ZFP36L1
IL-3 Signaling	0.002511886	JAK1,MAPK1,MAPK3,PIK3C2A,PIK3C3,PIK3R5,PRKCH,PTPN6,RAPGEF1,STAT5B,STAT6
Ephrin A Signaling	0.002691535	CDC42,CFL2,PIK3C2A,PIK3C3,PIK3R5,PTK2,ROCK1,VAV2
IGF-1 Signaling	0.002884032	IGF1,JAK1,MAPK1,MAPK3,MAPK8,PIK3C2A,PIK3C3,PIK3R5,PTK2,RASA1,RPS6KB1,SOCS4,SOS2
Role of Tissue Factor in Cancer	0.003019952	BLK,CDC42,CFL2,GNA13,HCK,ITGB1,MAPK1,MAPK3,PIK3C2A,PIK3C3,PIK3R5,PTEN,RPS6KB1,STAT5B
FcŒ≥ Receptor-mediated Phagocytosis in Macrophages and Monocytes	0.003467369	CBL,CDC42,FYB1,HCK,LCP2,MAPK1,MAPK3,PRKCH,PTEN,RPS6KB1,VAV2,WAS
Cholesterol Biosynthesis I	0.003548134	DHCR7,LSS,MSMO1,SC5D
Cholesterol Biosynthesis II (via 24,25-dihydrolanosterol)	0.003548134	DHCR7,LSS,MSMO1,SC5D
Cholesterol Biosynthesis III (via Desmosterol)	0.003548134	DHCR7,LSS,MSMO1,SC5D
Estrogen Receptor Signaling	0.003890451	ADCY3,CFL2,GNA13,GNG2,GNG7,IGF1,JAK1,MAPK1,MAPK3,MED1,

		MED12,MED13,MED13L,MED15,MED20,MED21,NRIP1,PIK3C2A,PIK3C3 PIK3R5,PLCL2,PPP1R12A,PRKAB1,PRKCH,PTEN,ROCK1,RPS6KB1,SOS2 ,ZDHHC21
IL-8 Signaling	0.003981072	BAX,CDC42,GNA13,GNG2,GNG7,IRAK3,IRAK4,ITGAX,MAPK1,MAPK3, MAPK8,PIK3C2A,PIK3C3,PIK3R5,PRKCH,PTK2,RAB11FIP2,RHOT1, ROCK1,RPS6KB1
IL-2 Signaling	0.004073803	JAK1,MAPK1,MAPK3,MAPK8,PIK3C2A,PIK3C3,PIK3R5,SOS2,STAT5B
TGF-Œ≤ Signaling	0.004073803	BMPR1A,BMPR2,CDC42,MAP2K6,MAP4K1,MAPK1,MAPK3,MAPK8, SMAD2,SMURF2,SOS2,TGFBR1
FGF Signaling	0.004073803	FGFR1,FRS2,MAP2K6,MAPK1,MAPK3,MAPK8,PIK3C2A,PIK3C3,PIK3R5, PTPN6,SOS2
Insulin Secretion Signaling Pathway	0.004168694	ADCY3,AGO3,AGO4,BET1L,GOSR1,HCK,JAK1,MAPK1,MAPK3,NAPB, NAPG,PDHX,PIK3C2A,PIK3C3,PIK3R5,PLCL2,PRKCH,RPS6KB1,SEC61A 2,STAT5B,STAT6,STX16,VAMP2
p53 Signaling	0.004786301	ATM,BAX,CCNK,MAPK8,MED1,PIAS1,PIK3C2A,PIK3C3,PIK3R5,PTEN, ST13,TP53INP1
ILK Signaling	0.004897788	ACTN4,ARHGEF6,CDC42,CFL2,FLNA,ITGB1,LEF1,MAP2K6,MAPK1, MAPK3,MAPK8,PIK3C2A,PIK3C3,PIK3R5,PPP1R12A,PTEN,PTK2,RHOT1 VCL
Actin Cytoskeleton Signaling	0.004897788	ACTN4,ARHGEF6,ARHGEF7,CDC42,CFL2,DIAPH2,FLNA,GNA13,ITGB1 MAPK1,MAPK3,PIK3C2A,PIK3C3,PIK3R5,PPP1R12A,PTK2,ROCK1,SOS2, VAV2,VCL,WAS
Coronavirus Pathogenesis Pathway	0.005011872	BAX,E2F4,E2F7,E2F8,IRF9,JAK1,MAPK1,MAPK3,MAPK8,PA2G4,RBL2, RPS20,RPS27L,TBK1,TGFBR1,TNPO2
UVB-Induced MAPK Signaling	0.005128614	MAPK1,MAPK3,MAPK8,PIK3C2A,PIK3C3,PIK3R5,PRKCH,RPS6KB1
Rac Signaling	0.005370318	CD44,CDC42,CFL2,ITGB1,MAPK1,MAPK3,MAPK8,PIK3C2A,PIK3C3, PIK3R5,PIP4K2B,PTK2,RPS6KB1
GDNF Family Ligand-Receptor Interactions	0.005888437	CDC42,FRS2,MAPK1,MAPK3,MAPK8,PIK3C2A,PIK3C3,PIK3R5,RASA1, SOS2
Neurotrophin/TRK Signaling	0.005888437	CDC42,FRS2,MAP2K6,MAPK1,MAPK3,MAPK8,PIK3C2A,PIK3C3,PIK3R5, SOS2
SAPK/JNK Signaling	0.006606934	CDC42,GNA13,GNG2,GNG7,MAP4K1,MAP4K5,MAPK8,MINK1,PIK3C2 A,PIK3C3,PIK3R5,SOS2
IL-22 Signaling	0.007079458	JAK1,MAPK1,MAPK3,MAPK8,STAT5B
Natural Killer Cell Signaling	0.007079458	CD226,CDC42,CFL2,IL18R1,IRAK4,ITGB1,LCP2,LILRB1,MAPK1,MAPK3, NFAT5,PIK3C2A,PIK3C3,PIK3R5,PTPN6,ROCK1,SOS2,VAV2,WAS
Endocannabinoid Cancer Inhibition Pathway	0.007585776	ADCY3,CNR2,GNA13,LEF1,MAP2K6,MAPK1,MAPK3,NSMAF,PIK3C2A, PIK3C3,PIK3R5,PRKAB1,PTK2,ROCK1,TCF4
Fc Epsilon RI Signaling	0.007762471	GRAP2,LCP2,MAP2K6,MAPK1,MAPK3,MAPK8,PIK3C2A,PIK3C3, PIK3R5,PRKCH,SOS2,SYNJ1,VAV2
Geranylgeranyldipho sphate Biosynthesis	0.007762471	COX10,FNTB
FLT3 Signaling in Hematopoietic Progenitor Cells	0.008317638	CBL,MAPK1,MAPK3,PIK3C2A,PIK3C3,PIK3R5,RPS6KB1,SOS2,STAT5B, STAT6

Role of JAK family kinases in IL-6-type Cytokine Signaling	0.008317638	JAK1,MAPK1,MAPK3,MAPK8,STAT5B
Protein Ubiquitination Pathway	0.00851138	BTRC,CBL,DNAJB14,DNAJC15,DNAJC24,MED20,PAN2,PSMA3,PSMB6, PSMD10,PSMD12,PSMD5,PSMD8,PSME2,SACS,SMURF2,UBD,UBE2A, UBE2R2,UBR1,USP16,USP25,USP3,USP8
Signaling by Rho Family GTPases	0.008709636	ARHGEF6,ARHGEF7,CDC42,CFL2,GNA13,GNG2,GNG7,ITGB1,MAPK1, MAPK3,MAPK8,PIK3C2A,PIK3C3,PIK3R5,PIP4K2B,PPP1R12A,PTK2, RHOT1,ROCK1,SEPTIN10,SEPTIN6,WAS
CNTF Signaling	0.008912509	CNTF,JAK1,MAPK1,MAPK3,PIK3C2A,PIK3C3,PIK3R5,RPS6KB1
Role of JAK1 and JAK3 in Œ≥c Cytokine Signaling	0.009120108	IL4R,JAK1,MAPK1,MAPK3,PIK3C2A,PIK3C3,PIK3R5,STAT5B,STAT6
Systemic Lupus Erythematosus In B Cell Signaling Pathway	0.009332543	CBL,CD22,CD72,CNTF,HCK,IGHG1,IRAK4,IRF9,JAK1,MALT1,MAPK1, MAPK3,NFAT5,PAG1,PIK3C2A,PIK3C3,PIK3R5,PRKCH,PTPN6,SOS2, SYNJ1,TBK1,TNFSF4,TRAF5
ErbB Signaling	0.009549926	CDC42,MAP2K6,MAPK1,MAPK3,MAPK8,PIK3C2A,PIK3C3,PIK3R5, PRKCH,RPS6KB1,SOS2
T Cell Exhaustion Signaling Pathway	0.009772372	BMPR2,HLA-DOB,HLA-DPB1,IRF9,JAK1,MAPK1,MAPK3,MAPK8,MGAT5,NFAT5,PDCD1LG2, PIK3C2A,PIK3C3,PIK3R5,PTPN6,SMAD2,TGFBR1
Adipogenesis pathway	0.01	ARNTL,BMPR1A,BMPR2,CLOCK,DGKD,EGR2,FGFR1,GTF2H1,HDAC8, MNAT1,RBBP4,RPS6KB1,STAT5B,TXNIP
Semaphorin Signaling in Neurons	0.012022644	CDC42,CFL2,ITGB1,MAPK1,MAPK3,PTK2,RHOT1,ROCK1
Endometrial Cancer Signaling	0.012022644	LEF1,MAPK1,MAPK3,PIK3C2A,PIK3C3,PIK3R5,PTEN,SOS2
Phagosome Maturation	0.012302688	ATP6V0A1,BET1L,CTSC,DCTN4,DYNC1LI2,GOSR1,NAPB,NAPG,PIK3C3 RAB5B,STX16,TSG101,TUBG2,VAMP2,VPS39
Glioblastoma Multiforme Signaling	0.012302688	CDC42,E2F4,E2F7,E2F8,IGF1,LEF1,MAPK1,MAPK3,PIK3C2A,PIK3C3, PIK3R5,PLCL2,PTEN,RHOT1,RPS6KB1,SOS2
FAT10 Signaling Pathway	0.012302688	MAP1LC3B,PSME2,PSMF1,UBD
HGF Signaling	0.012589254	CDC42,ITGB1,MAPK1,MAPK3,MAPK8,PIK3C2A,PIK3C3,PIK3R5,PRKCH PTK2,RAPGEF1,SOS2
3-phosphoinositide Biosynthesis	0.012882496	NUDT5,PAWR,PIK3C2A,PIK3C3,PIK3R5,PIP4K2B,PPFIBP2,PPP1R12A,PP P1R7,PPTC7,PTEN,PTPN6,PTPRJ,RASA1,SYNJ1,WBP11
PDGF Signaling	0.013489629	JAK1,MAPK1,MAPK3,MAPK8,PIK3C2A,PIK3C3,PIK3R5,RASA1,SOS2, SYNJ1
VEGF Signaling	0.013803843	ACTN4,MAPK1,MAPK3,PIK3C2A,PIK3C3,PIK3R5,PTK2,PTPN6,ROCK1, SOS2,VCL
Glioma Invasiveness Signaling	0.014454398	CD44,CDC42,MAPK1,MAPK3,PIK3C2A,PIK3C3,PIK3R5,PTK2,RHOT1
Leukocyte Extravasation Signaling	0.014454398	ACTN4,CD44,CDC42,CTNND1,F11R,ITGB1,MAPK1,MAPK8,NCF4, PIK3C2A,PIK3C3,PIK3R5,PRKCH,PTK2,ROCK1,VAV2,VCL,WAS
PKCŒ∏ Signaling in T Lymphocytes	0.015135612	CACNG3,GRAP2,HLA-DOB,LCP2,MALT1,MAPK1,MAPK3,MAPK8,NFAT5,PIK3C2A,PIK3C3, PIK3R5,POU2F1,SOS2,VAV2
IL-15 Signaling	0.015488166	JAK1,MAPK1,MAPK3,PIK3C2A,PIK3C3,PIK3R5,RPS6KB1,STAT5B,STAT6

Germ Cell-Sertoli Cell Junction Signaling	0.016982437	ACTN4,CDC42,CFL2,CTNND1,ITGB1,MAP2K6,MAPK1,MAPK3,MAPK8, PIK3C2A,PIK3C3,PIK3R5,PTK2,RHOT1,TGFBR1,VCL
Macropinocytosis Signaling	0.016982437	ABI1,ACTN4,CDC42,ITGB1,PIK3C2A,PIK3C3,PIK3R5,PRKCH,USP6NL
Aryl Hydrocarbon Receptor Signaling	0.016982437	AHR,ATM,BAX,GSTM2,GSTM4,GSTP1,MAPK1,MAPK3,MAPK8,MED1, NFIB,NRIP1,RBL2,TRIP11
Regulation of IL-2 Expression in Activated and Anergic T Lymphocytes	0.016982437	MALT1,MAPK1,MAPK3,MAPK8,NFAT5,SMAD2,SOS2,TGFBR1,TOB1, VAV2
IL-17A Signaling in Airway Cells	0.017378008	JAK1,MAPK1,MAPK3,MAPK8,PIK3C2A,PIK3C3,PIK3R5,PTEN
GŒ±12/13 Signaling	0.017782794	CDC42,GNA13,LPAR6,MAPK1,MAPK3,MAPK8,PIK3C2A,PIK3C3,PIK3R5 PTK2,RASA1,ROCK1,VAV2
Mouse Embryonic Stem Cell Pluripotency	0.018197009	BMPR1A,BMPR2,JAK1,LEF1,MAPK1,MAPK3,PIK3C2A,PIK3C3,PIK3R5, SOS2,TCF4
Sphingosine-1-phosphate Signaling	0.018620871	ADCY3,CDC42,GNA13,MAPK1,MAPK3,PIK3C2A,PIK3C3,PIK3R5,PLCL2, PTK2,RHOT1,S1PR2
CD40 Signaling	0.019054607	MAP2K6,MAPK1,MAPK3,MAPK8,PIK3C2A,PIK3C3,PIK3R5,TRAF5
ErbB2-ErbB3 Signaling	0.019054607	MAPK1,MAPK3,PIK3C2A,PIK3C3,PIK3R5,PTEN,SOS2,STAT5B
Regulation Of The Epithelial Mesenchymal Transition By Growth Factors Pathway	0.019054607	CDC42,FGFR1,FRS2,JAK1,LATS1,MAP2K6,MAPK1,MAPK3,MAPK8, PIK3C2A,PIK3C3,PIK3R5,SMAD2,SOS2,TGFBR1,TNFSF4,ZEB1
Agrin Interactions at Neuromuscular Junction	0.019498446	ARHGEF6,ARHGEF7,CDC42,GABPA,ITGB1,MAPK1,MAPK3,MAPK8, PTK2
Prostate Cancer Signaling	0.019498446	GSTP1,LEF1,MAPK1,MAPK3,PA2G4,PIK3C2A,PIK3C3,PIK3R5,PTEN, SOS2
PPARŒ±/RXRŒ± Activation	0.020892961	ADCY3,ADIPOR2,BMPR2,CLOCK,MAP2K6,MAPK1,MAPK3,MAPK8, MED1,MED12,NCOA6,PLCL2,PRKAB1,SMAD2,SOS2,STAT5B,TGFBR1
Cholecystokinin/Gastrin-mediated Signaling	0.020892961	CDC42,CREM,GNA13,MAP2K6,MAPK1,MAPK3,MAPK8,PRKCH,PTK2, RHOT1,ROCK1,SOS2
Thyroid Cancer Signaling	0.021379621	IGF1,LEF1,MAPK1,MAPK3,PIK3C2A,PIK3C3,PIK3R5,PTEN,TCF4
Role of IL-17A in Arthritis	0.021379621	MAP2K6,MAPK1,MAPK3,MAPK8,PIK3C2A,PIK3C3,PIK3R5
Cleavage and Polyadenylation of Pre-mRNA	0.021877616	CPSF6,CSTF3,PABPN1
PD-1, PD-L1 cancer immunotherapy pathway	0.021877616	HLA-DOB,HLA-DPB1,JAK1,LATS1,LCP2,PDCD1LG2,PIK3C2A,PIK3C3,PIK3R5,PTEN, STAT5B
CD28 Signaling in T Helper Cells	0.022387211	CDC42,GRAP2,HLA-DOB,LCP2,MALT1,MAPK8,NFAT5,PIK3C2A,PIK3C3,PIK3R5,PTPN6, WAS

Renal Cell Carcinoma Signaling	0.022908677	CDC42,MAPK1,MAPK3,PIK3C2A,PIK3C3,PIK3R5,RAPGEF1,SOS2,UBD
Colorectal Cancer Metastasis Signaling	0.023442288	ADCY3,BAX,CDC42,E2F4,GNG2,GNG7,JAK1,LEF1,LRP5,MAPK1,MAPK3 MAPK8,PIK3C2A,PIK3C3,PIK3R5,RHOT1,SIAH1,SMAD2,SOS2,TCF4, TGFBR1
Thrombin Signaling	0.023988329	ADCY3,ARHGEF6,CDC42,GNA13,GNG2,GNG7,MAPK1,MAPK3, PIK3C2A,PIK3C3,PIK3R5,PLCL2,PPP1R12A,PRKCH,PTK2,RHOT1, ROCK1,RPS6KB1
Galactose Degradation I (Leloir Pathway)	0.024547089	GALK2,GALT
Prolactin Signaling	0.024547089	MAPK1,MAPK3,PIK3C2A,PIK3C3,PIK3R5,PRKCH,SOCS4,SOS2,STAT5B
NF-ŒʃB Signaling	0.024547089	BMPR1A,BMPR2,BTRC,FGFR1,IRAK3,IRAK4,MALT1,MAP2K6,MAPK8, PIK3C2A,PIK3C3,PIK3R5,TAB3,TBK1,TGFBR1,TRAF5
Th2 Pathway	0.025118864	BMPR2,CCR1,HLA-DOB,HLA-DPB1,IL4R,JAK1,PIK3C2A,PIK3C3,PIK3R5,STAT5B,STAT6,TGFBR1, TNFSF4
Polyamine Regulation in Colon Cancer	0.025118864	MAX,PSME2,PSMF1,TCF4
Inhibition of ARE-Mediated mRNA Degradation Pathway	0.025118864	AGO3,AGO4,CNOT2,MAPK1,MAPK3,PABPN1,PSME2,PSMF1,TIA1, TNFSF4,ZFP36,ZFP36L1
Ephrin Receptor Signaling	0.025703958	ABI1,CDC42,CFL2,GNA13,GNG2,GNG7,ITGB1,MAPK1,MAPK3,PTK2, RAPGEF1,RASA1,ROCK1,SDCBP,SOS2,WAS
LPS-stimulated MAPK Signaling	0.02630268	CDC42,MAP2K6,MAPK1,MAPK3,MAPK8,PIK3C2A,PIK3C3,PIK3R5,PRK CH
PEDF Signaling	0.02630268	MAPK1,MAPK3,PIK3C2A,PIK3C3,PIK3R5,ROCK1,TCF12,TCF4,ZEB1
IL-9 Signaling	0.02630268	JAK1,PIK3C2A,PIK3C3,PIK3R5,STAT5B
Epithelial Adherens Junction Signaling	0.026915348	ACTN4,BMPR2,CDC42,CTNND1,FGFR1,LEF1,LMO7,PTEN,RAPGEF1, TCF4,TGFBR1,VAV2,VCL,WAS
Role of CHK Proteins in Cell Cycle Checkpoint Control	0.027542287	ATM,E2F4,E2F7,E2F8,HUS1,MRE11,RFC3
CXCR4 Signaling	0.027542287	ADCY3,CDC42,GNA13,GNG2,GNG7,MAPK1,MAPK3,MAPK8,PIK3C2A, PIK3C3,PIK3R5,PRKCH,PTK2,RHOT1,ROCK1
GM-CSF Signaling	0.028183829	HCK,MAPK1,MAPK3,PIK3C2A,PIK3C3,PIK3R5,SOS2,STAT5B
Hepatic Fibrosis Signaling Pathway	0.028840315	BMPR2,CDC42,FGFR1,FTH1,IRAK3,IRAK4,ITGB1,JAK1,LEF1,LRP5, MAP2K6,MAPK1,MAPK3,MAPK8,PIK3C2A,PIK3C3,PIK3R5,PRKCH, PTEN,PTK2,RHOT1,ROCK1,RPS6KB1,SMAD2,SOS2,TCF4,TGFBR1, TRPM7
TR/RXR Activation	0.030199517	MED1,NCOA6,NXPH2,PFKP,PIK3C2A,PIK3C3,PIK3R5,STRBP,UCP2
Small Cell Lung Cancer Signaling	0.030902954	MAX,PA2G4,PIK3C2A,PIK3C3,PIK3R5,PTEN,PTK2,TRAF5
Hereditary Breast Cancer Signaling	0.030902954	ATM,BLM,DDB2,HDAC8,MRE11,PIK3C2A,PIK3C3,PIK3R5,PTEN,RFC3, SMARCA2,SMARCE1,UBD
UVA-Induced MAPK Signaling	0.030902954	ATM,MAPK1,MAPK3,MAPK8,PARP2,PIK3C2A,PIK3C3,PIK3R5,PLCL2, RPS6KB1
Th1 and Th2 Activation Pathway	0.033113112	BMPR2,CCR1,HLA-DOB,HLA-DPB1,IL18R1,IL4R,JAK1,MAP2K6,PIK3C2A,PIK3C3,PIK3R5,STAT5B,

		STAT6,TGFBR1,TNFSF4
NGF Signaling	0.035481339	BAX,CDC42,MAPK1,MAPK3,MAPK8,PIK3C2A,PIK3C3,PIK3R5,ROCK1,RPS6KB1,SOS2
Endocannabinoid Developing Neuron Pathway	0.037153523	ADCY3,CNR2,GNG2,GNG7,MAP2K6,MAPK1,MAPK3,MAPK8,PIK3C2A,PIK3C3,PIK3R5
Apelin Endothelial Signaling Pathway	0.037153523	ADCY3,GNA13,MAPK1,MAPK3,MAPK8,PIK3C2A,PIK3C3,PIK3R5,PRKAB1,PRKCH,RPS6KB1
Spliceosomal Cycle	0.037153523	AQR,CWC25,DDX39B,MAGOHB,PRPF18,SF1
Estrogen-Dependent Breast Cancer Signaling	0.03801894	DHRS11,IGF1,MAPK1,MAPK3,PIK3C2A,PIK3C3,PIK3R5,STAT5B
Role of Osteoblasts, Osteoclasts and Chondrocytes in Rheumatoid Arthritis	0.038904514	BMPR1A,BMPR2,CBL,IGF1,IL18R1,ITGB1,LEF1,LRP5,MAP2K6,MAPK1,MAPK3,MAPK8,NFAT5,PIK3C2A,PIK3C3,PIK3R5,TCF4,TRAF5
fMLP Signaling in Neutrophils	0.038904514	CDC42,GNG2,GNG7,MAPK1,MAPK3,NFAT5,PIK3C2A,PIK3C3,PIK3R5,PRKCH,WAS
RANK Signaling in Osteoclasts	0.038904514	CBL,MAP2K6,MAPK1,MAPK3,MAPK8,PIK3C2A,PIK3C3,PIK3R5,TRAF5
HOTAIR Regulatory Pathway	0.039810717	AEBP2,AGO3,AGO4,CD44,EED,LEF1,PIK3C2A,PIK3C3,PIK3R5,PTEN,RBBP4,RCOR1,ROCK1,TCF4
Renin-Angiotensin Signaling	0.043651583	ADCY3,MAPK1,MAPK3,MAPK8,PIK3C2A,PIK3C3,PIK3R5,PRKCH,PTK2,PTPN6,SOS2
Melanoma Signaling	0.043651583	MAPK1,MAPK3,PIK3C2A,PIK3C3,PIK3R5,PTEN
Thrombopoietin Signaling	0.044668359	MAPK1,MAPK3,PIK3C2A,PIK3C3,PIK3R5,PRKCH,STAT5B
Docosahexaenoic Acid (DHA) Signaling	0.045708819	APP,BAX,PIK3C2A,PIK3C3,PIK3R5
Antiproliferative Role of Somatostatin Receptor 2	0.046773514	GNG2,GNG7,MAPK1,MAPK3,PIK3C2A,PIK3C3,PIK3R5,PTPN6
IL-1 Signaling	0.046773514	ADCY3,GNA13,GNG2,GNG7,IRAK3,IRAK4,MAP2K6,MAPK1,MAPK8
Phosphatidylcholine Biosynthesis I	0.047863009	CHKA,CHPT1
STAT3 Pathway	0.048977882	BMPR1A,BMPR2,FGFR1,IGF1,IL18R1,IL4R,MAPK1,MAPK3,MAPK8,PTPN6,SOCS4,TGFBR1

Supplementary Table S8. IPA functional pathways affected in SS fibroblasts

Network ID	Genes in Network	Score	Focus Molecules	Top Diseases and Functions
1	ARAP2,DDX5,EHBP1,ENPP4,G3BP1,H1-2, H2AC18/ H2AC19,Histone h2a,IGF2BP3, KCNE4,LAMA4,LDL-cholesterol,LGALS8,LMNA, MAP7,MAPK13,P-TEFb, PRKD2,PRKDC, RAB33A, RADX,RESF1,RNA polymerase II,Rnr,SFN,SLC20A1,SPATA6,SUPT16H, SYNGR1,TCF7L2,TMEM65,TMTC1,UBAP1,VEGFA, WDR36	44	30	Cancer, Nervous System Development and Function, Neurological Disease
2	aldehyde dehydrogenase,aldehyde dehydrogenase (NAD),ALDH1A3,ALDH3A2, ALDH6A1,ANTXR2, CDHE/CDHN,COL27A1,Ctbp,CTNNB1,HHEX,HILPDA,HMG CoA synthase, IFITM1,KCNK1,ME1,MOB1A, Neuropilin,NRP2,RAB7,RAB8B,RASSF4,SEMA3C,SLC16A6,SLC1A5,SLC38A5,ST8SIA1,SYNM,TAOK1,TCF,TDO2 TRAPPC13,TWIST,TWIST2,UGCG	35	26	Lipid Metabolism, Molecular Transport, Small Molecule Biochemistry
3	ADAM10,ADAM12,Alpha Actinin,BEST1,Cadherin, CDH2,COMP,DCLK1,DLL1,GALNT5,Growth factor, Hedgehog,HELLS,HOXA11, Hsp27,KBTBD11,KLF2, MAC,MOXD1,MYCBP2,NEAT1,Notch,PALLD,PARP, PRSS12,PTCH1,RALGPS2,RDH10,SFRP2,SH3D19, SH3PXD2A,SOX9,SRC (family),THBS1,VGLL3	35	26	Cancer, Organismal Injury and Abnormalities, Reproductive System Disease
4	ADAM33,ATM,BOLA2/BOLA2B,BRINP1,C15orf48, CCDC50,CHMP4B,DLST,FSTL1,H2BC8,H4C8,Hif1, HISTONE,Histone h3,IgG2b,IL12 (family), KLF4,KMT2C Immunoglobulin, LGALS3BP,Mitochondrial complex 1, NAMPT,NDUFA4L2,NDUFS1,NHLRC3,OGT,P3H2, PDK1,RAB11FIP1,Secretase gamma,SOD2,Tnf (family), TNFAIP3,TPK1,WARS2	35	26	Free Radical Scavenging, Neurological Disease, Organismal Injury and Abnormalities
5	ACSS3,AMPD3,CHRDL1,COL23A1,Collagen Alpha1, CSGALNACT1,CYGB,DDIT4,Ecm,ENPP5,ERO1A,GEM,GLDN,Growth hormone,IFI27,IGF receptor,Igfbp, IGFBP2,IGFBP5,Integrin alpha 3 beta 1,KIAA1671,LY75, MSC,Mucin, MYOC,NEDD9,PAPPA,Pdi,PDPN,PTGS1, RBMS3,Serine Protease,STC1,TSHZ3,Vegf	33	25	Cell Signaling, Free Radical Scavenging, Small Molecule Biochemistry
6	Ant,AOX1,BAG5,CDKN1C,CG,COL15A1,CP,CSTF2T, CYP19A1,DUSP4,EFEMP1,FSH,GALNT12,GK,HMGA2, Insulin,Lh,MTORC2,P4HA2,PEPCK,PFKFB3,PGK1, PMAIP1,PPI,PRLR,PSIP1,PTPase,PTPRE,REV3L,RFXAP, SCAF11,Sod,SON,TM7SF3,TRPV2	33	25	Cancer, Organismal Injury and Abnormalities, Reproductive System Disease
7	AP1AR,Ap2,Ap2 alpha,AP2B1,ARSG,BDH2, Beta adaptin,Clathrin,Cytokeratin,EIF4EBP1,EPS15, Epsin,FAM107A,FBXO21,FOXN3,Foxo,GCLM,GULP1, Keratin,Keratin II-6,KRT14,KRT15,KRT19,KRT34,KRT6A, KRT6B,KRT7,LTBP2,MCAM,MYRIP,PI3K(complex), PRKAA,RAB27B,STON2, ZNF800	31	24	Cell Morphology, Embryonic Development, Hair and Skin Development and Function
8	ADH1B,ANGPTL4,ATP2A3,C/EBP,CBS/CBSL,CES1, creatine kinase,cytokine,DIO2,DUB,EDEM3,ELOVL7, G0S2,HDL,HDL-cholesterol,LDL,N4BP2L2,NBEA,Nos, Nr1h,NXPE3,PNLIPRP3,PTGES,SAA2,SCARF2,SERINC2S	31	24	Cancer, Organismal Injury and Abnormalities,

	ESTD1,ST7L,triacylglycerol lipase,TRIB3,USP25,USP28, USP33,VLDL-cholesterol,ZFHX4			Reproductive System Disease
9	Actin,ACTR2,Alpha catenin,ANK2,Arp2/3, atypical protein kinase C,CIT,Cofilin,CTTN,Dynamin,EPB41L2, EPB41L3,Erm,F Actin, FAM83H,FER,IQGAP1,LACC1, MATR3,NAA15,NCK1,PCMTD1,PDE4DIP,PIP4P2, PPHLN1,RAI14,RAPH1,Ras homolog,Rock,Spectrin, SPTAN1, SPTBN1,SYNPO,TNIK,ZDBF2	31	24	Cellular Assembly Organization, Function and Maintenance, Nervous System Development and Function
10	ADAMDEC1,Ap1,caspase,CD3,Ck2,CXCL2,DDIT3, HMCN1,ID2,Igm,IL1,KCNMA1,KLK5,LYN,MEOX2, Metalloprotease,Mlc,MMP1,MMP3,NR3C1,NRG1, PGAP1Pka catalytic subunit,PPID, PRKCD,Pro-inflammatory Cytokine,secreted MMP,SLITRK4,SMAD3, SPESP1, SPINK5,TCF4,TPR,TXNIP,ZNF395	31	24	Cellular Movement, Inflammatory Response, Organismal Injury and Abnormalities
11	AK4,ANKRD37,ANKRD44,ANO3,c-Src,C1orf198, CCDC58,CMPK2,Collagen type ix,CSRP2,EGLN, EPSTI1,FMOD,GAS2L3,GDF5,GNL3L,Hif,Immunoglobulin Lambda Light Chain,IRAK1BP1, JUN/JUNB/JUND, KCND3,NDPK,NFkB (complex),peptidase,PLK2, PPARα-RXRα, PRKG2,RAPGEF5,SERPINB13,SERPINB7, SLC16A3,T3-TR-RXR,TNFAIP6,TRIM13,Vacuolar H ATPase	29	23	Nucleic Acid Metabolism, Skeletal and Muscular System Development and Function, Small Molecule Biochemistry
12	AQP1,ATP8A1,ATPase,C8,Casein,CD24,Cr3,DHX9, FBN2GAS1,H2BC5,hemoglobin,Holo RNA polymerase II, HSPA1A/HSPA1B, Icam,ICAM1,IKZF2,MATN2, P glycoprotein,Pde4,Pgk,PIR,PLAG1,PLAU,PSMC2, RHOBTB3,RRAGB,RTTN,SMCHD1,STAT1/3/5 dimer, STAU2,TH2 Cytokine,TRIM16,VWA8,ZMAT3	29	23	Cancer, Embryonic Development, Organismal Injury and Abnormalities]
13	AHNAK,AHNAK2,AURK,CCN4,CDK4/6,CLIC6,CYP26B1, EGFL6,ERK,FGF1,Filamin,FKBP11,gelatinase, Gq-coupled receptor,Has,HAS2, HAS3,p38 Sapk, PAG1,PDLIM4,PLOD2,PMEPA1,QSER1,S100,S100A10, S100A14,S100A7,SERCA,SMAD1/5,SMAD1/5/9, SPATA18SULF2,SYNPO2TMSB4,ZFPM2	28	22	Carbohydrate Metabolism, Drug Metabolism, Small Molecule Biochemistry]
14	AChR,ALDH,ALDH1L2,ALDH2,ALDOC,BCR(complex), BTG1,Calcineurin A,Calcineurin protein(s),CD3 group, CLEC3B,COL6A6,FUBP1, GOLIM4,GSK3B,Laminin (family),MEF2,Na,K -ATPase,NFAT (complex), Nfat (family), NLGN1,PI3K (family),POU2F2,PPP3CC, RPS6KA, SERPINI1,SLC2A1,SLC4A7,SLC6A15,SLC6A8, SLC7A11,SLC7A5,SSR3,UBR7,UTRN	28	22	Developmental Disorder, Neurological Disease, Psychological Disorders
15	20s proteasome,26s Proteasome,AKIRIN1, AMFR,calpain, Cathepsin,CEP126,CH25H,CSTA,CTSC,CTSH,CTSS,Cyclin E,EFS,GCH1,GNG12, Iga,INTERLEUKIN,KCTD15, Lysosomal Protease, MAP1LC3,MCTP1,MSTN,N-cor, NFKBIA,QPRT,RAD23B,SAT1,SCAF4,Scf,SERPINB3, SVIP,TIP60,Ubiquitin,ZNF518A	28	22	Post-Translational Modification, Protein Degradation, Protein Synthesis
16	AFF4,APOL6,CASP8,Caspase 3/7,COL18A1, CSPG,CYP, DNAJC14,FAM13C,Focal adhesion kinase,HORMAD1, HSP,Hsp70,Hsp90,HSPA12B, IFN Beta,IL12 (complex), Interferon alpha, LRPPRC,MED4,mediator,MHC Class II (complex),MIF,NAP1L3,NIM1K,NIPBL,PLAT,PRSS23,	28	22	Hematological System Development and Function, Immune Cell Trafficking, Inflammatory Response

	SMC3,SPON1,Tgf beta,TLR4,TSC22D3, TTC37,VCAN			
17	ADAMTSL4,ADCY,ADD3,ADRB2,AGTR1,AKAP12, Alpha tubulin,BAALC,Calmodulin,CAPZA1, CCR1, CLGN,Creb,GTPase,LEP,LRRK2,MBP,Mek,MID1,Mmp, MTORC1,NIBAN1,NMDA Receptor, p70 S6k,PAX6, PLC,Proinsulin,PTGER3,RAB38, SCN2A,SPAST,STXBP5, TAC1,tubulin,UNC5B	28	22	Cardiac Dysfunction, Cardiovascular Disease, Organismal Injury and Abnormalities
18	alcohol group acceptor phosphotransferase, CEMIP2, DAB2IP,FBN1,GCNT1,GDF15,GJB2, Integrin α, ITGA7, ITGA8,Jnk,LRRC32,MAP2K1/2, MAP3K,MAP3K12, MKK3/6,PAK3,POSTN,PPM1L,R-Smad, RUNX3, Smad2/3,Smad2/3-Smad4,SMOOTH MUSCLE ACTIN, STMN2,TEAD, Tenascin,TGFBR,THSD4,TLCD4,TNXB, Tropomyosin,Troponin t,tubulin (family),VPS36	22	19	Cell-To-Cell Signaling and Interaction, Cellular Assembly and Organization, Tissue Development
19	ALT,ASPH,Cbp/p300,COL14A1,cytokine receptor,GOT, GPCPD1,I kappa b kinase,IFN type 1,IL21R,IL27RA, IRF,JAK,LIF,MANEA, MINDY2,NCR3LG1,NOVA1, Pias,PSG1,PSG3,PSG5,PSG6,PSG9,RAS,Rsk,SCG5,SDK2, STAT,STAT4,STAT5a/b,TLR2/3/4,TMEM30B, transglutaminase,tyrosine kinase	22	19	Cellular Function and Maintenance, Hematological and Reproductive System Development and Function
20	ABHD2,AHR,ATM/ATR,Cdc2,Cyclin A,Cyclin B, Cyclin D,E2f,estrogen receptor,FAM20A,Gsk3,H2BC4 Hdac,histone deacetylase,Histone h4,KNTC1,MCM4, MDM4,NCOA3,OLFML1,PCDHB14,PLIN2,PPARGC1A, Rb,RBBP6,RBL1,RECQL,Rxr,SLC7A8,SMARCA2,SP1, THRB,thymidine kinase,thyroid hormone receptor, VitaminD3-VDR-RXR	22	19	Cell Cycle, Cellular Development, Cellular Growth and Proliferation
21	BGN,C1q,CCL20,CCL28,chemokine,Complement, CXCL1CXCL14,CXCL3,CXCL8,DNA-methyltransferase, Fcgr3, Fibrinogen,HLA-DR,Ifn,Ifn gamma,IL-1R/TLR, IL1RN, IL23,IL32,IL33,IL4I1,IL7,Inflammasome (Nalp1),Asc, Casp1, Casp4),Lfa1,MX1,PRC2, RORA, S100A9,SAA,TH1 Cytokine,Tlr,TLR2, TLR3,WNT5A	20	18	Cellular Movement, Hematological System Development and Function, Tissue Development
22	ADAT2,AKIRIN1,ANKRD13A,ATP2C1,C18orf54, CARMIL1,CXCL8,ELAVL1,Eotaxin,FAM76B,FOXJ3, FOXN3,HNRNPL,KRTAP2-3/KRTAP2-4,LINC00312, PAPOLA,PCAT6,RAB34,RABL2B,RASEF,RDH10, RNF182,SAMD12,SLF2,SOBP,SPTLC3,TAFA5,TM7SF3, TP53INP1,TUT4,WBP2,YBX1,ZDHHC14,ZMAT1,ZNF528	20	18	Cardiovascular Disease, Connective Tissue Disorders, Hematological Disease
23	Akt,AMIGO2,CCN3,Collagen type VI,CYBA,DOCK4,F10, Fibrin,FKHR,H/K/NRAS,INPP4B,IRS,JINK1/2,MAML2, MEG3,Myosin,NANOS1,NCK,NMB,NOTCH3,Pak,Pdgf (complex),Pdgf Ab,PDGF-AA,PDGFDD,PDGFD,PGF, SGMS2,Smad,Sphk,ST3GAL6, STAM2,SUSD5,Timp,VAV	19	17	Cardiovascular Disease, Cellular Movement, Organismal Injury and Abnormalities
24	14-3-3,Adaptor protein,ADRB,APC (complex),BNIP3, CAMK2D,CaMKII,CAVIN2,CDA,CLCN3,cytochrome C, DZIP1,FGFR1OP2,Glycogen synthase,GRIA3,GYS1, Histone H1,Hspg,ICE2,Importin alpha,ITPR,K ATP Channel,Laminin1,Mt,PHACTR3,Pka,PP1 protein complex group,PP2A,PPP1R9A,Ppp2c,RGS7,RRAD, RTN2,SIKE1,SRXN1	19	17	Nervous System Development and Function, Organ Morphology, Tissue Morphology

25	APP,ARFGAP3,ARGLU1,ARL6,BHMT2,BUD23,C11orf87,C4orf46,CALHM6,CD33,CDKAL1,CES2,ELMOD3, FAM126B,HABP2,HNF4A,ICAM3,IFNG,IGSF10,IL22RA1IL26,IL4I1,Oas,PNKD,RBM18,REG1A,RNF24,ROBO3, SBNO2,SCAVENGER receptor CLASS A,SRPK1,TAF9B, TMEM97,ZFP90,ZNF706	17	16	Cell-To-Cell Signaling and Interaction, Inflammatory Disease, Inflammatory Response

Supplementary Table S9. IPA functional pathways affected in SS lymphoblasts

Network ID	Genes in Network	Score	Focus Molecules	Top Diseases and Functions
**1**	ANGEL1,ARHGAP25,ASCC2,CARD8,DIAPH2,DIDO1, HCFC2,HERC3,IKZF5,KDM5C,LACTB2,MBIP,MIS18BP1 NAGK,NFRKB,NRCAM,OR7C2,PATL1,PEMT,PPHLN1, Presenilin,RGS9,RNF26,SGF29,SMC5,SMDT1,TASOR2, TENT4B,TMEM62,UTP18,Vegf,WDR37,WDR74,ZNF143, ZNF33A	45	33	Endocrine System Disorders, Hereditary Disorder, Organismal Injury and Abnormalities
**2**	AGO3,AGO4,ANTXR2,APTX,ARFGAP3,ARGONAUTE, BICD1,CCP110,CENPJ,CEP290,CNOT2,CNTROB,CRTC3 DMAC2L,FBXO22,HMGXB4,KIAA0753,KIZ,LYST, MPHOSPH9,MRPL17,MRPL39,MRPL41,MRPS14,OFD1, PAN3,PIBF1,Pka,PPP2R3C,RNASEH2B,SMG9,TNRC6A, TNRC6B,TSPYL5,VBP1	45	33	Cell Cycle, Cellular Assembly and Organization, DNA Replication, Recombination, and Repair
**3**	ACIN1,ANKRD10,API5,ARID4A,ATP11C,ATP6V0A1, BCCIP,CENPV,CHORDC1,COX15,DPP9,DRG1,FAM98B, GABP,GABPA,GPCPD1,HNRNPH3,Immunoglobulin, MIER2,NSRP1,PHKG2,PIGNPRKAB1,RBBP4,RYBP, SCGB1D2,SINHCAF,ST13,TMEM135,TMEM63A,TMLHE TNPO2,YAF2,YTHDF1,ZFP36L1	45	33	Cardiovascular Disease, Developmental Disorder, Digestive System Development and Function
**4**	APP,BRWD1,C1orf112,CENPI,CHURC1,DCXR,DENND1 C,DTD2,DTWD1,EVI2A,FAM50A,FMNL3,GIMAP5, GIMAP6, GTDC1,Irp,KCNMB2,KLF7,NAA25,PCIF1,Pi3k class III, PRKRIP1,PUS7L,SP140L,T2r,TAS2R20,TAS2R4, TAS2R46,TAS2R50,TCP11L2,TICRR,TIGD1,ZMYM5, ZNF431,ZNF75A	42	32	Hereditary Disorder, Metabolic Disease, Organismal Injury and Abnormalities
**5**	Adaptor protein 1,ADPGK,alcohol group acceptor phosphotransferase,AP1G2,AP1S3,ARL15,CEP83, DCAF10,DPY19L3DYRK1A,Exocyst,GGA2,HAUS2,HIPK 1,KLHL36,MAP2K6,MAPK3,MZT2A,PHLPP1,PIGB, POMK,PRKCH,PRKX,RALGAPA1,RALGAPA2, RALGAPB,RFX7,RIPOR2,SCAF11,TMEM120A,TUBG2, TUBGCP4,UFC1,ZNF507,ZNFX1	42	32	Auditory Disease, Cellular Development, Cellular Growth and Proliferation
**6**	26s Proteasome,APEX1,ATF7IP2,BTRC,C15orf41,CCR1, CREBRF,CX3CR1,EMC4,ETNK1,GALK2,GNA13,Histone h3,IFT46,IFT74, LINS1,MFSD11,MPV17,NHLRC2,NIN, NPIPB4 (includes others),OGA,Proinsulin,PRRC2B, PSMA3,REL,RELCH,RSKR,SELL,SIAH1,SLC41A2, SLC9B2,SPAG9,UBQLN1,UBQLN2	42	32	Developmental Disorder, Hereditary Disorder, Neurological Disease
**7**	ALG13,C18orf21,CEP95,CHKA,CHRNA5,DUSP7,EFL1, ERK,ERLIN1,FAR1,HECTD3,LENG8,MAPK1IP1L, MRPS31,MYEF2,NCF4,NRG(family),PDP2,POP4, RASAL3,RBM4,RBM4B,RhoGap,RPP30,RPP38,SERCA, SON,TEX9,TRAF3IP3TRIM69,TTLL5,WAC,YME1L1, ZNF317,ZZEF1	40	31	Cellular Assembly and Organization, Developmental Disorder, Hereditary Disorder
**8**	AQP9,ARL2BP,ARL5B,ATP23,BAZ2B,CDKL5,CHPT1, CLASRP,DCTN4,EPC1,FADS1,HDGFL3,Histone h2a, HSDL2,Insulin,INTS13, LMBR1,MBTD1,Mlcp,NARS2, NIPSNAP3A,NME4,NUCB2,RFX3,RRAGB,RRAGD, RTL8C,SELENOP,SLC25A16,STXBP4,SYNJ2BP,TIP60,	40	31	Connective Tissue Disorders, Developmental Disorder, Hereditary Disorder

	TRNT1,ZNF280B,ZNF655			
**9**	AKAP8L,AQR,ARGLU1,ATXN10,ATXN2,BCAT1,CHD3, CRYZL1,DDX52,DICER1,DMXL1,DNAJC15,EED,HELZ, IGF2BP1,ITPR,KRR1MAN2A2,MYSM1,NKRF,NMDA Receptor,NOC3L,PRC2,Rnr,RPS27L,SCAF4,SLTM,SMU1, SMYD2,STAU2,TEX10,TIMM23,XPNPEP1,ZCCHC8, ZFC3H1	40	31	Cancer, Organismal Injury and Abnormalities, RNA Post-Transcriptional Modification
**10**	ABI3,ADK,Akt,ARHGEF6/7,ARNTL,CALCOCO1,CCDC 6,CIPC,CLOCK,CRK/CRKL,DEPDC5,FKTN,GIT2, GLIPR1,GOLGA5,IRS,LPXN,MAX,NR2C1,NUP58, PDGFDD,PHF21A,RABGAP1L,RCBTB1,RCOR1, RPRD1A,SOCS4,Tap,TRIP11VPS53,WDFY2,WDR59,ZDH HC20,ZFAT,ZNF175	35	29	Cell Morphology, Cell Signaling, Cellular Assembly and Organization
**11**	ABCA5,ABLIM1,APC (complex),CBX5,CD44,CDK9, Cyclin B,ERCC6,HistoneH1,LATS1,LIN7C,LPGAT1, MED12, MED13, MED21,MLKL,MNAT1,NBPF1 (includes others), RAB35,RASSF2, RNA polymerase II,Secretase gamma, STT3B,TFIIH,TRIP4,TRPM7, UBE2A,VAPA,ZMYM2, ZMYM3,ZNF195, ZNF280D, ZNF45, ZNF561,ZNF649	35	29	Cell Death and Survival, Developmental Disorder, Gene Expression
**12**	Ap1 gamma,AP3S2,BNIP2,CD84,Cdc2,Cdk,COMMD6, DHX35,E2f,E2F4,E2F7,E2F8,EAPP,EFHC1,EPSTI1,ERG28, FUCA1,IL18BP,IL18R1,MAP3K7CL,NFkB (complex), PDLIM1,peptidase,RAB27A, RNF141,RTF1,SCYL2,TAB3, thymidine kinase,TMOD2,TRIM5,TRIM6,USPL1,ZFP64 ZNF83	33	28	Cell Morphology, Organ Morphology, Organismal Injury and Abnormalities
**13**	BCL11A,BNIP3,CDK4/6,CPSF6,CPSF7,CSTF3,EMSY, HNRNPUL1,Importin beta,KLHL6,KPNA,MAP3K, MIRLET7, Nucleoporin,NUFIP2,NUP214,NUP98, P3H2,PHC3,PLAGL2,QSER1,RAS,RBBP6,RBM14,RBM25, RC3H2,RNPS1,SP4,SRSF11,TDRD3,TOP3B,WWP1, YLPM1,ZBTB33,ZNF207	33	28	Infectious Diseases, Organismal Injury and Abnormalities, RNA Post-Transcriptional Modification
**14**	ACBD3,ANKRD46,Arf,ARFGEF1,ARHGDIB,ARL1, ARMH3,ATPaseBLM,C10orf88,C8orf37,CR1,DDX39B, DYRK2,ENTPD1,FBXO21,Fc receptor,KIF20B,MICU2, MORC2,MTPAP, MYO1D,MYO9A, MYO9B, P glycoprotein,PITRM1,Pld,Ras homolog,Rho gdi, RHOBTB3,SLC11A2,TNFRSF19,UFM1, WSB1,XPNPEP3	33	28	Developmental Disorder, Hereditary Disorder, Neurological Disease
**15**	Betacatenin/TCF,Calcineurin protein(s),Calmodulin, CAPN3,CBFA2T2,CTPS2,Cyclin D,DMTF1,DONSON, LEF1, LONRF1, MAGOHB,MBNL2,MBNL3,MCTS1, NLK,Pkg, PLAG1,RCBTB2,RNF138,SPG21,SPIN1, STAMBPL1,STRN3, TCF,TCF/LEF,TCF12,TCF4,THOC2, TMOD1,TXNL4B,VAMP1, WBP2,YPEL2,ZNF652	33	28	Developmental Disorder, Hereditary Disorder, Organismal Injury and Abnormalities
**16**	2-oxoglutarate:oxygen oxidoreductase,ALKBH1,Caspase 3/7,CIRBP, Cyclin A,DPF2,GPATCH8,H4-16,HEATR1, HINFP,HISTONE, histone deacetylase,Histone h4, KDM4C, KDM5B,KDM6A,KMT5B, LRRC37B,LTN1, LUC7L3,MON2, NPAT,PCSK7,PHF8,PIAS1, PRDM2PROSER1,RBL2,SLC15A3,SLC15A4,SMARCA2, SMARCE1,STAT,TET2,TRERF1	33	28	Developmental Disorder, Hereditary Disorder, Neurological Disease

**17**	ACAP1,CCDC186,CHM,CHML,Dgk,DGKA,DGKD,DGKH,ERK1/2,FAM76B,FCGR1A/2A/3A,GDI1,GTPBP1,MMD MOSPD2,MUC13,NADPH oxidase,PALM2AKAP2, PIEZO1,Pki,Rab11,RAB11FIP1,RAB11FIP2,RAB31,RBM26 RRP7BP,Septin,SEPTIN10,SEPTIN6,SH3BGRL,SLC35E1, SNX33,SSBP2,TLR7/8,ZNF544	31	27	Hereditary Disorder, Organismal Injury and Abnormalities, Post-Translational Modification
**18**	Adaptor protein,amylase,ASAP1,CCDC126,CTNND1, Cytochrome bc1,DAPK2,DDX10,ERGIC2,FRS2,GPM6A, GRPEL2,HYPK,Mapk,MFF,MGAT5,Mitochondrial complex 1,NDUFA6,NEMF,NWASP,PXMP2,QKI,Rap, RAPGEF1,RBM23,RNF144A,SLC39A8,Sos,SOS2,SRFBP1, TMEM39A,TPD52,UQCR10,VDAC3,ZNF227	31	27	Developmental Disorder, Hereditary Disorder, Infectious Diseases
**19**	AEBP2,AKAP11,ARIH1,BASP1,CENPC,CK1,COPS2, CSNK1E,CSNK1G2,DCLRE1C,DSN1,DUB,ENaC,FBXW4, Gsk3, H2AF,HERC1KIN,KLHL13,KLHL28,KLHL9, LRCH3,LRP5,MORC3,N-Cadherin, PAN2,PIP, TTC9C,USP16, USP25,USP3, USP8,VLDL cholesterol, Wnt,ZNF234	31	27	Cellular Assembly and Organization, DNA Replication, Recombination, and Repair, Post-Translational Modification
**20**	ABI1,BET1L,CAMK2,CARS2,Caveolin,COG1,COG3,COG6,COG7,DNMBP,ELP4,GARRE1,GOSR1,HECTD4, KRBOX4, N-type Calcium Channel,NAPB,NAPG,Pkc(s), PRMT3,Rab5, SNAP23, Snare,STX16,STX2,STX3,STX5, Syntaxin,Syntaxin1, tubulin (family),VAMP2, VAMP4, VPS45,ZBTB39,ZDHHC13	30	26	Cellular Assembly and Organization, Developmental Disorder, Hereditary Disorder
**21**	Cbp/p300,CCNK,CCNT1,COIL,CWF19L1,CYB5R2, Dishevelled,DPM1,EAF2,Early Elongation,FEZ1,Frizzled, GEMIN7, GTF2H1, Holo RNA polymerase II,ICE2, Igh (family),Importin alpha,KPNA3, KPNA5,MAPK8, P-TEFb,PRPF18,PRPF3,PRPF39,RNF214,SF1, SH3GL1, snRNP, SNRPA1,SUPT5H,TAF1D,TAF2,TAF9,TXNL4A	30	26	Cell Morphology, Gene Expression, RNA Post-Transcriptional Modification
**22**	60S ribosomal subunit,Ap1,BAZ2A,BRWD3,CPNE8, CTDSPL2, EGLN,Eif2,FUBP3,Hif1,INTS4,INTS6,LCOR, LDHA,LIG4,MRI1,NCBP1,NFAT (complex),PA2G4, PABPN1,PARP2,Ppp2c,RFC3,RNA polymerase I, RPL30,RPL31,RPL5,RPL8,Smad,SSX3, TARDBP, UPF2,WBP1L,XRCC1,ZBTB25	30	26	Cell Death and Survival, Protein Synthesis, RNA Damage and Repair
**23**	adenylate kinase,AK2,AK3,Basc,BPTF,BRIP1,BUB1B, C2CD2, CCSER2, Collagen Alpha1,CWC25,CWF19L2, Cytoplasmic Dynein,DENND5B,DHCR7,DNAPK, DYNC1LI2,Dynein,EPS15L1,Filamin,Focal adhesion kinase,HAS2,MLLT10,MRE11,NDEL1,NSD3,RPA, SLC31A1,SMURF2,TOX4,trypsinTXNIP,VEZF1,YARS2, ZNF557	28	25	Cancer, Nucleic Acid Metabolism, Small Molecule Biochemistry
**24**	Actin,ADAMTS12,Alphacatenin,ARHGEF7,Cadherin, CDC42, Collagen type I (complex),DOCK9,Ecm,FAM13B, FAM204A,Fgf,FOXD4L3/FOXD4L6,GTPase,HOMEZ, HSPBAP1,Integrin,KLHL3,LMO7,MAPRE2,Mmp, NDRG1,NDRG3,PCID2,PIEZO2,RABGAP1,Rock,RPS20, SDCBP,SMAD2,THNSL1,UBR1,VCL,VEZT,WNK1	28	25	Cell-To-Cell Signaling and Interaction, Cellular Assembly and Organization, Cellular Function and Maintenance

**25**	Ahr-aryl hydrocarbon-Arnt,ALOX5,CCDC138,CELF2, CLIC2,CLIC6, cytochrome-c oxidase,glutathione peroxidase, glutathione transferase,Glutathione-S-transferase,GSDMB, GST,GSTM2,GSTM4, GSTP1,IL-1R,Jnk,KLC1,KRTAP2-3/KRTAP2-4,LARP4,Ldh (complex),LSS,MB21D2,Mi2, MINDY2,MTA3,Nuclear factor 1, RBM12,RHOT1,TRAK1, TRAPPC10,TRAPPC2, WBP2NL,ZADH2,ZNF417/ZNF587	26	24	Drug Metabolism, Protein Synthesis, Small Molecule Biochemistry
